# Comprehensive Analysis and Identification of Prognostic Biomarkers and Therapeutic Targets Among FAM83 Family Members for Gastric Cancer

**DOI:** 10.3389/fcell.2021.719613

**Published:** 2021-11-19

**Authors:** Tianhao Zhang, Shurong Lai, Yuzhi Cai, Zhixin Huang, Ying Li, Sile Chen, Zhimei Zhang, Zhijun Ye, Xiaoling Lai, Ertao Zhai, Shirong Cai, Jianhui Chen

**Affiliations:** ^1^Center of Gastrointestinal Surgery, The First Affiliated Hospital, Sun Yat-sen University, Guangzhou, China; ^2^Gastric Cancer Center, Sun Yat-sen University, Guangzhou, China; ^3^Laboratory of General Surgery, The First Affiliated Hospital, Sun Yat-sen University, Guangzhou, China; ^4^Department of Hepatobiliary Surgery, Guangzhou Panyu Central Hospital, Guangzhou, China; ^5^Guangdong Provincial Key Laboratory of Microbial Safety and Health, State Key Laboratory of Applied Microbiology Southern China, Institute of Microbiology, Guangdong Academy of Sciences, Guangzhou, China; ^6^Department of Pathology, The First Affiliated Hospital, Sun Yat-sen University, Guangzhou, China

**Keywords:** gastric cancer, FAM83 family members, prognostic biomarker, lasso regression analysis, nomogram prognostic model, immune cell infiltration

## Abstract

**Background:** Gastric cancer (GC) is one of the most common and poor prognosis malignancy in the world. The Family with sequence similarity 83 (FAM83) comprises of eight members of A–H. Accumulating evidence confirmed important roles for FAM83 family in tumorigenesis and progression. However, the prognostic values of FAM83 family in GC still have not been clarified.

**Methods:** ONCOMINE, UALCAN, GEPIA, THE HUMAN PROTEIN ATLAS, Kaplan–Meier Plotter, cBioPortal, DAVID, STRING and TIMER databases and R software were adopted in this study.

**Results:** In this study, we demonstrated that the mRNA levels of FAM83 B/C/D/H were significantly up-regulated in stomach adenocarcinoma (STAD), but the protein level of FAM83G/H were remarkable lowly in STAD. Next, FAM83C/D/G/H were significantly associated with tumor stages in STAD patients. Then, the mutation rate of FAM83 family members in STAD patients was 46%, and the highest mutation rate was FAM83H (23%). Furthermore, the functions of FAM83 family and their 259 co-expression genes were primarily related to Shigellosis, RNA degradation and Ribosome biogenesis in eukaryotes pathway. Besides, we have established the prognostic model of FAM83 family in STAD, including the prognostic model of STAD patients (FAM83C/D/G), STAD with lymph node metastasis (FAM83C/D/G/H) and STAD with ERBB2 high expression (FAM83G/H). FAM83C/D high expression with a poor prognosis, while FAM83G/H high expression with a favorable prognosis of STAD. Additionally, we found that the expression of FAM83C/D/G/H were significantly correlated with the infiltration of six types of immune cells [B cells, CD8^+^T cells, CD4^+^T cells, macrophages and Myeloid dendritic cells (DC)], whereas CD4^+^T cells and Macrophage cells have higher risk scores (HR > 1) when FAM83C lowly expression and FAM83D highly expression. The risk score of NK cells was significantly reduced when FAM83G lowly expression and FAM83H highly expression (HR < 1).

**Conclusion:** These findings suggested that FAM83C/D/G/H might play key roles in STAD tumorigenesis and progression, and FAM83C/D might be risk factors but FAM83G/H might be favorable prognostic factors for STAD patients. In addition, CD4^+^T cells and Macrophage cells may be the promoters of FAM83D in progression of STAD, while NK cells may promote the protective effect of FAM83H on STAD patients.

## Introduction

Gastric cancer (GC) is one of the most common malignant cancers. At present, GC is the fifth most commonly diagnosed cancer (5.6% of total cancer cases) and the fourth most commonly leading cause of cancer deaths (7.7% of total cancer cases) in the world (Sung et al., 2021). Globally, there are more than one million new cases of GC per year, and China accounts for about half of these (Chen et al., 2016; Sung et al., 2021). In recent years, GC diagnosis and treatment technology have developed rapidly, but early diagnosis and treatment strategies remain insufficient. About 90% of patients with GC in China are already at the advanced stage at the first visit, and the overall 5-year survival rate is less than 30% (Allemani et al., 2015). Moreover, the molecular characteristics of stomach adenocarcinoma (STAD) are currently unknown. The lack of effective GC diagnostic methods and reliable biomarkers may be one of the most important reasons for the low survival rate of GC. Therefore, it is necessary to deeply explore the molecular mechanism of GC occurrence and development, clarify the potential molecular network of GC malignant progression, screen suitable biomarkers, and formulate effective diagnosis and treatment measures.

In recent years, some FAM83 family members have been shown to be remarkably upregulated in many cancer types (Snijders et al., 2017). The FAM83 family includes eight members from A to H, and these members are classified according to a highly conserved domain with unknown N-terminal function (DUF1669). However, the C-terminal regions of different family members are obviously inconsistent (Bartel et al., 2016; Snijders et al., 2017; Bozatzi and Sapkota, 2018). Furthermore, accumulating evidence has demonstrated crucial roles of the FAM83 family in tumorigenesis and tumor progression (Snijders et al., 2017). However, the biological role of FAM83 family members in GC remains unclear, and an in-depth understanding of biological and molecular mechanisms is necessary for the development of new effective treatment options.

Initially, Bissell (Lee et al., 2012) and Jackson Laboratories (Cipriano et al., 2012) conducted a preliminary detailed analysis of the FAM83 family and identified FAM83A and FAM83B from different genetic screenings. Subsequently, various studies have gradually confirmed that FAM83 family members play a critical role in cell growth, proliferation, metastasis, and drug resistance (Bartel et al., 2016; Zhou et al., 2019). Furthermore, according to multiple studies, FAM83 family members have shown significant prognostic value in certain tumors in the past few years. For example, FAM83A and B were found to be related to the PI3K and EGFR pathway, making surviving tumor cells resistant to TKI therapy in breast cancer (Cipriano et al., 2013; Bartel and Jackson, 2017), and FAM83B has been identified as a novel oncogene associated with activating CRAF/MAPK signal transduction and driving epithelial cell transformation (Cipriano et al., 2012). Moreover, FAM83D has been identified as a key factor for regulating the invasion and proliferation of ovarian cancer cells and inhibiting autophagy through the PI3K/AKT/m-TOR signaling pathway (Zhu et al., 2019). Moreover, a study confirmed that miR-143 leads to G1/G0 arrest of esophageal squamous cell carcinoma cells by down-regulating the expression of FAM83F (Mao et al., 2016), and reported that FAM83F promoted the biological behavior of thyroid follicular cells by regulating the MAPK and TGF signaling pathways (Fuziwara et al., 2019). Additionally, studies have shown that the up-regulation of FAM83 members is significantly related to the increase of breast tumor grade and the decrease of overall survival (OS) (Cipriano et al., 2014). In addition, compared with normal tissues, FAM83H is highly expressed in human cancer tissues (Sasaroli et al., 2011; Snijders et al., 2017; Chen et al., 2019), which is also related to the poor prognosis of cancers of the uterus (Snijders et al., 2017; Chen et al., 2019), liver (Kim et al., 2017), kidneys (Kim et al., 2019a), and bones (Kim et al., 2019b), but FAM83H is significantly associated with a favorable prognosis of glioma and head and neck cancer (Snijders et al., 2017). Thus, the carcinogenic effects of FAM83 family members in different cancers may be different.

Therefore, a systematic study of the FAM83 family in GC will contribute toward revealing the molecular mechanisms related to the occurrence and development of GC and unearthing new prognostic and therapeutic targets. For example, Zou et al. (2018) confirmed that Linc00324 promotes GC cell proliferation through binding with HuR and stabilizing FAM83B expression. Hussein et al. (2020) confirmed that FAM83H and SCRIB are prognostic indicators of GC and demonstrated that FAM83H and SCRIB participate in the malignant progression of GC by stabilizing β-catenin.

In the present study, we combined available STAD databases to identify and analyze the correlations between FAM83 family members and GC via expression profiles, mutation profile, prognostic model construction, and immune cell infiltration analysis. This study provides further strong evidence regarding the essential role of FAM83 family members in the occurrence and development of GC which might contribute toward unearthing new prognostic and therapeutic targets.

## Materials and Methods

### ONCOMINE

ONCOMINE^1^ is the world’s largest oncogene chip database and integrated data-mining platform, which contains 715 gene expression data sets and data from 86,733 cancer and normal tissues (Rhodes et al., 2004). In this study, the expression levels of the FAM83 family were assessed in STAD tissues relative to their expression in normal tissues, and a *p*-value of 0.05, a fold change of 2, and a gene rank in the top 10% were set as the significance thresholds.

### UALCAN

UALCAN^2^ provides analyses based on The Cancer Genome Atlas (TCGA) (Chandrashekar et al., 2017). In this study, UALCAN was utilized to analyze the expression profiles and prognostic values of FAM83 family members in STAD.

### GEPIA 2.0

GEPIA 2.0^3^ is an analysis tool developed by Peking University that contains the RNA sequence expression data of 9,736 tumor tissues and 8,587 normal tissue samples (Tang et al., 2017). In this study, the “Expression Analysis” module of GEPIA 2.0 was used to perform a differential gene expression analysis of tumor and normal tissues, and prognostic analysis of FAM83 family members in STAD. The Kaplan-Meier curve was used for prognostic analysis and the *p*-value cut-off was 0.05.

### Kaplan-Meier Plotter

The Kaplan-Meier Plotter^4^ can evaluate the impact of 54,000 genes on the survival rate of 21 cancer types, including breast cancer, ovarian cancer, lung cancer, and GC (Nagy et al., 2018). It was used in this study to identify the prognostic value of FAM83 family members in GC.

### cBioPortal

TCGA project has been used to perform the molecular characterization of more than 20,000 primary cancers and matched normal samples covering 33 cancer types. The STAD (TCGA, Firehose Legacy) dataset in TCGA, which includes the data from 478 cases with pathology reports, was used for analysis. cBioPortal^5^ is a comprehensive web resource that can be used to visualize and analyze cancer genomics data. In this study, we analyzed the genomic profiles of FAM83 family members, which contained mutations, copy-number alterations, and mRNA (RNASeq V2 RSEM) and protein expression z-scores with a z-score threshold of 2.0 (Cerami et al., 2012; Gao et al., 2013).

### STRING

STRING^6^ is a database of known proteins and predicted protein–protein interactions (PPI) (Szklarczyk et al., 2019). In this study, we used STRING to develop and construct the PPI network of FAM83 family members and 259 co-expressed genes.

### Database for Annotation, Visualization, and Integrated Discovery 6.8

The Database for Annotation, Visualization and Integrated Discovery (DAVID) 6.8^7^ is a functional annotation website that contributes to the clarification of the biological functions of genes (Huang da et al., 2009a,b). In this study, we used DAVID 6.8 for the Gene Ontology (GO) and Kyoto Encyclopedia of Genes and Genomes (KEGG) pathway enrichment analysis of FAM83 family members and 259 co-expressed genes. GO enrichment analysis included the following three modules: biological process (BP), cell component (CC), and molecular function (MF). The *p*-value cut-off was 0.05.

### TIMER2.0

TIMER,^8^ is a comprehensive resource for the analysis of immune infiltrates across different cancer types and can be used to explore clinical and genomic features of cancers (Li et al., 2017). In this study, we used TIMER 2.0 for Immune cell infiltration analysis.

### The Human Protein Atlas

The Human Protein Atlas^9^ is a comprehensive resource for mapping all human proteins in cells, tissues, and organs through multiple omics technologies, including antibody-based imaging, mass spectrometry-based proteomics, transcriptomics, and systems biology (Uhlen et al., 2015). In this study, we used the database for further protein expression profiling.

### Least Absolute Shrinkage and Selection Operator Regression Analysis

Raw counts of RNA sequencing data and corresponding clinical information from 375 patients with STAD were obtained from TCGA dataset. The Kaplan-Meier survival analysis with log-rank test was also used to compare the survival difference between different groups. The least absolute shrinkage and selection operator (LASSO) regression analysis was performed on the dataset to further determine the best prognostic genes by using the package glmnet in R software (Friedman et al., 2010). The *p*-value in the Kaplan-Meier curve and the hazard ratio (HR) with 95% confidence interval (CI) were generated by the log-rank test and univariate Cox proportional hazard regression.

### Construction and Assessment of the Nomogram

The nomogram model can be used to calculate the recurrence risk of a single patient through the points related to each risk factor. Univariate and multivariate cox regression analysis was performed to identify the appropriate factors for constructing the nomogram. Multivariate Cox regression analysis was adopted to screen independent prognostic factors, including age, gender, pT stage, pN stage, and pM stage. The forest was utilized to display the *p*-value, HR, and 95% CI of each variable by the “forestplot” package in R. A nomogram prognostic model was established based on the results of univariate and multivariate cox regression analysis to predict the 1-, 2-, 3-, and 5- year OS of patients with STAD.

### Immune Cell Infiltration Analysis

The TIMER database and the Immunedeconv, an R package that integrates six of the most advanced algorithms: TIMER, xCell, MCP-counter, CIBERSORT, EPIC, and quanTIseq, were used for immune infiltration analysis. All of the above analysis methods and R packages were implemented using R software version 4.0.3.

## Results

### Expression Level of FAM83 Family Members in Patients With Stomach Adenocarcinoma

**FIGURE 1:**
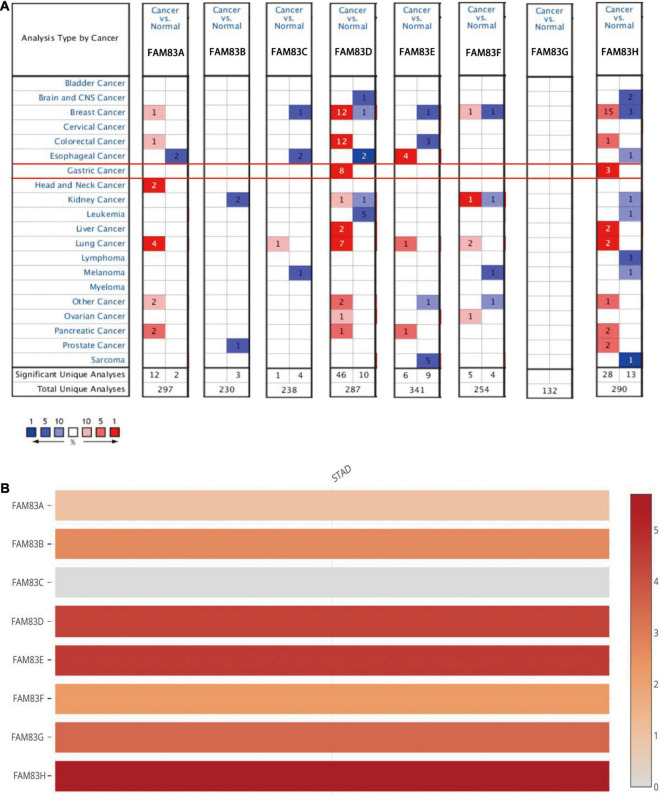
The mRNA expression level of FAM83 family in STAD and other distinct types of cancer (ONCOMINE and GEPIA database). **(A)** This figure shows the number of datasets with statistically significant mRNA up-regulation (red) or down-regulation (blue) in the FAM83 family. The student’s *t*-test was used to compare different transcriptions. The cut-off values of *p*-value and fold change were as follows: *p*-value: 0.05, fold change: 2, gene rank: 10%, data type: mRNA. **(B)** Relative level of FAM83 family members in STAD. FAM83H was the highest mRNA expression in STAD.

In this study, the ONCOMINE database, GEPIA, and UALCAN were used to explore the expression level of FAM83 family members in primary cancer tissues of STAD and normal stomach tissues. The results showed that the relative mRNA expression levels of the FAM83 family in STAD and FAM83D/H were significantly up-regulated in primary cancer tissues in multiple datasets (Figure 1).

In the Chen Gastric dataset (Chen et al., 2003), FAM83D was overexpressed in STAD (*N* = 64) compared with normal stomach tissues (*N* = 28) with a fold change of 2.246 (*p* = 2.23E-15). In the Cho gastric dataset (Cho et al., 2011), FAM83D was overexpressed in STAD (*N* = 20) compared with normal stomach tissues (*N* = 19) with a fold change of 2.410 (*p* = 1.70E-5), while DErrico found a 3.061-fold increase in FAM83D mRNA expression in 26 STAD samples (*p* = 9.09E-12) (D’Errico et al., 2009). In the DErrico Gastric dataset (D’Errico et al., 2009), FAM83H was overexpressed in STAD (*N* = 26) compared with normal stomach tissues (*N* = 31) with a fold change of 3.267 (*p* = 2.84E-13), while Cho JY found a 2.002-fold increase in FAM83H mRNA expression in 31 diffuse gastric adenocarcinoma samples (*p* = 2.03E-5)and a 2.307-fold increase in 10 gastric mixed adenocarcinoma tissues (*p* = 3.42E-4) (Cho et al., 2011).

**FIGURE 2:**
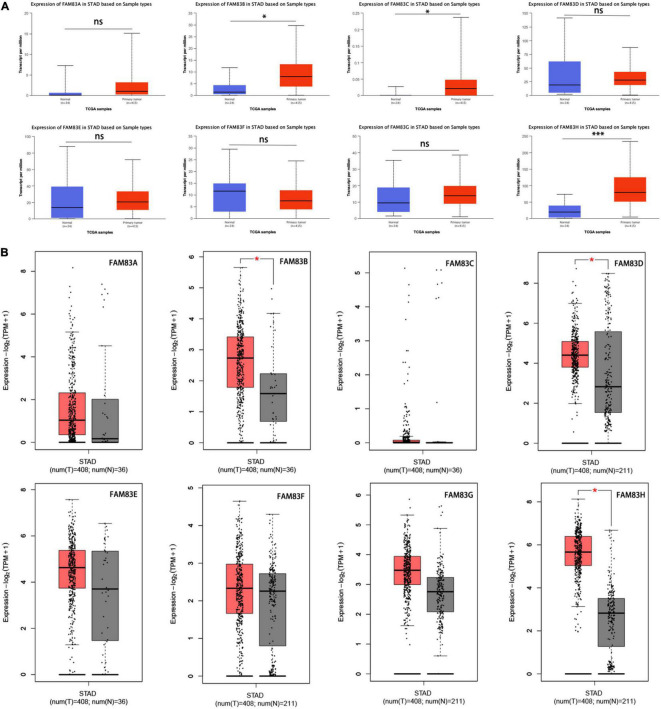
**(A)** The mRNA expressions of FAM83B/C/H were found to be over-expressed in STAD tissues compared to normal samples in UALCAN. ^∗∗∗^*p* < 0.001 and ^∗^*p* < 0.05. **(B)** The mRNA expressions of FAM83B/D/H were discovered to be over-expressed in STAD tissues compared to normal stomach tissues in GEPIA2.0. Significance of difference estimated by Student’s *t*-test and analysis of variance (ANOVA). ^∗^*p* < 0.01.

Furthermore, the UALCAN database was used to compare and show the differential mRNA expression of FAM83 family members between 415 primary tumor tissues and 34 normal tissues. As a result, FAM83B/C/H expression was higher in primary cancer tissues than in normal tissues (Figure 2A). Taken together, we discovered that FAM83B/D/H expression was higher in primary cancer tissues than in normal tissues based on the analysis of GEPIA 2.0 (Figure 2B).

**FIGURE 3:**
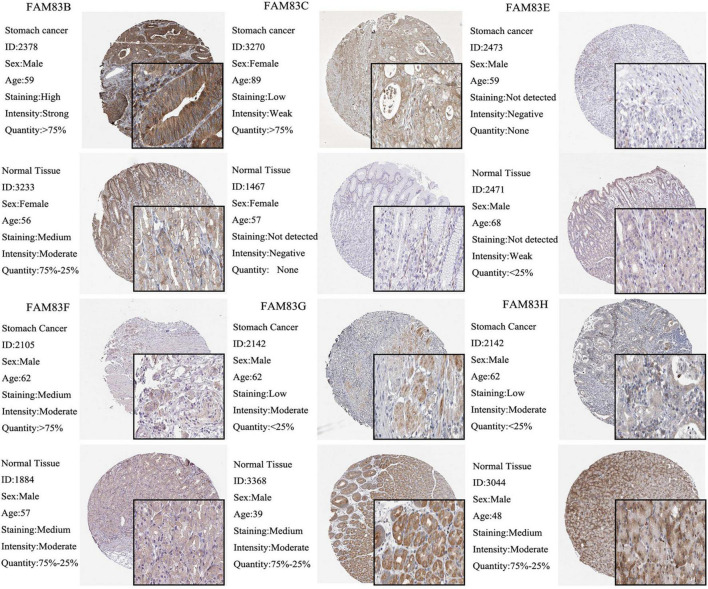
The IHC analysis of FAM83 family with prognostic values. Differentially expressed proteins of FAM83 family members with prognostic values in STAD and normal stomach tissues in The Human Protein Atlas database.

Moreover, we used The Human Protein Atlas database for protein expression profiling. Interestingly, the mRNA level of FAM83G and FAM83H was highly expressed in STAD, while the protein level was significantly lowly expressed in STAD tissues (Figure 3).

### Correlation Between FAM83 Family Members and Individual Cancer Stages of Patients With Stomach Adenocarcinoma

**FIGURE 4:**
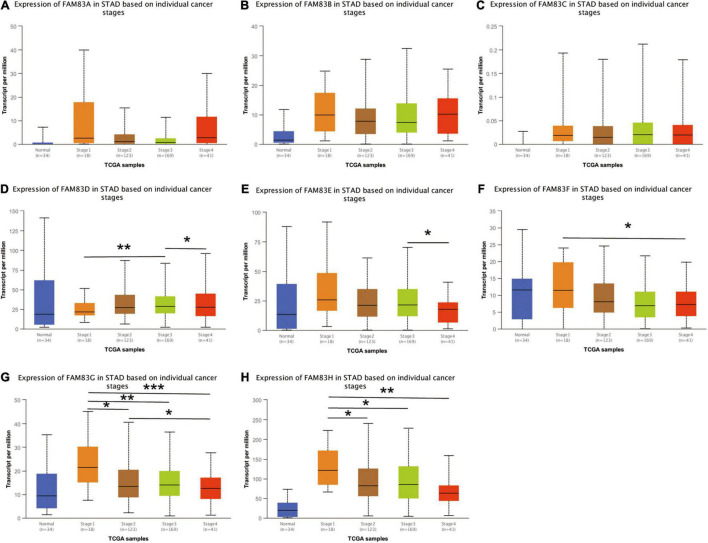
**(A–H)** The correlation between mRNA expression levels of FAM83 family and individual cancer stage in STAD patients (UALCAN). FAM83D/E/F/G/H mRNA expressions were significantly related to patients’ individual cancer stage, while mRNA expressions of FAM83A/B/C were not remarkably associated with patients’ individual cancer stages. Significance of difference estimated by student’s *t*-test considering unequal variance. ^∗^*p* < 0.05, ^∗∗^*p* < 0.01 and ^∗∗∗^*p* < 0.001.

We used the GEPIA and UALCAN database to analyze the correlation between the mRNA expressions of FAM83 family members at different individual tumor stages of patients with STAD. In our results, FAM83D, FAM83E, FAM83F, FAM83G, and FAM83H expression significantly varied in different stages of GC. According to AJCC TNM staging, the mRNA expression level of FAM83D was enhanced in STAD as the stage of the tumor progressed, and stage III (169 samples) and IV (41 samples) showed the highest expression in STAD, while FAM83E, FAM83F, FAM83G, and FAM83H had significant down-regulation in their expression level with the malignant progression of tumors, and had the lowest expression level in stage IV (41 samples) STADs (Figure 4). In summary, the results showed that the level of mRNA expression of FAM83D/E/F/G/H might be related to the individual cancer stages of STAD. In addition, patients with advanced cancer stages were more likely to have higher expression of FAM83D and lower mRNA level of FAM83E/F/G/H.

### Genetic Alterations in the FAM83 Family and Their Correlation With Each Other in Patients With Stomach Adenocarcinoma

**FIGURE 5:**
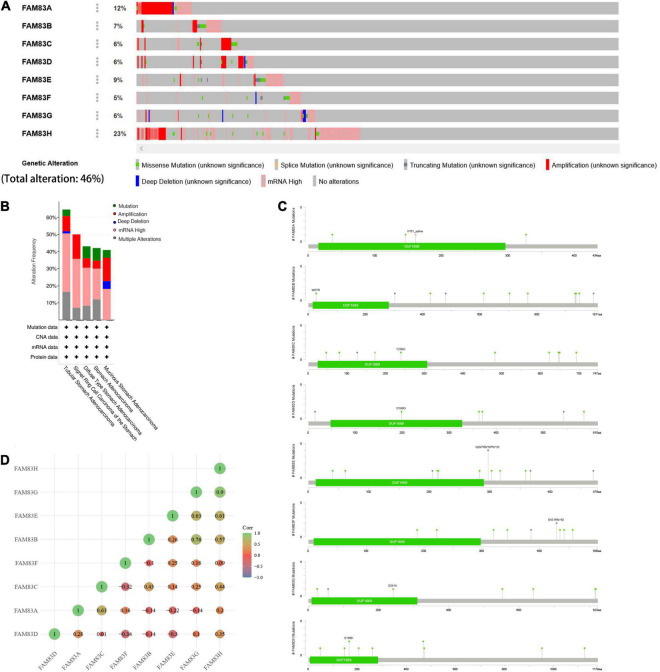
Genetic alterations in FAM83 family members and their correlation with each members in STAD (cBioPortal). **(A,B)** Genetic alterations in different expressed FAM83 family members in STAD. **(C)** Lollipop plot displaying mutation distribution and protein domains for FAM83 family members in STAD with the labeled recurrent hotspots. **(D)** Correlations of different FAM83 family members with each other in STAD.

The cBioPortal was utilized to analyze the genetic alterations and correlations of the FAM83 family in STAD. As a result, FAM83 family members were different in 222 samples from 515 patients with STAD (46%) and were mainly characterized by amplification and high mRNA expression (Figures 5A,B). Among them, FAM83H/A/E/B were the top four genes with higher genetic alterations and mutation probabilities of 23, 12, 9, and 7%, respectively (Figure 5A). As a result, Lollipop plot displaying mutation distribution and protein domains for FAM83 family members in STAD with the labeled recurrent hotspots and all these members have a highly conserved domain—DUF1669 (Figure 5C). Furthermore, we also analyzed the data in TCGA to demonstrate the correlation between FAM83 family members in STAD. The results exposed an obviously positive relationship in the following groups: FAM83G-FAM83H, FAM83B-FAM83G, FAM83E-FAM8G, FAM83A-FAM83C, and FAM83E-FAM83H (correlation coefficient > 0.6). Furthermore, the highest correlation was observed for FAM83G and FAM83H with a correlation coefficient of 0.90. FAM83B and FAM83G were also strongly correlated, and their correlation coefficients was 0.76 (Figure 5D).

### Gene Ontology and Kyoto Encyclopedia of Genes and Genomes Enrichment Analysis

**FIGURE 6:**
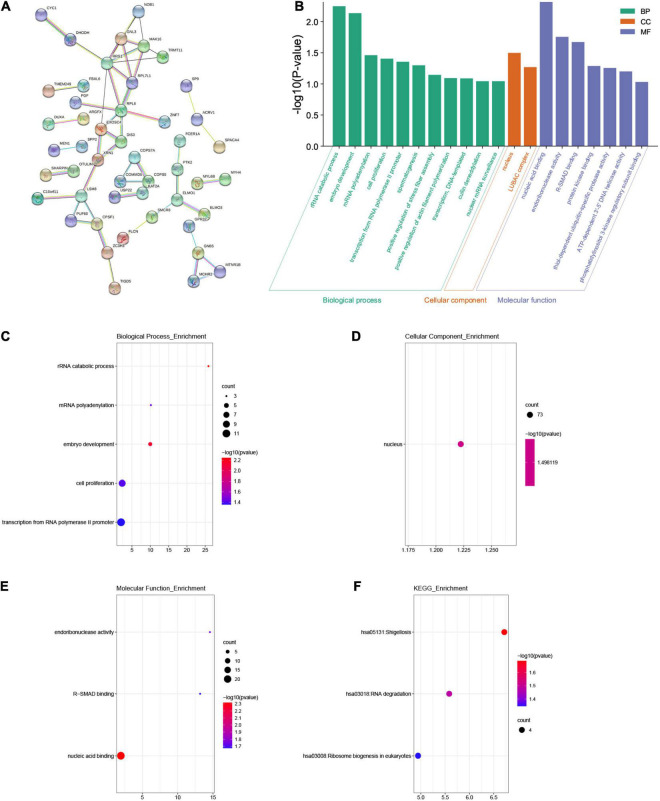
The enrichment analysis of FAM83 family members and their 259 co-expression genes in STAD patients (STRING and DAVID). **(A)** The PPI network with high confidence (0.7) and hide disconnected nodes in the network. The nodes meant proteins, and the edges meant protein interaction. **(B)** Displayed the results of GO enrichment analysis with bar graphs. **(C–E)** BP, CC and MF in GO enrichment analysis. **(F)** KEGG enrichment analysis.

We used the “co-expression” module of cBioportal to analyze 259 co-expressed genes that were significantly related to mutations in the FAM83 family. Next, we constructed an integrated network through the STRING database (Figure 6A). In addition, we used GO and KEGG analyses in DAVID6.8 to explore the potential connection between the FAM83 family and its 259 co-expressed genes. We found that BPs such as GO:0016075 (rRNA catabolic process), GO:0009790 (embryo development), GO:0006378 (mRNA polyadenylation), GO:0008283 (cell proliferation), and GO:0006366 (transcription from RNA polymerase II promoter) were significantly regulated by the genetic alteration of FAM83 family members in STAD. However, only GO:0005634 (nucleus) was greatly associated with the functions of the FAM83 family in the CC module. Moreover, mutations of FAM83 family members also remarkably affected the MF, such as GO:0003676 (nucleic acid binding), GO:0004521 (endoribonuclease activity), and GO:0070412 (R-SMAD binding) (Table 1 and Figures 6B–E). In the KEGG analysis, including hsa05131 (Shigellosis), hsa03018 (RNA degradation) and hsa03008 (ribosome biogenesis in eukaryotes) were remarkably related to the FAM83 family alterations in STAD (Table 2 and Figure 6F).

**TABLE 1:** GO terms associated with FAM83 family members and their 259 co-expression genes in STAD patients (*P* < 0.05).

GO ID	Description	*p*-value	Number of genes
GO:0016075	rRNA catabolic process(BP)	0.005645812	3
GO:0009790	Embryo development(BP)	0.007295419	4
GO:0006378	mRNA polyadenylation(BP)	0.03454626	3
GO:0008283	Cell proliferation(BP)	0.039302518	9
GO:0006366	Transcription from RNA polymerase II promoter(BP)	0.044081045	11
GO:0005634	Nucleus(CC)	0.031760052	73
GO:0003676	Nucleic acid binding(MF)	0.004814467	21
GO:0004521	Endoribonuclease activity(MF)	0.017525156	3
GO:0070412	R-SMAD binding(MF)	0.021221777	3

**TABLE 2:** KEGG enriched pathways associated with FAM83 family members and their 259 co-expression genes in STAD patients (*P* < 0.05).

KEGG ID	Description	*p*-value	Number of genes
Hsa05131	Shigellosis	0.020524468	4
Hsa03018	RNA degradation	0.03315375	4
Hsa03008	Ribosome biogenesis in eukaryotes	0.04507032	4

### Prognostic Value of FAM83 Family Members

**FIGURE 7:**
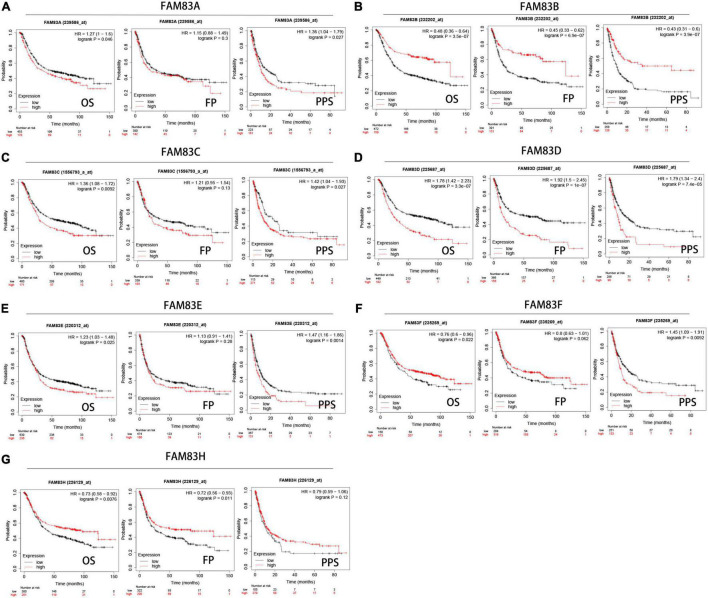
**(A–G)** Prognostic analysis of FAM83 family members in gastric cancer patients (Kaplan–Meier plotter). The Kaplan-Meier plotter database was used to plot OS, FP, and PPS survival curves at the threshold of *p*-value < 0.05 to compare patients with high (red) and low (black) expression of FAM83 family members in gastric cancer.

We analyzed the prognostic value of FAM83 family members in patients with GC by the Kaplan–Meier plotter database, including OS, progression-free survival (FP) and post-progression survival (PPS). The results showed the reverse relationship between mRNA expression levels of FAM83A/C/D/E and OS in patients with GC, but there was a positive relationship between FAM83B/F/G and OS. There was an insignificant correlation between FP and FAM83A/C/E/F in GC; however, higher mRNA expression levels of FAM83B/H led to increased FP and higher FAM83D mRNA expression, which was obviously related to short FP. Furthermore, higher mRNA expression of FAM83A/C/D/E/F was significantly related to the reduction of PPS. Unexpectedly, we did not find a correlation between the FAM83G mRNA level and prognosis (Figure 7).

**FIGURE 8:**
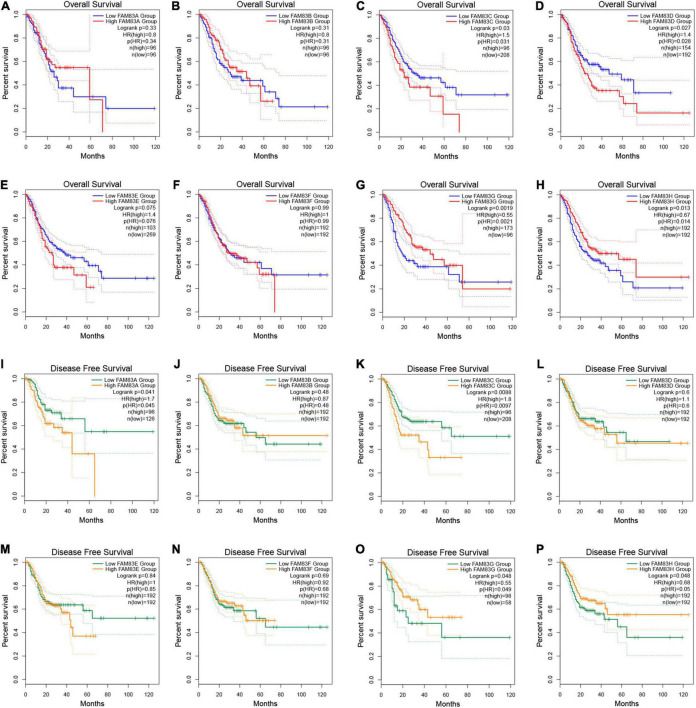
**(A–P)** The prognostic value of FAM83 family members in STAD patients in the overall survival (OS) and disease free survival (DFS) curve (GEPIA2.0).

In addition, we performed prognostic analysis by GEPIA. Based on combined Kaplan–Meier curves with the log-rank *p*-test, FAM83C/D/G/H were obviously associated with OS (*p* < 0.05), and FAM83C/G/H were significantly related to disease-free survival (DFS) (*p* < 0.05) of STAD. Among them, high expression of FAM83C and D might be related to a poor prognosis, but the high mRNA expression level of FAM83G and H might be favorable in STAD (Figure 8).

### Construction and Verification of the Least Absolute Shrinkage and Selection Operator Prognostic Model

In this study, LASSO Cox regression analysis was used to construct the most predictive genes from the FAM83 family as a prognostic model. λ was selected when the median of the sum of squared residuals took the minimum value. As a result, three potential predictors, FAM83C, FAM83D, and FAM83G, were identified as prognostic factors for STAD. The risk score for predicting the prognostic value was constructed with the following formula:


(1) Riskscore=(0.0453)*FAM83C+(0.0348)*FAM83D+(-0.1971)*FAM83G.

**FIGURE 9:**
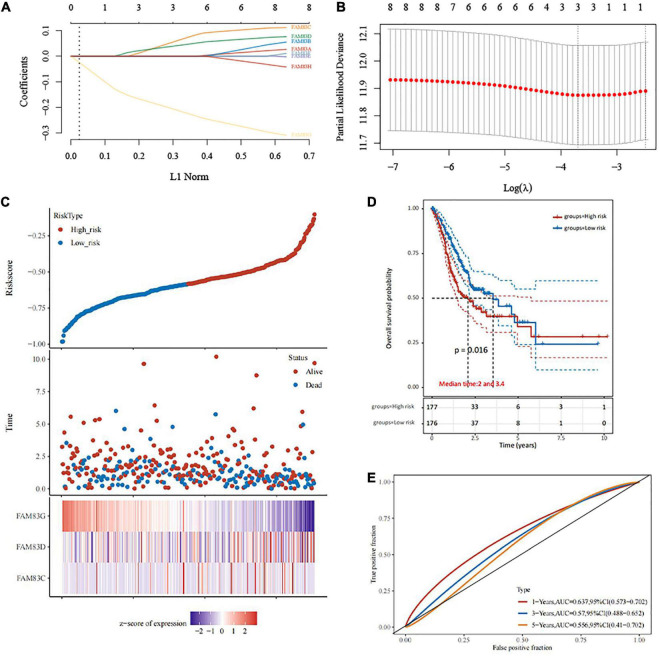
The construction of prognostic model of gene signature from FAM83 family in STAD (TCGA)by LASSO analysis. **(A)** Coefficients of selected features are shown by lambda parameter. **(B)** Partial likelihood deviance vs. log (λ) was drawn using LASSO Cox regression model. **(C)** The relationship between survival status—risk score rank and expression of FAM83 family. **(D)** Kaplan–Meier survival plots of the two groups (high risk and low risk). **(E)** ROC curves of the survival model in STAD.

In this result, patients were divided into high- and low-risk groups based on the combination model of the expression levels of the three candidate genes. We found that the prognosis of the low-risk group was significantly better than that of the high-risk group. We also compared the prognostic efficiency of risk factors through receiver operating characteristic (ROC) curves. Our results indicated that areas under the ROC curve (AUCs) were 0.637, 0.57, and 0.556 in 1, 3, and 5 years, respectively (Figure 9).

Meanwhile, we found that FAM83C, FAM83D, FAM83G, and FAM83H play a crucial role in the prognosis of patients with STAD with lymph node metastasis (pN stage > 0). The risk score for predicting the prognostic value was constructed with the following formula:


(2) Riskscore=(0.1331)*FAM83C+(0.1048)*FAM83D+(-0.1478)*FAM83G+(-0.0552)*FAM83H

The results showed that the AUCs were 0.683, 0.601, and 0.641 in 1, 3, and 5 years respectively (Supplementary Figure 1). Furthermore, FAM83G and FAM83H were identified as prognostic factors for STAD with high ERBB2 expression. The risk score for predicting the prognostic value was constructed with the following formula:


(3) Riskscore=(-0.2736)*FAM83G+(-0.1192)*FAM83H.

The results showed that the AUCs were 0.628, 0.643, and 0.657 in 1, 3, and 5 years, respectively (Supplementary Figure 2).

### Establishment of the Nomogram Prognostic Model

**FIGURE 10:**
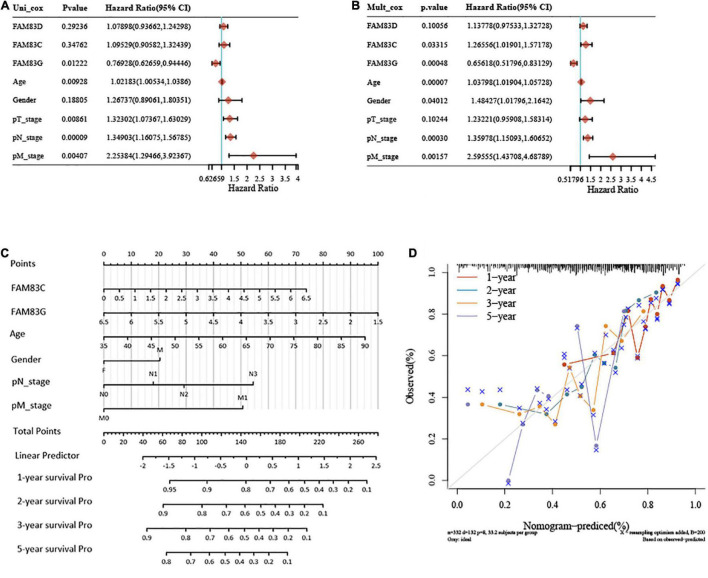
Univariate and multivariate Cox regression analyses and establishment and verification of Nomogram for the FAM83 family genes and clinical risk factors of STAD patients. **(A,B)** Forest plots for hazard ratios (HRs) of survival-associated FAM83 family genes and clinical risk factors in STAD. **(C)** Nomogram to predict the 1-, 2-, 3-, and 5-year overall survival of STAD patients. **(D)** Calibration curve for the overall survival nomogram model in the discovery group. A dashed diagonal line represents the ideal nomogram, and the brown line, blue line, orange line and purple line represent the 1-, 2-, 3-, and 5-year observed nomograms, respectively.

In this study, univariate and multivariate cox regression analyses were performed to identify the appropriate factors to construct the nomogram model, and five high-risk variables of recurrence were discovered as follows: age (hazard ratio, HR = 1.03798), gender (HR = 1.48427), pN stage (HR = 1.35978), pM stage (HR = 2.59555), and FAM83C (HR = 1.26556), and one protective factor: FAM83G (HR = 0.65618). Subsequently, a nomogram was established based on the results of the multivariate Cox proportional hazards analysis, to calculate the risk of recurrence for a patient by the points related to five risk factors (age, gender, pN stage, pM stage, and FAM83C) and one protective factor (FAM83G). The total score was from 0 to 280, and each variable was calculated and merged. A high score indicates a high risk of a poor prognosis. Our results showed that the nomogram (combined model) predicted 1-, 2-, and 3-year survival rates better than 5-year survival rates for their relatively minor deviation between actual and predicted OS (C-index: 0.679, *p* < 0.001) (Figures 10A–C). Then, a calibration plot was constructed by using bootstrap sample corrections to assess the internal validity for the constructed nomogram (Figure 10D), and the results showed that the predicted values had a certain degree of correlation with the actual observed values.

In addition, we constructed a nomogram prognostic model of GC with lymph node metastasis and high expression of ERBB2. We found the following five high-risk variables of recurrence for GC with lymph node metastasis by univariate and multivariate analysis: age (HR = 1.04403), pN stage (HR = 1.40148), pM stage (HR = 2.88206), FAM83C (HR = 1.37216) and FAM83D (HR = 1.20422), and the C-index of the nomogram model was 0.649, *p* < 0.001. Then, we found five high-risk variables of recurrence for GC with high expression of ERBB2: age (HR = 1.05011), pN stage (HR = 1.41825), and pM stage (HR = 2.76747), and the C-index of the nomogram model was 0.681, *p* < 0.001 (Supplementary Figures 3, 4).

### Immune Cell Infiltration of FAM83 Family Members With a Prognostic Value in Patients With Stomach Adenocarcinoma

**FIGURE 11:**
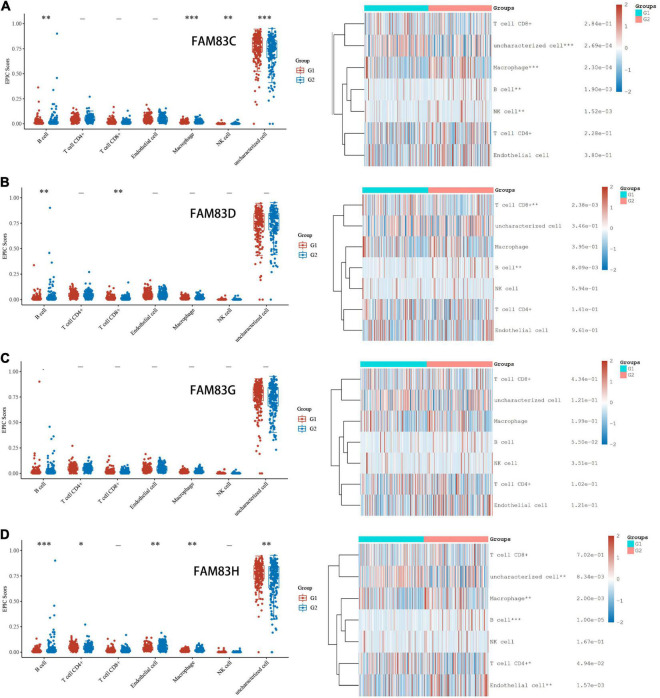
**(A–D)** Immune Cell Infiltration of FAM83 FAM83C/D/G/H in STAD (TCGA). The box diagram on the left represented the score distribution of FAM83C/D/G/H in STAD tissues and normal tissues, where the horizontal axis represents different groups of samples, the vertical axis represents the gene expression distribution, where different colors represent different groups, and the upper left corner represents the significance *p*-value test method. The diagram on the left were immune cell score heat map, where different colors represent the expression trend in different samples, and the significance of two groups of samples passed the Wilcox test. Asterisks represent levels of significance. The asterisk represents the degree of importance (*p < 0.05, **p < 0.01, ***p < 0.001).

**FIGURE 12:**
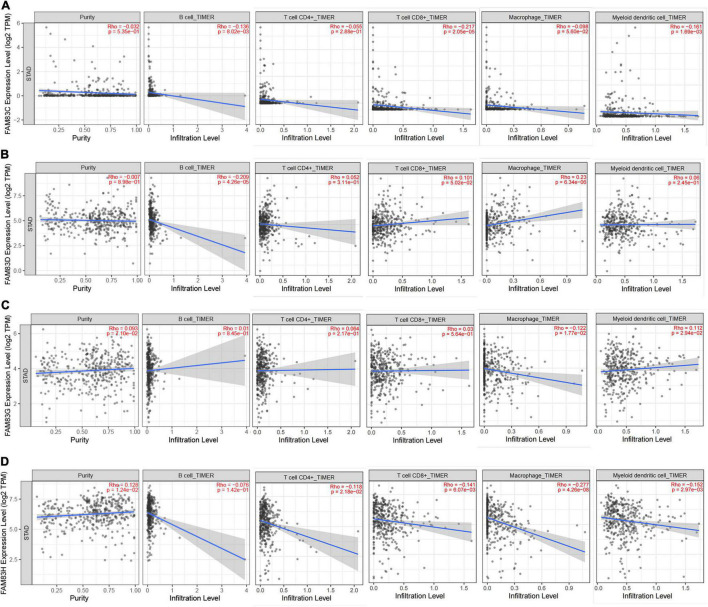
**(A–D)** The correlation between different expressed FAM83 family members and immune cell infiltration (TIMER2.0).

In order to comprehensively explore the relationship between FAM83 family members and immune cells, we performed an immune cell infiltration analysis based on the TIMER database and Immunedeconv R package. As a result, there was a negative correlation between FAM83C expression and the infiltration of B cells (Cor = –0.136, *p* = 8.02e-3), CD8^+^ T cells (Cor = –0.217, *p* = 2.05e-5), and myeloid dendritic cells (DCs) (Cor = –0.161, *p* = 1.69e-3). FAM83D expression was negatively associated with the infiltration of B cells (Cor = –0.209, *p* = 4.26e-6) and positively associated with the infiltration of macrophages (Cor = 0.23, *p* = 6.34e-6). Then, FAM83G expression was negatively associated with the infiltration of macrophages (Cor = –0.122, *p* = 1.77e-2) and positively associated with the infiltration of DCs (Cor = 0.112, *p* = 2.94e-2). In addition, there was a negative correlation between FAM83H expression and the infiltration of CD4^+^ cells (Cor = –0.118, p = 2.18e-2), CD8^+^ T cells (Cor = –0.141, *p* = 6.07e-3), macrophages (Cor =–0.277, *p* = 4.26e-8), and DCs (Cor = –0.152, *p* = 2.97e-3) (Figures 11, 12).

**FIGURE 13:**
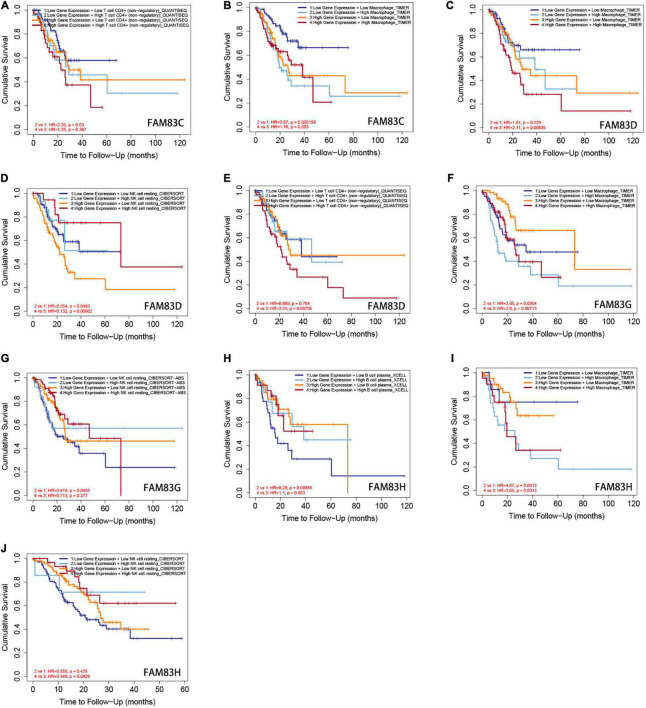
**(A–J)** The Kaplan-Meier curves for the immune infiltrates, expression level of FAM83C/D/G/H and STAD. The infiltration level is divided into low and high levels, and the hazard ratio and *p*-value for Cox model and the log-rank *p*-value for KM curve were shown on the KM curve plot (TIMER2.0).

Furthermore, from the COX regression model analysis of the immune cell infiltration score, we recognized that the abundance of CD4^+^ cells and macrophage infiltration were significantly related to the poor prognosis of patients with GC having low FAM83C expression levels. The abundance of CD4^+^ cells and macrophage infiltration were significantly related to a poor prognosis, and NK cells were significantly related to a poor prognosis of patients with GC with high expression levels of FAM83D. Next, the abundance of macrophage infiltration was significantly associated with a poor prognosis with the expression of FAM83G, but NK cells were significantly related to the favorable prognosis of patients with GC with low expression levels of FAM83G. Then, the abundance of macrophage infiltration was significantly associated with a poor prognosis with the expression of FAM83H, and B cells were significantly related to the favorable prognosis of low expression levels of FAM83H, but NK cells were significantly associated with the favorable prognosis of patients with GC with high expression levels of FAM83H (Figure 13).

## Discussion

Although FAM83 family members have been proven to play a critical role in multiple human cancer types, their biological roles and prognostic value in patients with STAD have rarely been characterized. Eight FAM83 family members share the highly conserved domain with unknown N-terminal function (DUF1669); however, the remaining possess unique C-terminal regions (Fulcher et al., 2018). This also explains the poor enrichment function. In the present study, we first analyzed the mRNA expression, mutation, immunity, and prognostic values of FAM83 family members in STAD.

In recent years, there have been few studies on FAM83C in tumors. According to these studies, FAM83C and FAM83E are up-regulated in bladder and ovarian cancers (Cipriano et al., 2014; Snijders et al., 2017), and FAM83A, FAM83C, FAM83D, and FAM83E have been shown to promote the transformation of human breast epithelial cells (Cipriano et al., 2014). The present study shows that FAM83C is highly expressed in STAD. Furthermore, the higher expression of FAM83C was significantly related to the shortening of OS, DFS, and PPS in patients with STAD and individual cancer stages of STAD. Moreover, the abundance of CD4^+^ cells and macrophage infiltration were significantly related to the poor prognosis of patients with GC with low FAM83C expression levels.

Recently, studies have found that FAM83D is significantly overexpressed in a variety of cancers, including lung cancer, hepatocellular carcinoma, gastric cancer, invasive ovarian cancer, and colorectal cancer (Liao et al., 2015; Mu et al., 2017; Wang et al., 2019; Zhang et al., 2019; Yin et al., 2020). One study discovered that FAM83D knockdown regulates the biological behavior of colorectal cancer by inhibiting the FBXW7/Notch-1 signaling pathway (Mu et al., 2017). Furthermore, studies have found that the mRNA and protein levels of FAM83D are up-regulated in GC tissue and cell lines, and are negatively correlated with the OS and DFS of patients with GC (Wang et al., 2019). In the present study, we found that FAM83D was more highly expressed in STAD than in normal stomach tissues, and a higher mRNA expression level of FAM83D was significantly associated with a shorter OS, FP, and PPS of patients with STAD. Furthermore, FAM83D expression was obviously related to individual cancer stage in the patients with STAD. Then, the abundance of CD4^+^ cells and macrophage infiltration were significantly related to the a poor prognosis, and NK cells were significantly related to a poor prognosis of patients with GC with high expression levels of FAM83D.

We identified few studies assessing the role of FAM83G in tumorigenesis and progression. Okada et al. (2020) demonstrated that FAM83G is a novel inducer of apoptosis, wherein S356 phosphorylation modulates HSP27 phosphorylation and apoptosis regulation, and HSP27 is a counterpart of FAM83G. Then, Vogt et al. (2014) showed that FAM83G is a substrate for type I bone morphogenetic protein (BMP) receptors and modulates BMP signaling, which is known to play an important role in tumorigenesis and metastasis (Yamamoto et al., 2002; Bailey et al., 2007). In this study, interestingly, the mRNA level of FAM83G was highly expressed in GC but was not significant, while its protein level had significantly low expression in GC and was significantly related to the individual cancer stage in patients with STAD. Moreover, the high expression of FAD83G was significantly related to the improvement of OS and DFS in patients with STAD. The abundance of macrophage infiltration was significantly associated with a poor prognosis with expression of FAM83G, but NK cells were significantly related to the favorable prognosis of patients with GC with low expression levels of FAM83G.

Hussein et al. (2020) demonstrated that the individual and combined expression patterns of nuclear FAM83H and SCRIB are prognostic indicators of gastric carcinomas and FAM83H and SCRIB are involved in the progression of gastric carcinomas by stabilizing β-catenin. Another study demonstrated that FAM83H regulates the progression of osteosarcoma by involving mechanisms that stabilize β-catenin and promote proliferation and invasiveness (Kim et al., 2019b). In our study, coincidentally, FAM83H was significantly highly expressed in GC, while its protein level was lowly expressed in GC and significantly related to individual cancer stage in patients with STAD. Furthermore, a high level of FAM83H was significantly associated with better OS, FP, and DFS in patients with STAD. Moreover, the abundance of macrophage infiltration was significantly associated with a poor prognosis with the expression of FAM83H, and B cells were significantly related to a favorable prognosis and low expression levels of FAM83H, but NK cells were significantly associated with a favorable prognosis of patients with GC with high expression levels of FAM83H.

Recently, epigenetic changes have been found to play an increasingly important role in the early stage of malignant tumors (Cancer Genome Atlas Network, 2012). The high somatic mutation rate of fam83 family members in STAD means that it might affect the genetic characteristics of patients with STAD, and is mainly characterized by amplification and high mRNA expression. FAM83H might be one of the potential major mutation factors affecting the occurrence and development of STAD.

At present, immunotherapy has gradually become an important pillar for tumor treatment. It is vital to know more about the metabolic interdependence of infiltrating immune cells and malignancy (Leone and Powell, 2020). In our results, CD4^+^ T cells and macrophages might be poor prognostic factors for STAD, while NK and B cells might be favorable prognostic factors. However, CD4^+^ T cells and macrophages have higher risk scores when FAM83C was lowly expressed and FAM83D was highly expressed. As a result of expression profiling mentioned earlier, FAM83D is expressed significantly higher than FAM83C in GC. Additionally, NK cells have lower risk scores when FAM83G is lowly and FAM83H highly expressed, indicating that NK cells might contribute to the role of FAM83H in prolonging the survival time of patients with STAD.

Lymph node metastasis is the most common metastasis in the malignant progression of GC. In our study, we confirmed that FAM83C/D/G/H play an important role in the prognosis of patients with GC lymph node metastasis. Among them, we found that FAM83C/D were independent prognostic risk factors, and the high expression of FAM83C/D was significantly related to the poor prognosis of patients with GC lymph node metastasis.

In addition, we constructed a prognostic model of FAM83 family members in patients with high ERBB2 expression. Human epidermal growth factor receptor (EGFR)-2 (HER2) is a member of the EGFR tyrosine kinase family that is encoded by ERBB2 (Moasser, 2007). In the ToGA trial, compared with chemotherapy alone, the treatments combining the anti-HER2 monoclonal antibody trastuzumab with chemotherapy showed a significantly favorable prognosis in patients with HER2-positive GC (Bang et al., 2010). However, most HER2-targeting agents have failed to show survival benefits in patients with GC despite demonstrating significant activities in HER2-positive breast cancer. Therefore, these studies indicate that the development of anti-HER2 therapy for HER2-positive GC faces unique challenges. In our study, incredibly, we found that high expression of FAM83G/H was significantly related to the favorable prognosis of patients with GC lymph node metastasis. Therefore, we consider that the prognostic value of FAM83 family members is inconsistent for different types of GC, and could even be an opposite relationship in the malignant progression of gastric cancer among each family member.

To sum up, we found that FAM83C/D/G/H play an important role in the prognosis of patients with GC, and the high expression of FAM83C/D was significantly related to the poor prognosis of patients with STAD, but the high expression of FAM83G/H was remarkably associated with the favorable prognosis of patients with GC. Therefore, we believe that the roles of FAM83 family members in the occurrence and development of GC are not uniform or unique.

However, there were some limitations in our study. First, the data utilized were obtained from different databases, which might not avoid the heterogeneity between different data, so further studies with larger sample sizes are needed to confirm our findings. Secondly, we did not conduct further *in vivo* or *in vitro* experiments to verify the data and the results of bioinformatics analysis. We also realize that further experiments should be performed to improve and verify our results.

## Conclusion

In this study, we formulated the expression, genetic alteration, immune cell infiltration and prognostic values of FAM83 family members in STAD, which also provide a new direction for the individualized diagnosis and treatment of STAD. We have established the best prognostic model of FAM83 family members in GC, including the prognostic model of patients with GC (FAM83C/D/G), patients with STAD with lymph node metastasis (FAM83C/D/G/H) and patients having STAD with high expression of ERBB2 (FAM83/G/H). In addition, we found obvious correlations among the expression levels of FAM83C/D/G/H and the infiltration of six different immune cells (B cells, CD8^+^ T cells, CD4^+^ T cells, macrophages and myeloid DCs) and discovered that CD4^+^ T cells and macrophages might be poor prognostic factors for STAD, while NK and B cells might be favorable prognostic factors.

In general, our results indicate that FAM83C/D/G/H might be significantly correlated with oncogenesis and the progression of STAD. FAM83C/D might be risk factors, but FAM83G/H might be favorable prognostic factors for the survival of patients with STAD. In addition, CD4^+^ cells and macrophages may be the promoters of FAM83D in promoting the malignant progression of STAD, while NK cells may promote the protective effect of FAM83H in patients with STAD.

## Data Availability Statement

The datasets presented in this study can be found in online repositories. The names of the repository/repositories and accession number(s) can be found in the article/Supplementary Material.

## Ethics Statement

The studies involving human participants were reviewed and approved by The First Affiliated Hospital of Sun Yat-sen University. Written informed consent for participation was not required for this study in accordance with the national legislation and the institutional requirements. Written informed consent was not obtained from the individual(s) for the publication of any potentially identifiable images or data included in this article.

## Author Contributions

TZ, SL, and YC contributed to including study concept and design, analysis and interpretation of data, drafting of the manuscript, and critical revision of the manuscript for important intellectual content conceived of and designed the study. ZH, YL, SLC, ZZ, ZY, XL, and EZ participated in the literature search, acquisition of data, tissue specimen collection and statistical analysis. SRC and JC supervised the whole study and edited and reviewed the manuscript. All authors read and approved the final manuscript.

## Conflict of Interest

The authors declare that the research was conducted in the absence of any commercial or financial relationships that could be construed as a potential conflict of interest.

## Publisher’s Note

All claims expressed in this article are solely those of the authors and do not necessarily represent those of their affiliated organizations, or those of the publisher, the editors and the reviewers. Any product that may be evaluated in this article, or claim that may be made by its manufacturer, is not guaranteed or endorsed by the publisher.

## Footnotes

1
http://www.oncomine.org

2
http://ualcan.path.uab.edu/analysis.html

3
http://gepia.cancer-pku.cn/index.html

4
http://kmplot.com/analysis/

5
http://www.cbioportal.org

6
https://string-db.org/

7
https://david.ncifcrf.gov/home.jsp

8
https://cistrome.shinyapps.io/timer/

9
https://www.proteinatlas.org/

## Abbreviations

AUC: Areas Under the Curve
BP: Biological Process
CC: Cellular Component
DC: Dendritic Cell
FP: Free Progression survival
DFS: Disease-Free Survival
GC: Gastric Cancer
GEO: Gene Expression Omnibus
GO: Gene Ontology
HR: Hazard Ratios
KEGG: Kyoto Encyclopedia of Genes and Genomes
MF: Molecular Function
OS: Overall Survival
PPI: Protein-protein Interaction Network
PPS: Post Progression Survival
STAD: Stomach Adenocarcinoma
TCGA: The Cancer Genome Atlas
TIMER: Tumor Immune Estimation Resource.
