# Supplementary figures and images for: Comprehensive Analysis and Identification of Prognostic Biomarkers and Therapeutic Targets Among FAM83 Family Members for Gastric Cancer (part 1 of 2)
Source: Front Cell Dev Biol. 2021 Nov 19;9:719613. doi: 10.3389/fcell.2021.719613 (PMC8640971; doi:10.3389/fcell.2021.719613)

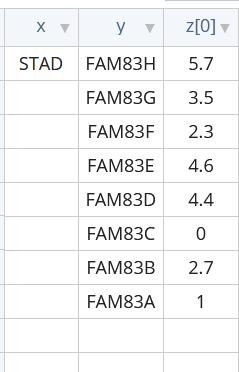

Supplement: Supplementary file 1 [file Data_Sheet_1.ZIP › Supplementary materials fig.1/Data of fig1.B.jpg]

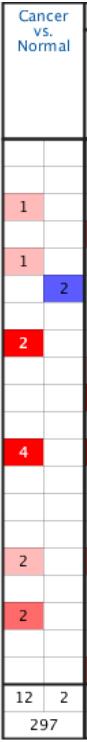

Supplement: Supplementary file 1 [file Data_Sheet_1.ZIP › Supplementary materials fig.1/FAM83A.jpg]

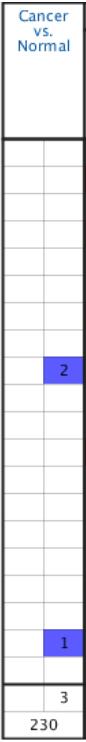

Supplement: Supplementary file 1 [file Data_Sheet_1.ZIP › Supplementary materials fig.1/FAM83B.jpg]

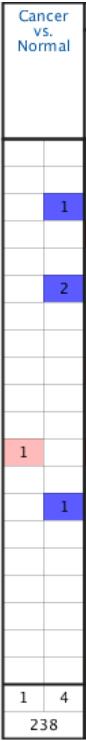

Supplement: Supplementary file 1 [file Data_Sheet_1.ZIP › Supplementary materials fig.1/FAM83C.jpg]

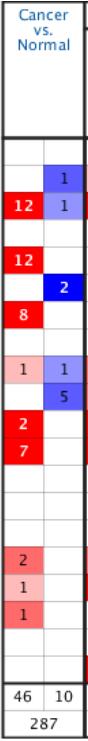

Supplement: Supplementary file 1 [file Data_Sheet_1.ZIP › Supplementary materials fig.1/FAM83D.jpg]

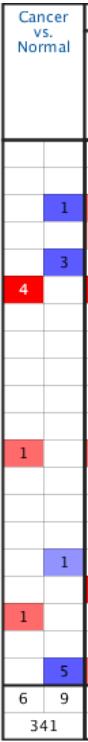

Supplement: Supplementary file 1 [file Data_Sheet_1.ZIP › Supplementary materials fig.1/FAM83E.jpg]

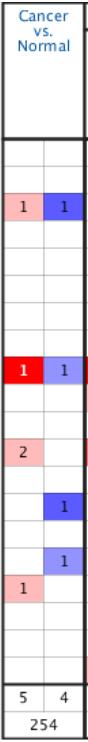

Supplement: Supplementary file 1 [file Data_Sheet_1.ZIP › Supplementary materials fig.1/FAM83F.jpg]

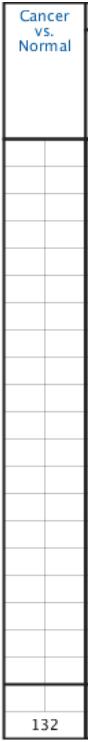

Supplement: Supplementary file 1 [file Data_Sheet_1.ZIP › Supplementary materials fig.1/FAM83G.jpg]

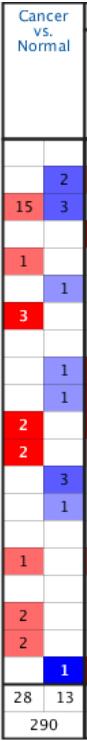

Supplement: Supplementary file 1 [file Data_Sheet_1.ZIP › Supplementary materials fig.1/FAM83H.jpg]

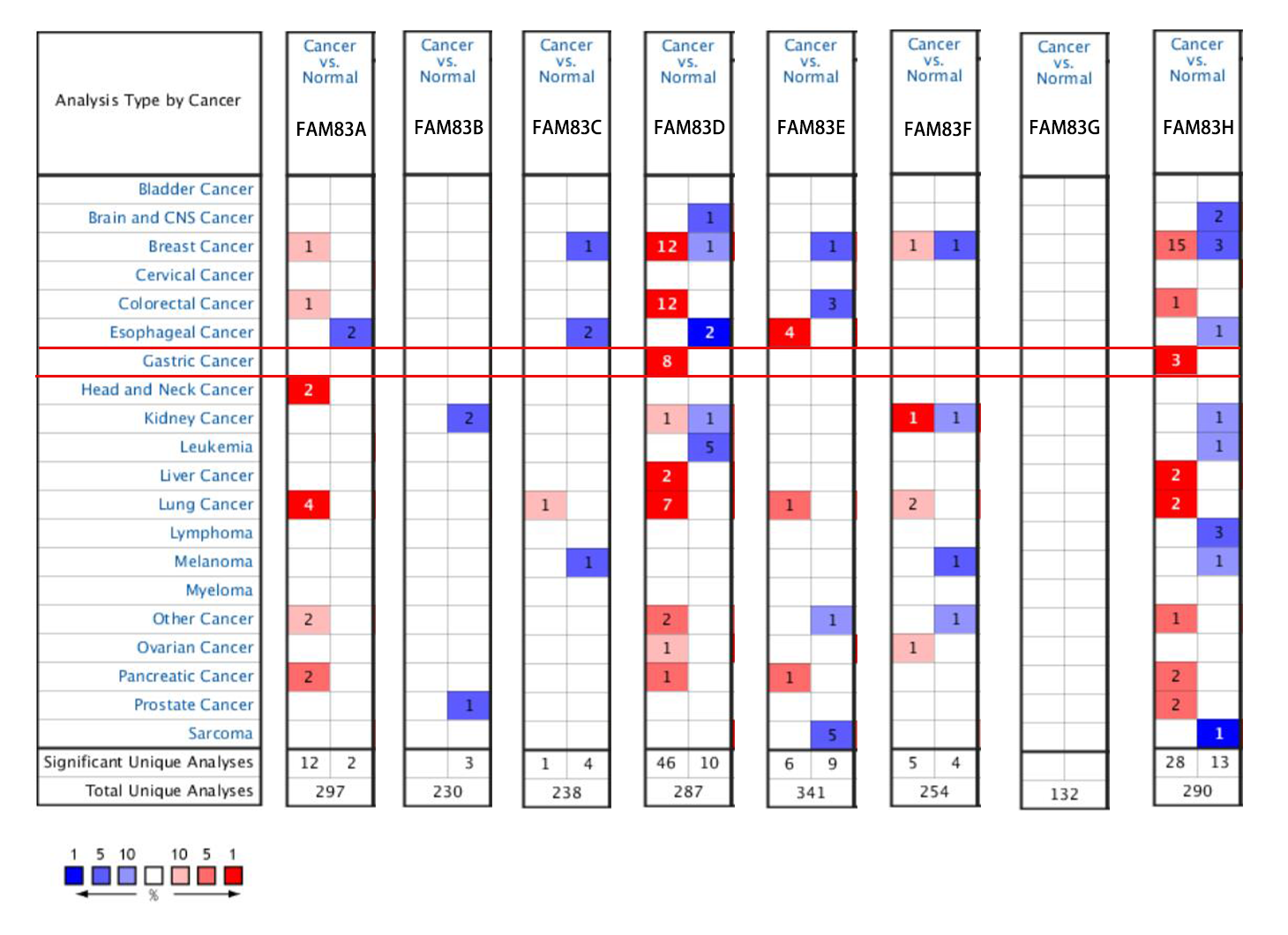

Supplement: Supplementary file 1 [file Data_Sheet_1.ZIP › Supplementary materials fig.1/fig1.A.tif]

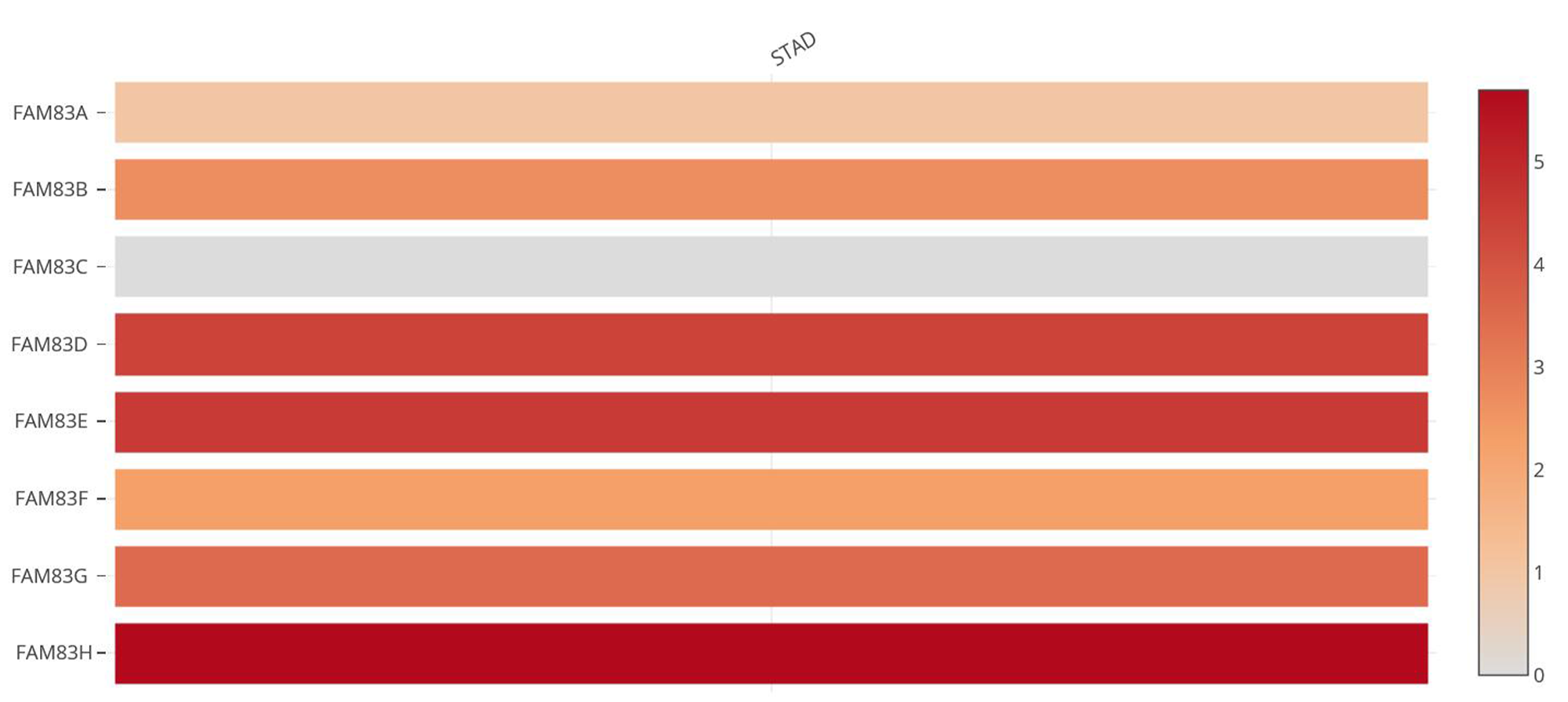

Supplement: Supplementary file 1 [file Data_Sheet_1.ZIP › Supplementary materials fig.1/fig1.B.tif]

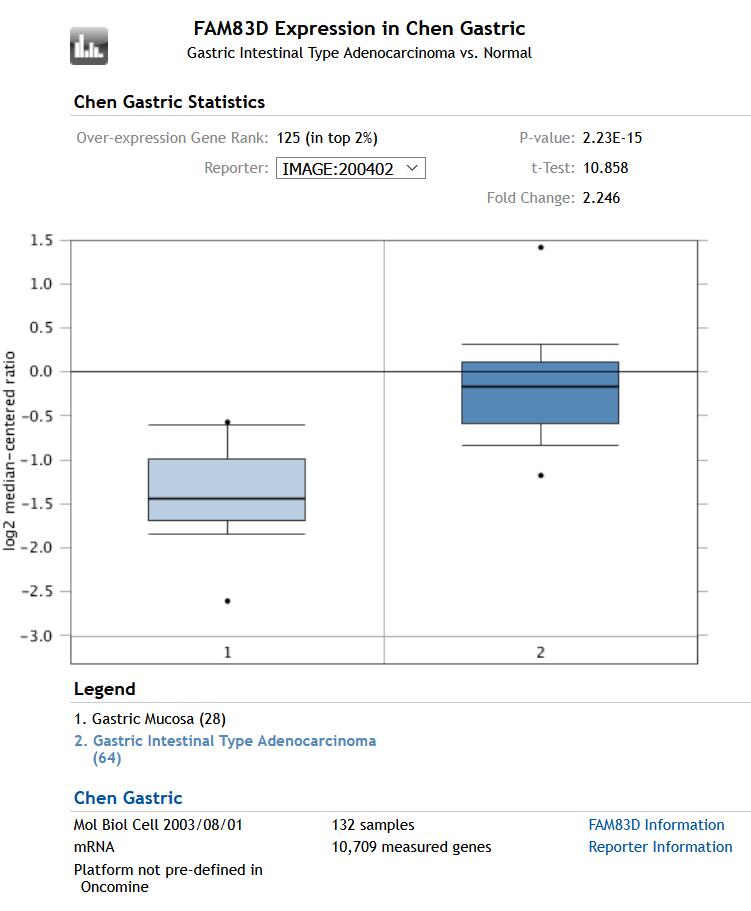

Supplement: Supplementary file 1 [file Data_Sheet_1.ZIP › Supplementary materials fig.1/oncomine/FAM83D/1.jpg]

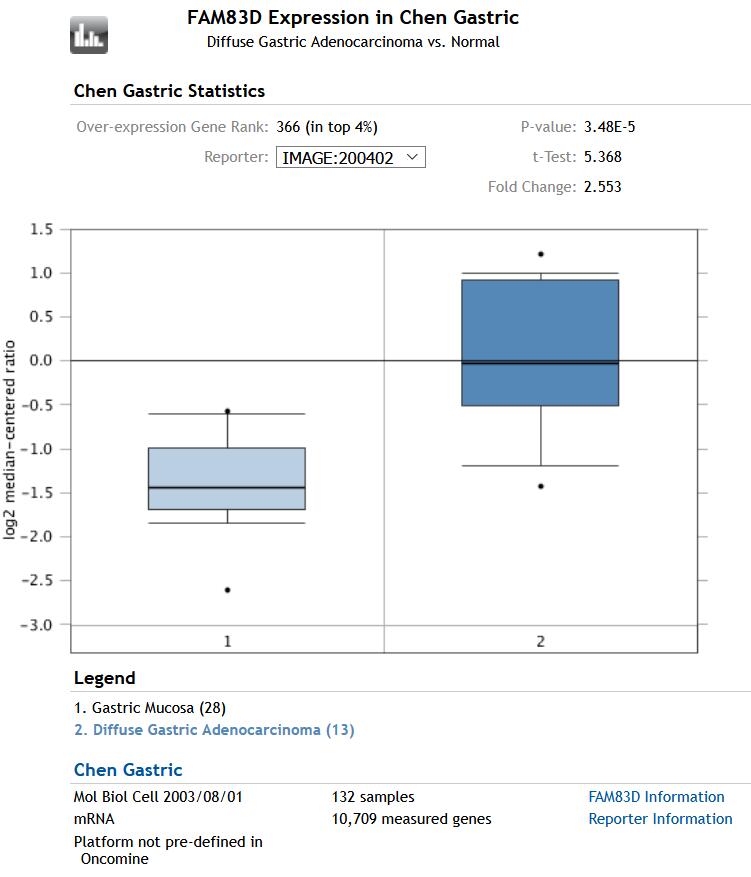

Supplement: Supplementary file 1 [file Data_Sheet_1.ZIP › Supplementary materials fig.1/oncomine/FAM83D/2.jpg]

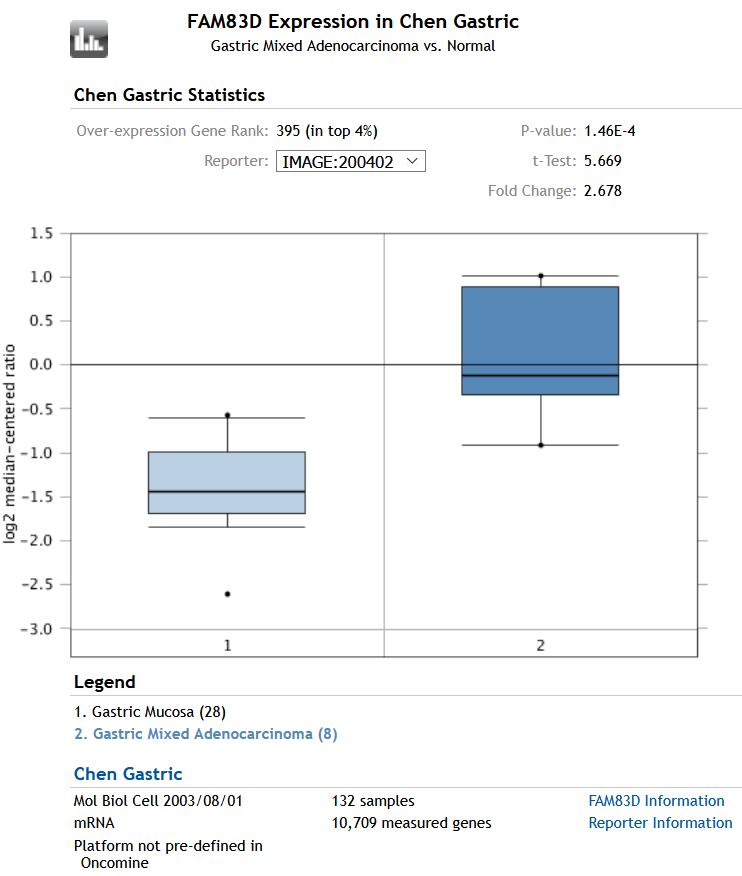

Supplement: Supplementary file 1 [file Data_Sheet_1.ZIP › Supplementary materials fig.1/oncomine/FAM83D/3.jpg]

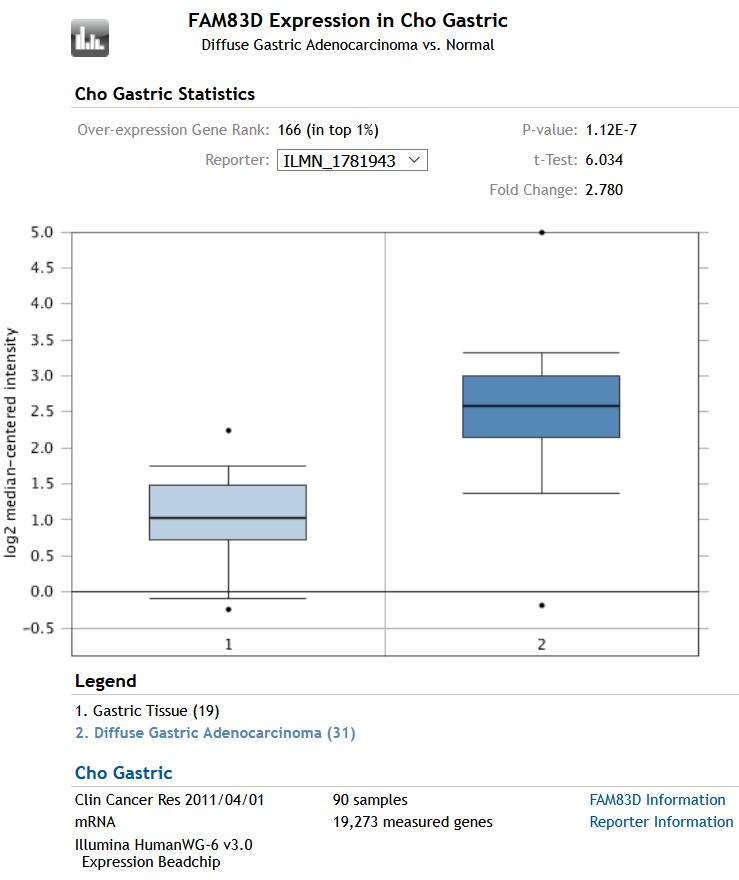

Supplement: Supplementary file 1 [file Data_Sheet_1.ZIP › Supplementary materials fig.1/oncomine/FAM83D/4.jpg]

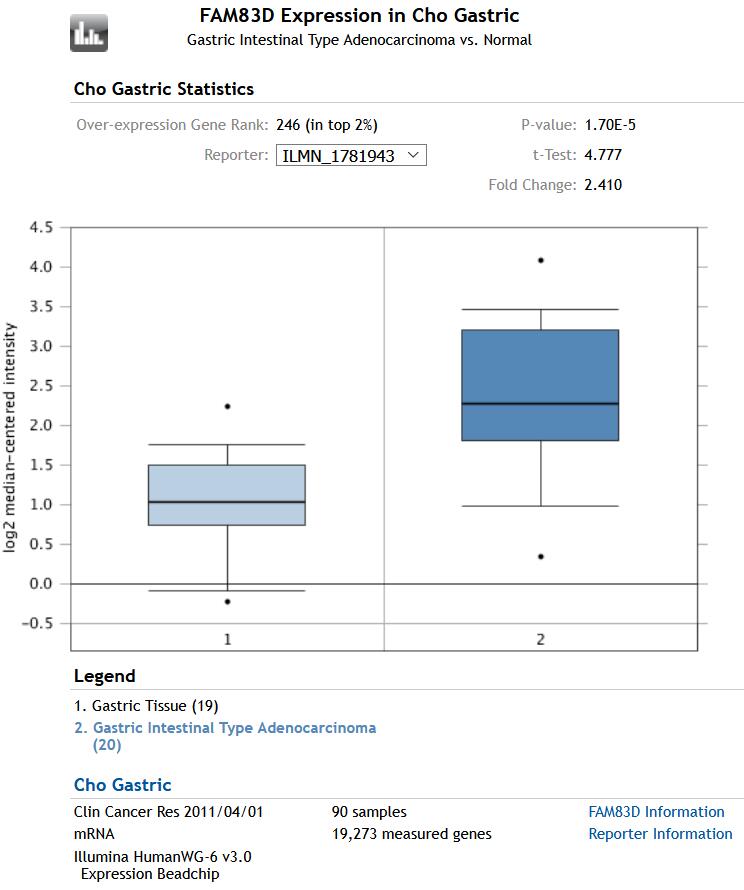

Supplement: Supplementary file 1 [file Data_Sheet_1.ZIP › Supplementary materials fig.1/oncomine/FAM83D/5.jpg]

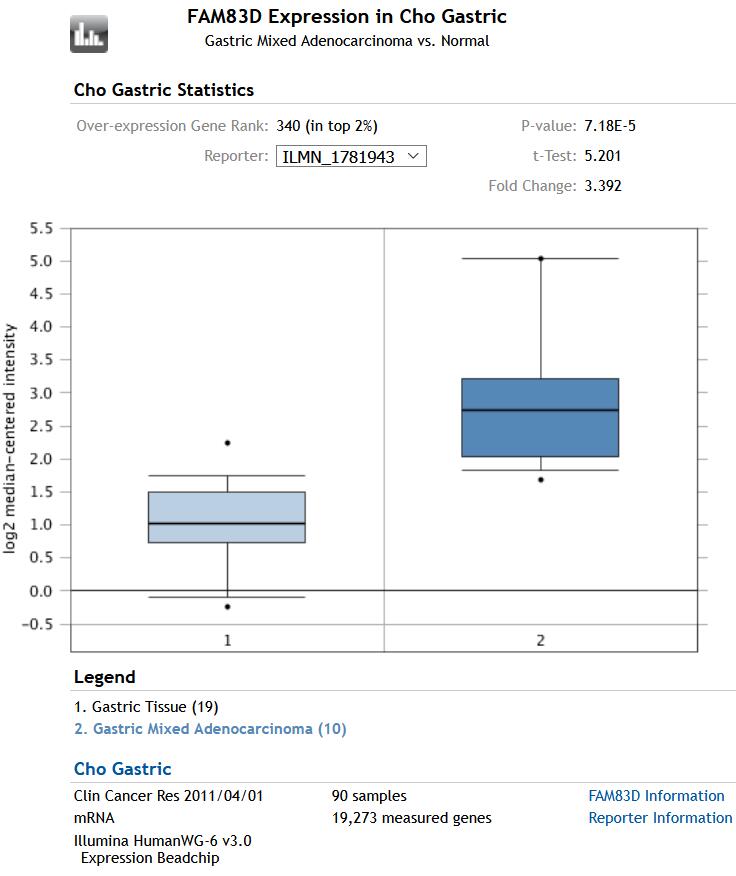

Supplement: Supplementary file 1 [file Data_Sheet_1.ZIP › Supplementary materials fig.1/oncomine/FAM83D/6.jpg]

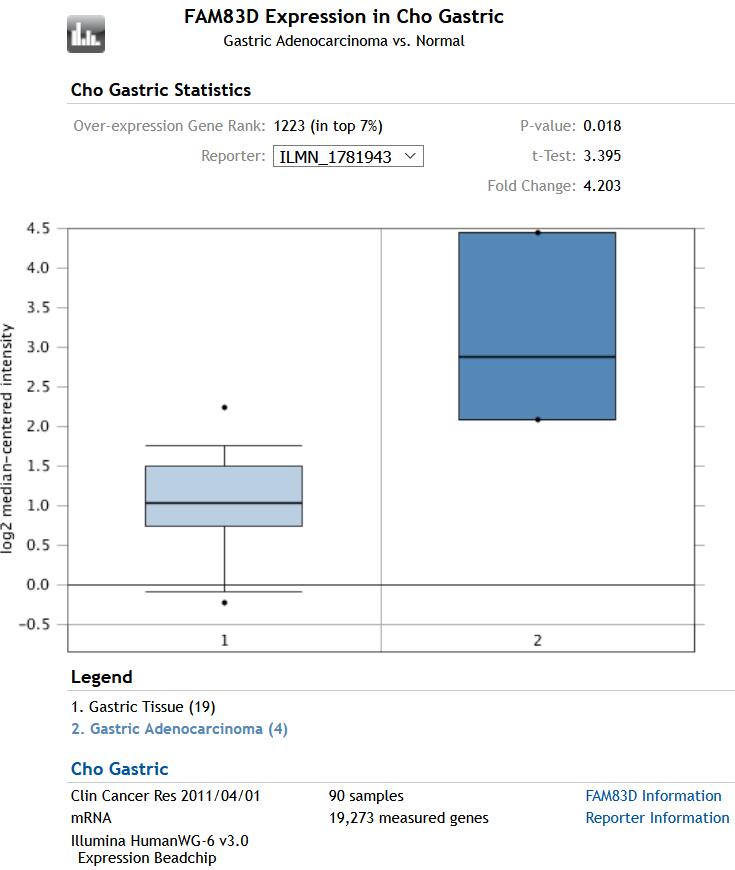

Supplement: Supplementary file 1 [file Data_Sheet_1.ZIP › Supplementary materials fig.1/oncomine/FAM83D/7.jpg]

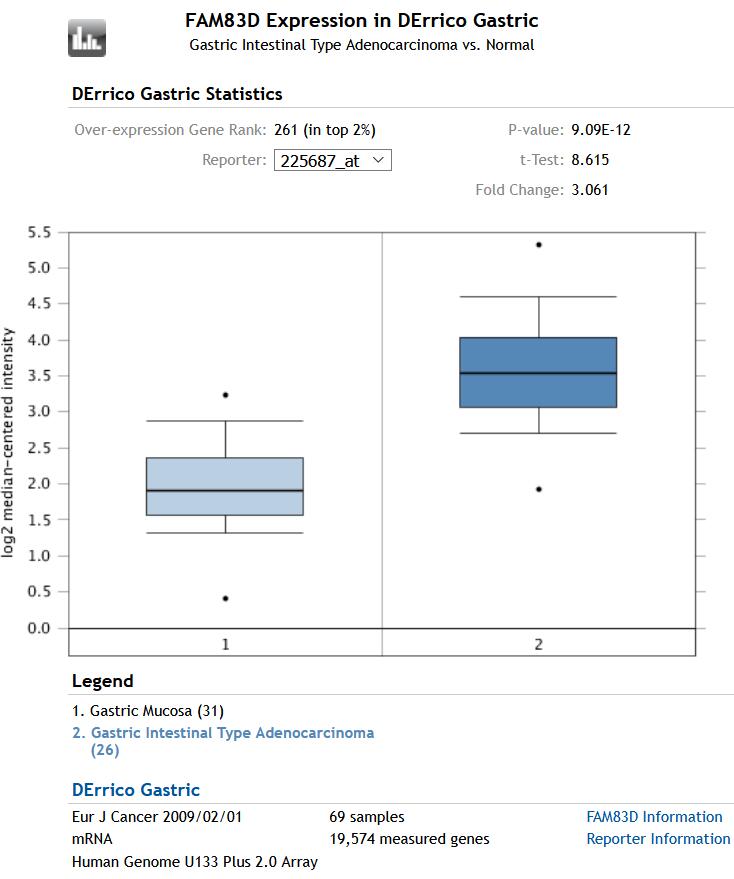

Supplement: Supplementary file 1 [file Data_Sheet_1.ZIP › Supplementary materials fig.1/oncomine/FAM83D/8.jpg]

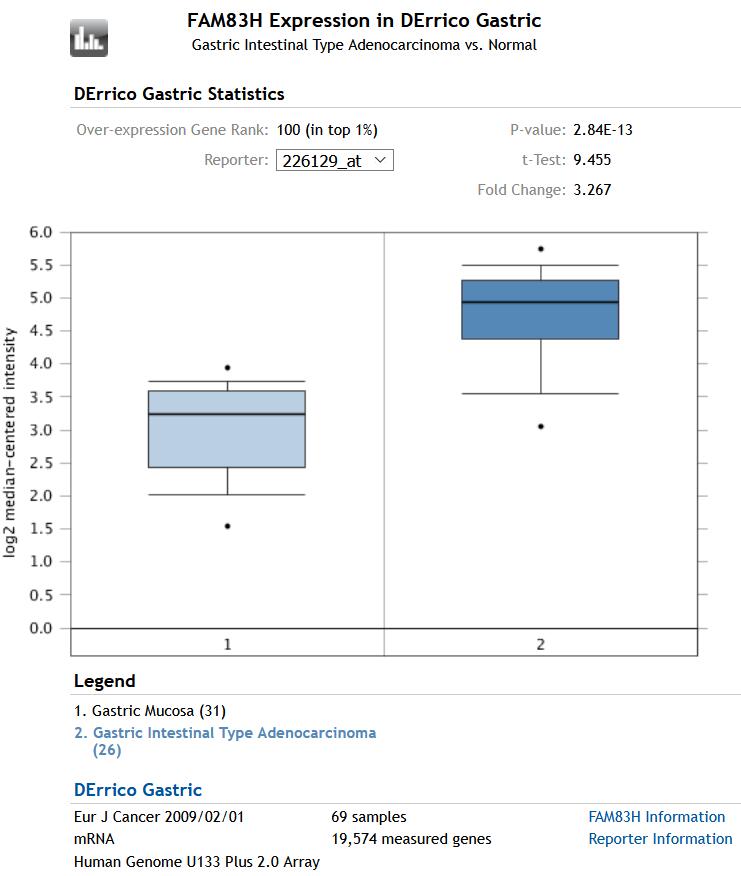

Supplement: Supplementary file 1 [file Data_Sheet_1.ZIP › Supplementary materials fig.1/oncomine/FAM83H/1.jpg]

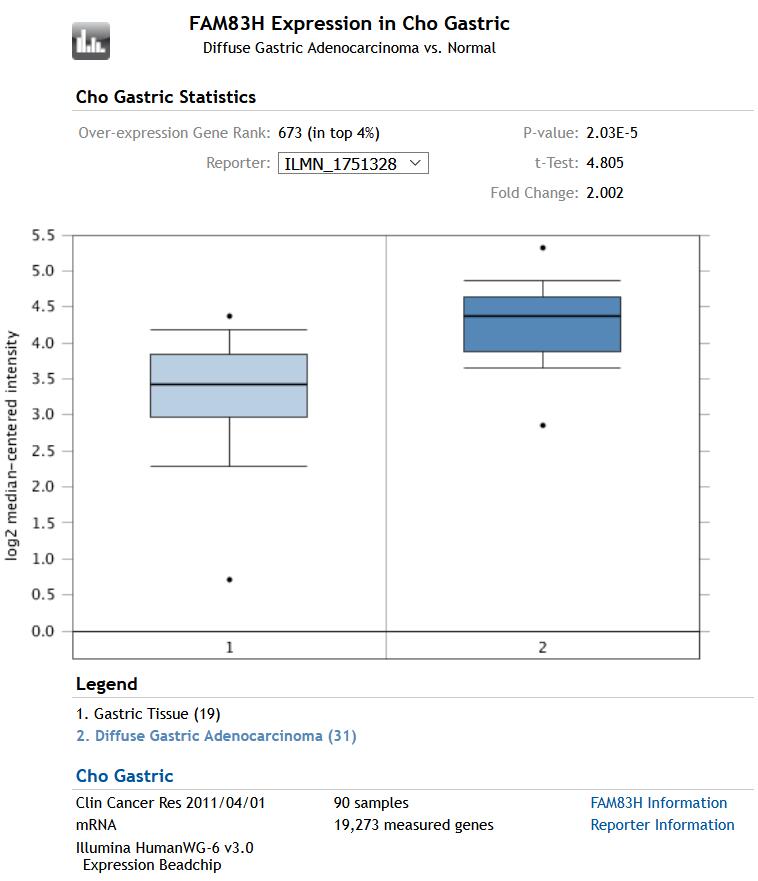

Supplement: Supplementary file 1 [file Data_Sheet_1.ZIP › Supplementary materials fig.1/oncomine/FAM83H/2.jpg]

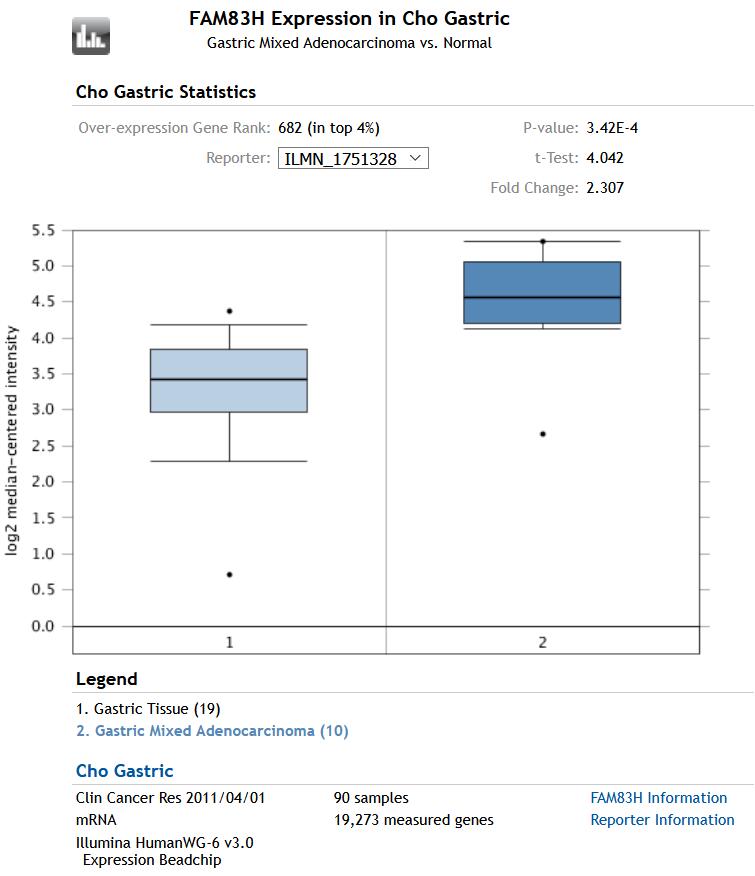

Supplement: Supplementary file 1 [file Data_Sheet_1.ZIP › Supplementary materials fig.1/oncomine/FAM83H/3.jpg]

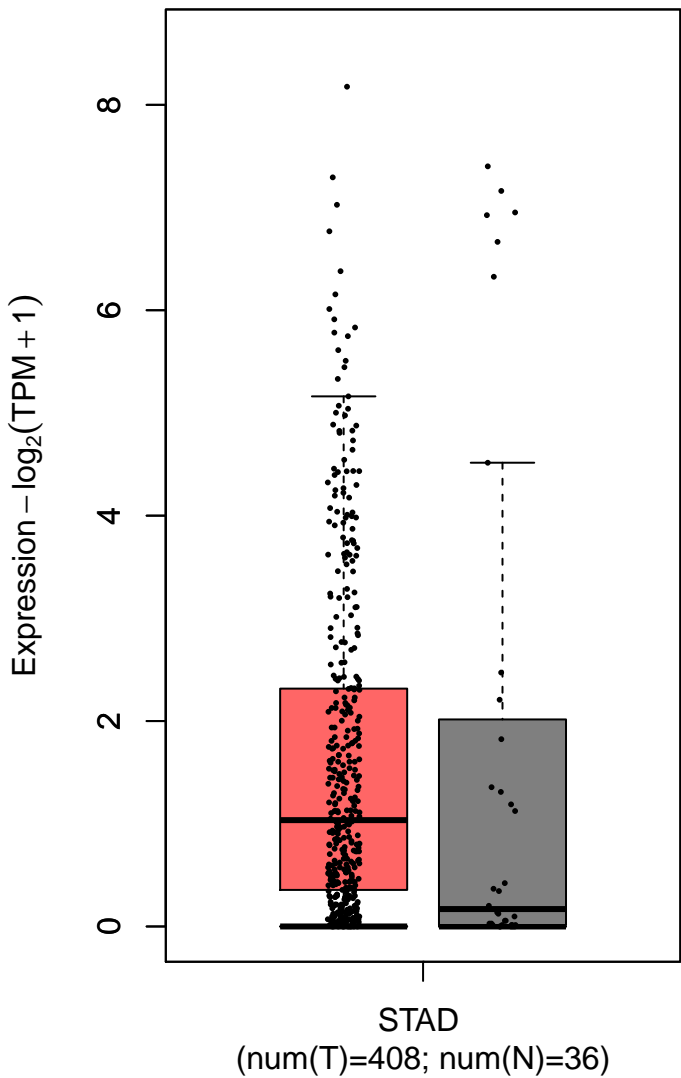

Supplement: Supplementary file 2 [file Data_Sheet_2.ZIP › Supplementary materials fig.2/GEPIA2.0/FAM83A_boxplot_S5Z30.pdf]

Expression  $-\log_2(\text{TPM} + 1)$

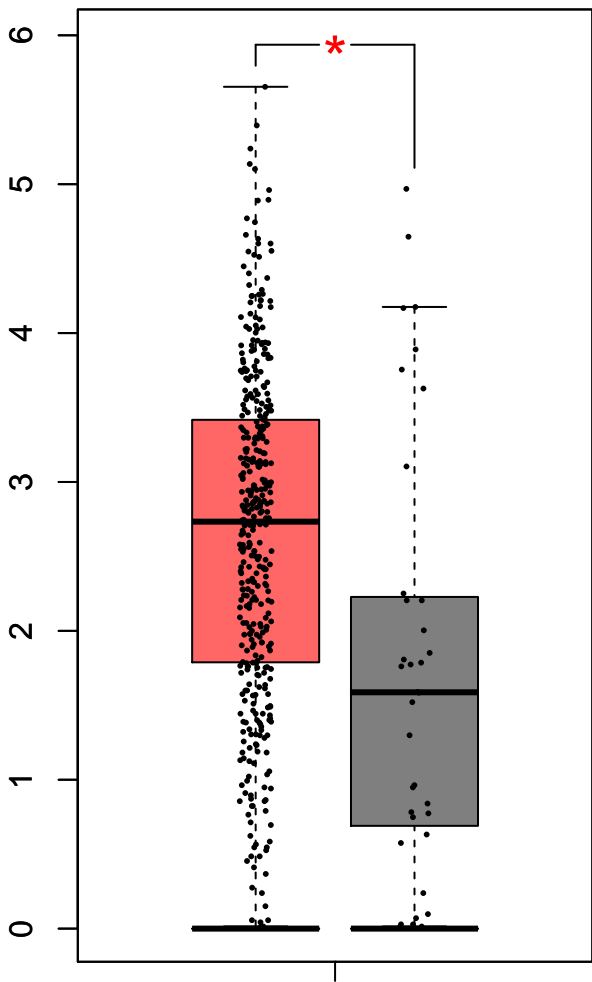

STAD

(num(T)=408; num(N)=36)

Supplement: Supplementary file 2 [file Data_Sheet_2.ZIP › Supplementary materials fig.2/GEPIA2.0/FAM83B_boxplot_D2mu5.pdf]

Expression  $-\log_2(\text{TPM} + 1)$

5  
4  
3  
2  
1  
0

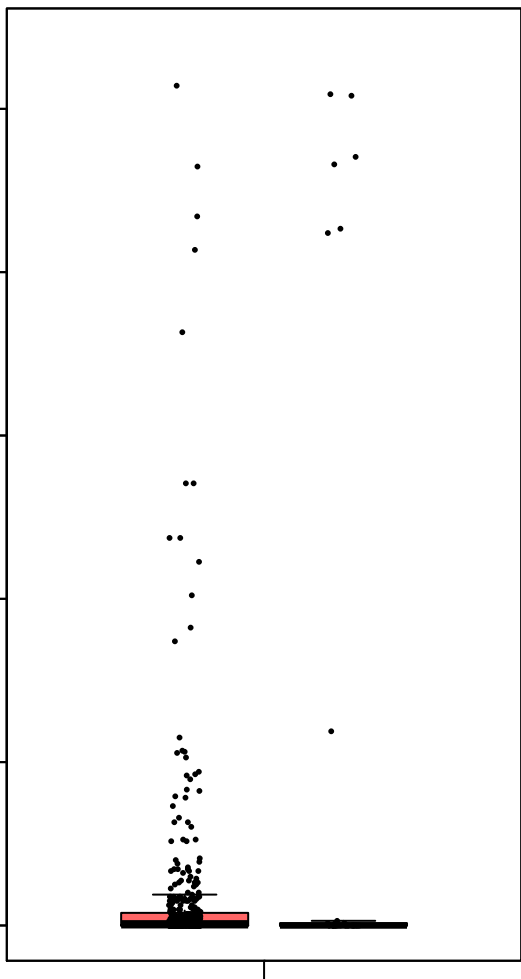

STAD

(num(T)=408; num(N)=36)

Supplement: Supplementary file 2 [file Data_Sheet_2.ZIP › Supplementary materials fig.2/GEPIA2.0/FAM83C_boxplot_vkgVc.pdf]

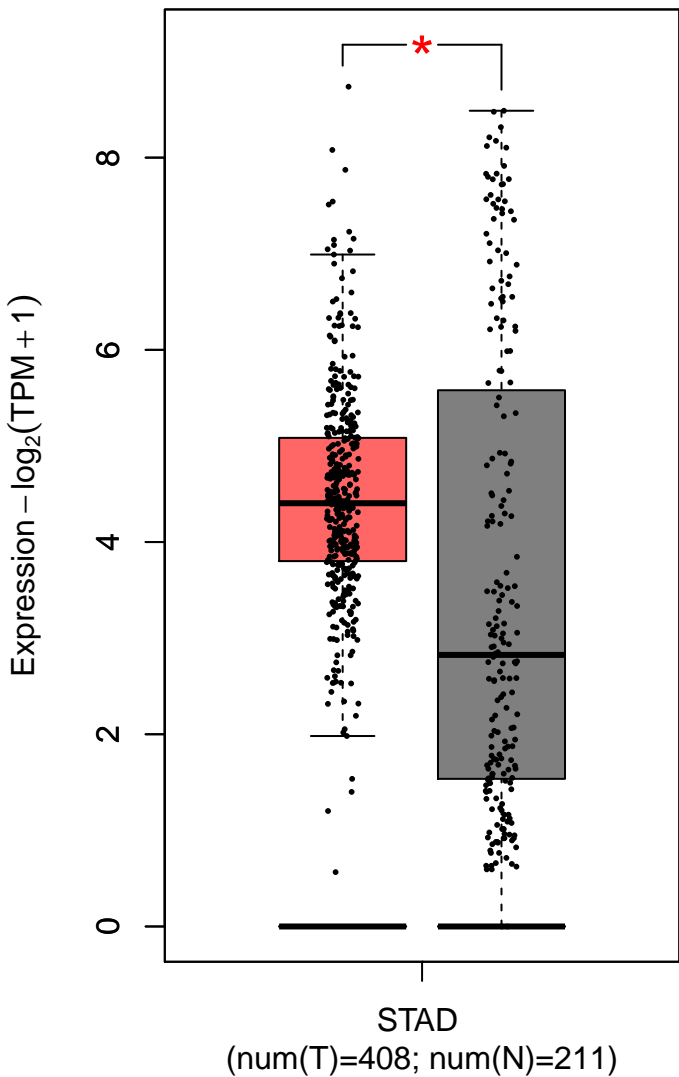

Supplement: Supplementary file 2 [file Data_Sheet_2.ZIP › Supplementary materials fig.2/GEPIA2.0/FAM83D_boxplot_ODMth.pdf]

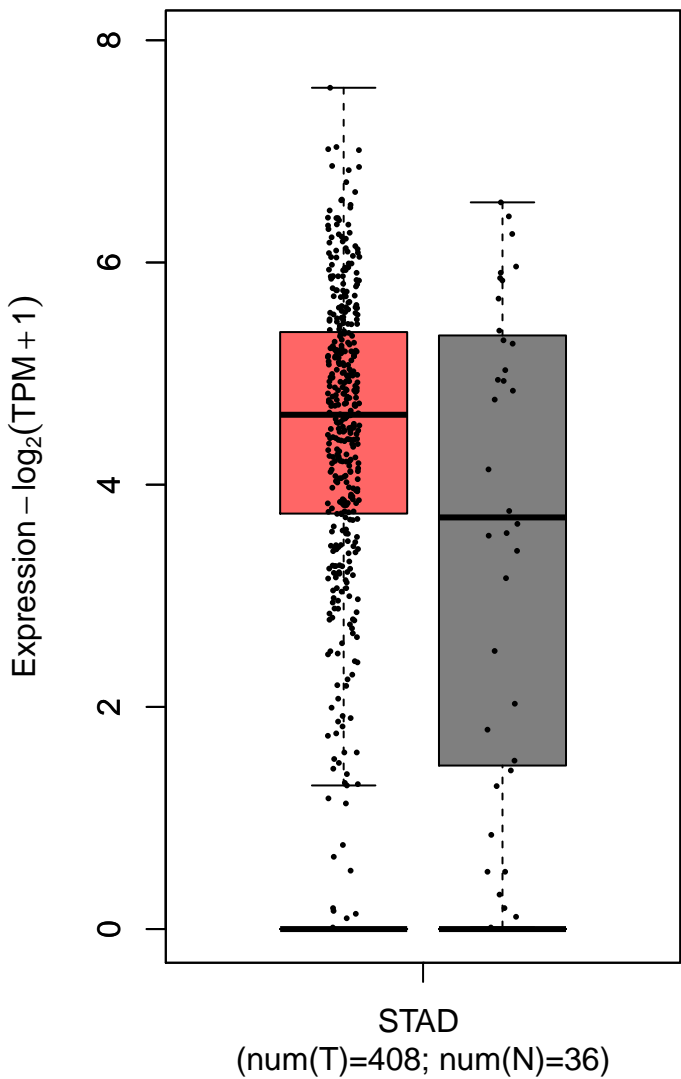

Supplement: Supplementary file 2 [file Data_Sheet_2.ZIP › Supplementary materials fig.2/GEPIA2.0/FAM83E_boxplot_QQQVw.pdf]

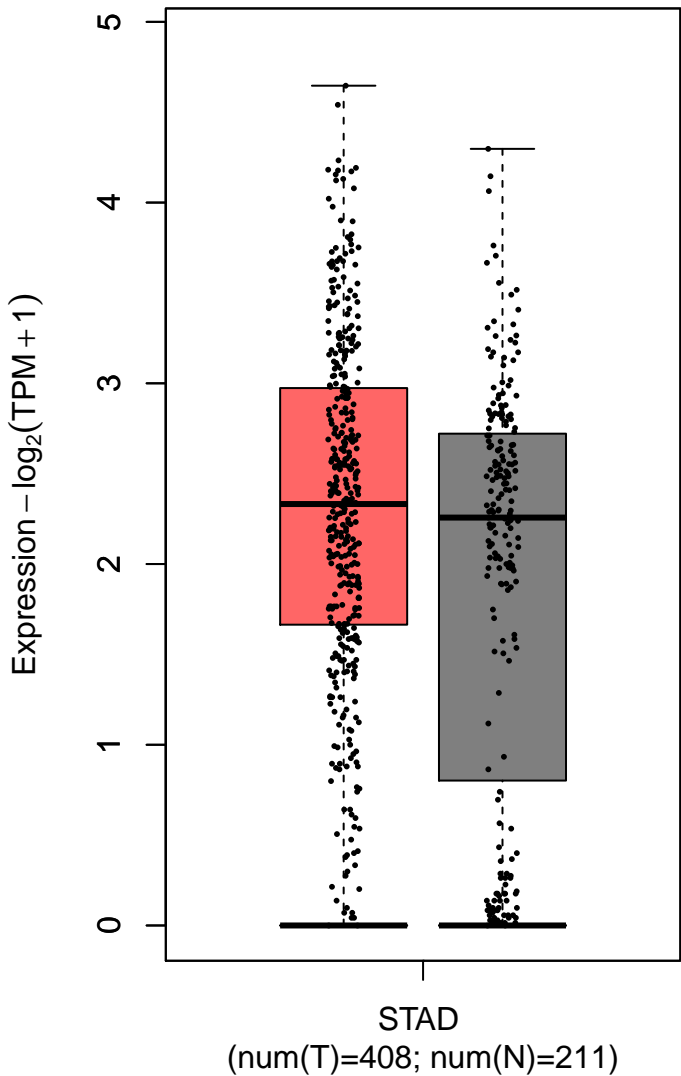

Supplement: Supplementary file 2 [file Data_Sheet_2.ZIP › Supplementary materials fig.2/GEPIA2.0/FAM83F_boxplot_SCzaH.pdf]

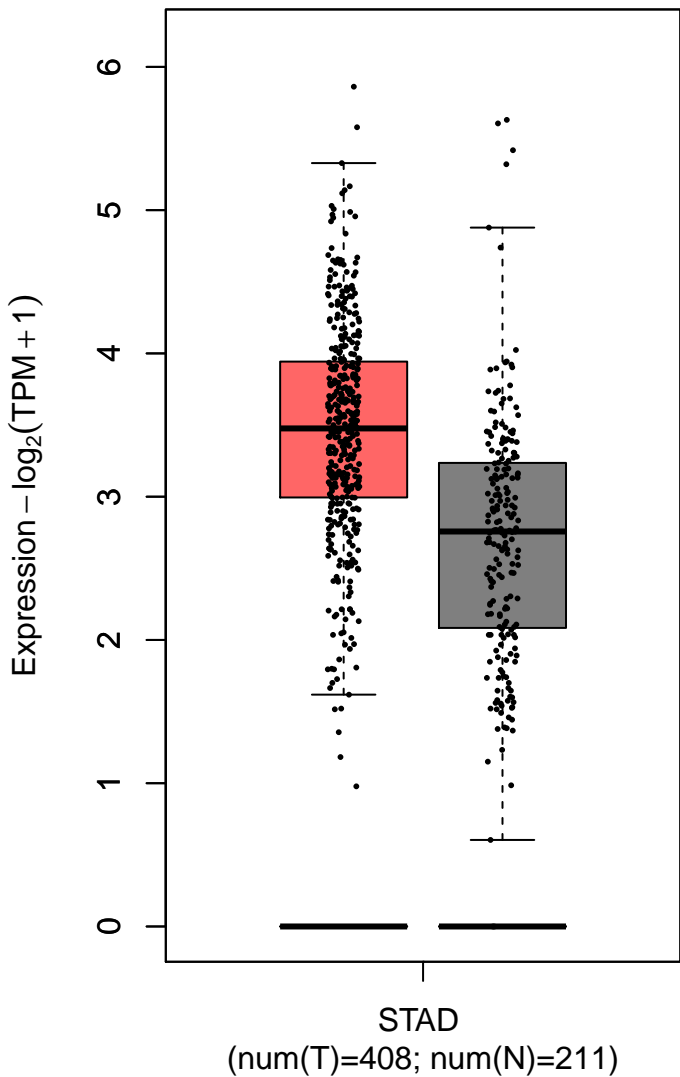

Supplement: Supplementary file 2 [file Data_Sheet_2.ZIP › Supplementary materials fig.2/GEPIA2.0/FAM83G_boxplot_1zY3D.pdf]

Expression  $-\log_2(\text{TPM} + 1)$

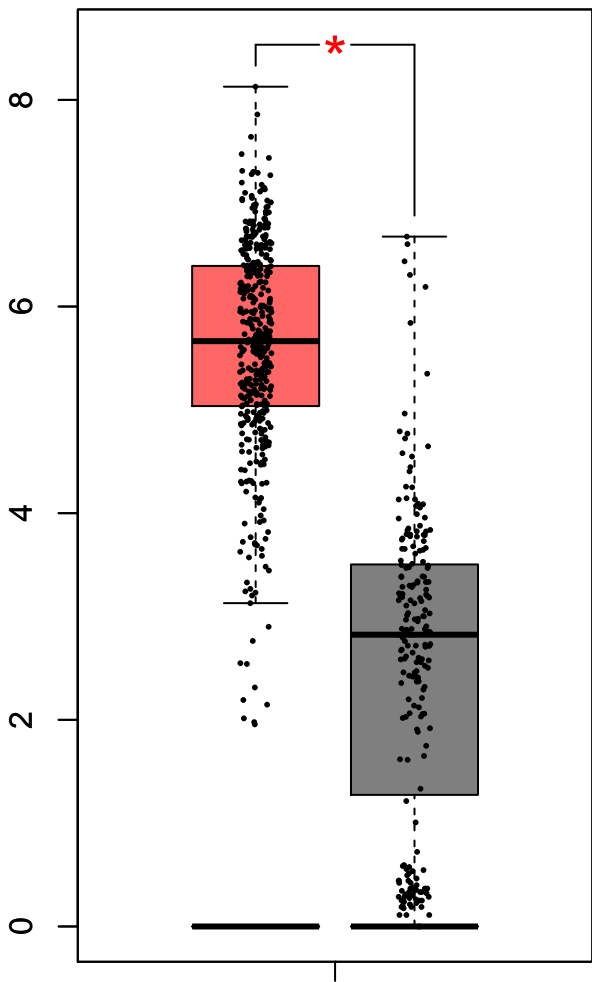

STAD

(num(T)=408; num(N)=211)

Supplement: Supplementary file 2 [file Data_Sheet_2.ZIP › Supplementary materials fig.2/GEPIA2.0/FAM83H_boxplot_XbnsF.pdf]

# Expression of FAM83A in STAD based on Sample types

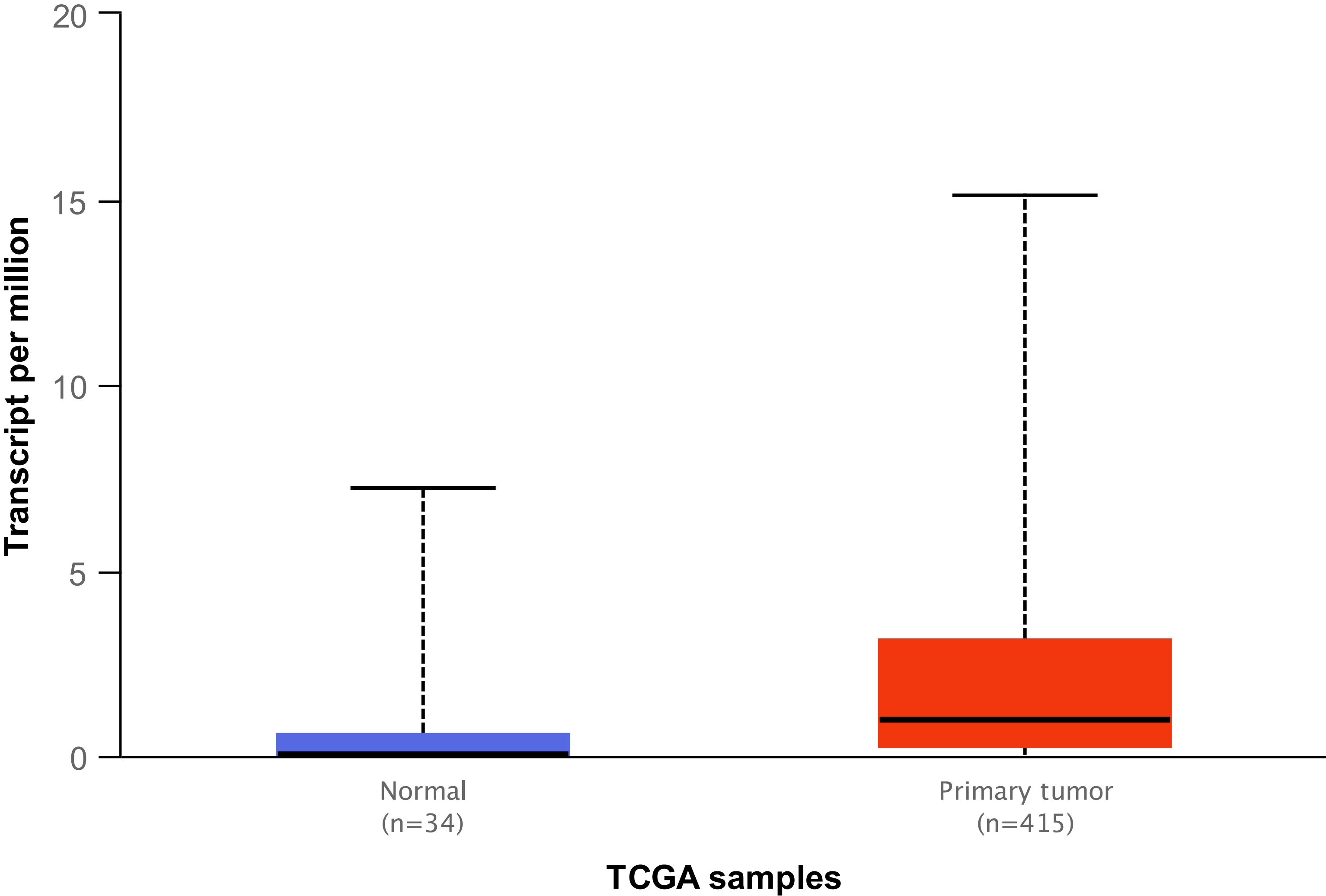

Supplement: Supplementary file 2 [file Data_Sheet_2.ZIP › Supplementary materials fig.2/UALCAN/expression-of-fam83a-in.pdf]

# Expression of FAM83B in STAD based on Sample types

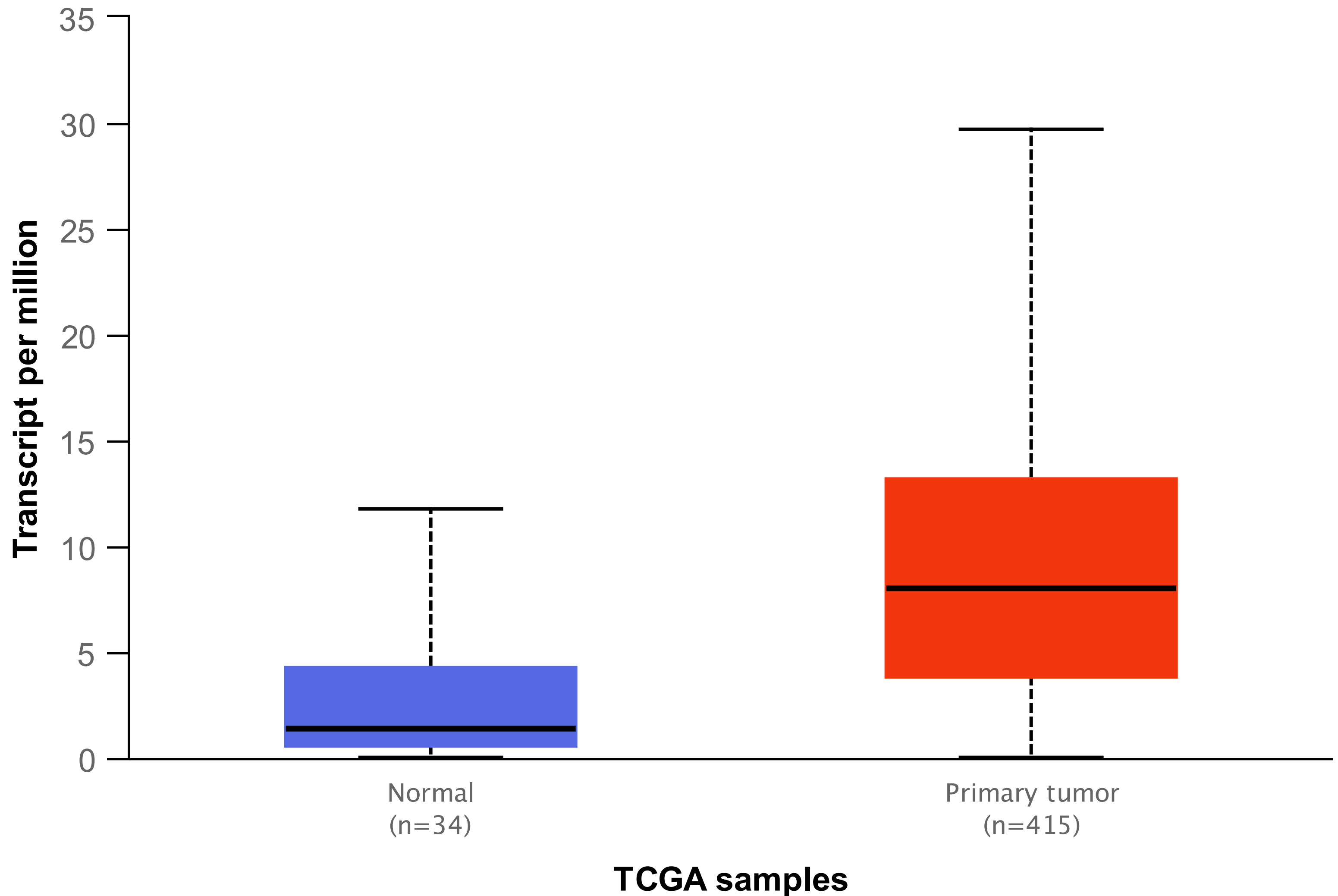

Supplement: Supplementary file 2 [file Data_Sheet_2.ZIP › Supplementary materials fig.2/UALCAN/expression-of-fam83b-in.pdf]

# Expression of FAM83C in STAD based on Sample types

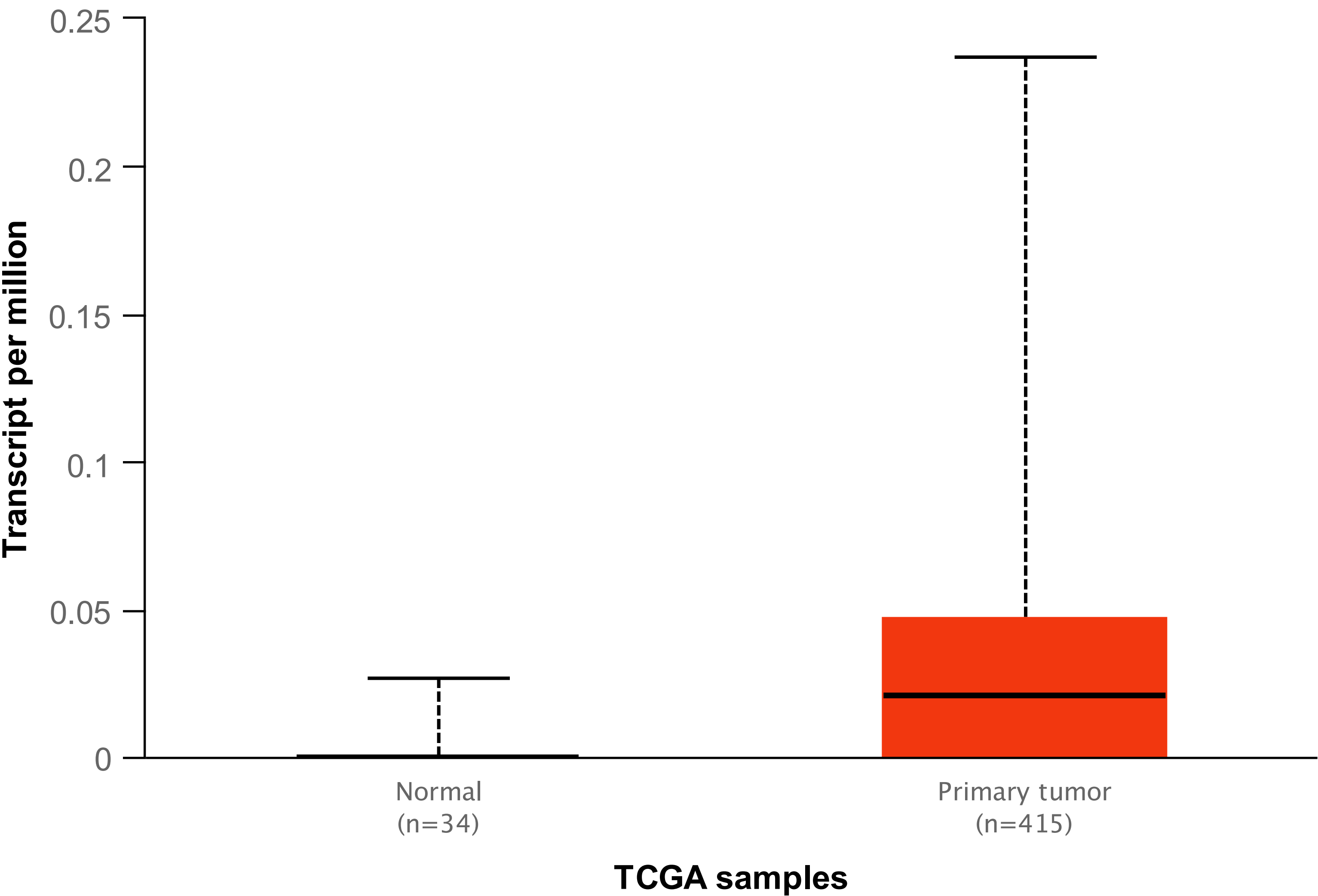

Supplement: Supplementary file 2 [file Data_Sheet_2.ZIP › Supplementary materials fig.2/UALCAN/expression-of-fam83c-in.pdf]

# Expression of FAM83D in STAD based on Sample types

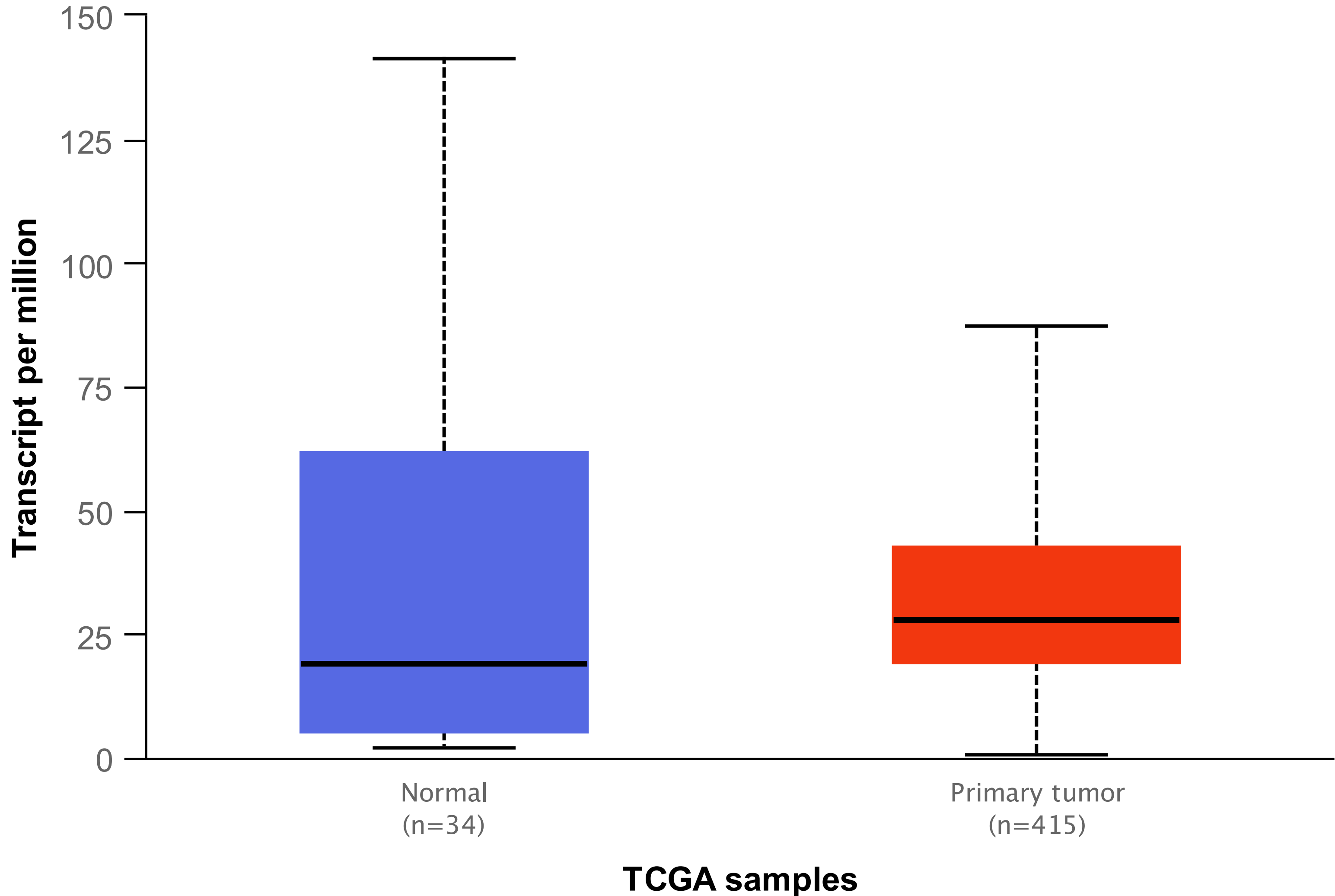

Supplement: Supplementary file 2 [file Data_Sheet_2.ZIP › Supplementary materials fig.2/UALCAN/expression-of-fam83d-in.pdf]

# Expression of FAM83E in STAD based on Sample types

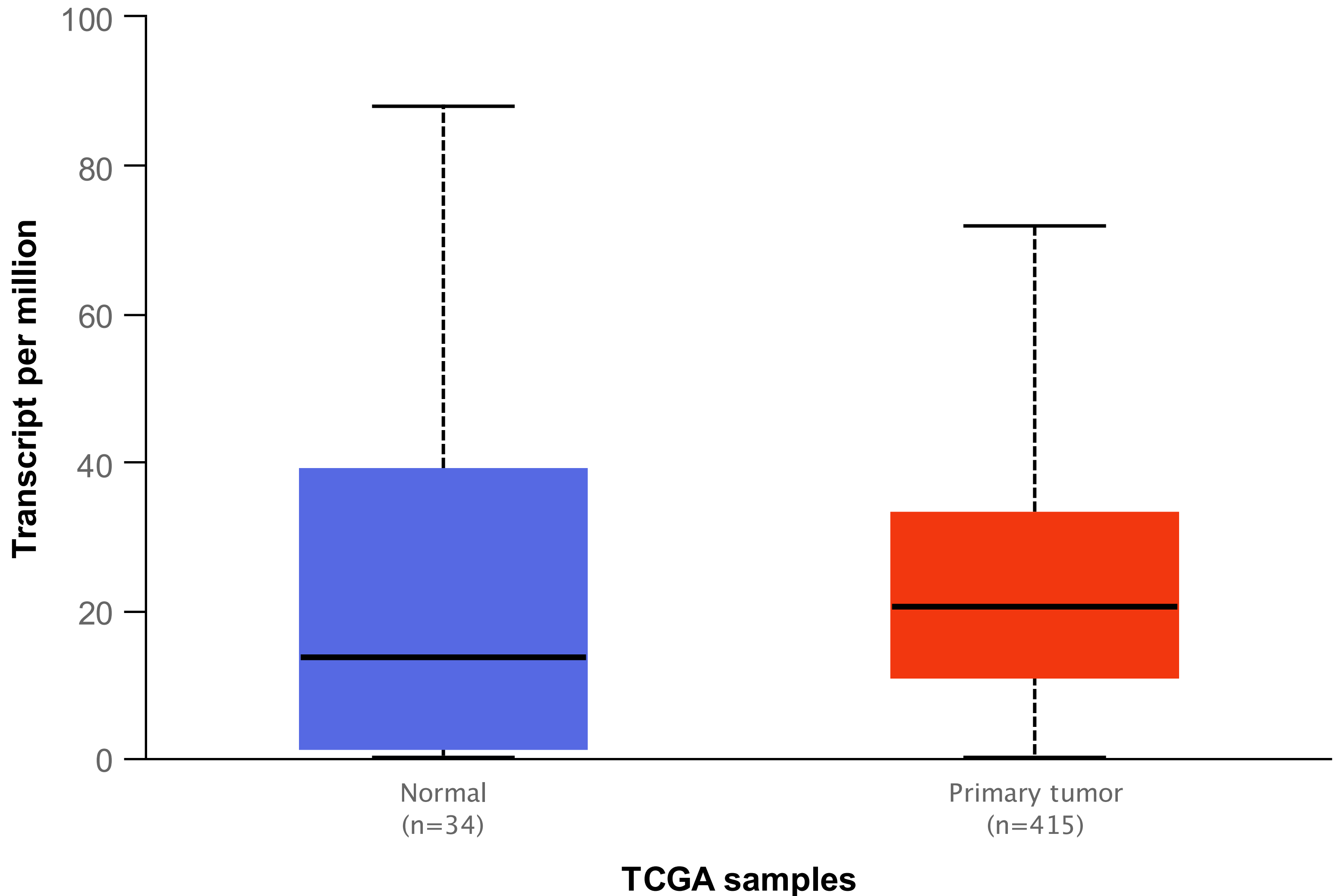

Supplement: Supplementary file 2 [file Data_Sheet_2.ZIP › Supplementary materials fig.2/UALCAN/expression-of-fam83e-in.pdf]

# Expression of FAM83F in STAD based on Sample types

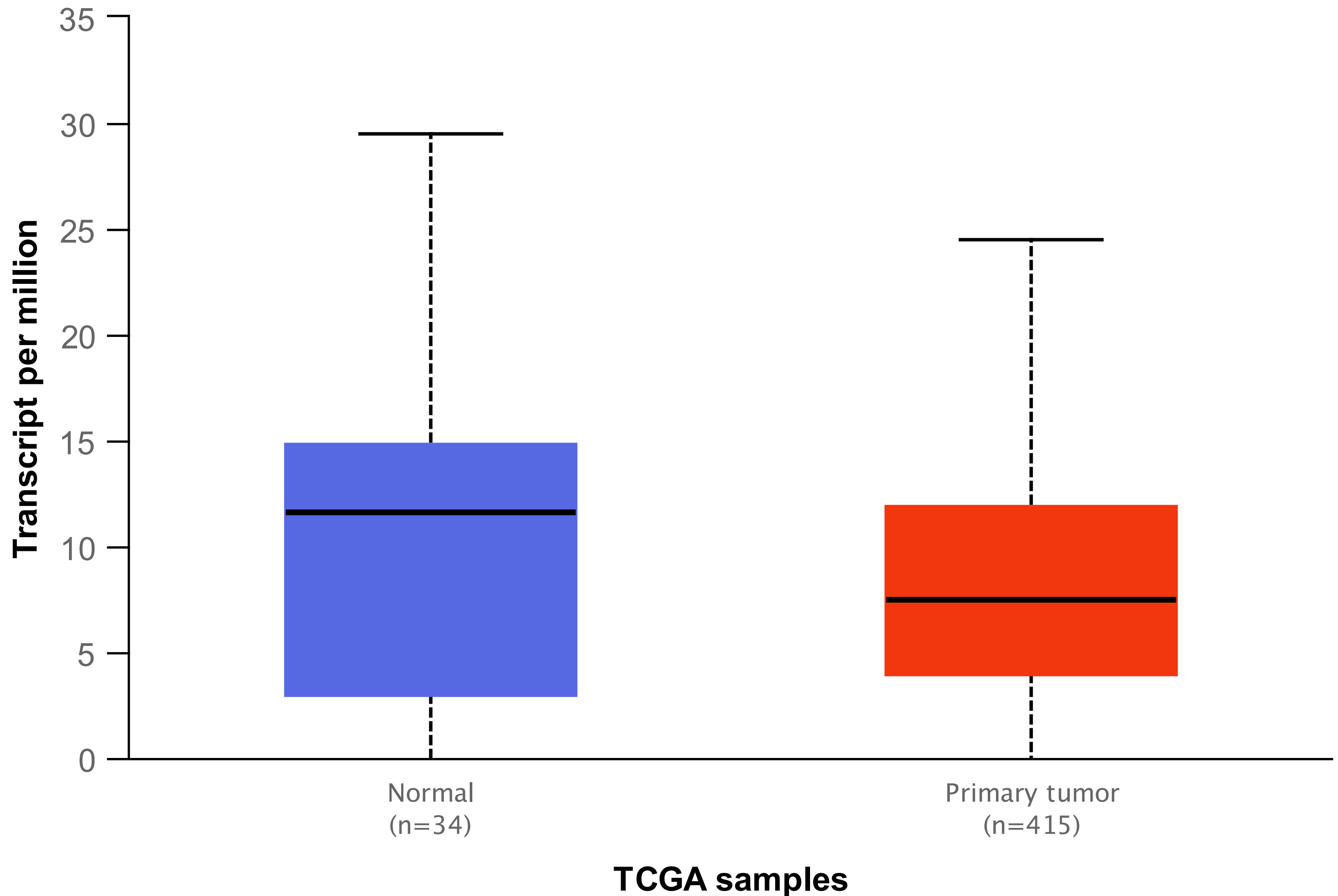

Supplement: Supplementary file 2 [file Data_Sheet_2.ZIP › Supplementary materials fig.2/UALCAN/expression-of-fam83f-in.pdf]

# Expression of FAM83G in STAD based on Sample types

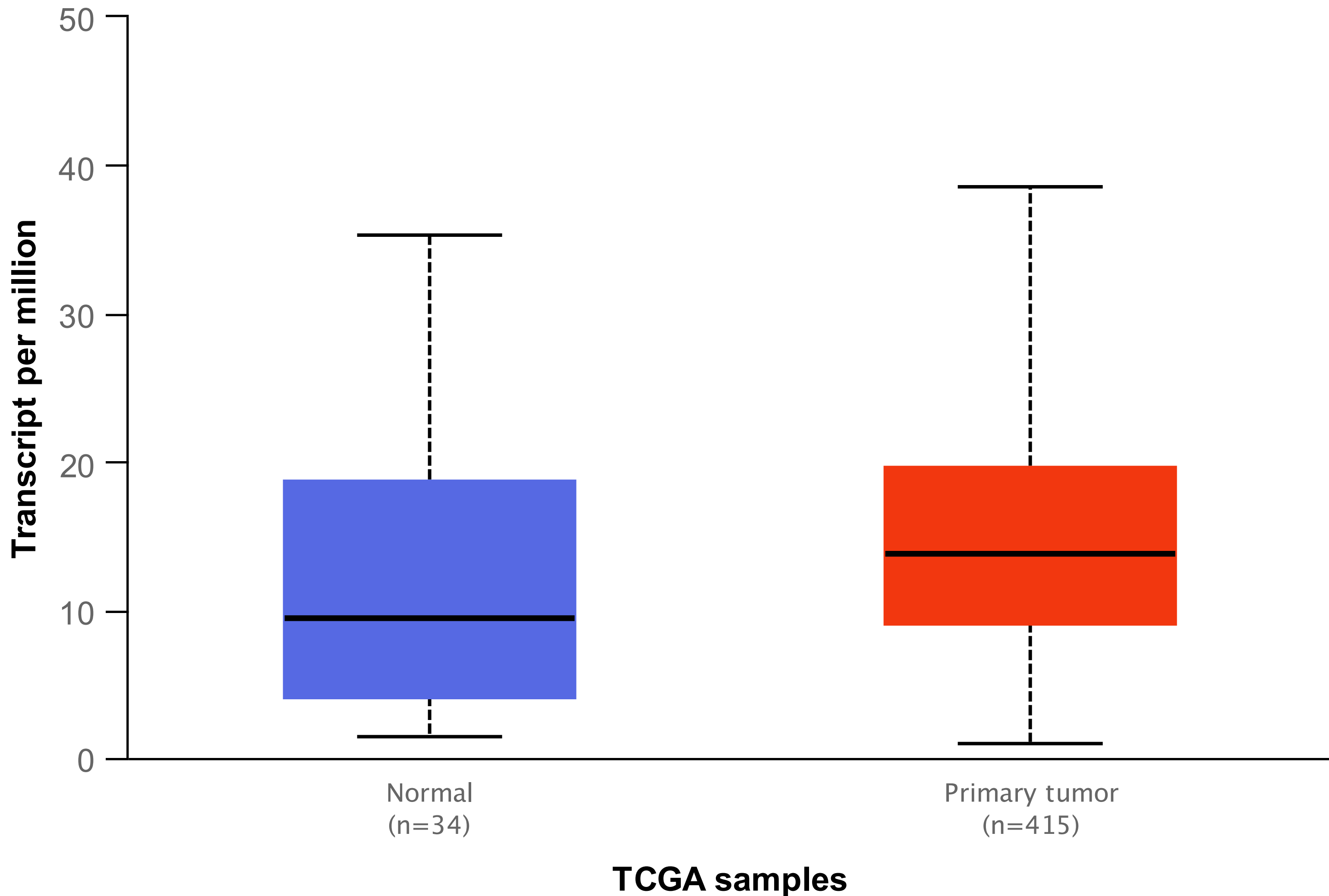

Supplement: Supplementary file 2 [file Data_Sheet_2.ZIP › Supplementary materials fig.2/UALCAN/expression-of-fam83g-in.pdf]

# Expression of FAM83H in STAD based on Sample types

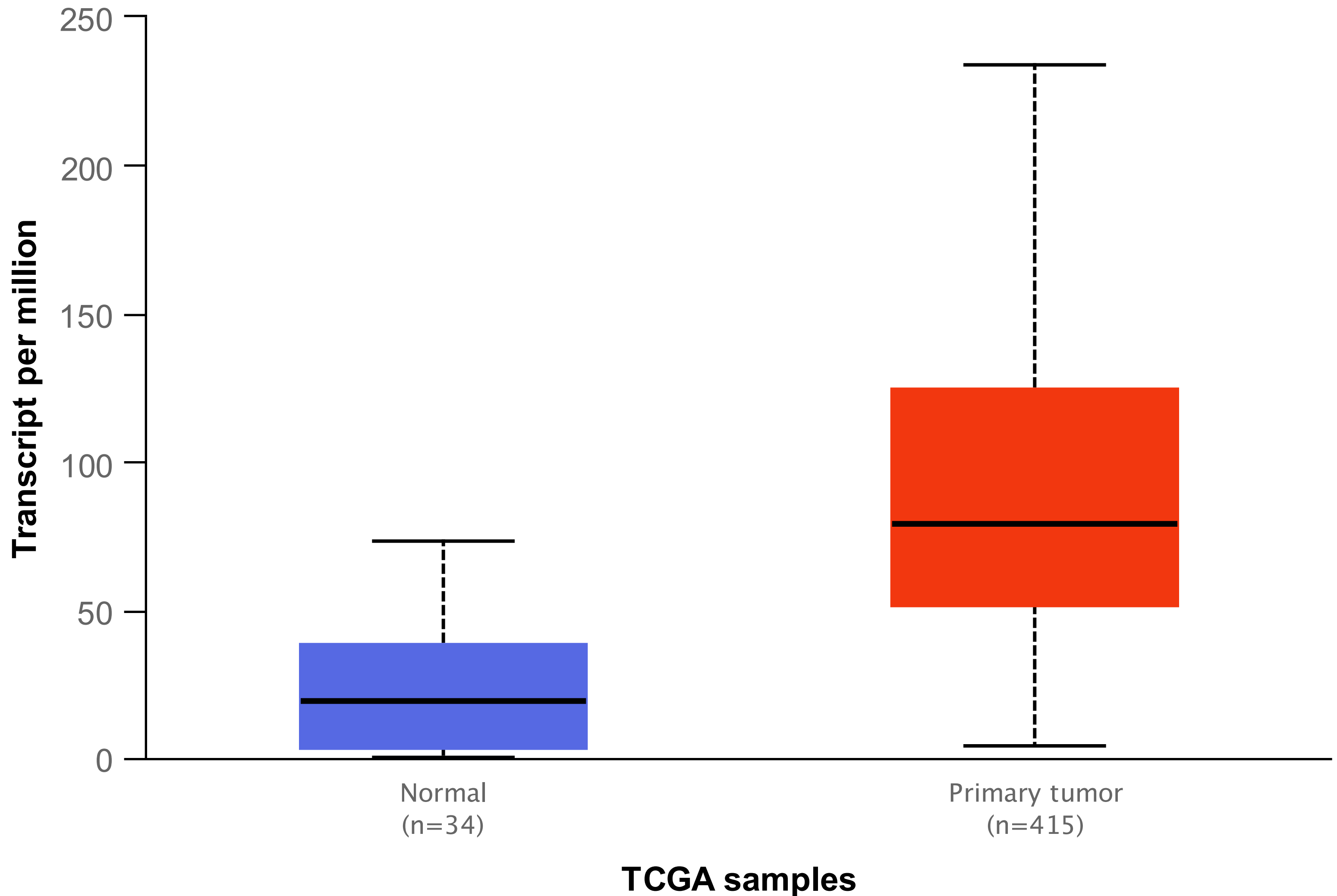

Supplement: Supplementary file 2 [file Data_Sheet_2.ZIP › Supplementary materials fig.2/UALCAN/expression-of-fam83h-in.pdf]

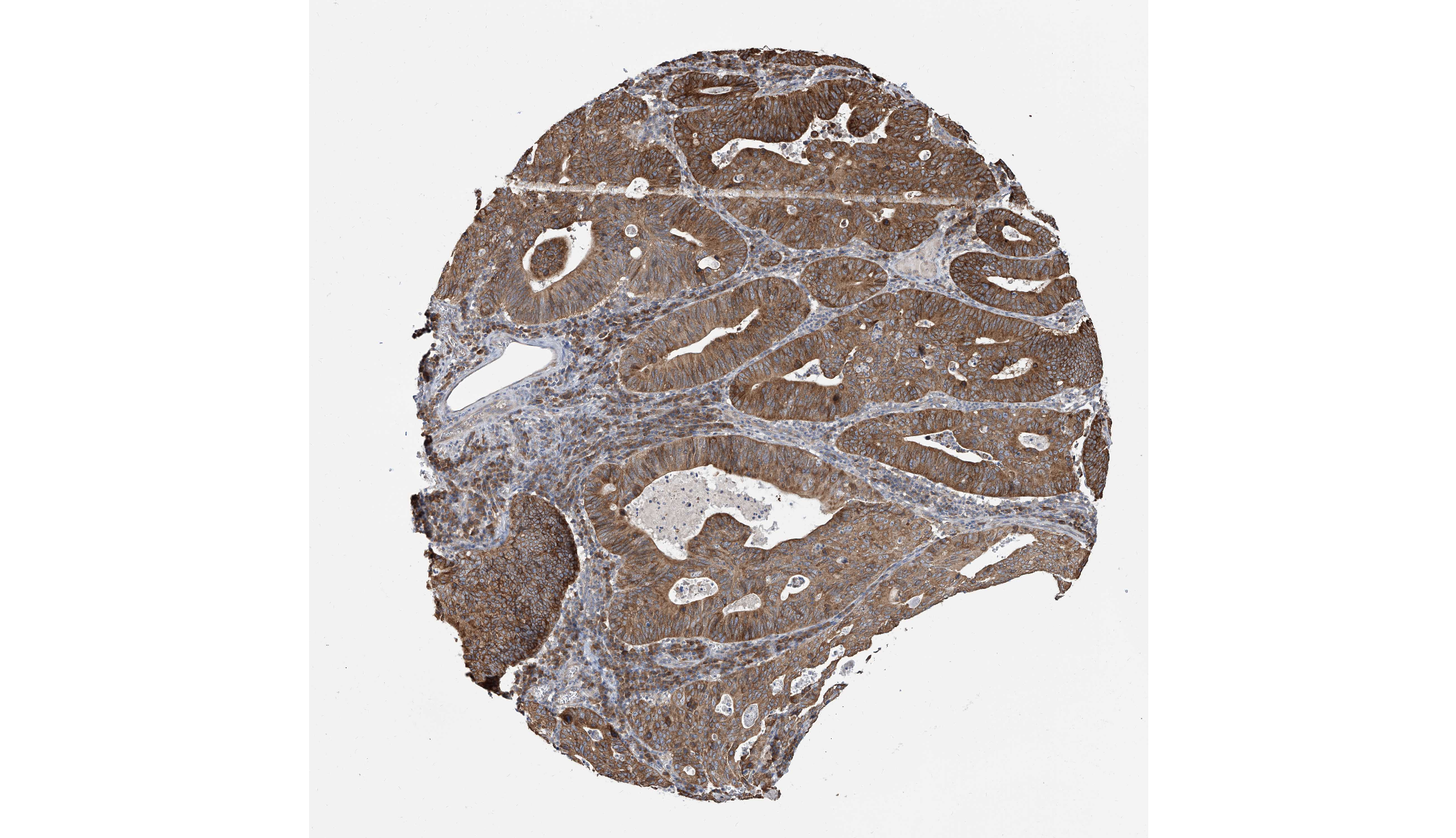

Supplement: Supplementary file 3 [file Data_Sheet_3.ZIP › Supplementary materials fig.3(1)/FAM83B(immunohistochemistry images)/cancer1.png]

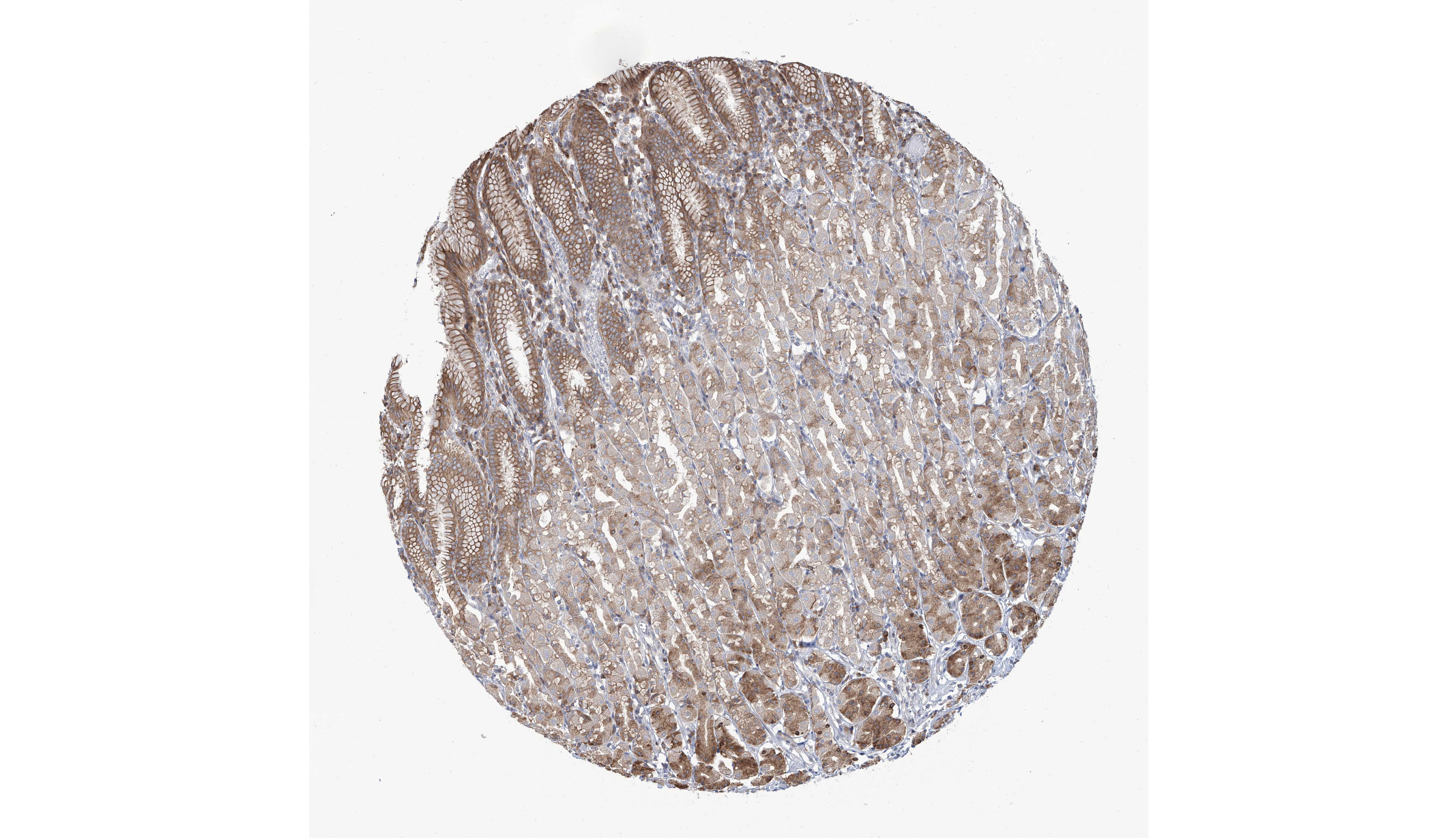

Supplement: Supplementary file 3 [file Data_Sheet_3.ZIP › Supplementary materials fig.3(1)/FAM83B(immunohistochemistry images)/normal1.png]

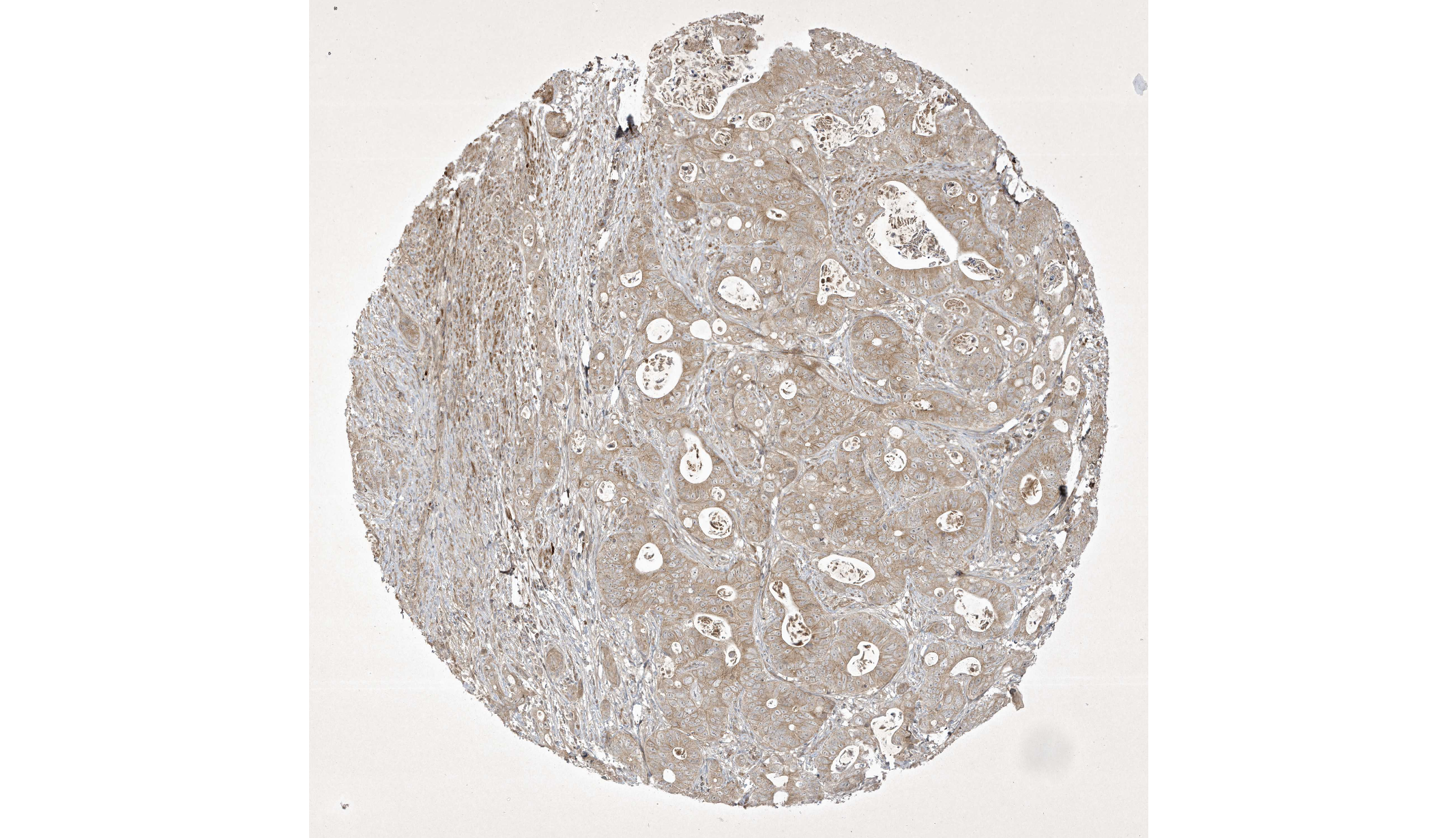

Supplement: Supplementary file 3 [file Data_Sheet_3.ZIP › Supplementary materials fig.3(1)/FAM83C(immunohistochemistry images)/cancer1.png]

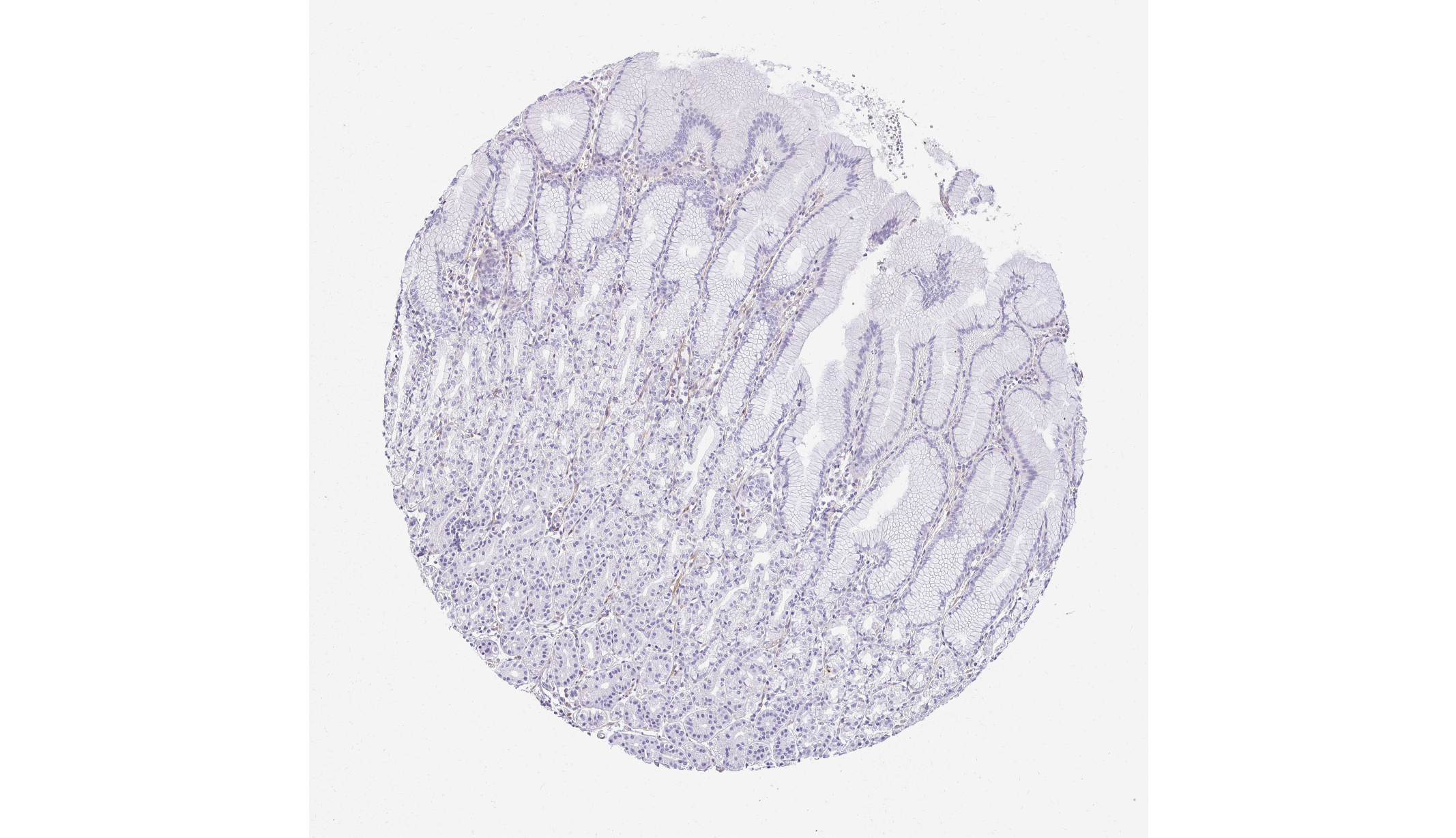

Supplement: Supplementary file 3 [file Data_Sheet_3.ZIP › Supplementary materials fig.3(1)/FAM83C(immunohistochemistry images)/normal1.png]

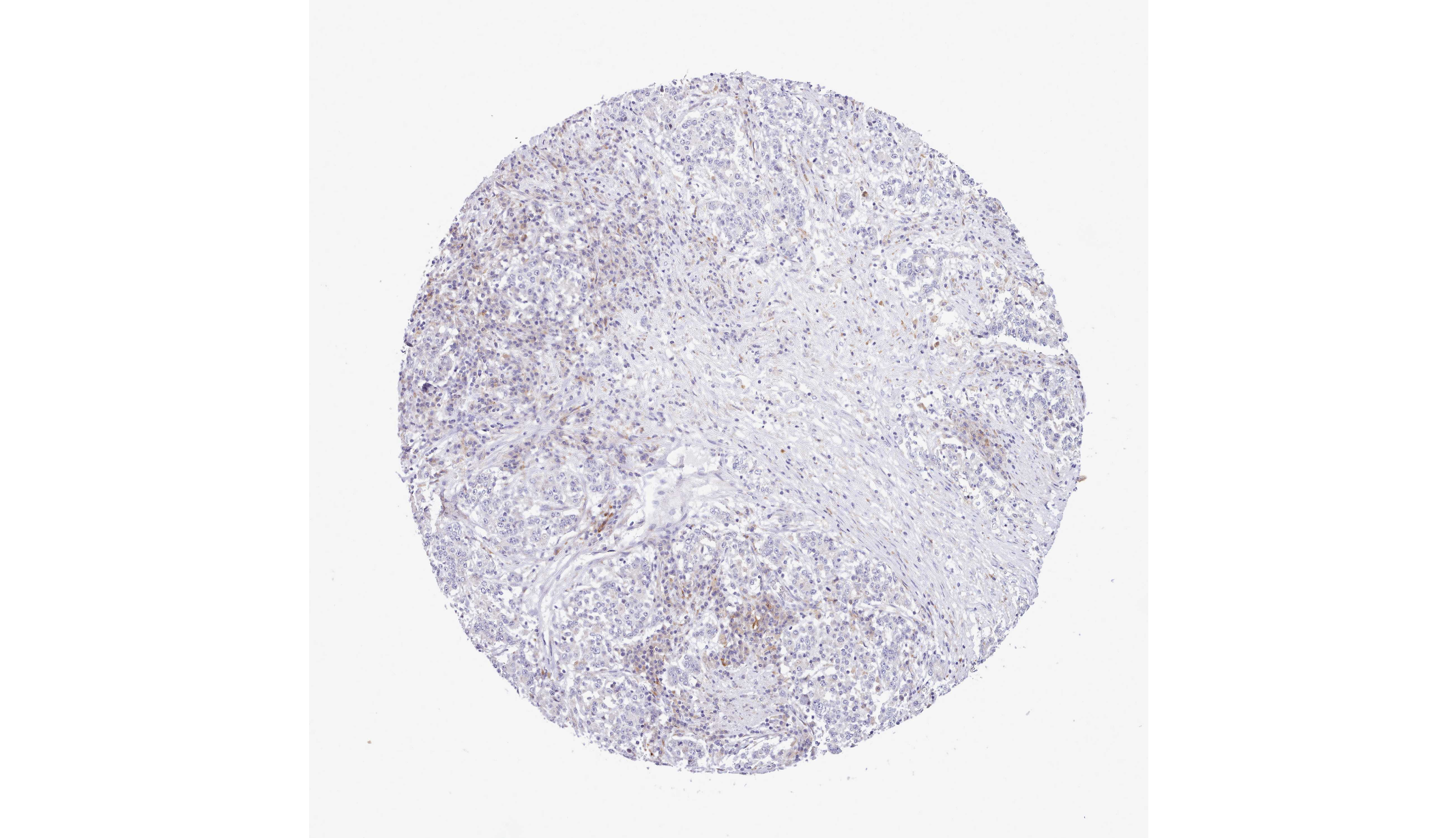

Supplement: Supplementary file 3 [file Data_Sheet_3.ZIP › Supplementary materials fig.3(1)/FAM83E(immunohistochemistry images)/cancer1.png]

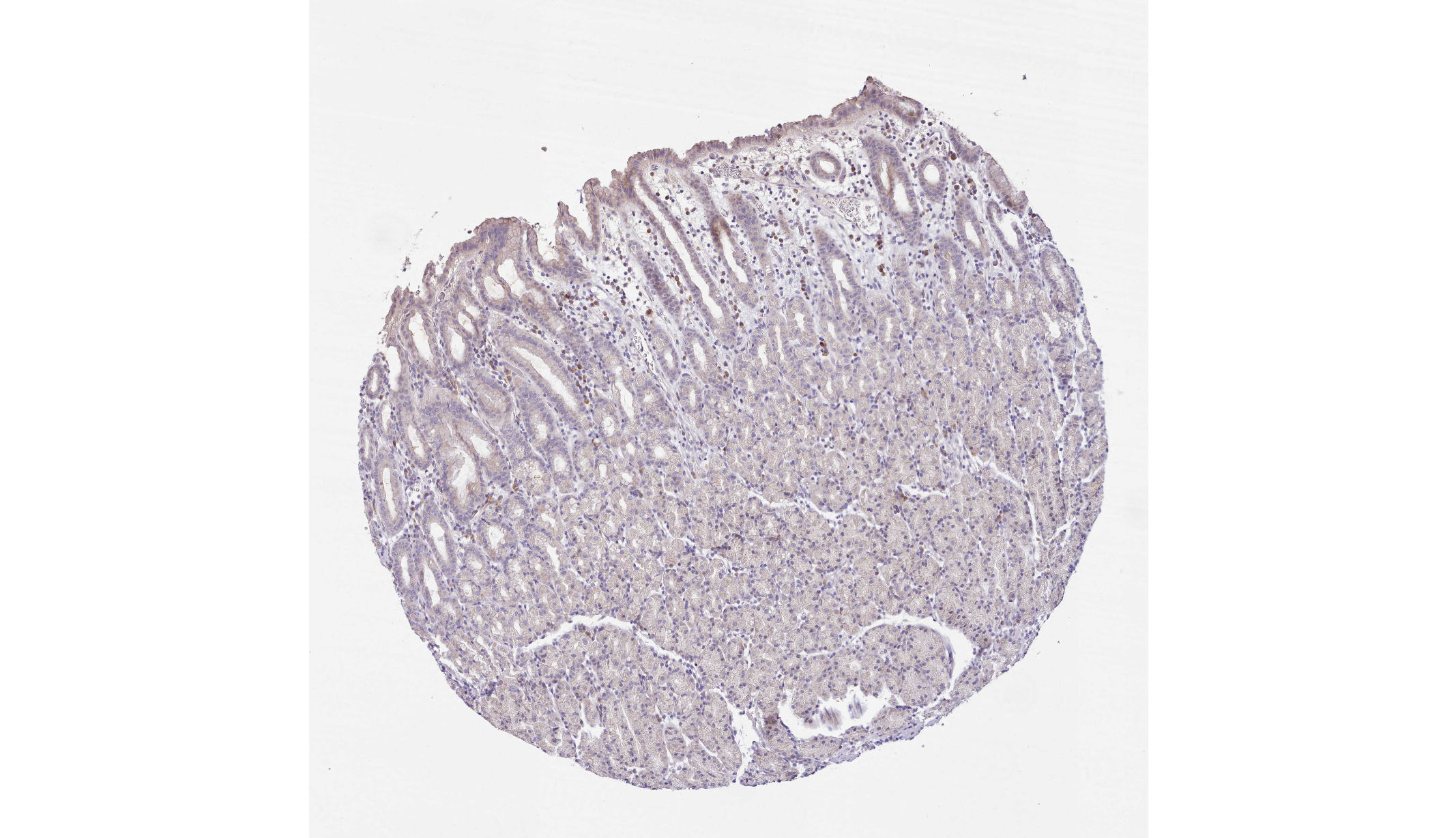

Supplement: Supplementary file 3 [file Data_Sheet_3.ZIP › Supplementary materials fig.3(1)/FAM83E(immunohistochemistry images)/normal1.png]

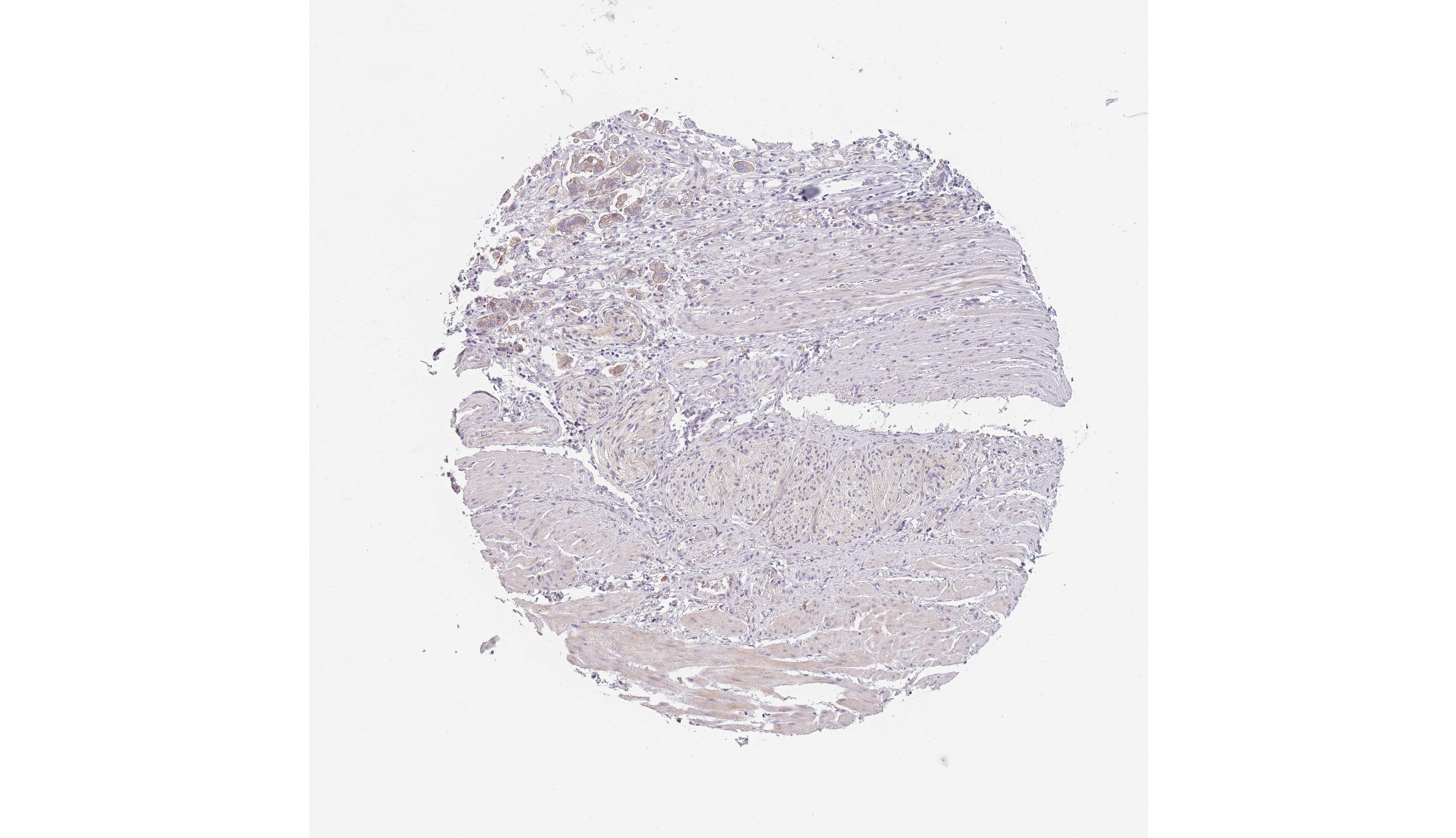

Supplement: Supplementary file 4 [file Data_Sheet_4.ZIP › Supplementary materials fig.3(2)/FAM83F(immunohistochemistry images)/cancer1.png]

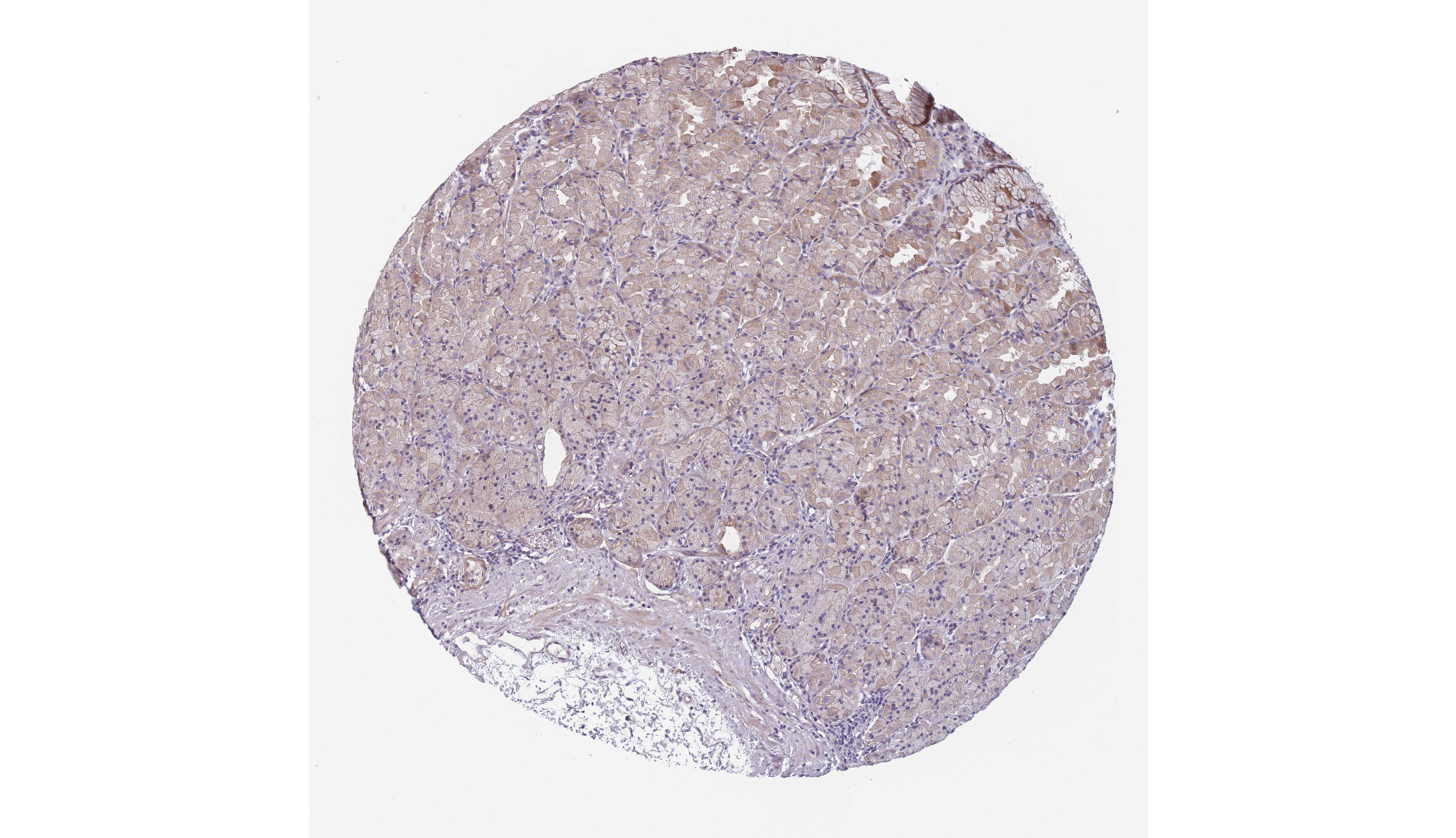

Supplement: Supplementary file 4 [file Data_Sheet_4.ZIP › Supplementary materials fig.3(2)/FAM83F(immunohistochemistry images)/normal1.png]

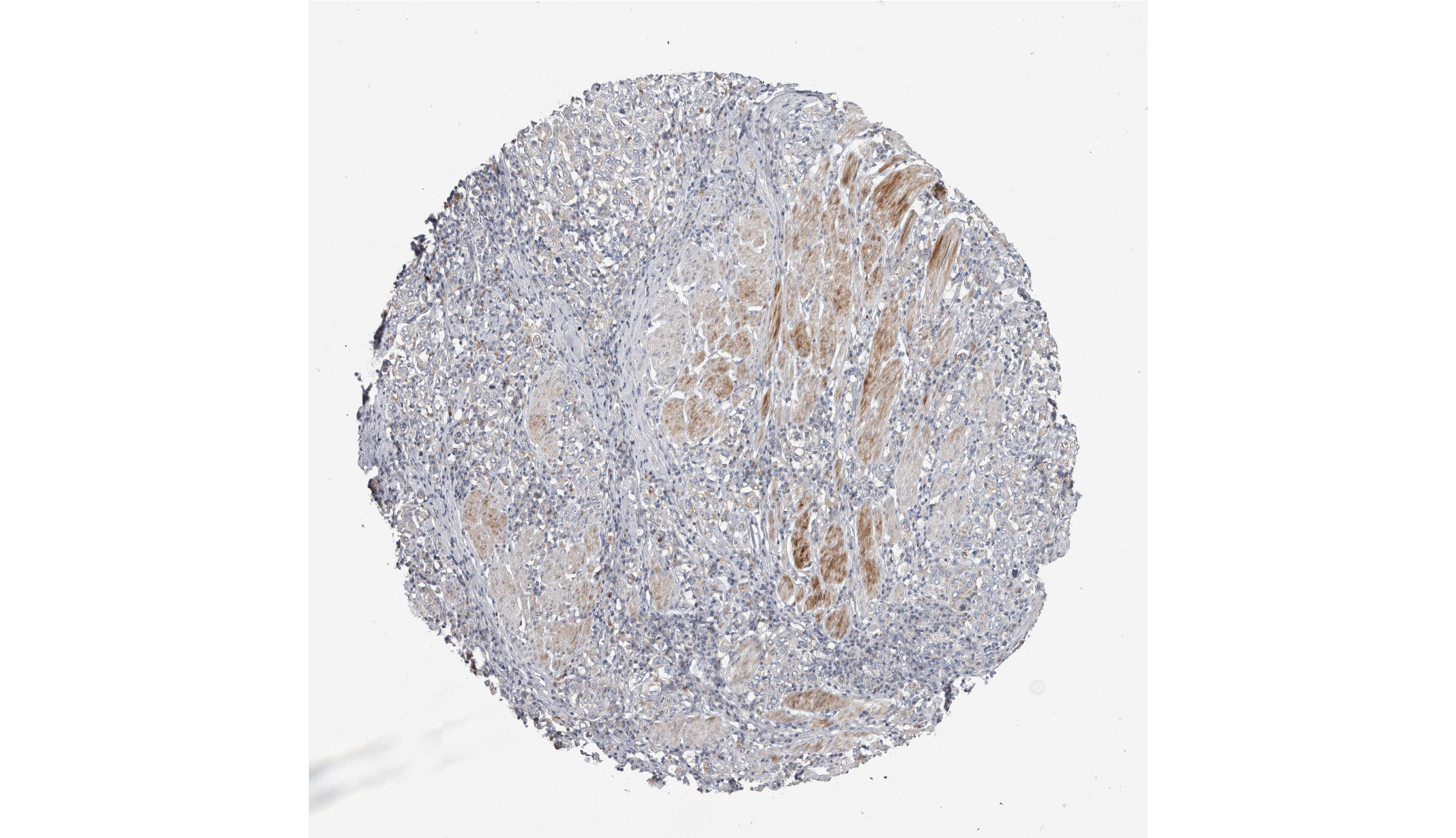

Supplement: Supplementary file 4 [file Data_Sheet_4.ZIP › Supplementary materials fig.3(2)/FAM83G(immunohistochemistry images)/cancer1.png]

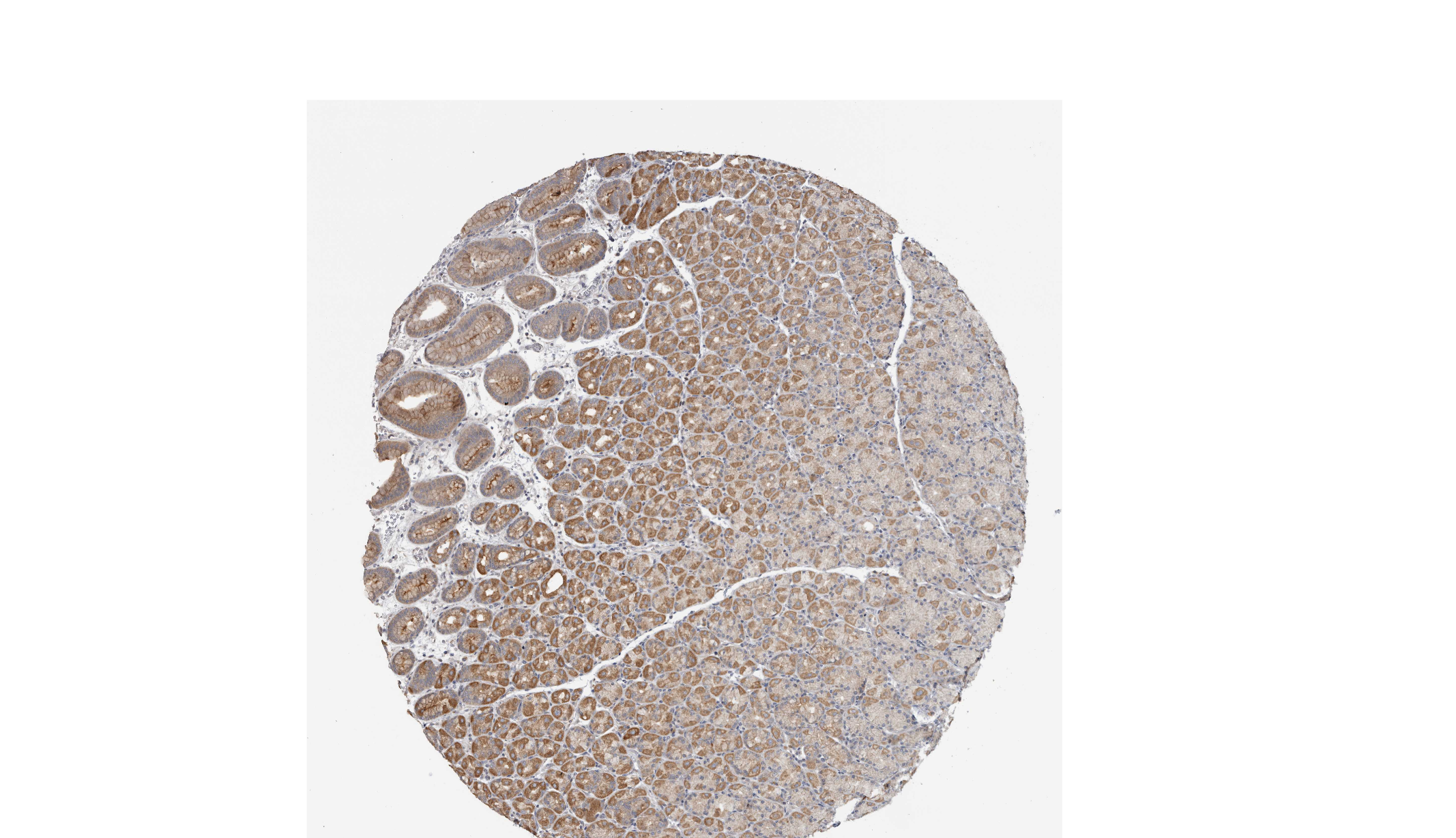

Supplement: Supplementary file 4 [file Data_Sheet_4.ZIP › Supplementary materials fig.3(2)/FAM83G(immunohistochemistry images)/normal1.png]

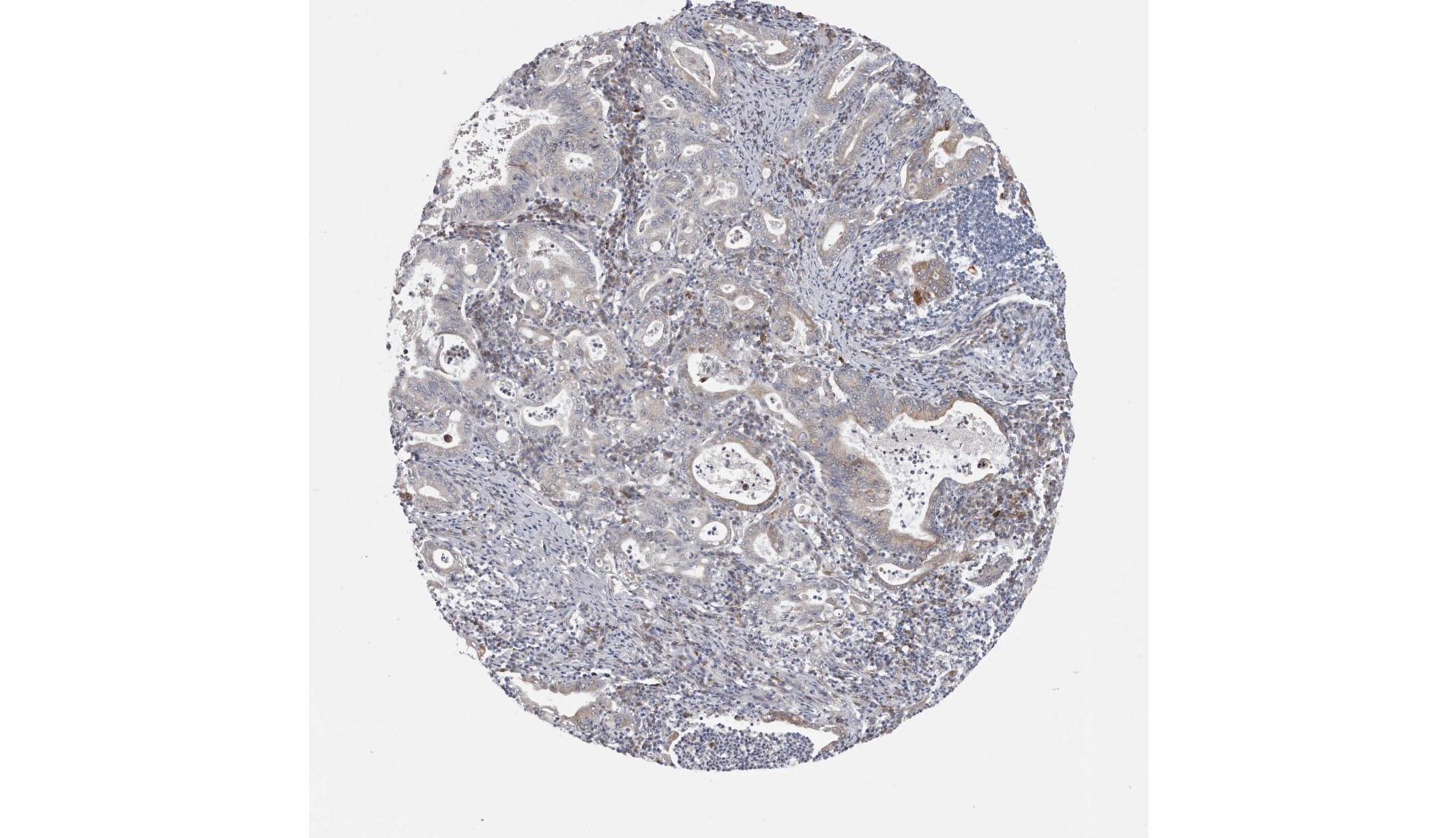

Supplement: Supplementary file 4 [file Data_Sheet_4.ZIP › Supplementary materials fig.3(2)/FAM83H(immunohistochemistry images)/cancer1.png]

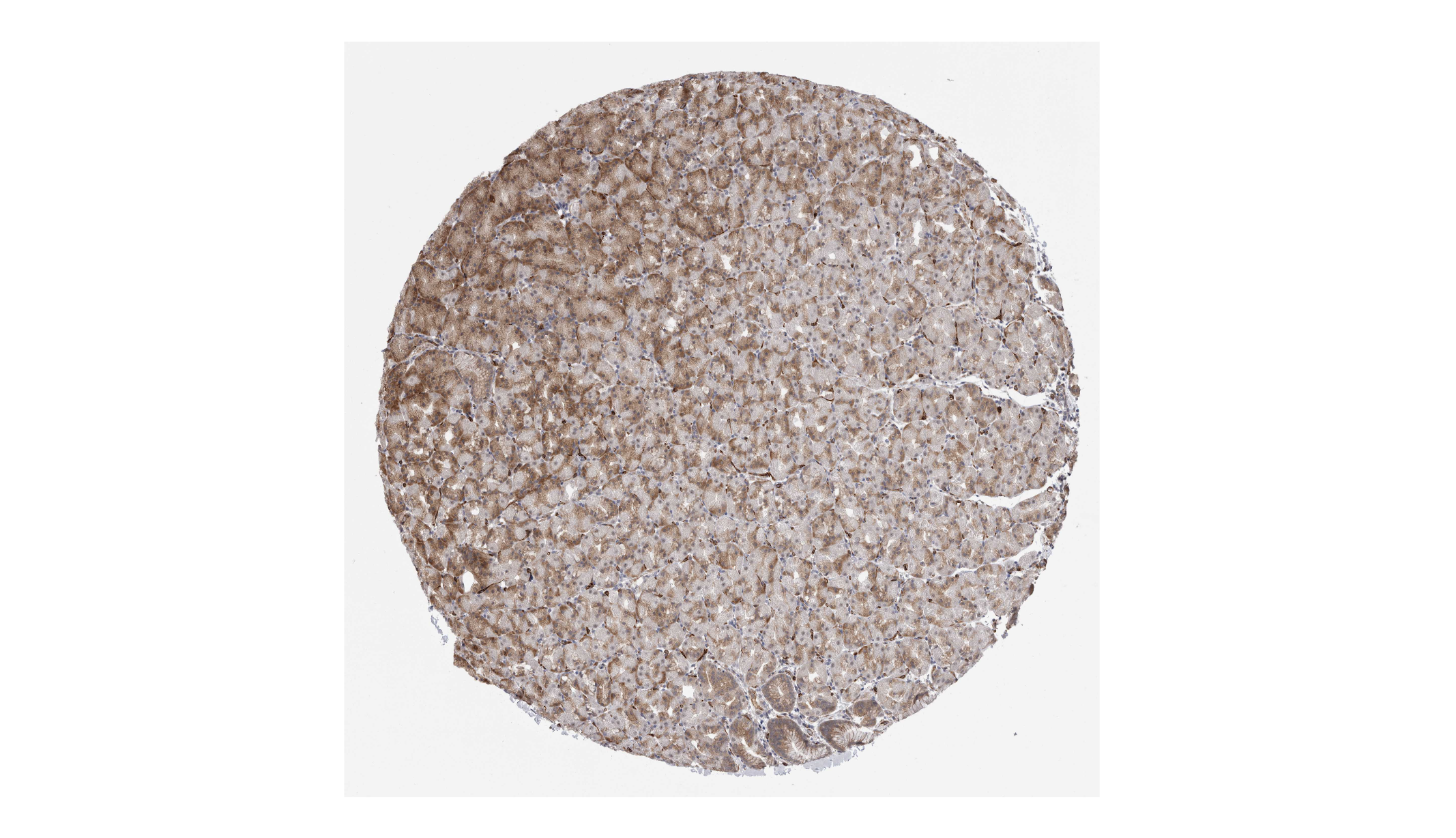

Supplement: Supplementary file 4 [file Data_Sheet_4.ZIP › Supplementary materials fig.3(2)/FAM83H(immunohistochemistry images)/normal1.png]

# Expression of FAM83A in STAD based on individual cancer stages

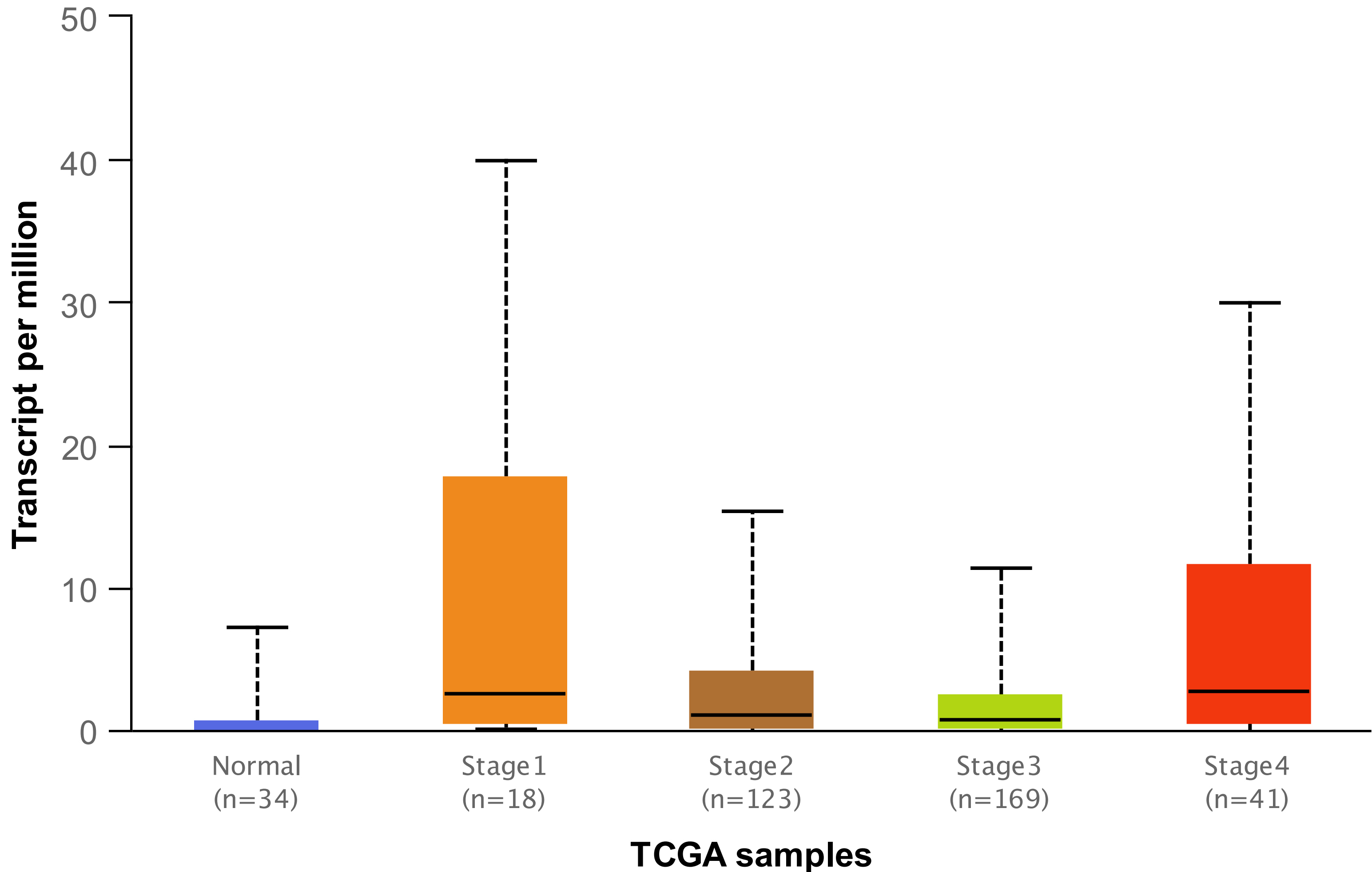

Supplement: Supplementary file 5 [file Data_Sheet_5.ZIP › Supplementary materials fig.4/expression-of-fam83a-in.pdf]

# Expression of FAM83B in STAD based on individual cancer stages

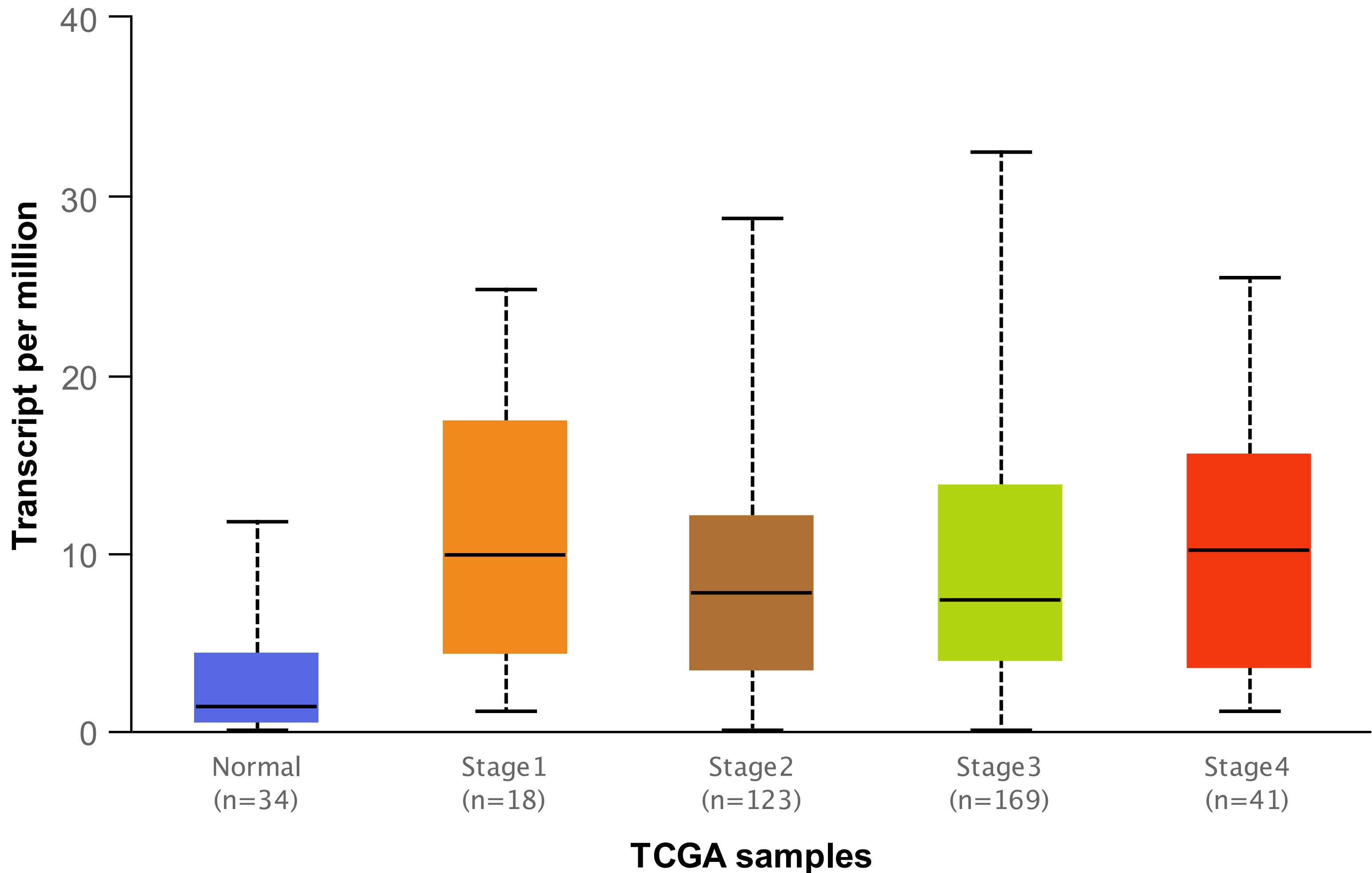

Supplement: Supplementary file 5 [file Data_Sheet_5.ZIP › Supplementary materials fig.4/expression-of-fam83b-in.pdf]

# Expression of FAM83C in STAD based on individual cancer stages

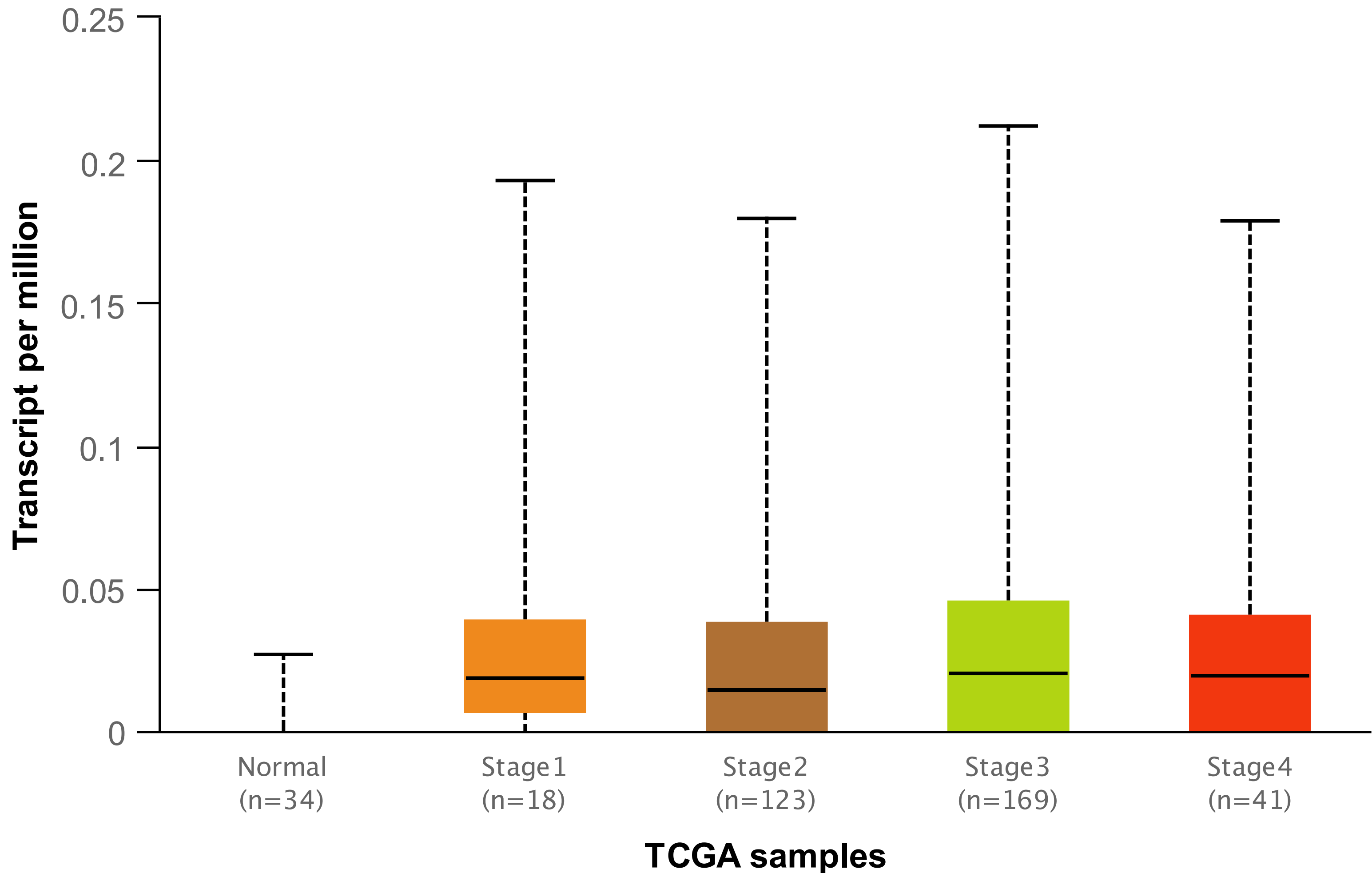

Supplement: Supplementary file 5 [file Data_Sheet_5.ZIP › Supplementary materials fig.4/expression-of-fam83c-in.pdf]

# Expression of FAM83D in STAD based on individual cancer stages

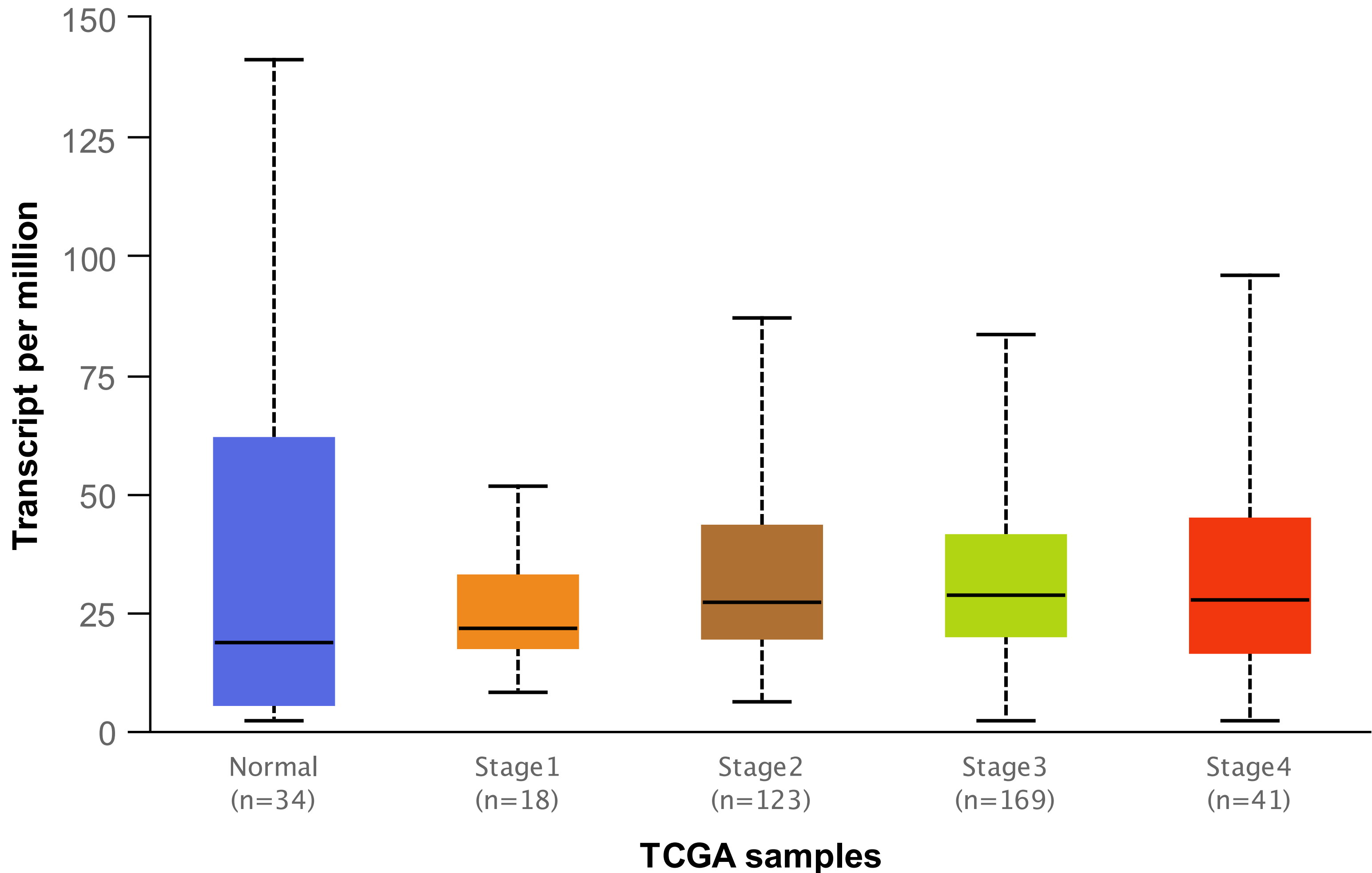

Supplement: Supplementary file 5 [file Data_Sheet_5.ZIP › Supplementary materials fig.4/expression-of-fam83d-in.pdf]

# Expression of FAM83E in STAD based on individual cancer stages

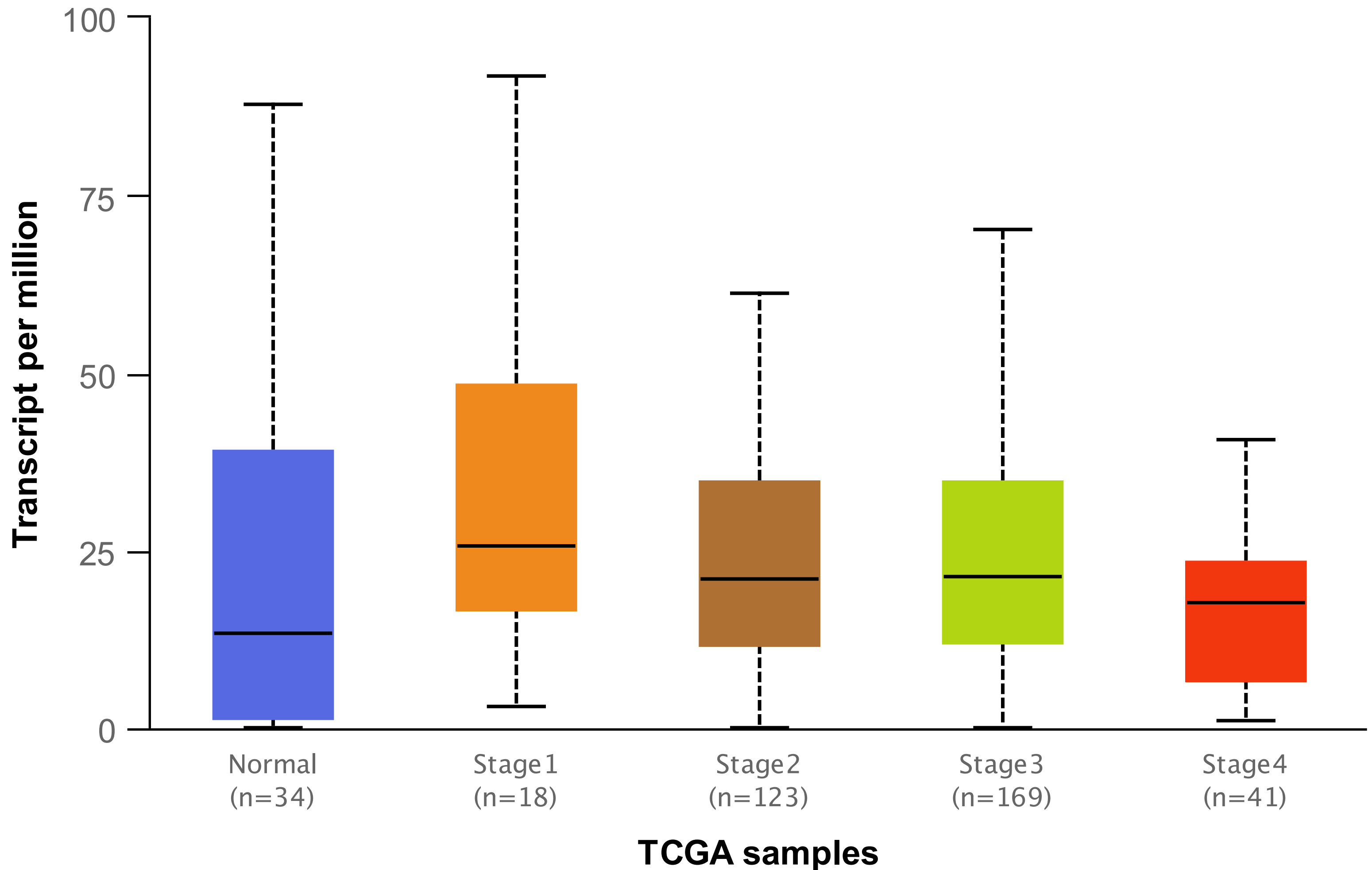

Supplement: Supplementary file 5 [file Data_Sheet_5.ZIP › Supplementary materials fig.4/expression-of-fam83e-in.pdf]

# Expression of FAM83F in STAD based on individual cancer stages

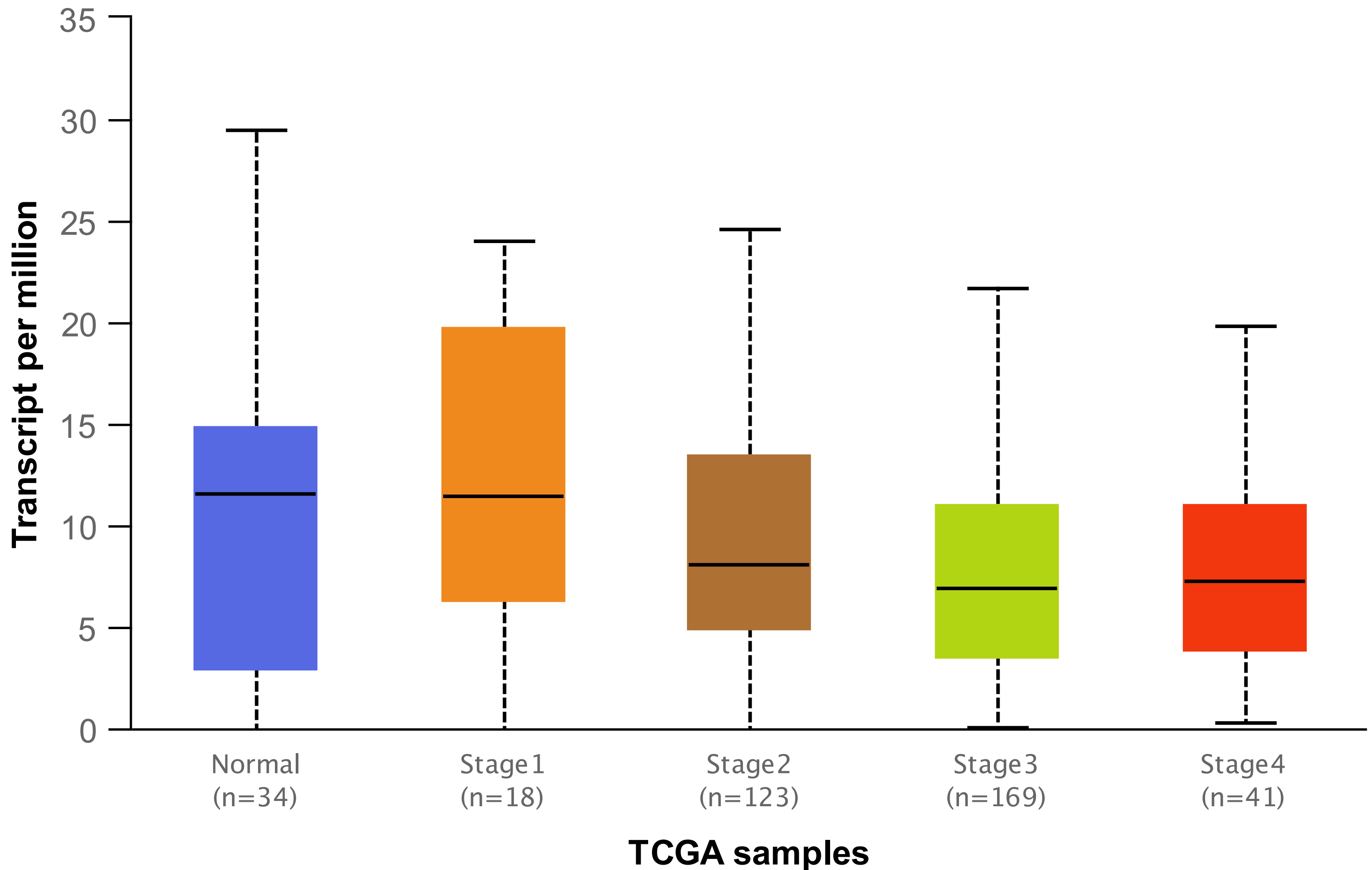

Supplement: Supplementary file 5 [file Data_Sheet_5.ZIP › Supplementary materials fig.4/expression-of-fam83f-in.pdf]

# Expression of FAM83G in STAD based on individual cancer stages

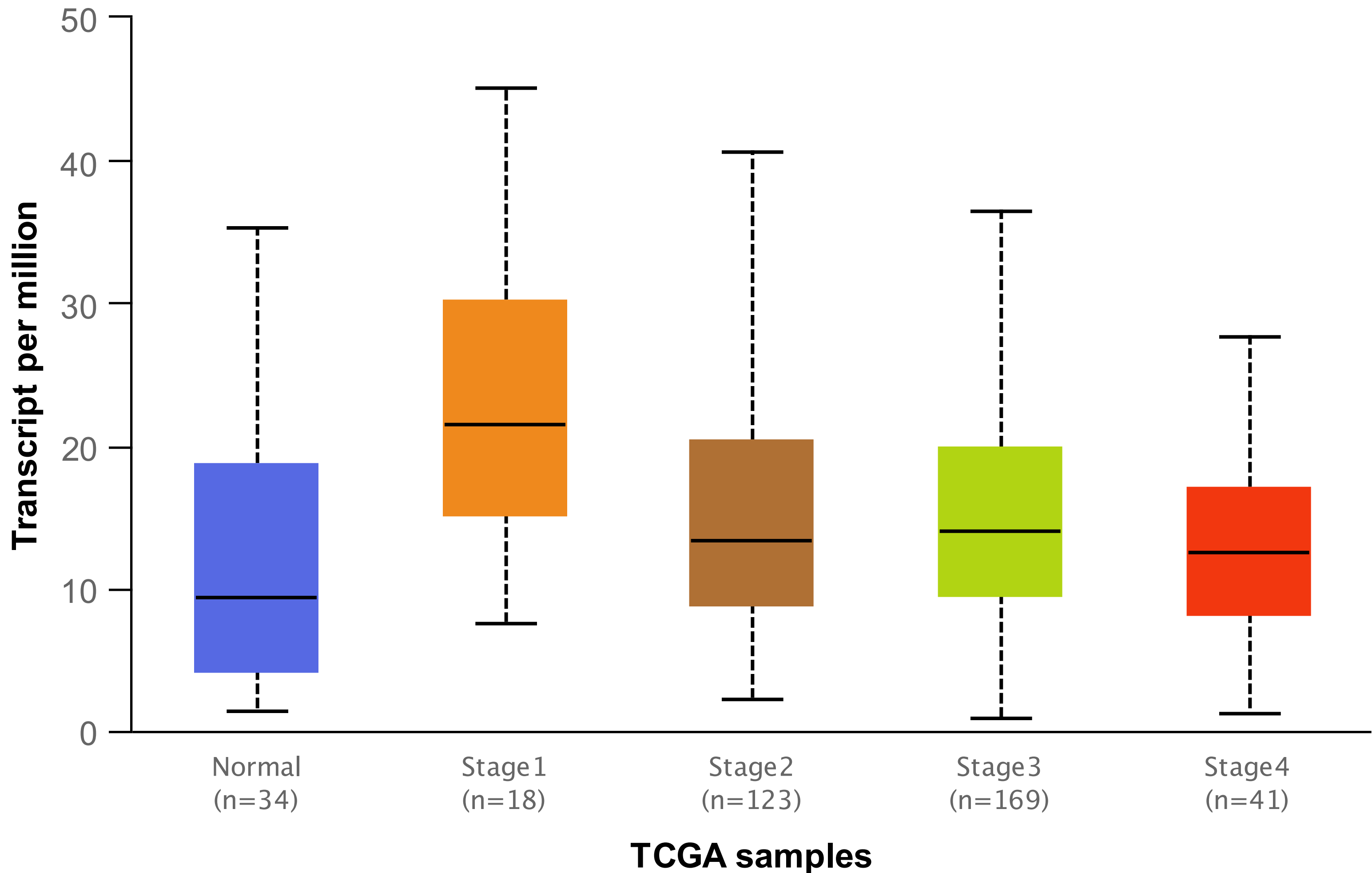

Supplement: Supplementary file 5 [file Data_Sheet_5.ZIP › Supplementary materials fig.4/expression-of-fam83g-in.pdf]

# Expression of FAM83H in STAD based on individual cancer stages

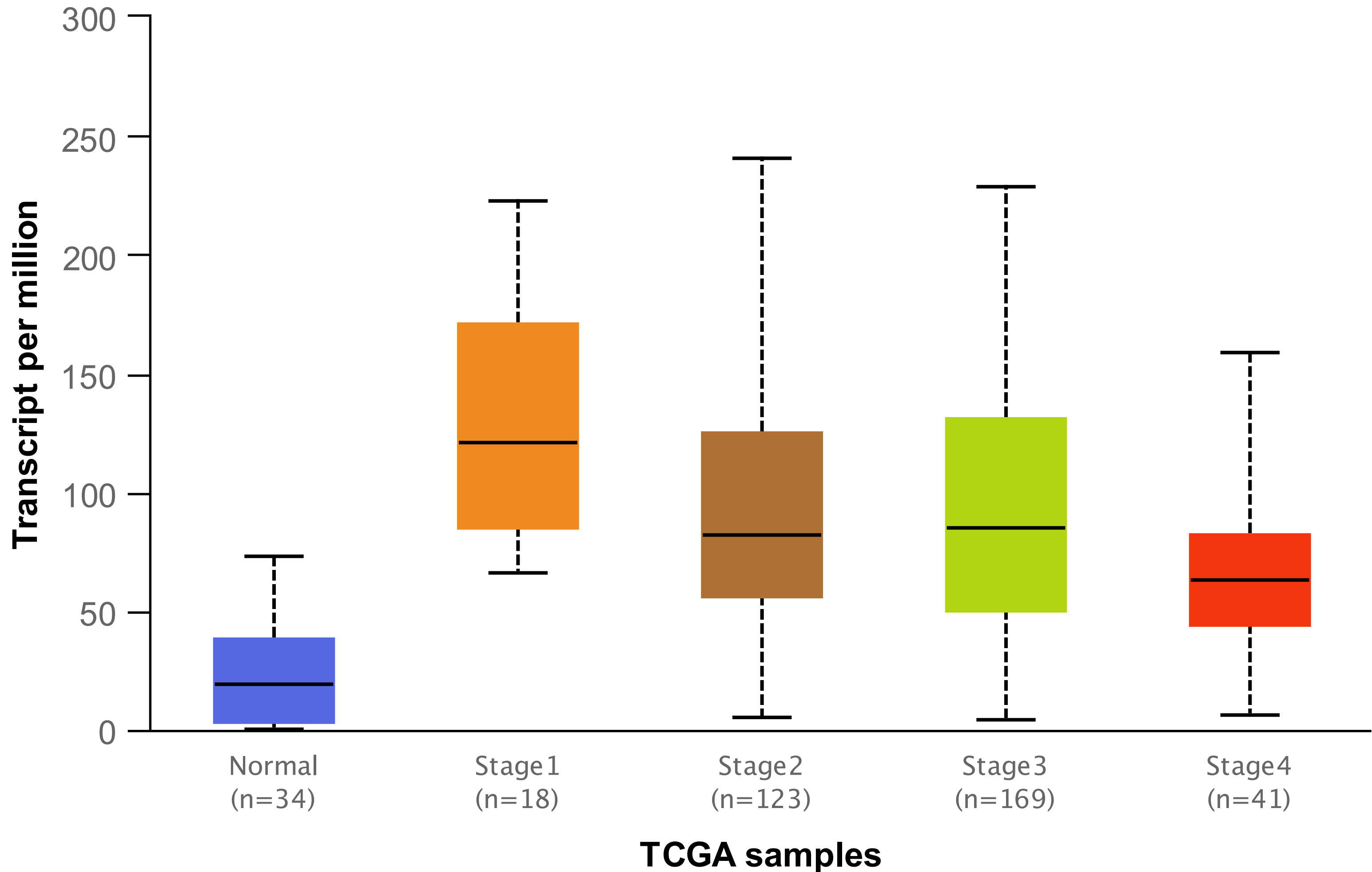

Supplement: Supplementary file 5 [file Data_Sheet_5.ZIP › Supplementary materials fig.4/expression-of-fam83h-in.pdf]

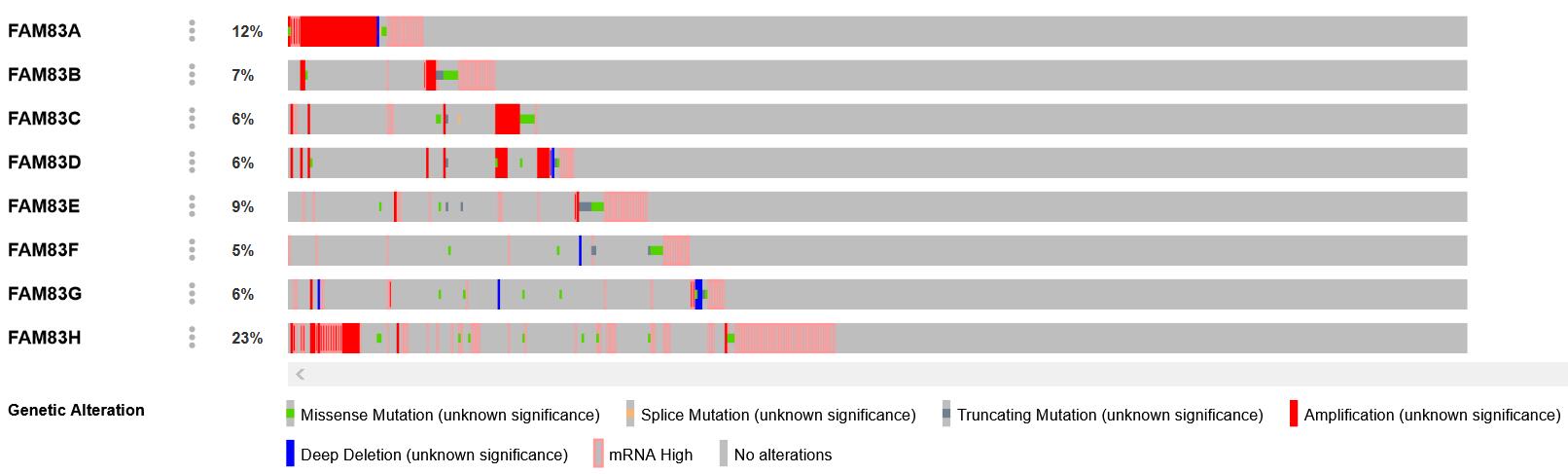

Supplement: Supplementary file 6 [file Data_Sheet_6.ZIP › Supplementary materials fig.5/fig5A.jpg]

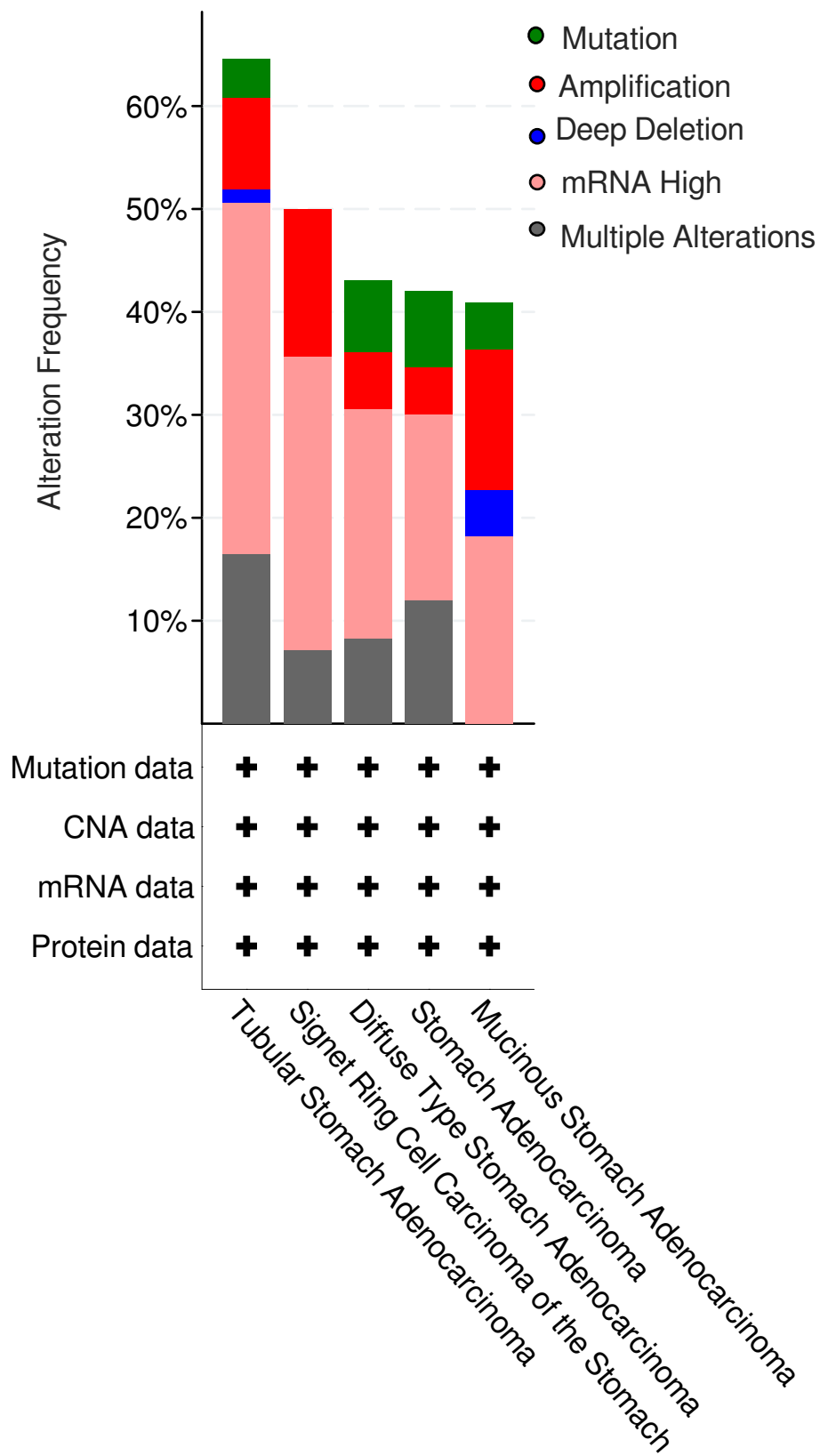

Supplement: Supplementary file 6 [file Data_Sheet_6.ZIP › Supplementary materials fig.5/fig5B.pdf]

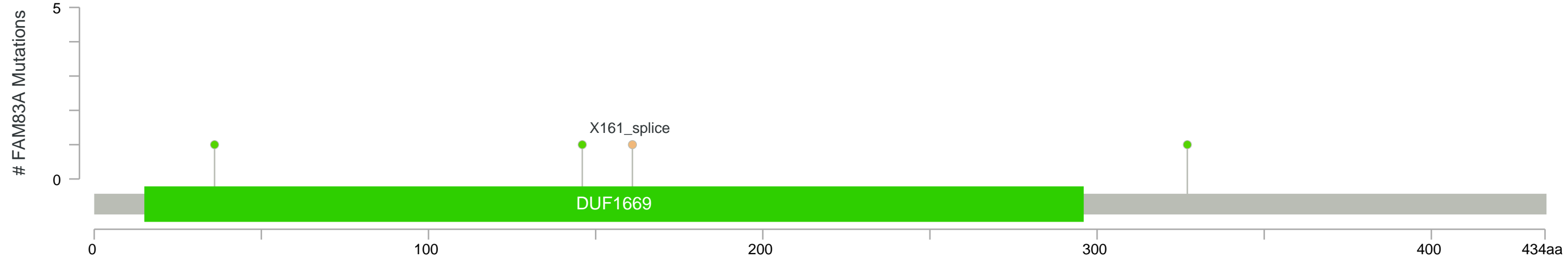

Supplement: Supplementary file 6 [file Data_Sheet_6.ZIP › Supplementary materials fig.5/fig5C/FAM83A_lollipop.svg.pdf]

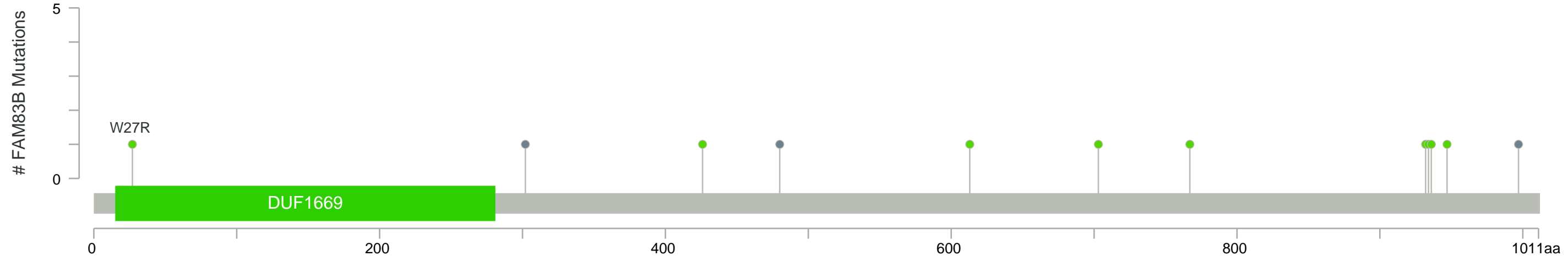

Supplement: Supplementary file 6 [file Data_Sheet_6.ZIP › Supplementary materials fig.5/fig5C/FAM83B_lollipop.svg.pdf]

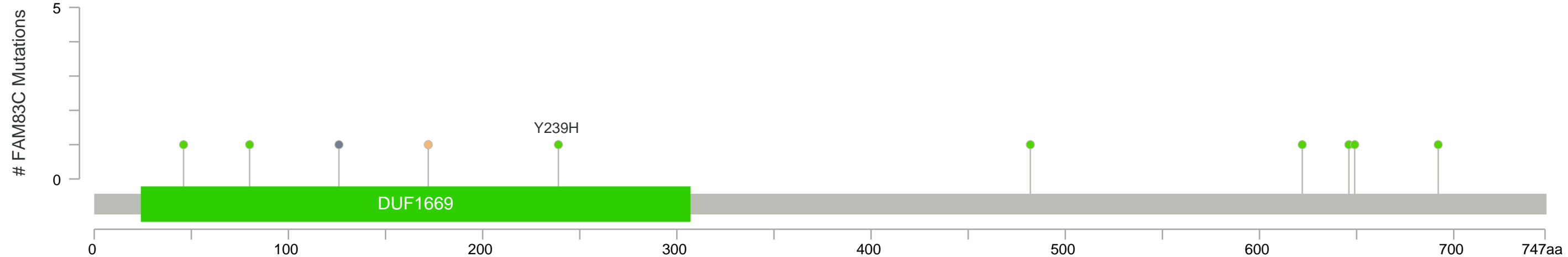

Supplement: Supplementary file 6 [file Data_Sheet_6.ZIP › Supplementary materials fig.5/fig5C/FAM83C_lollipop.svg.pdf]

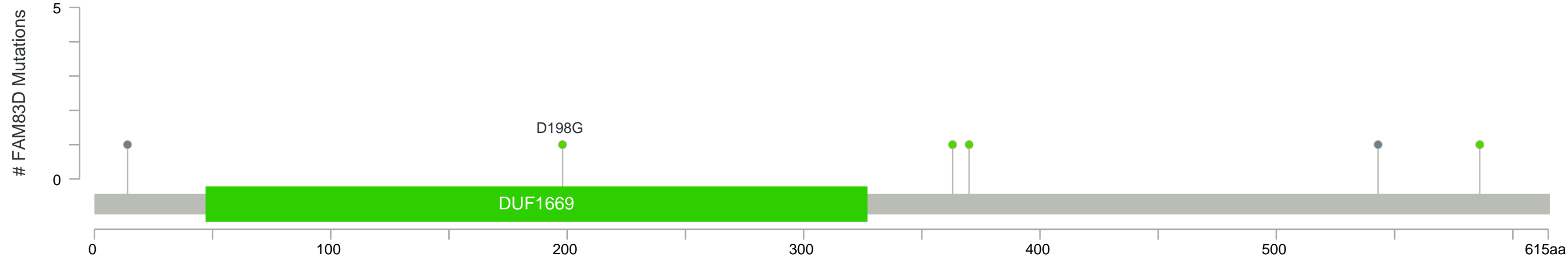

Supplement: Supplementary file 6 [file Data_Sheet_6.ZIP › Supplementary materials fig.5/fig5C/FAM83D_lollipop.svg.pdf]

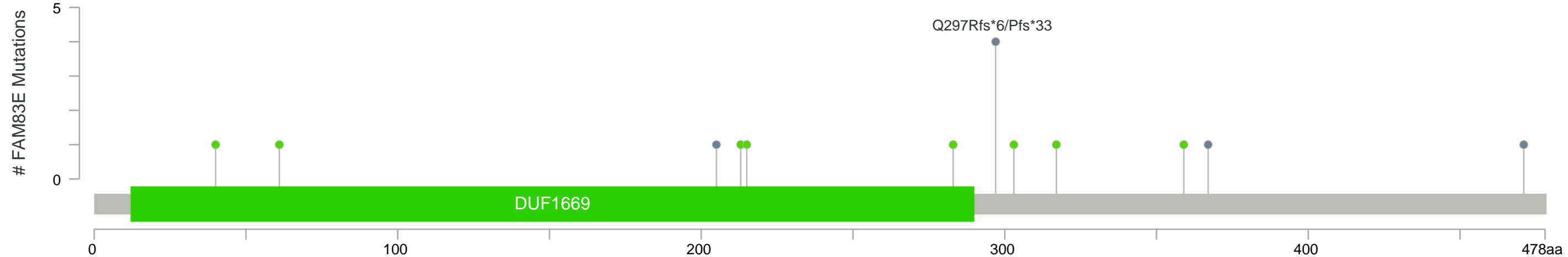

Supplement: Supplementary file 6 [file Data_Sheet_6.ZIP › Supplementary materials fig.5/fig5C/FAM83E_lollipop.svg.pdf]

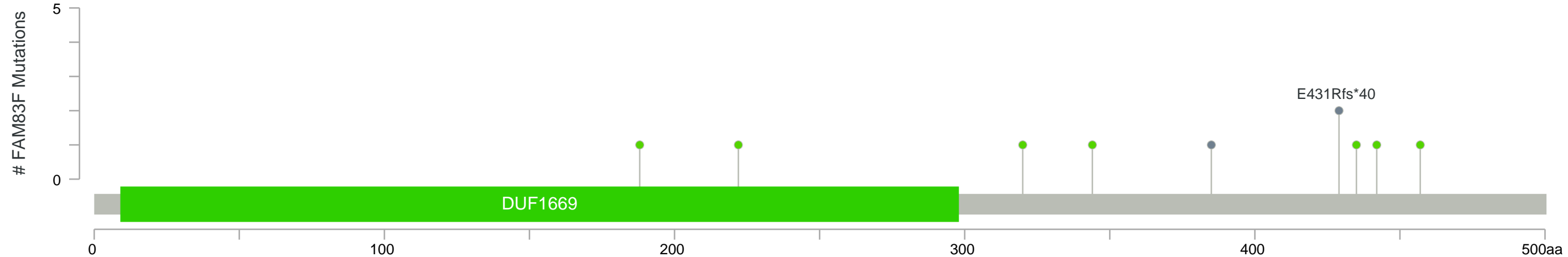

Supplement: Supplementary file 6 [file Data_Sheet_6.ZIP › Supplementary materials fig.5/fig5C/FAM83F_lollipop.svg.pdf]

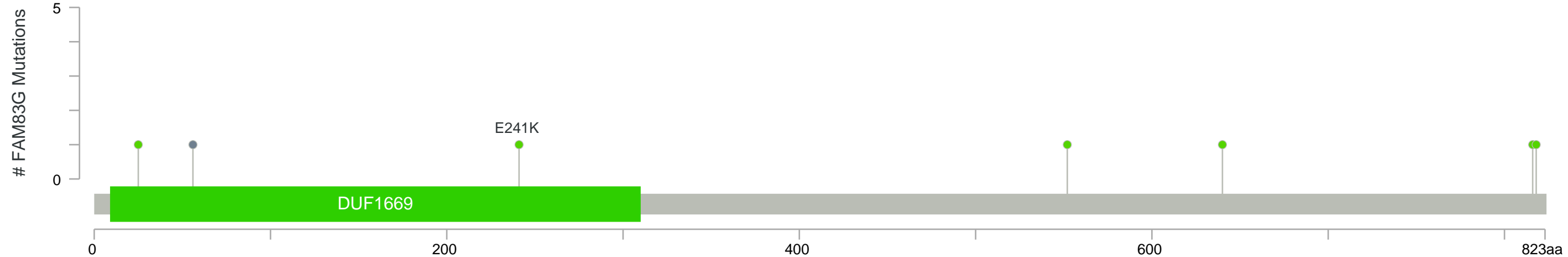

Supplement: Supplementary file 6 [file Data_Sheet_6.ZIP › Supplementary materials fig.5/fig5C/FAM83G_lollipop.svg.pdf]

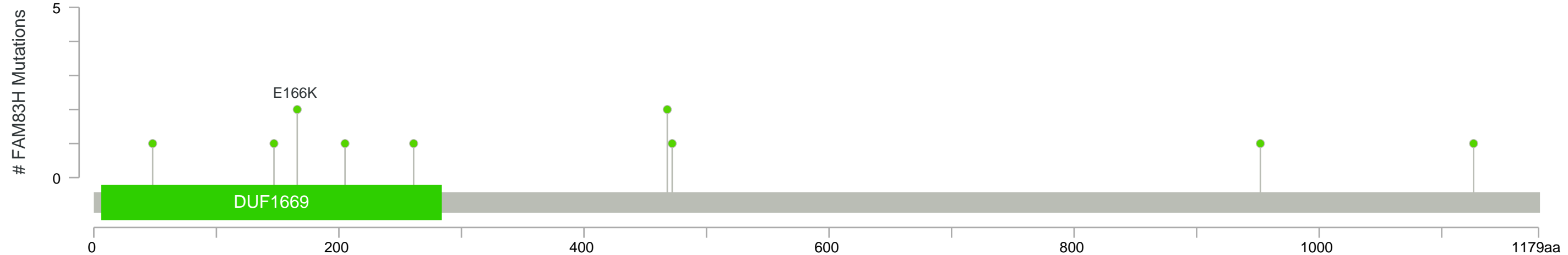

Supplement: Supplementary file 6 [file Data_Sheet_6.ZIP › Supplementary materials fig.5/fig5C/FAM83H_lollipop.svg.pdf]

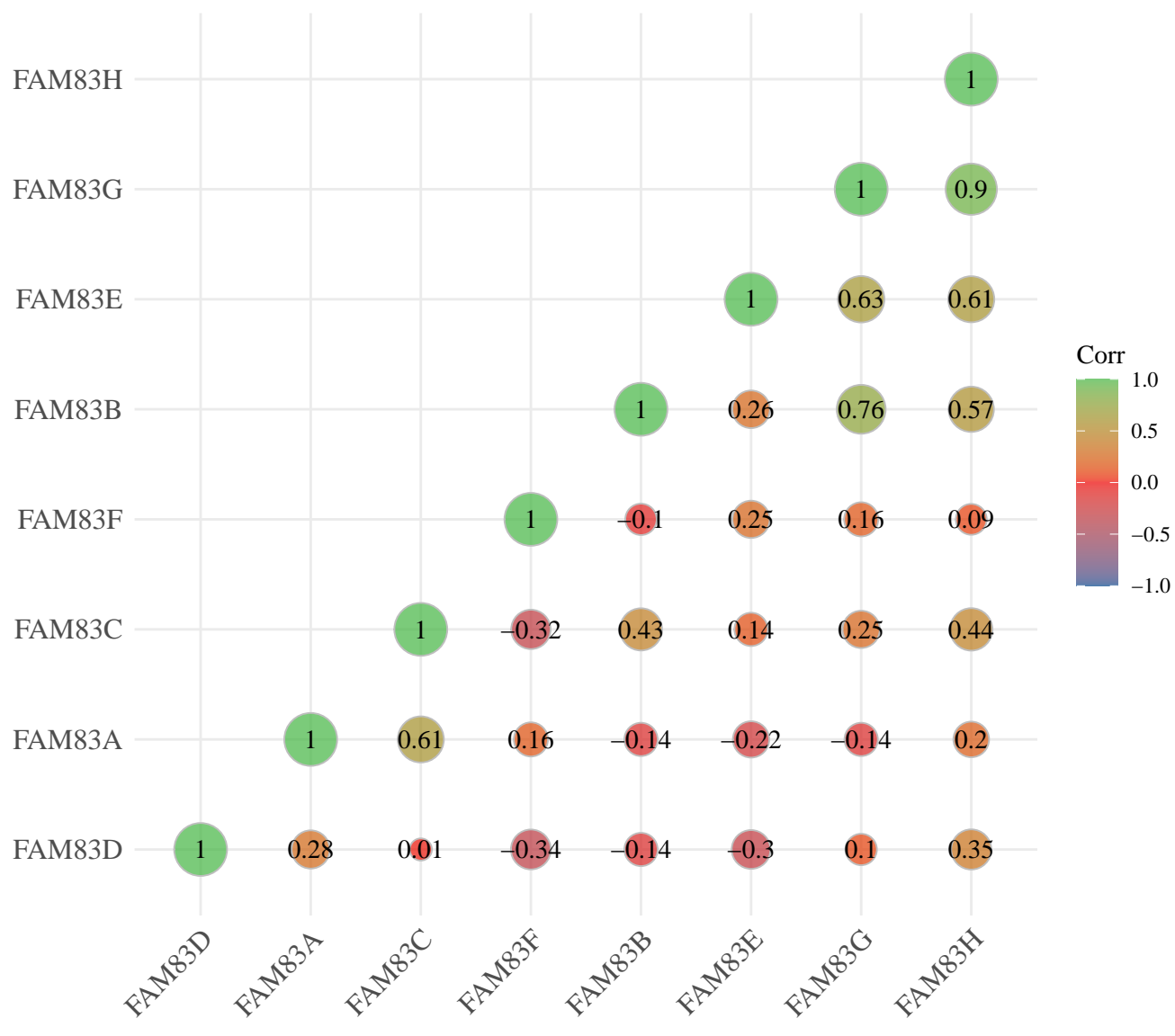

Supplement: Supplementary file 6 [file Data_Sheet_6.ZIP › Supplementary materials fig.5/fig5D/fig5D.pdf]

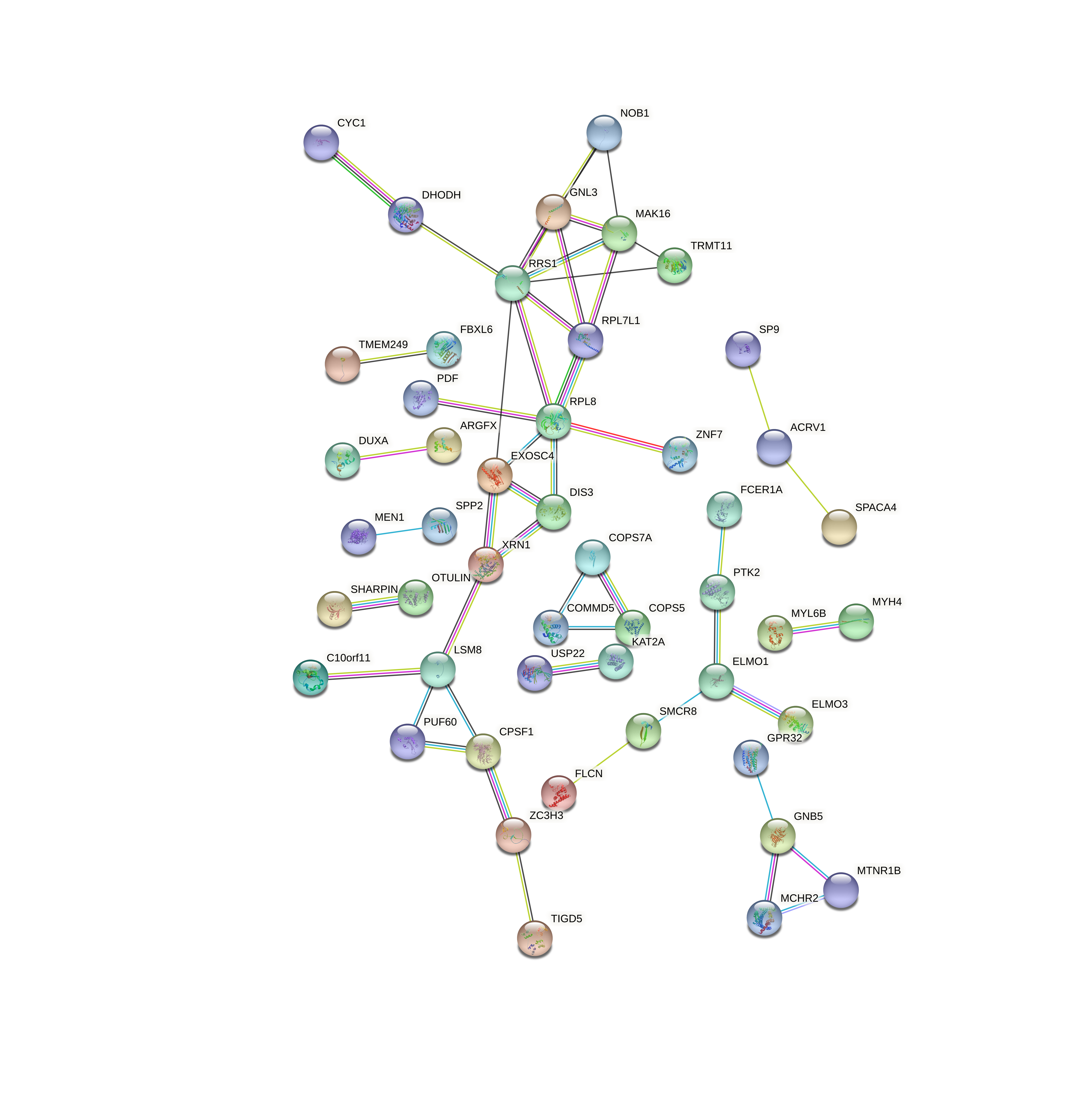

Supplement: Supplementary file 7 [file Data_Sheet_7.ZIP › Supplementary materials fig.6/fig6A.png]

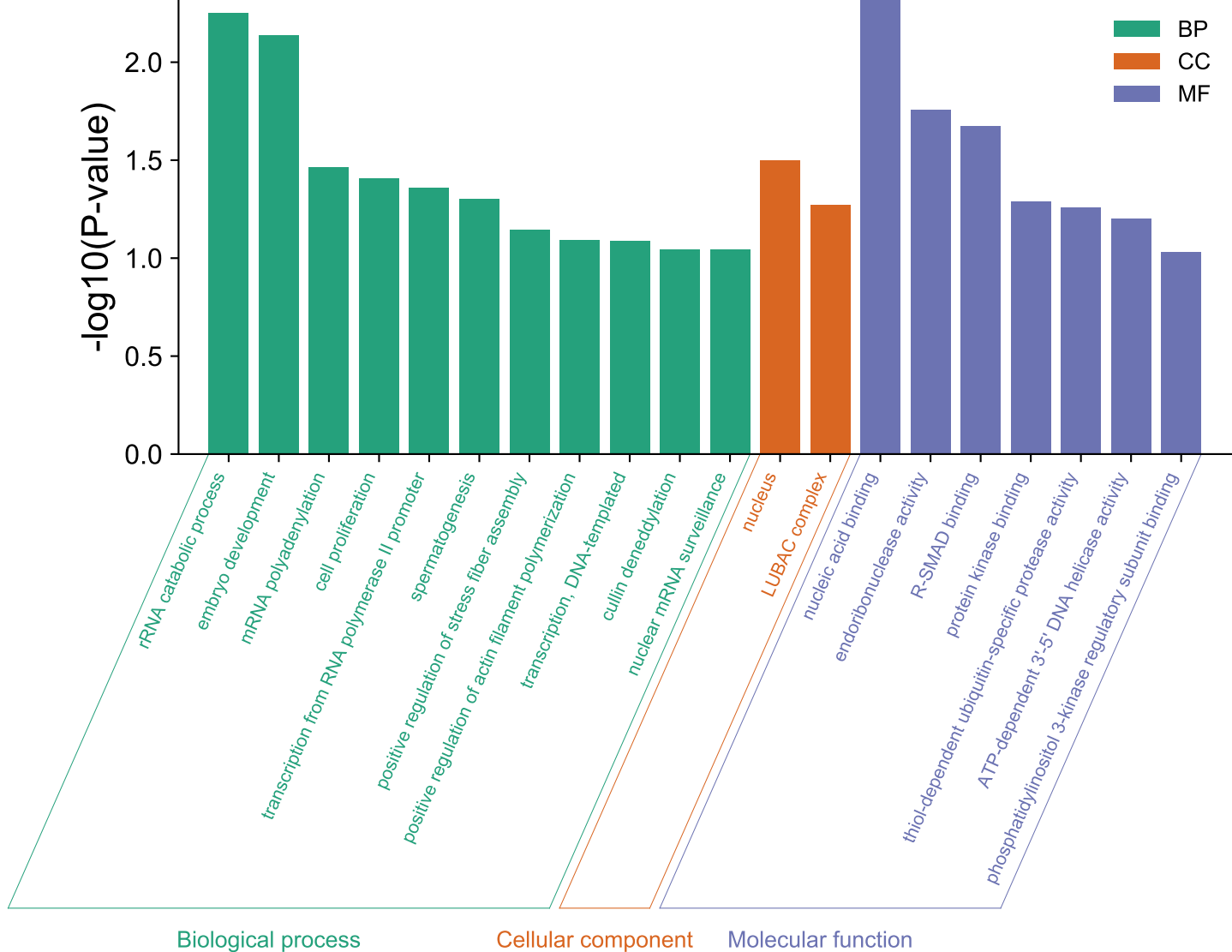

Supplement: Supplementary file 7 [file Data_Sheet_7.ZIP › Supplementary materials fig.6/fig6B.pdf]

# Biological Process\_Enrichment

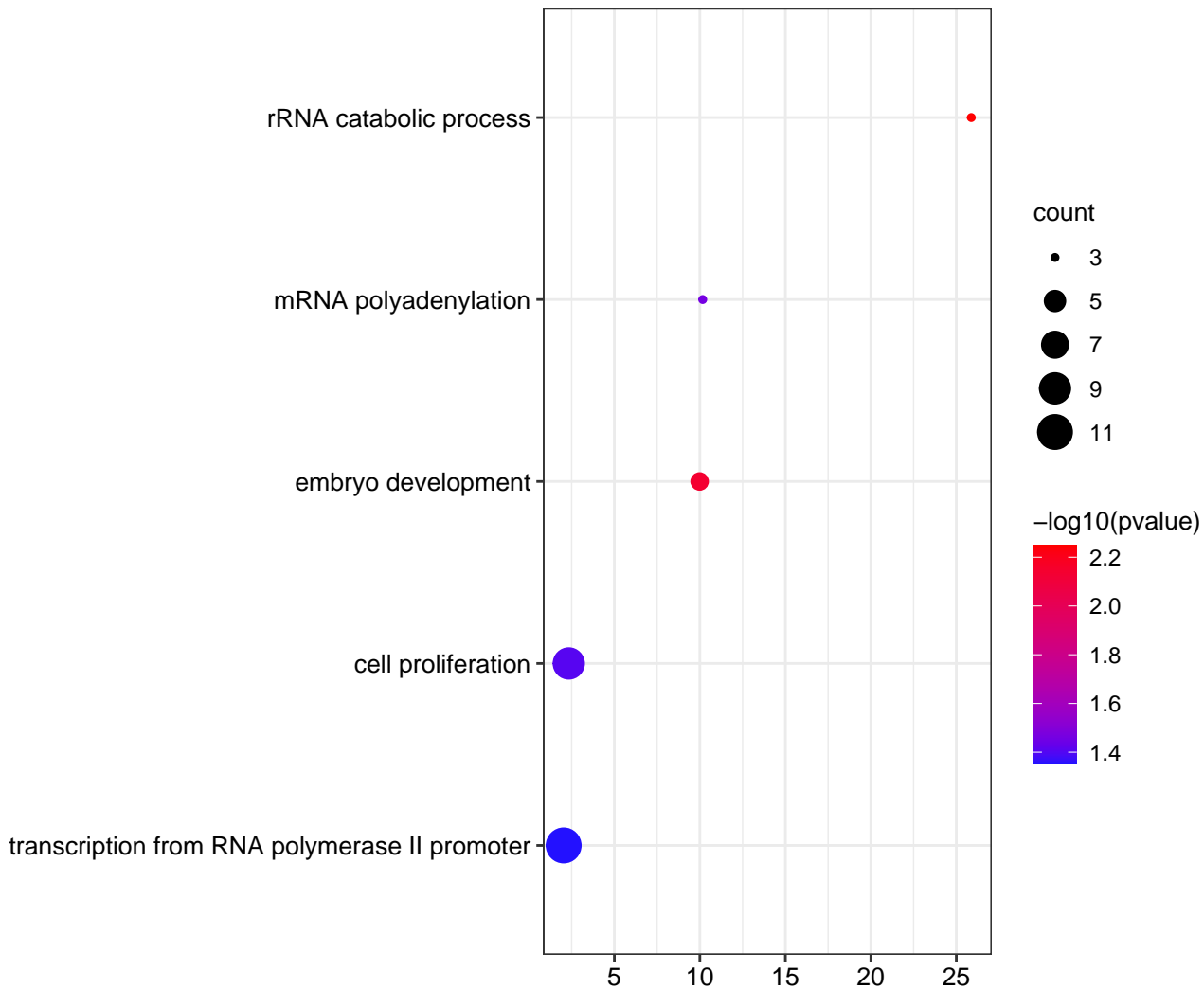

Supplement: Supplementary file 7 [file Data_Sheet_7.ZIP › Supplementary materials fig.6/fig6C.pdf]

# Cellular Component\_Enrichment

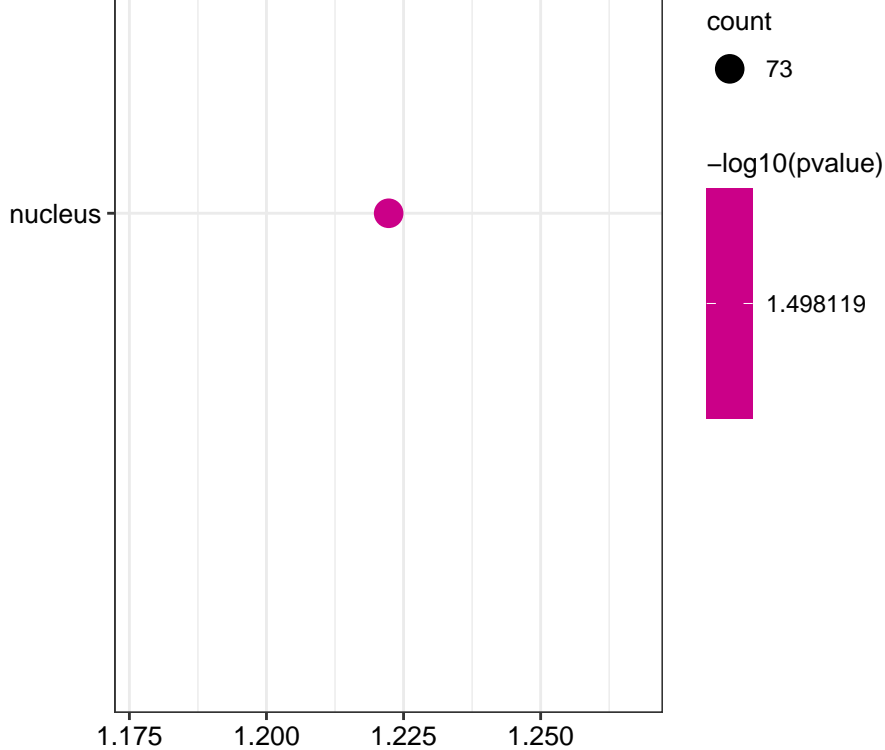

Supplement: Supplementary file 7 [file Data_Sheet_7.ZIP › Supplementary materials fig.6/fig6D.pdf]

# Molecular Function\_Enrichment

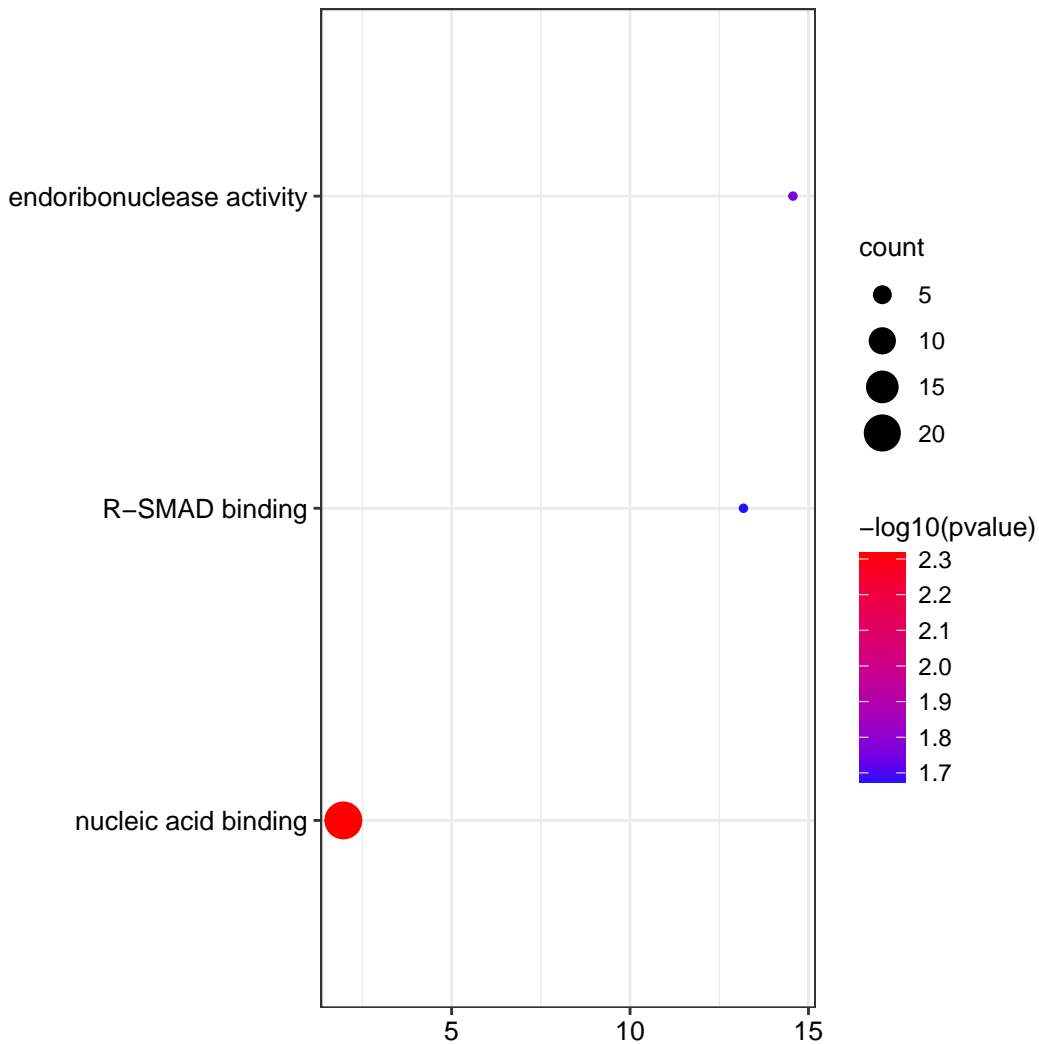

Supplement: Supplementary file 7 [file Data_Sheet_7.ZIP › Supplementary materials fig.6/fig6E.pdf]

# KEGG\_Enrichment

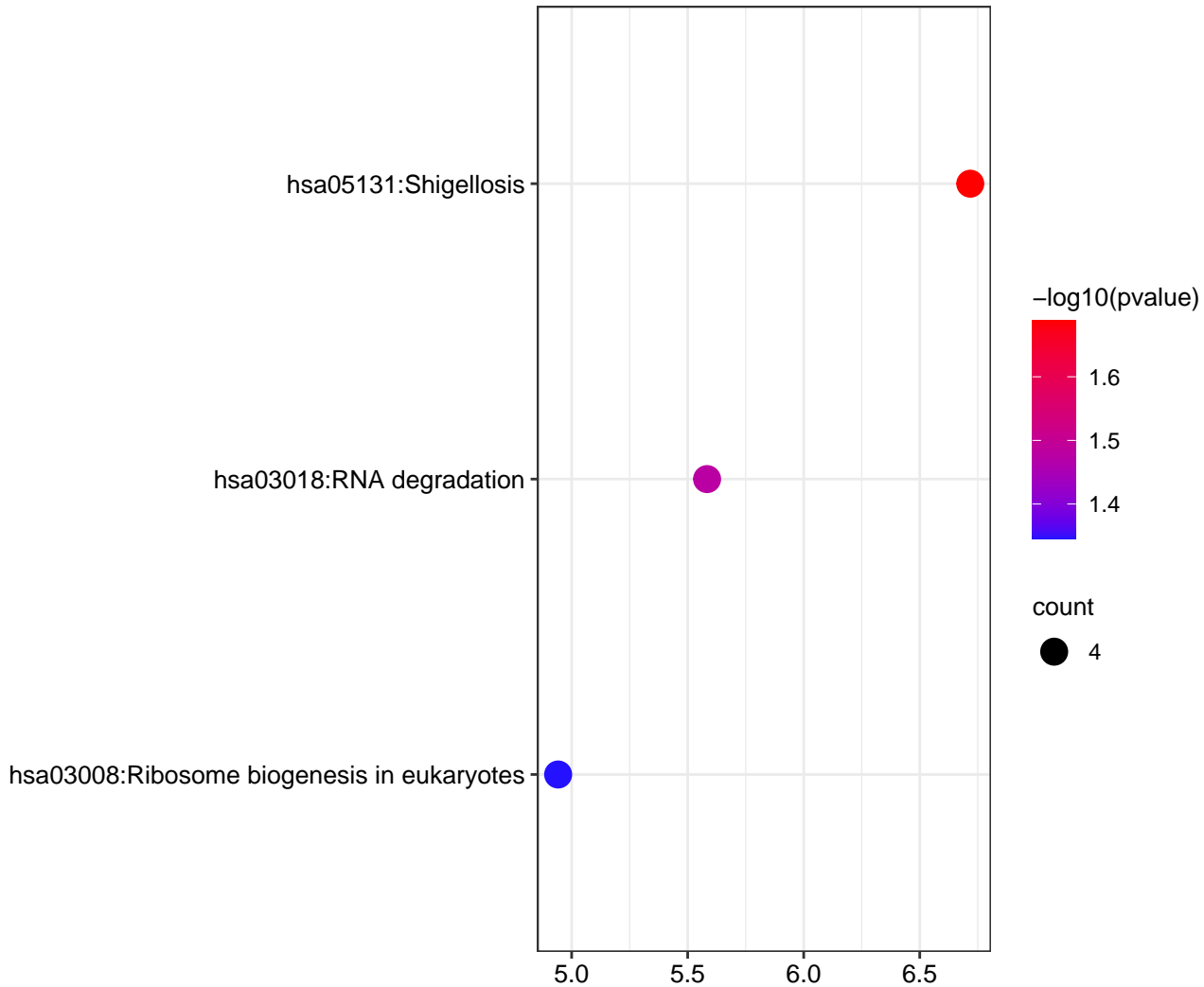

Supplement: Supplementary file 7 [file Data_Sheet_7.ZIP › Supplementary materials fig.6/fig6F.pdf]

# FAM83A (239586\_at)

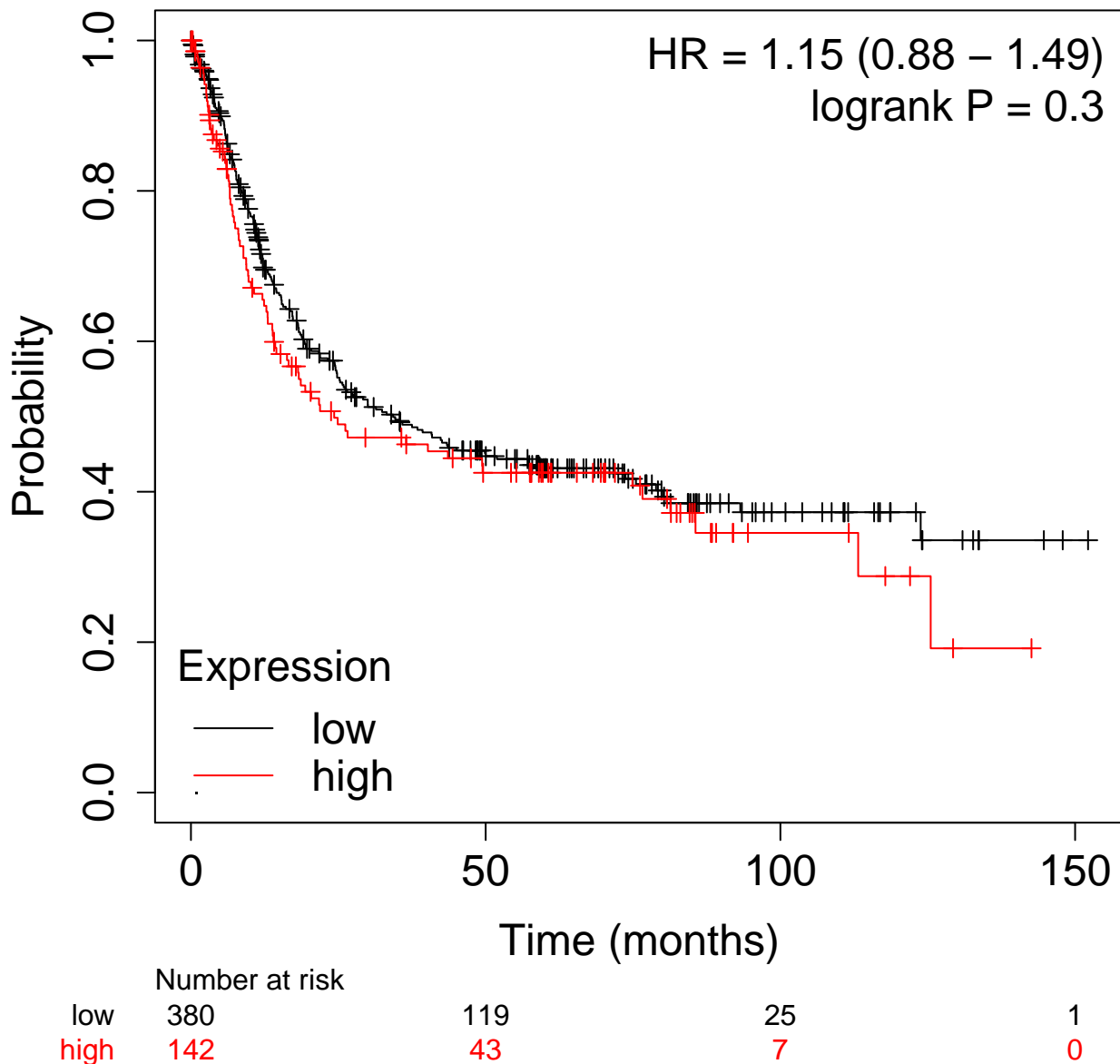

Supplement: Supplementary file 8 [file Data_Sheet_8.ZIP › Supplementary materials fig.7/FP/km_210422_152223_802600_239586_at.pdf]

# FAM83B (232202\_at)

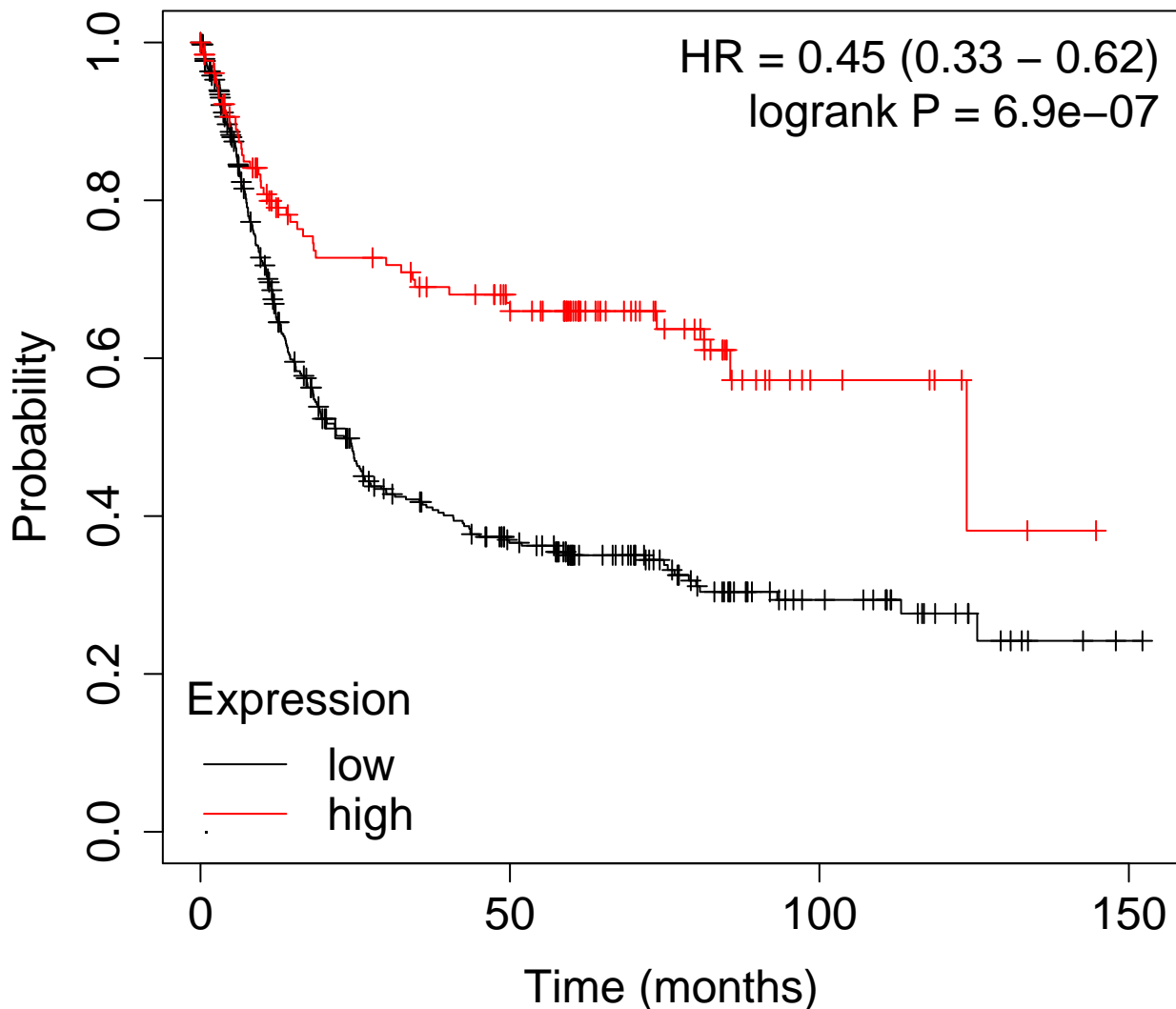

Number at risk

low  
high

391  
131

98  
64

25  
7

1  
0

Supplement: Supplementary file 8 [file Data_Sheet_8.ZIP › Supplementary materials fig.7/FP/km_210422_152226_055600_232202_at.pdf]

# FAM83C (1556793\_a\_at)

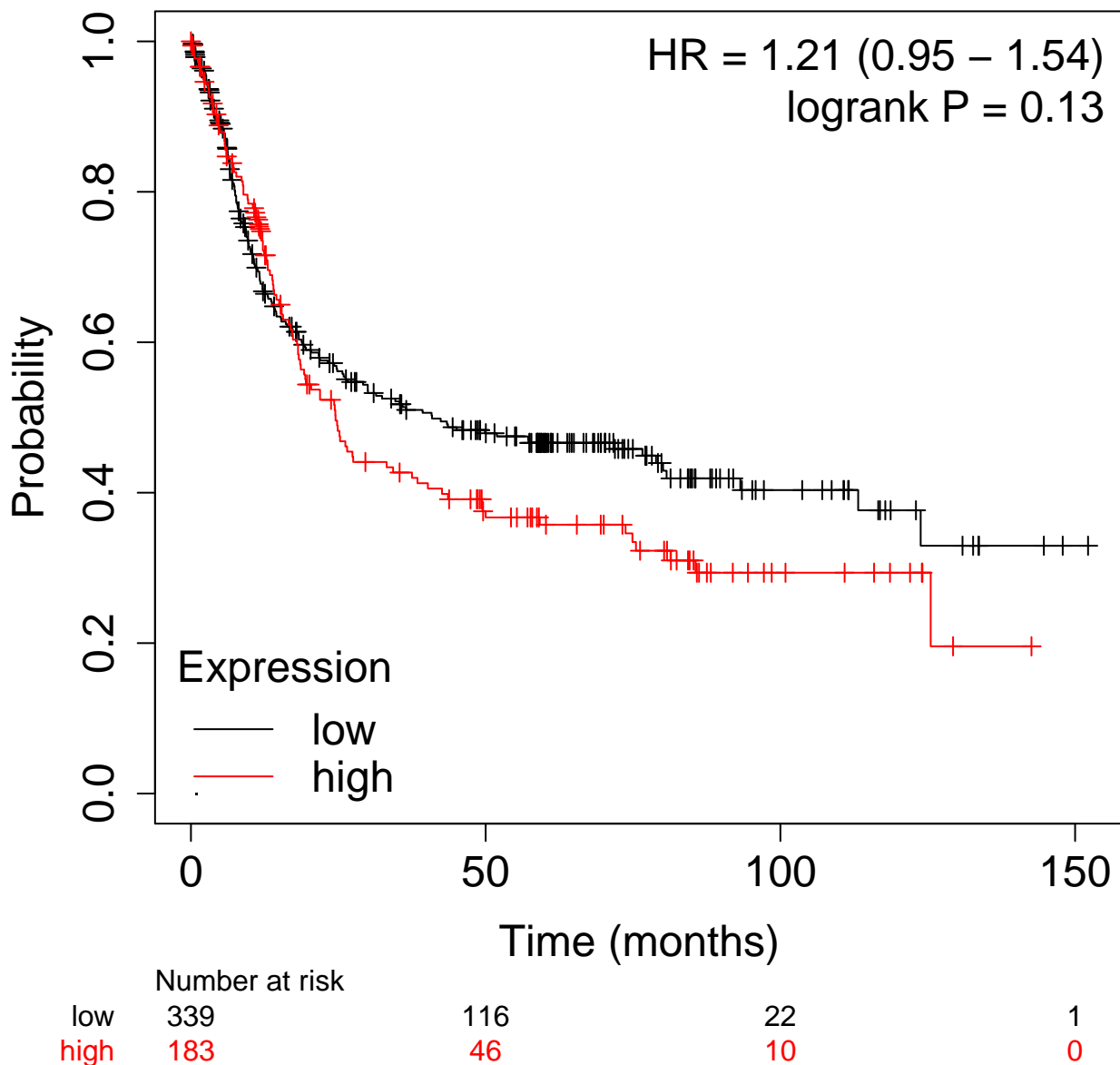

Supplement: Supplementary file 8 [file Data_Sheet_8.ZIP › Supplementary materials fig.7/FP/km_210422_152228_405400_1556793_a_at.pdf]

# FAM83D (225687\_at)

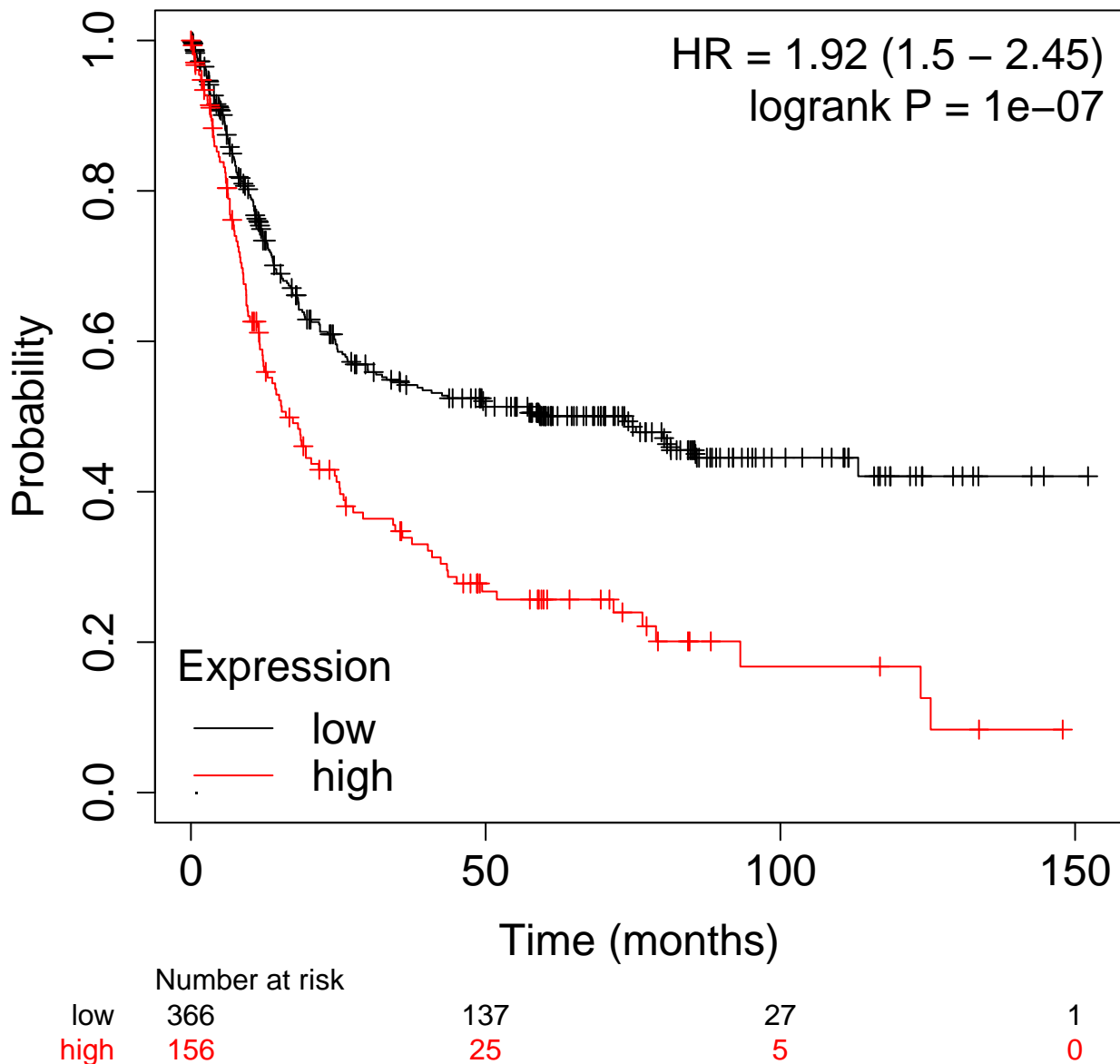

Supplement: Supplementary file 8 [file Data_Sheet_8.ZIP › Supplementary materials fig.7/FP/km_210422_152230_566600_225687_at.pdf]

# FAM83E (220312\_at)

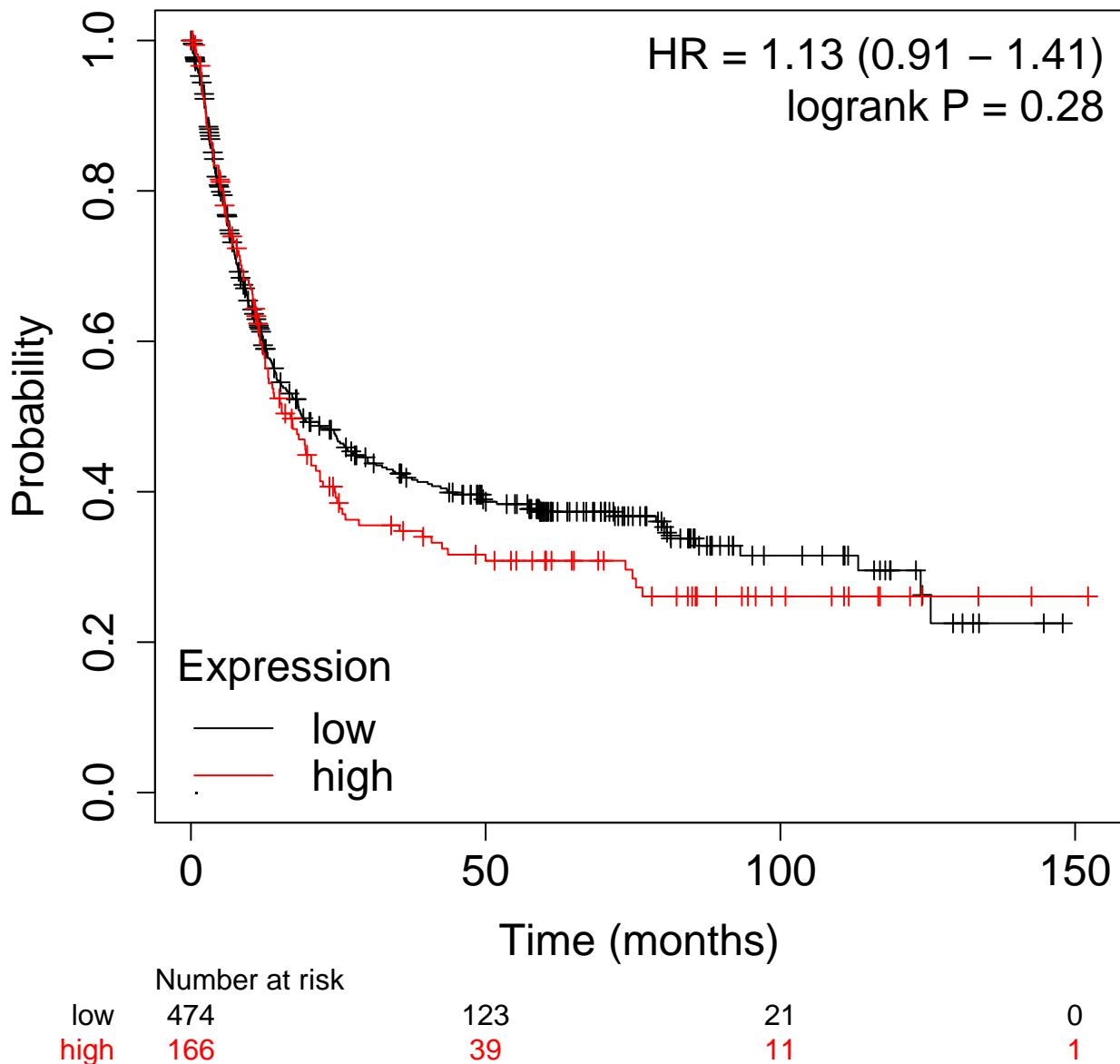

Supplement: Supplementary file 8 [file Data_Sheet_8.ZIP › Supplementary materials fig.7/FP/km_210422_152232_749900_220312_at.pdf]

# FAM83F (235269\_at)

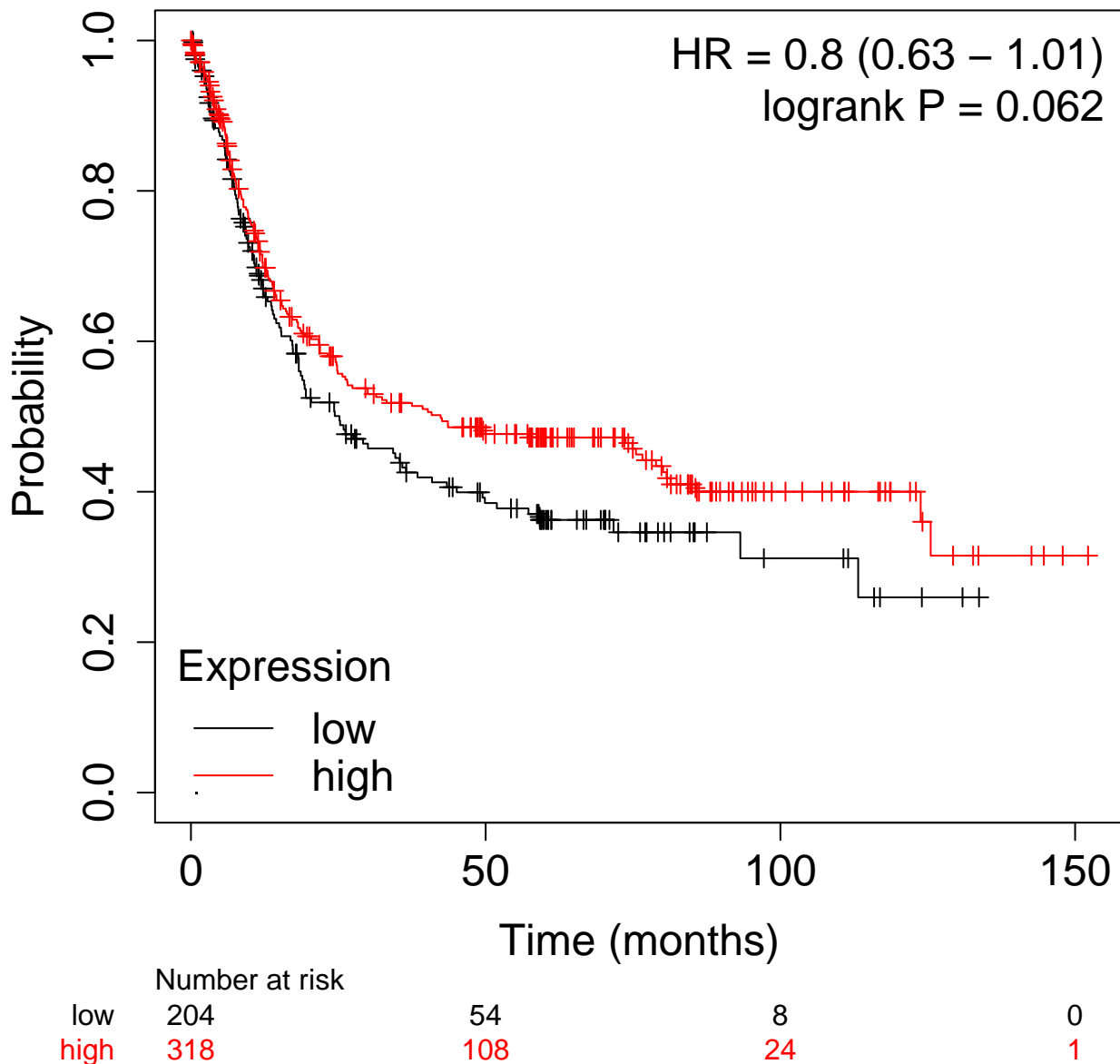

Supplement: Supplementary file 8 [file Data_Sheet_8.ZIP › Supplementary materials fig.7/FP/km_210422_152235_680300_235269_at.pdf]

# FAM83H (226129\_at)

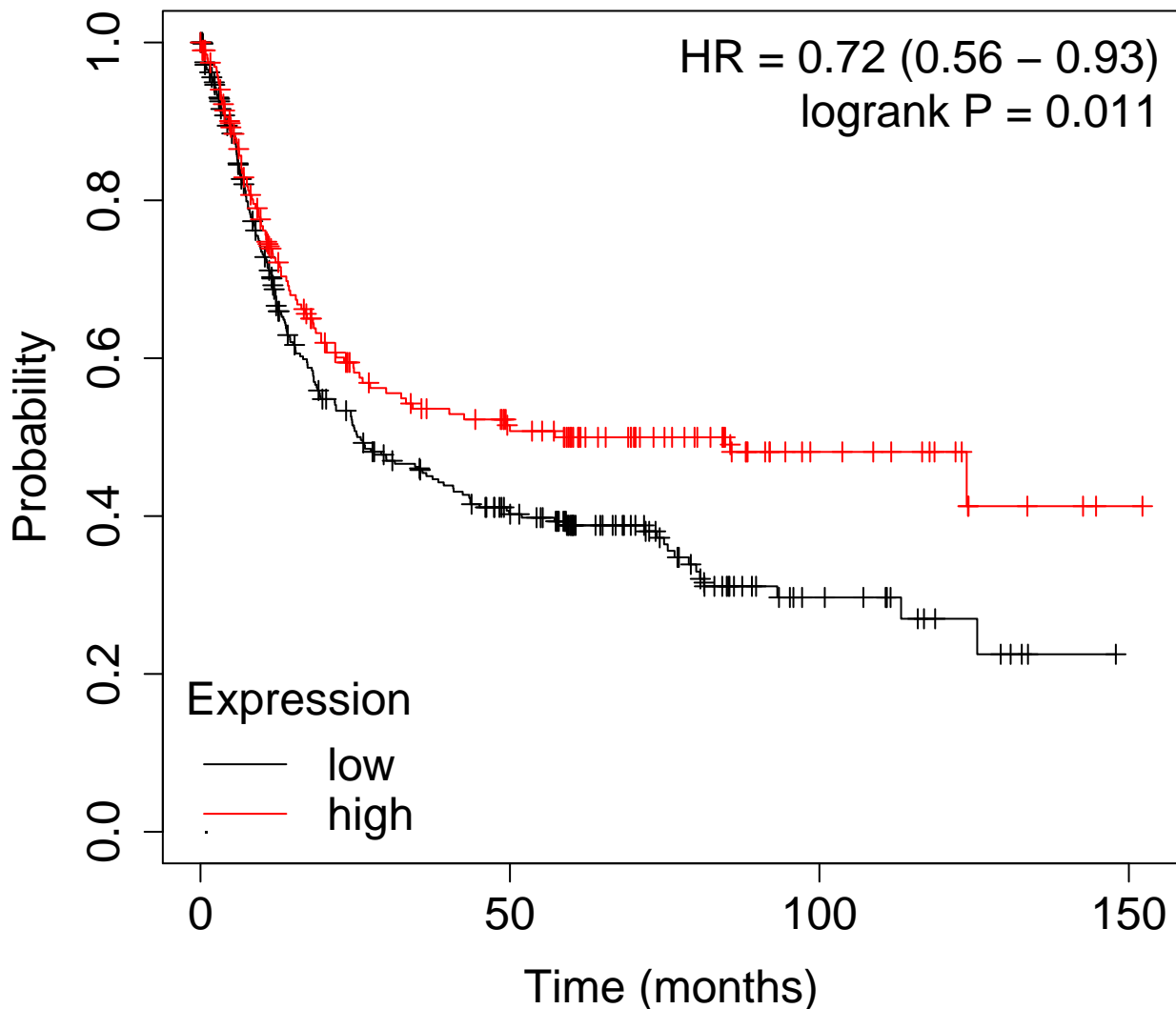

Number at risk

low  
high

322  
200

93  
69

17  
15

0  
1

Supplement: Supplementary file 8 [file Data_Sheet_8.ZIP › Supplementary materials fig.7/FP/km_210422_152238_089600_226129_at.pdf]

# FAM83A (239586\_at)

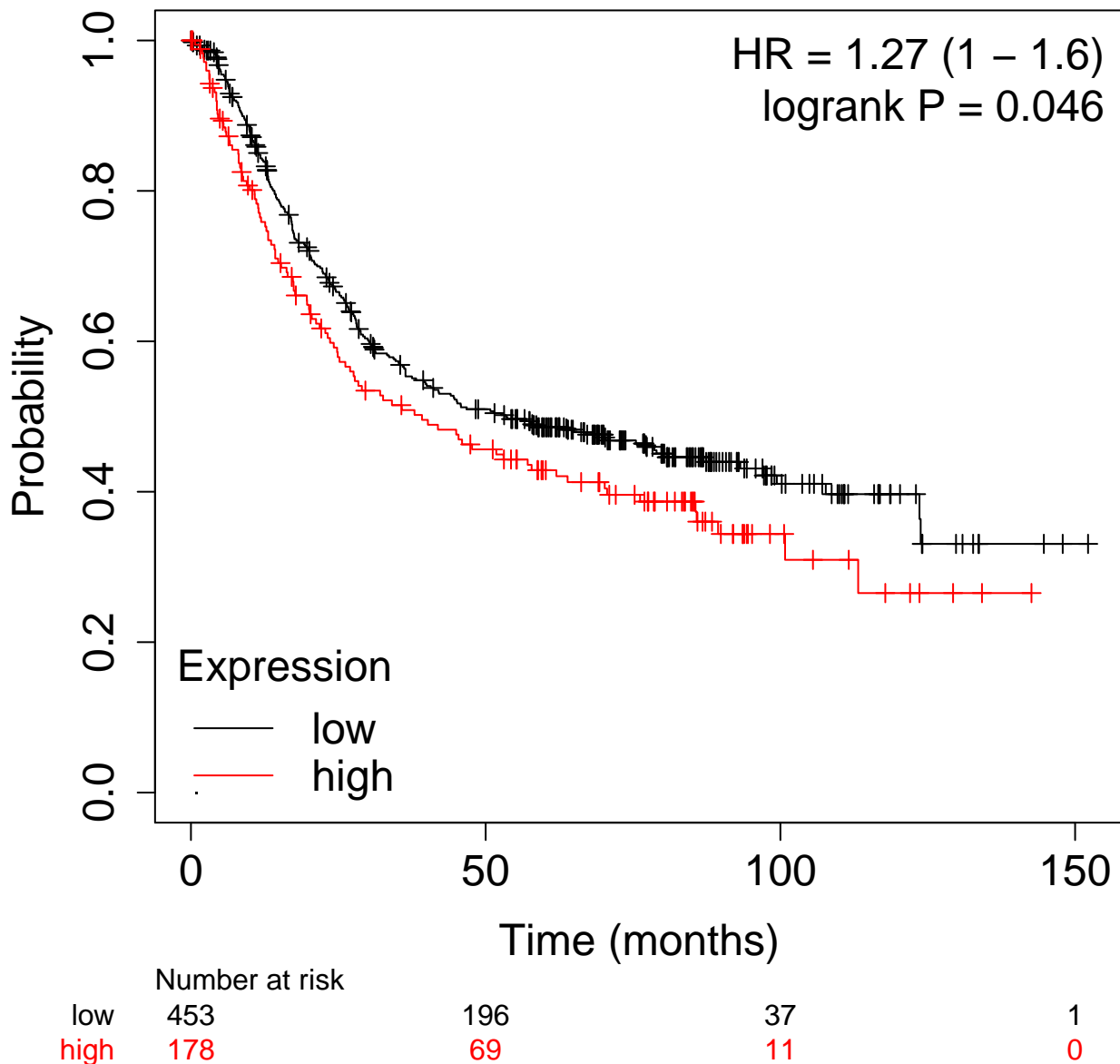

Supplement: Supplementary file 8 [file Data_Sheet_8.ZIP › Supplementary materials fig.7/OS/km_210422_144430_565300_239586_at.pdf]

# FAM83B (232202\_at)

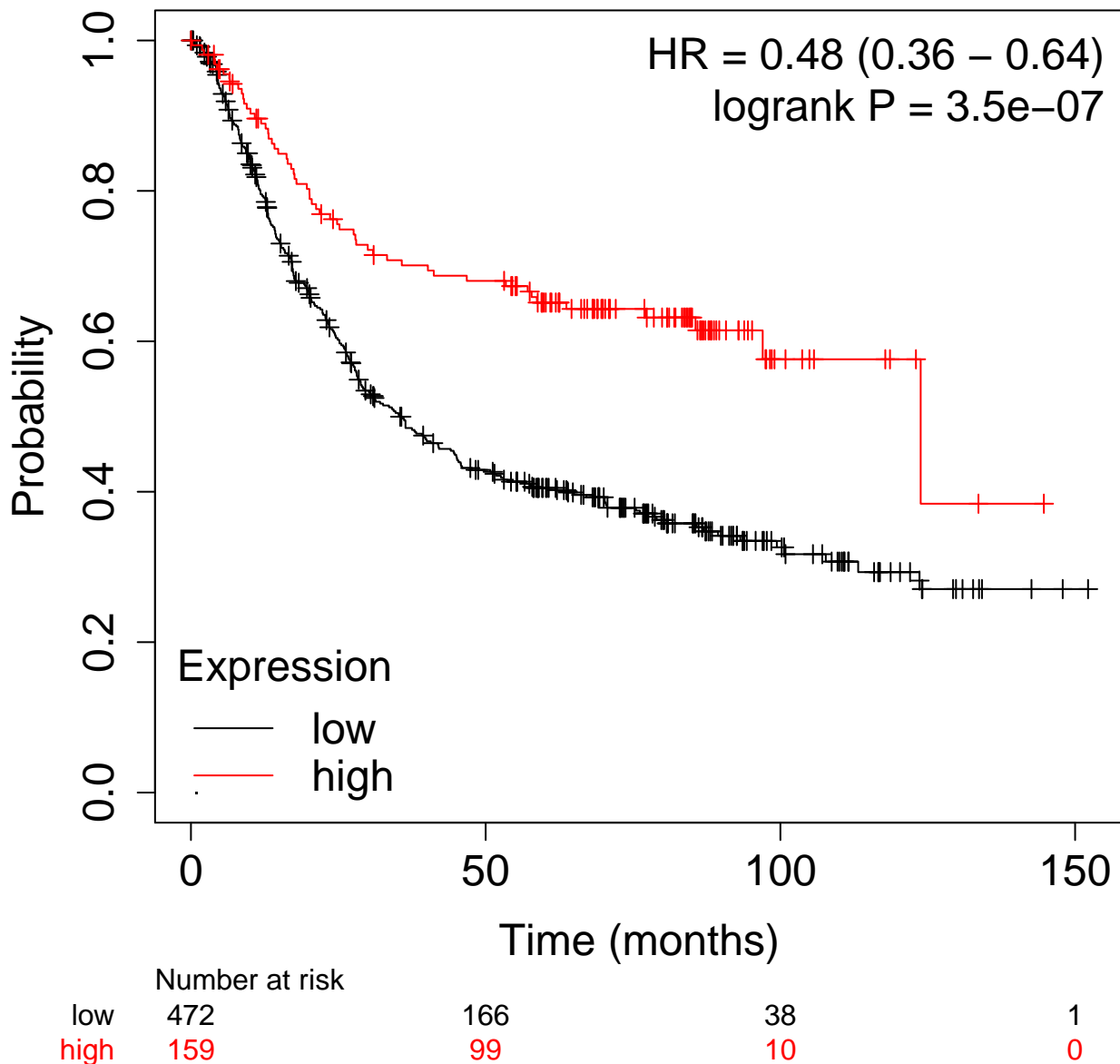

Supplement: Supplementary file 8 [file Data_Sheet_8.ZIP › Supplementary materials fig.7/OS/km_210422_144432_869100_232202_at.pdf]

# FAM83C (1556793\_a\_at)

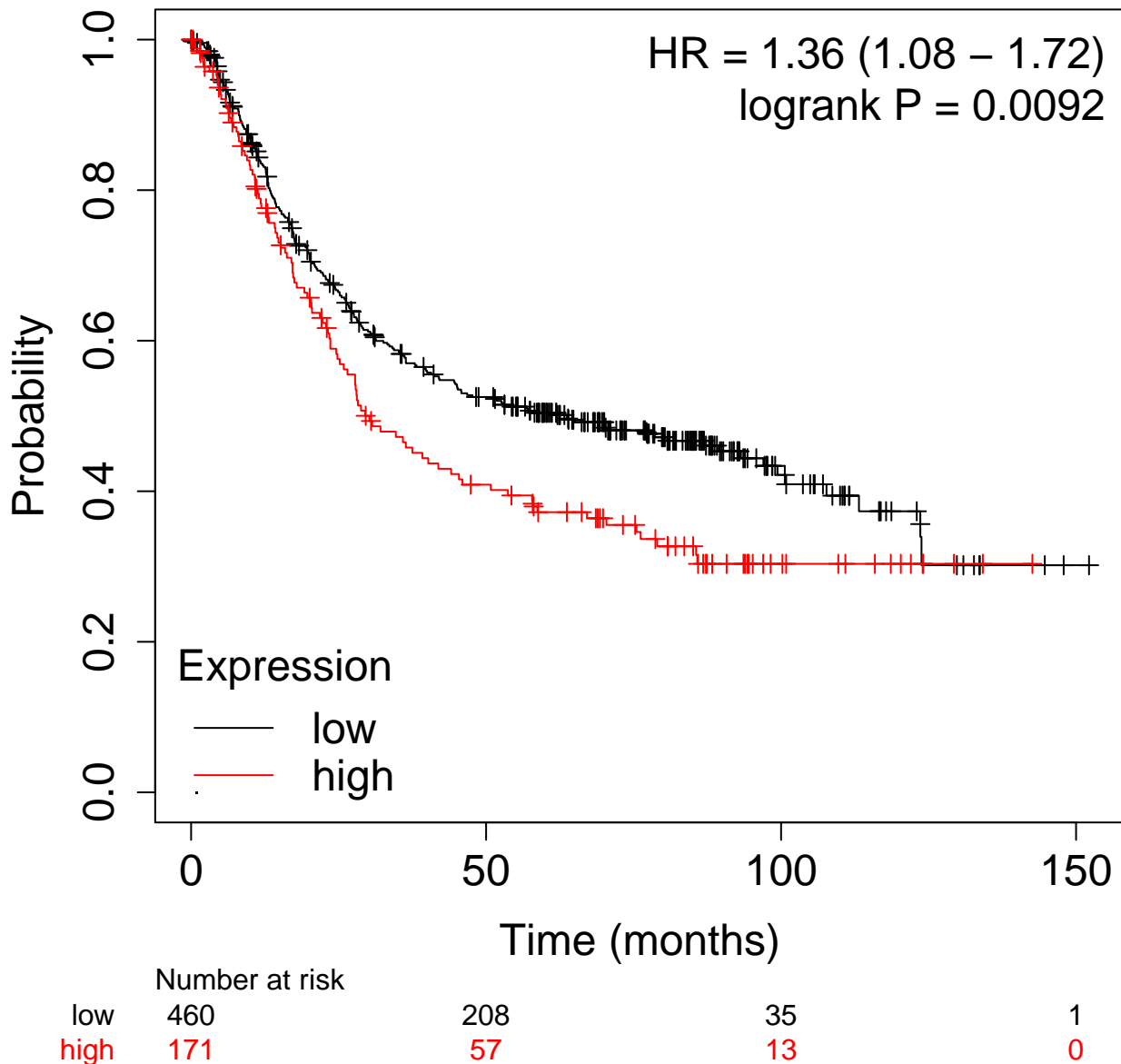

Supplement: Supplementary file 8 [file Data_Sheet_8.ZIP › Supplementary materials fig.7/OS/km_210422_144435_300100_1556793_a_at(1).pdf]

# FAM83D (225687\_at)

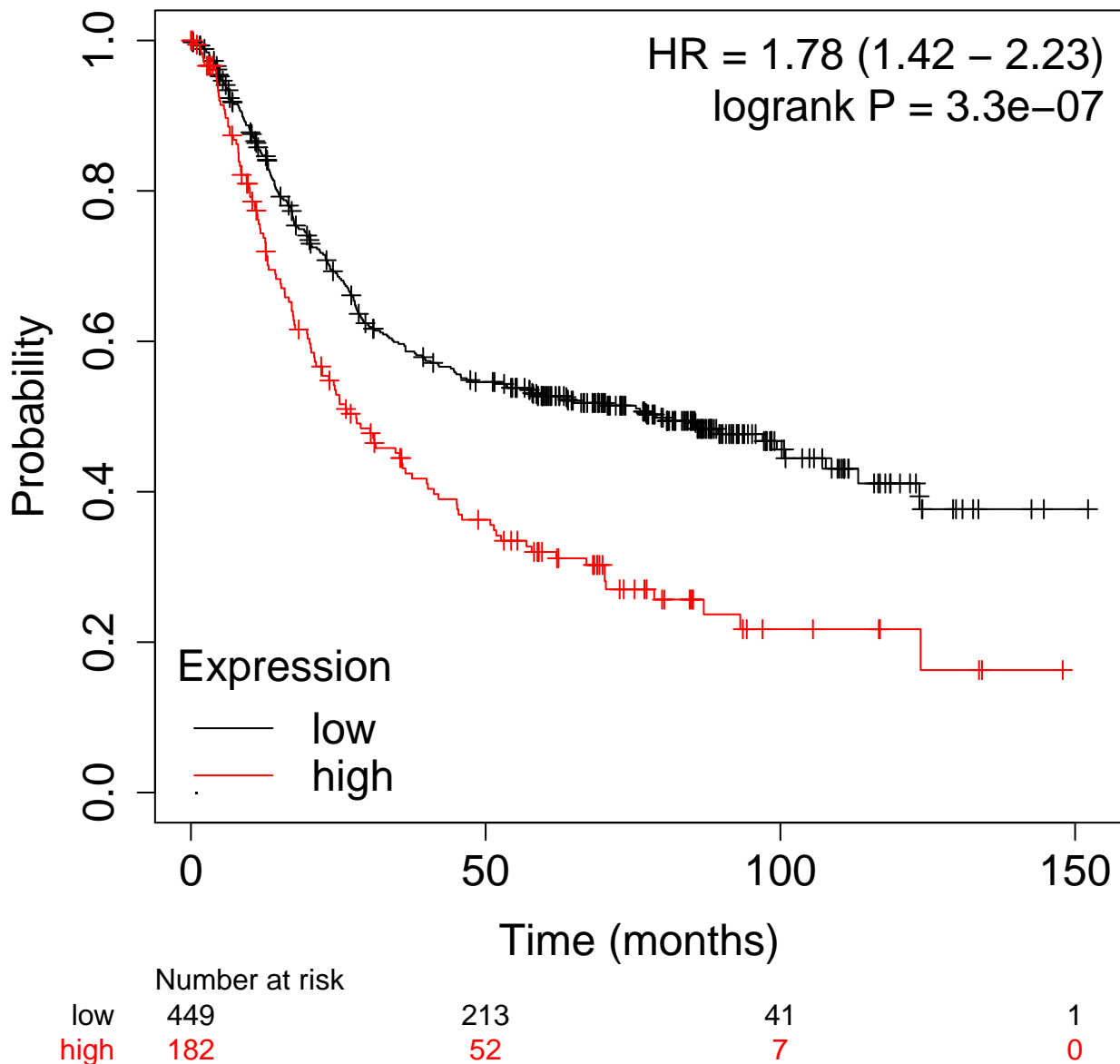

Supplement: Supplementary file 8 [file Data_Sheet_8.ZIP › Supplementary materials fig.7/OS/km_210422_144437_614300_225687_at.pdf]

# FAM83E (220312\_at)

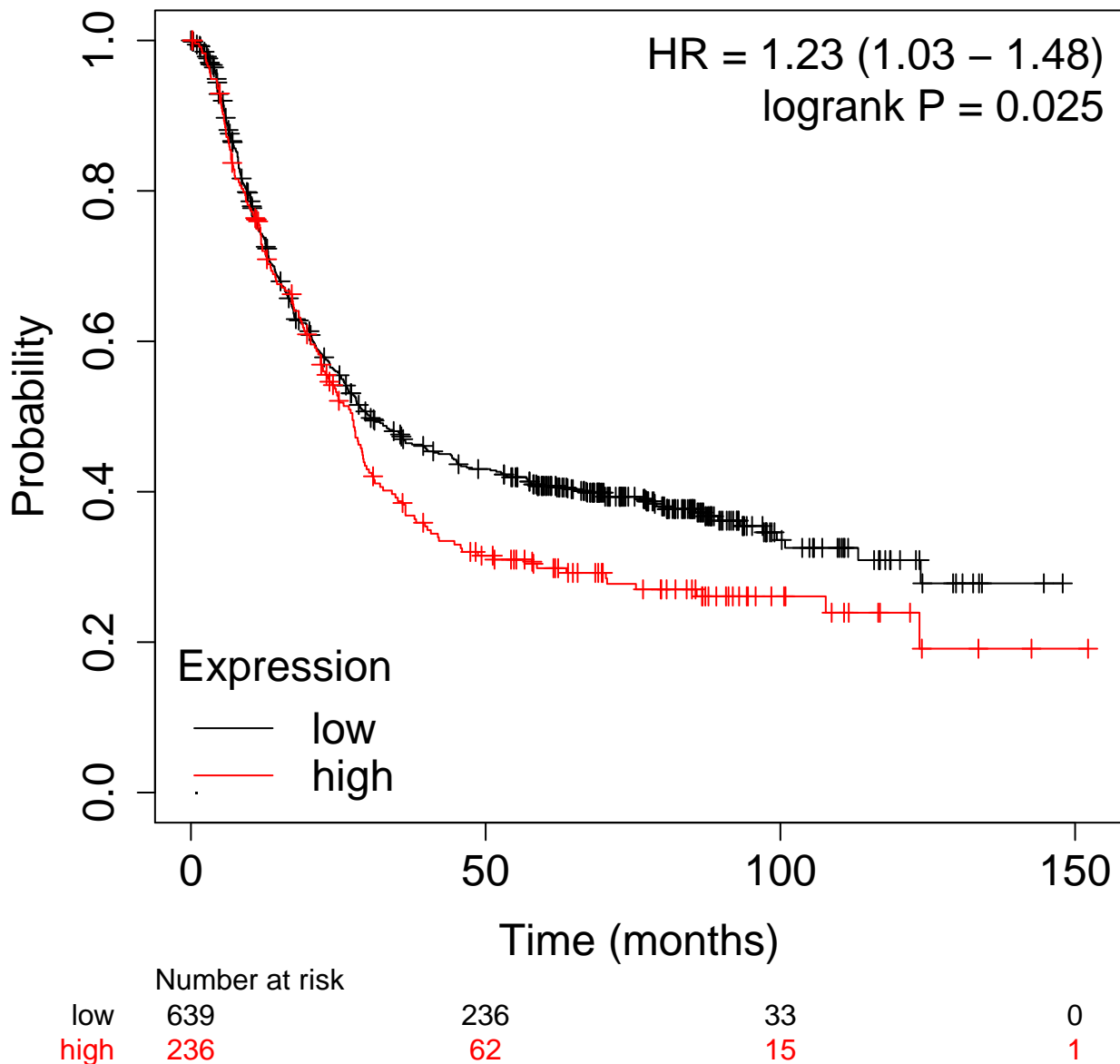

Supplement: Supplementary file 8 [file Data_Sheet_8.ZIP › Supplementary materials fig.7/OS/km_210422_144439_938500_220312_at.pdf]

# FAM83F (235269\_at)

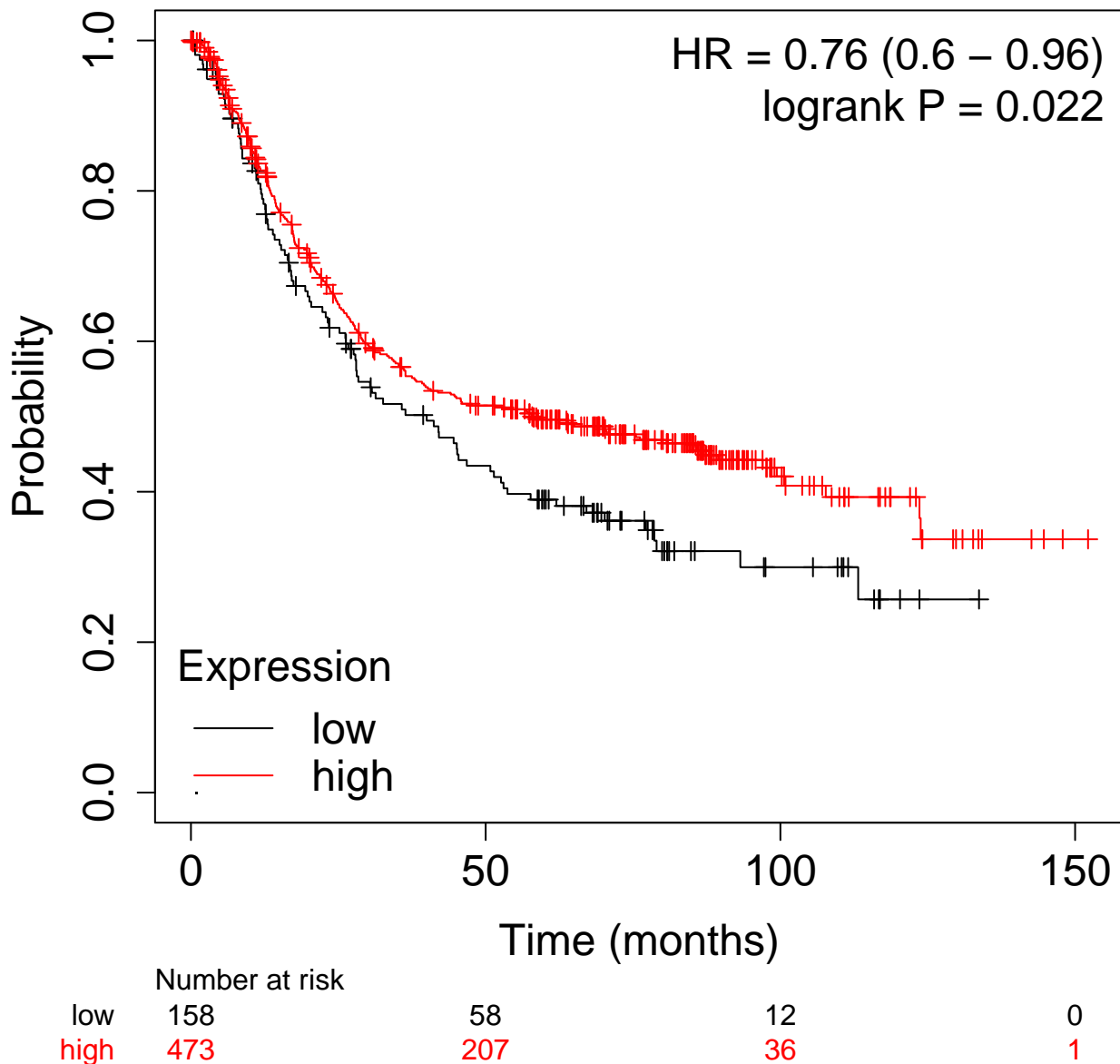

Supplement: Supplementary file 8 [file Data_Sheet_8.ZIP › Supplementary materials fig.7/OS/km_210422_144442_842500_235269_at.pdf]

# FAM83H (226129\_at)

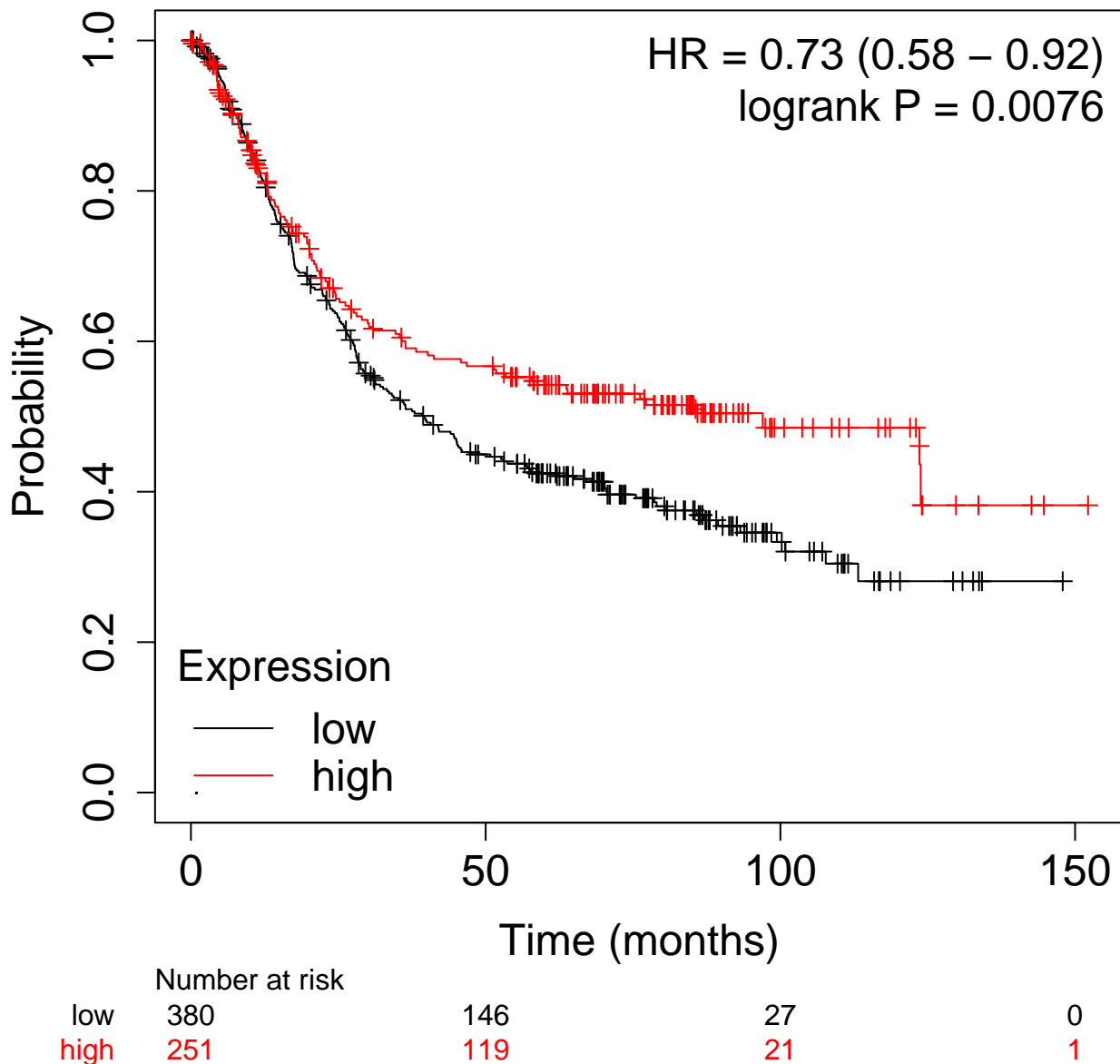

Supplement: Supplementary file 8 [file Data_Sheet_8.ZIP › Supplementary materials fig.7/OS/km_210422_144445_781900_226129_at(1).pdf]

# FAM83A (239586\_at)

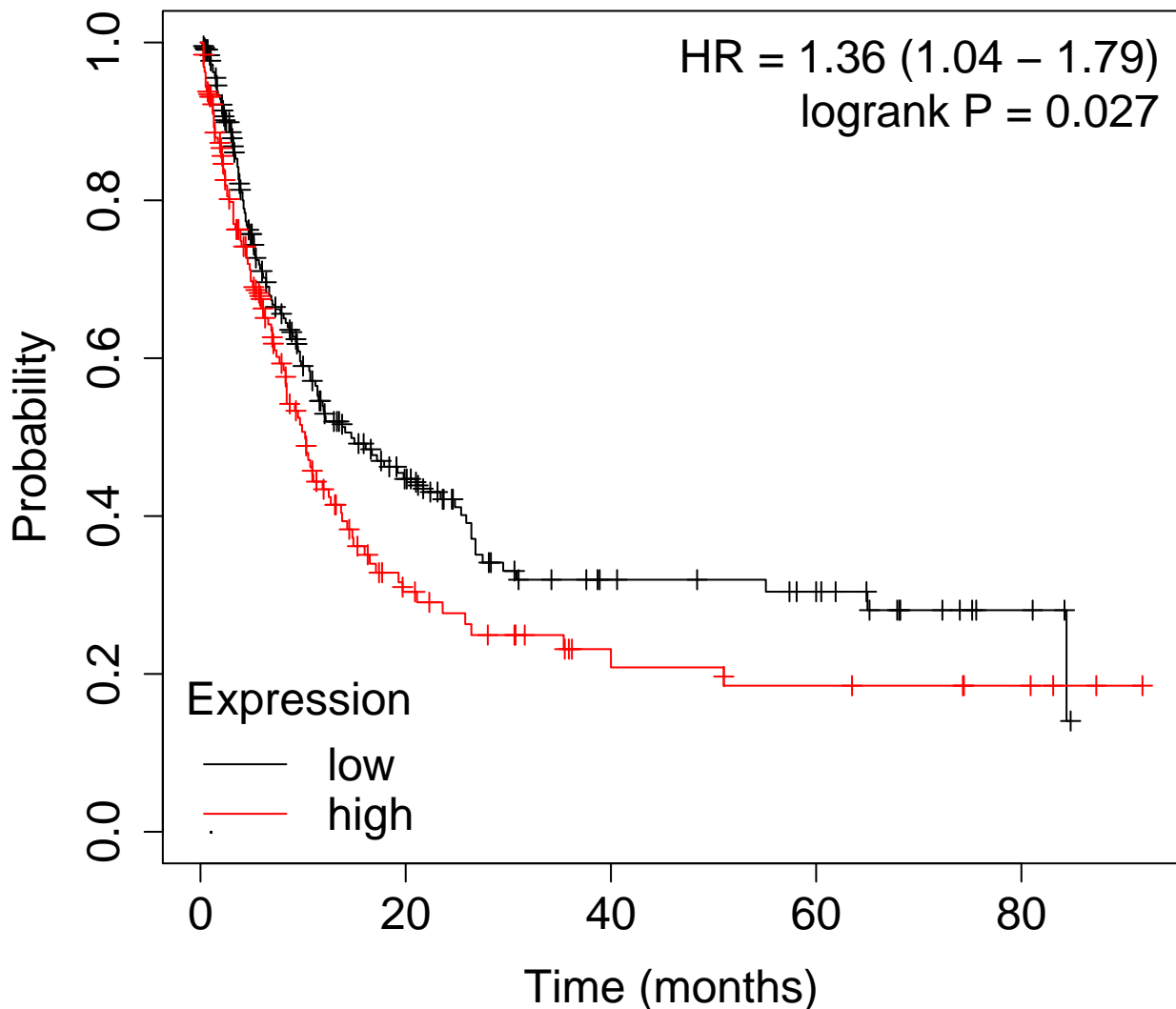

Number at risk

|      |     |    |    |    |   |
|------|-----|----|----|----|---|
| low  | 223 | 57 | 24 | 17 | 4 |
| high | 161 | 24 | 10 | 7  | 4 |

Supplement: Supplementary file 8 [file Data_Sheet_8.ZIP › Supplementary materials fig.7/PPS/km_210422_163807_740900_239586_at.pdf]

# FAM83B (232202\_at)

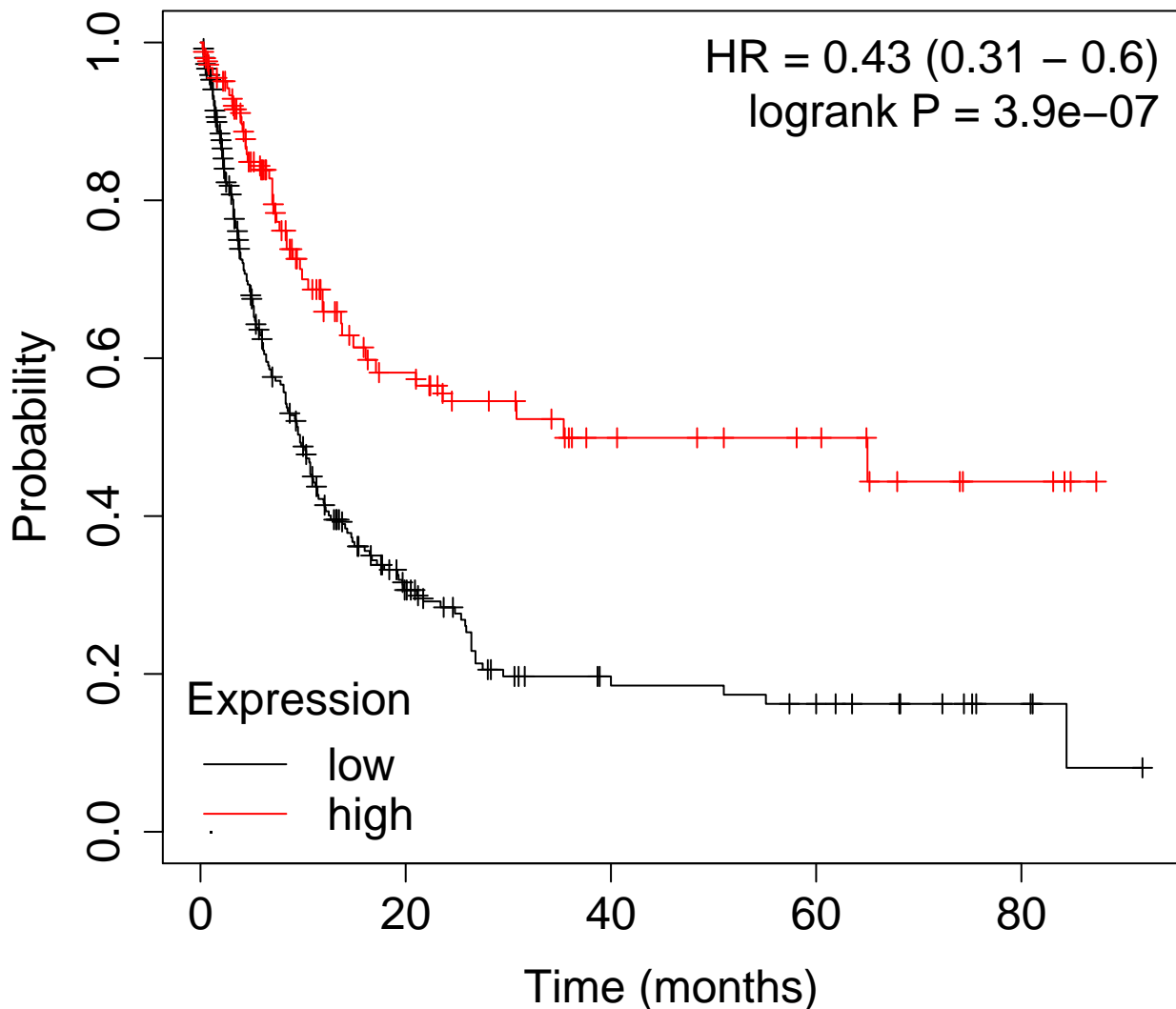

Number at risk

|      |     |    |    |    |   |
|------|-----|----|----|----|---|
| low  | 258 | 46 | 17 | 13 | 4 |
| high | 126 | 35 | 17 | 11 | 4 |

Supplement: Supplementary file 8 [file Data_Sheet_8.ZIP › Supplementary materials fig.7/PPS/km_210422_163809_732500_232202_at.pdf]

# FAM83C (1556793\_a\_at)

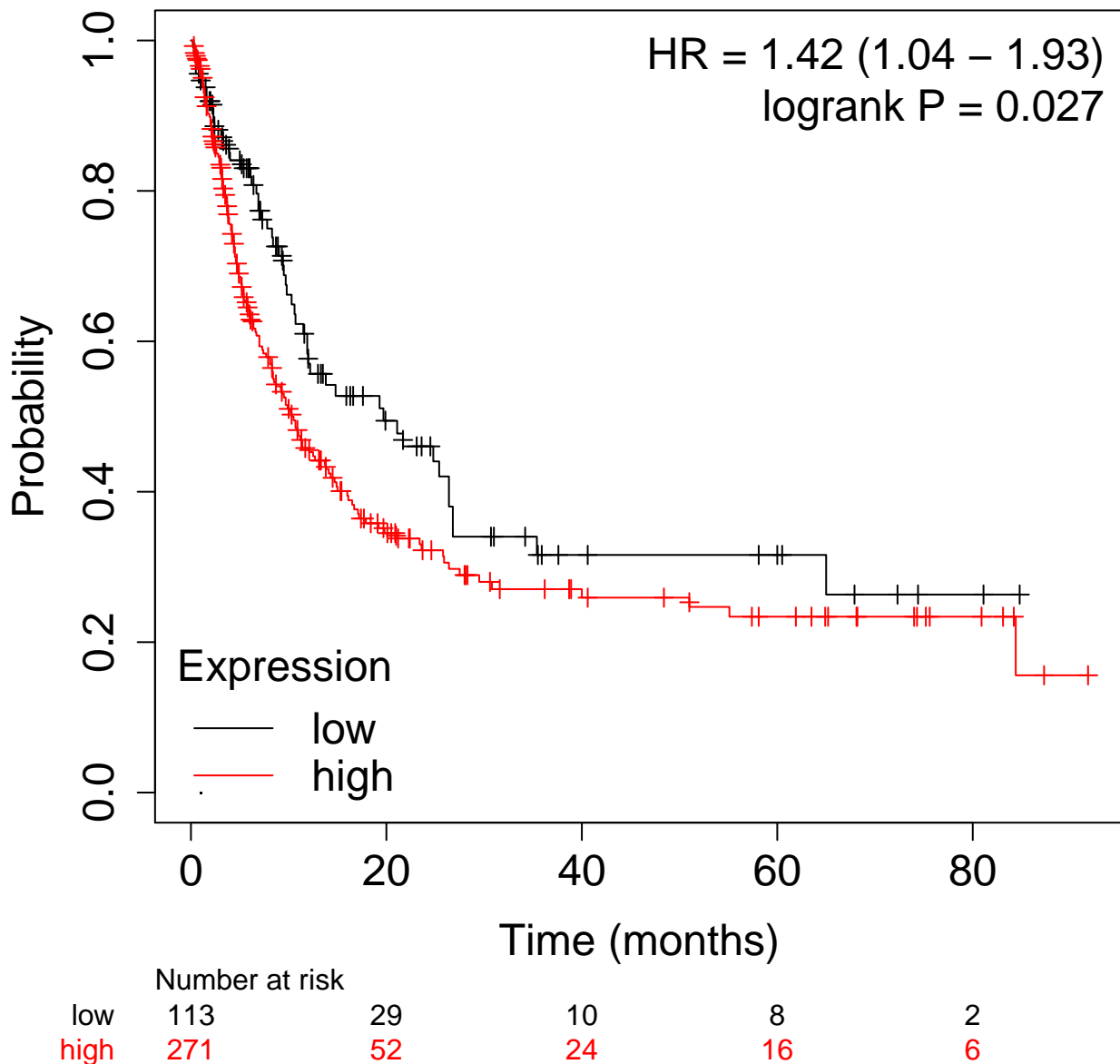

Supplement: Supplementary file 8 [file Data_Sheet_8.ZIP › Supplementary materials fig.7/PPS/km_210422_163811_843100_1556793_a_at.pdf]

# FAM83D (225687\_at)

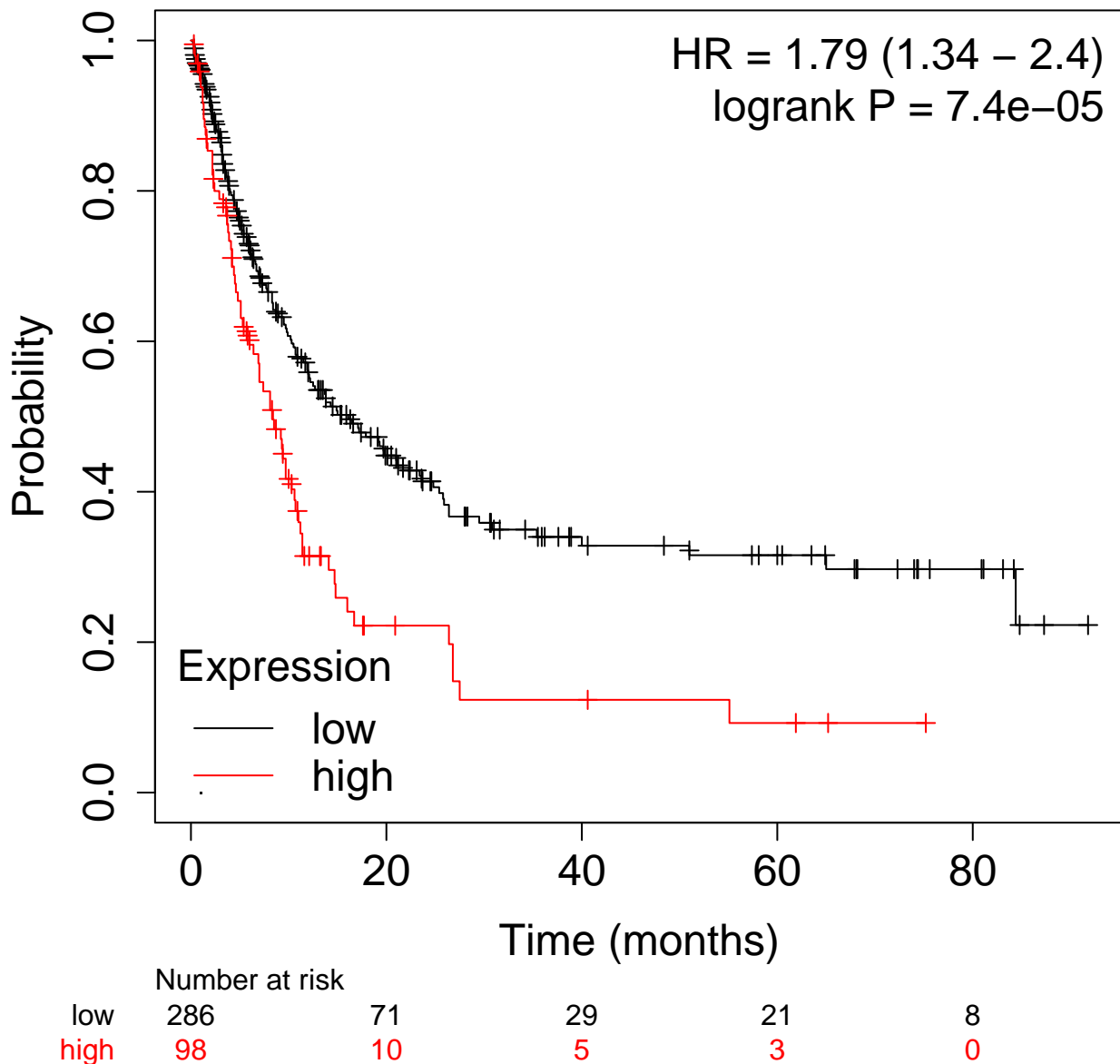

Supplement: Supplementary file 8 [file Data_Sheet_8.ZIP › Supplementary materials fig.7/PPS/km_210422_163813_865500_225687_at.pdf]

# FAM83E (220312\_at)

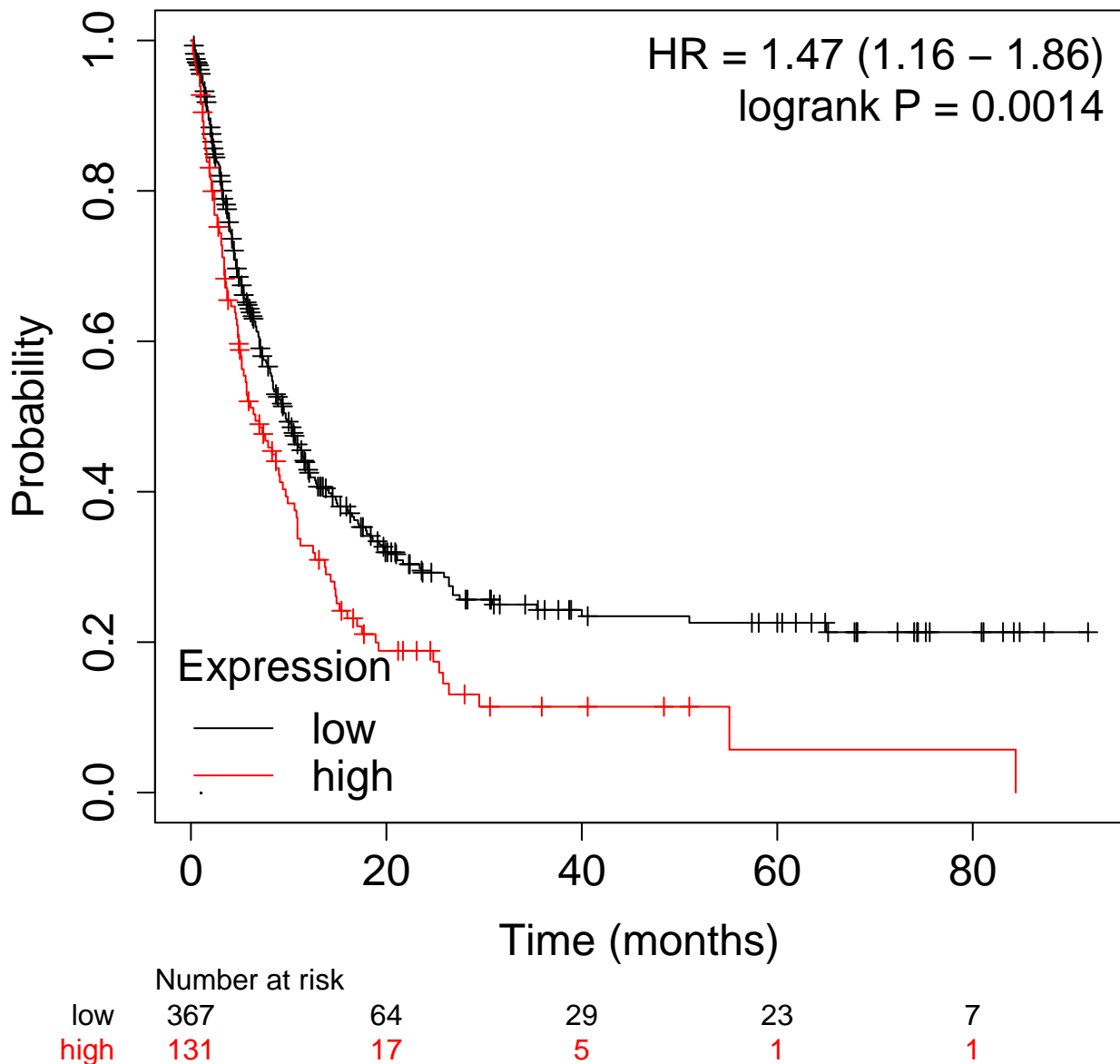

Supplement: Supplementary file 8 [file Data_Sheet_8.ZIP › Supplementary materials fig.7/PPS/km_210422_163815_962800_220312_at.pdf]

# FAM83F (235269\_at)

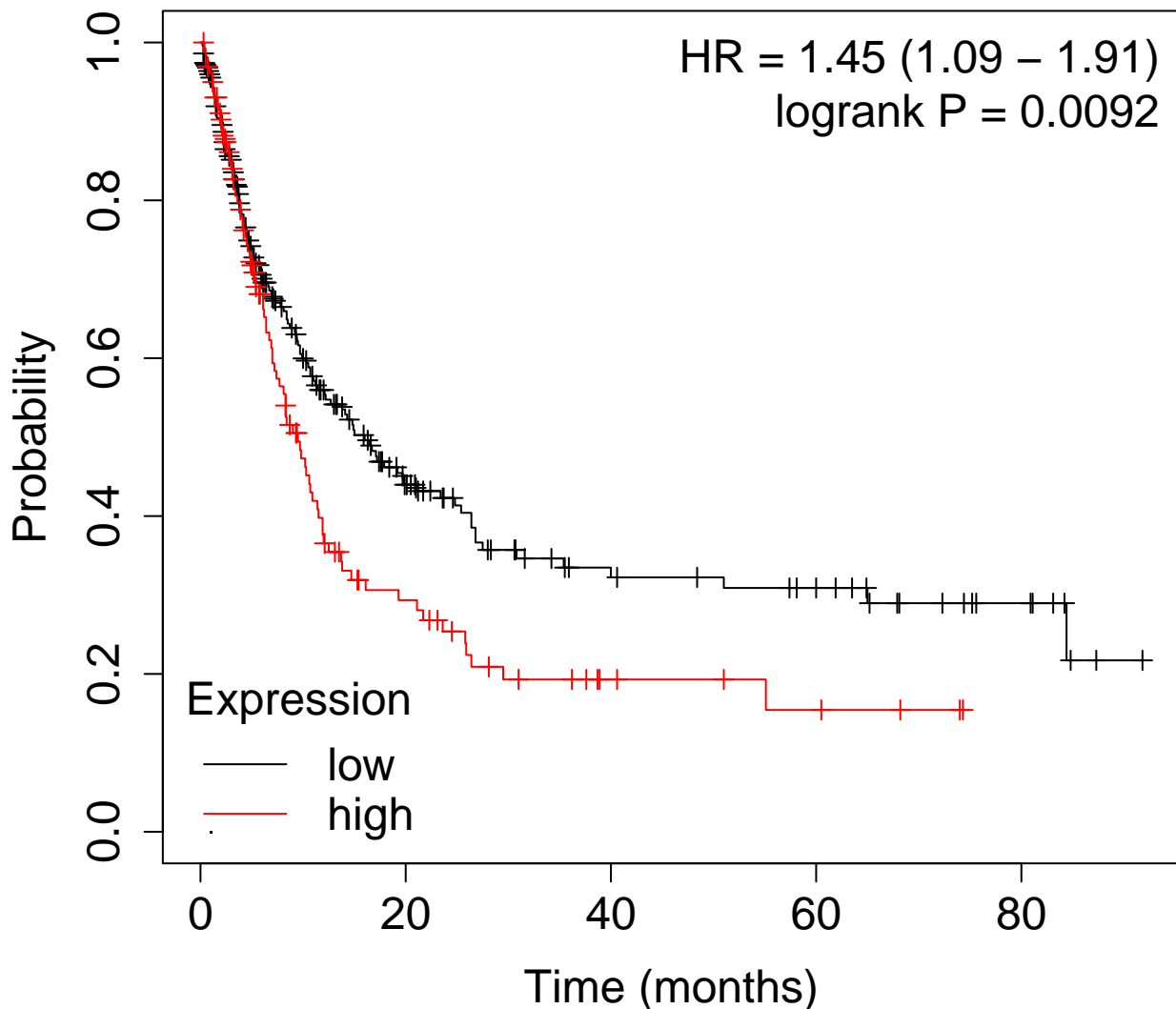

Number at risk

|      |     |    |    |    |   |
|------|-----|----|----|----|---|
| low  | 251 | 58 | 27 | 20 | 8 |
| high | 133 | 23 | 7  | 4  | 0 |

Supplement: Supplementary file 8 [file Data_Sheet_8.ZIP › Supplementary materials fig.7/PPS/km_210422_163817_996600_235269_at.pdf]

# FAM83H (226129\_at)

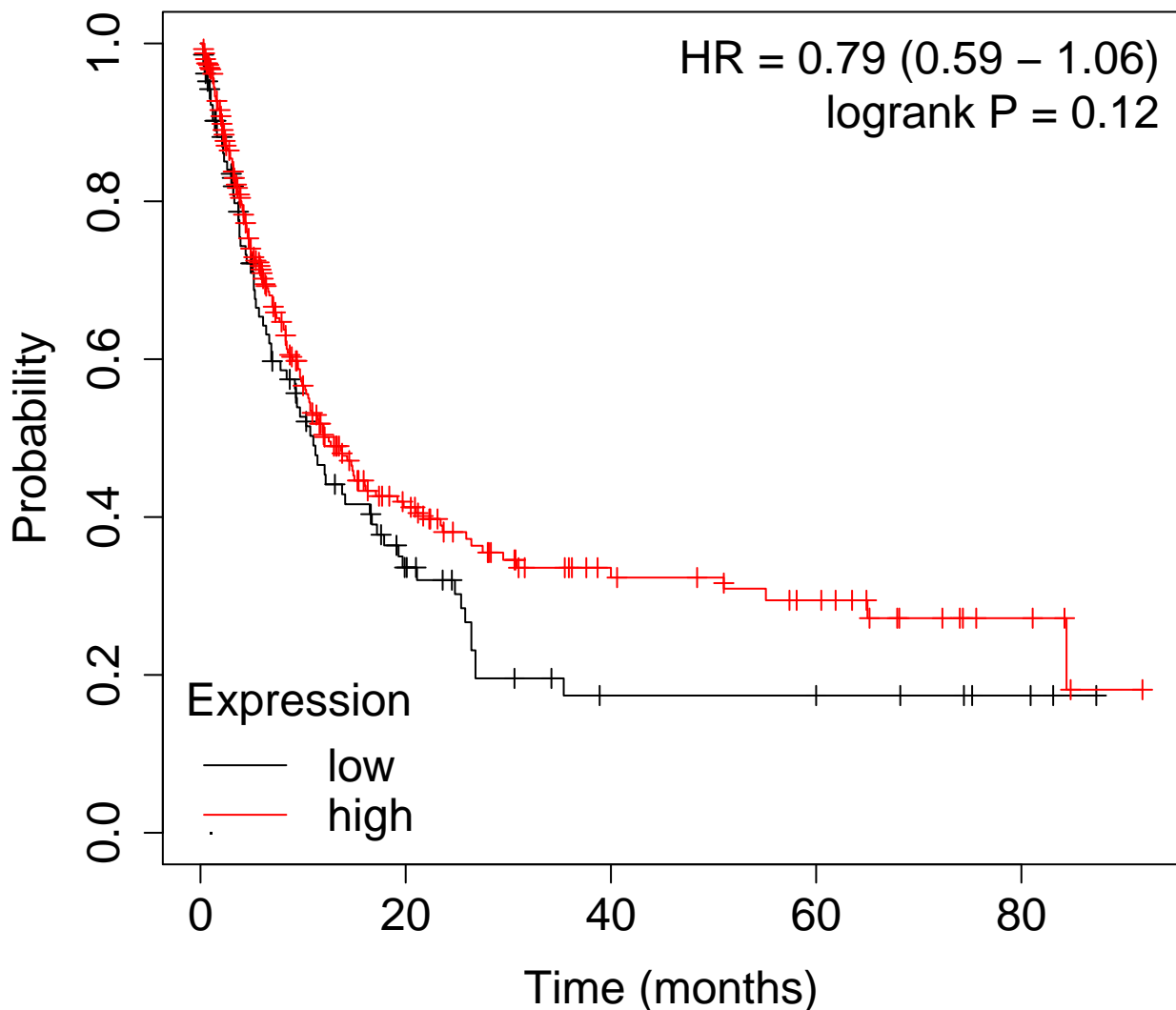

Number at risk

|      |     |    |    |    |   |
|------|-----|----|----|----|---|
| low  | 105 | 23 | 7  | 7  | 3 |
| high | 279 | 58 | 27 | 17 | 5 |

Supplement: Supplementary file 8 [file Data_Sheet_8.ZIP › Supplementary materials fig.7/PPS/km_210422_163820_045100_226129_at.pdf]

# Disease Free Survival

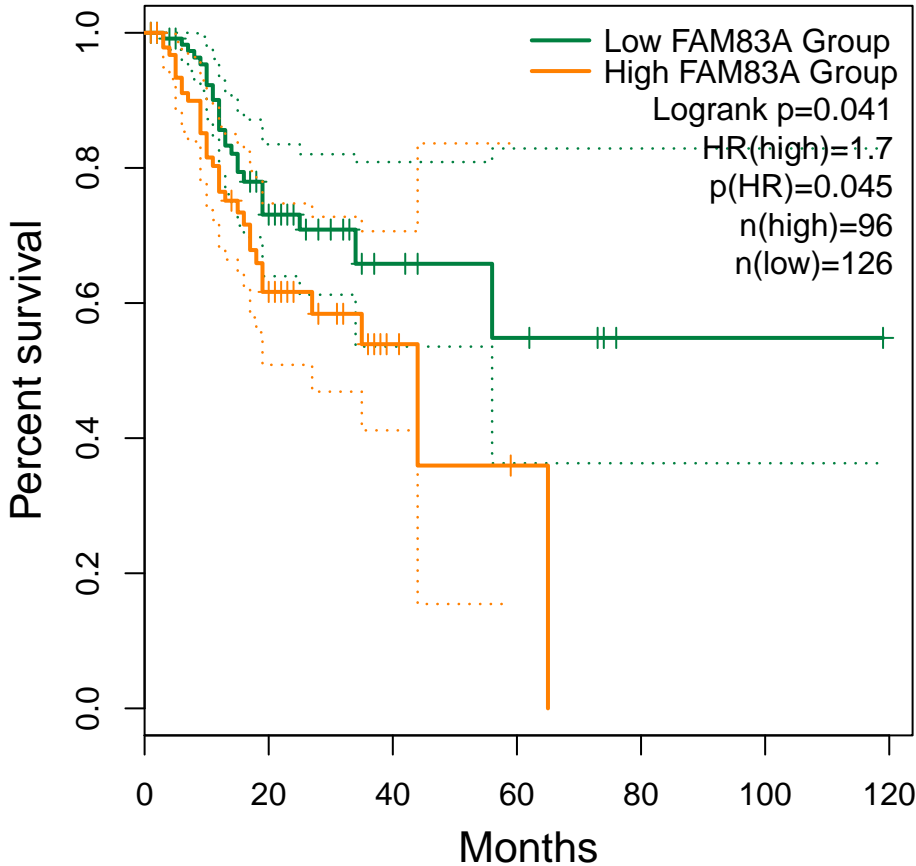

Supplement: Supplementary file 9 [file Data_Sheet_9.ZIP › Supplementary materials fig.8/DFS/FAM83A_survival_ozoCf.pdf]

# Disease Free Survival

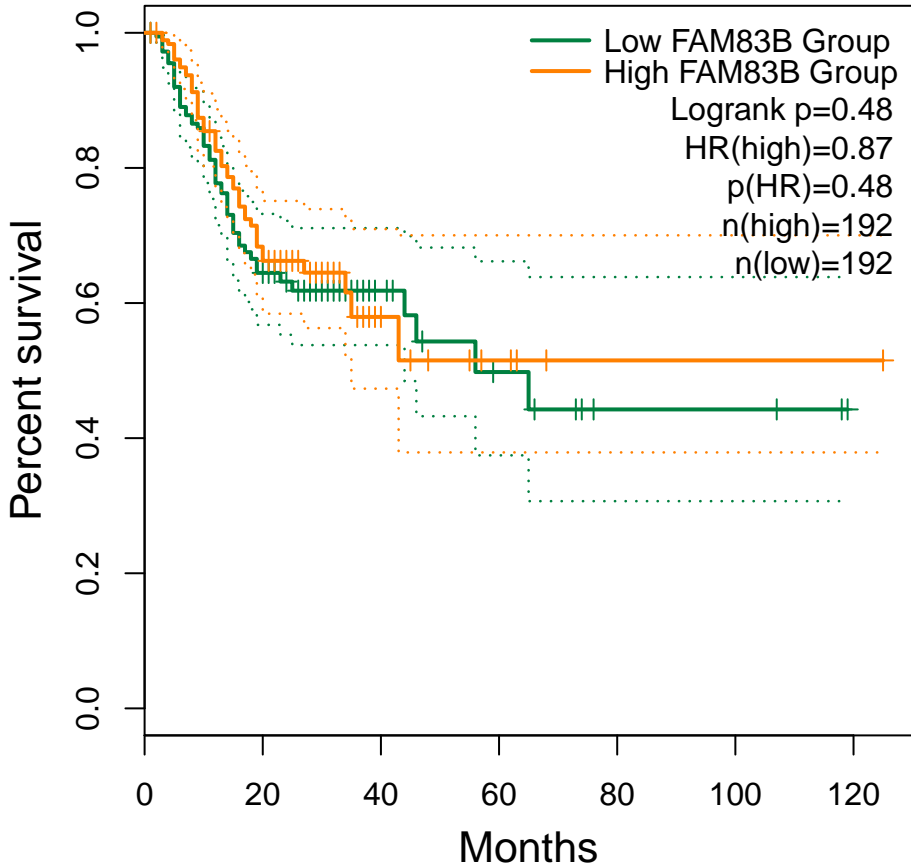

Supplement: Supplementary file 9 [file Data_Sheet_9.ZIP › Supplementary materials fig.8/DFS/FAM83B_survival_eHs3Q.pdf]

# Disease Free Survival

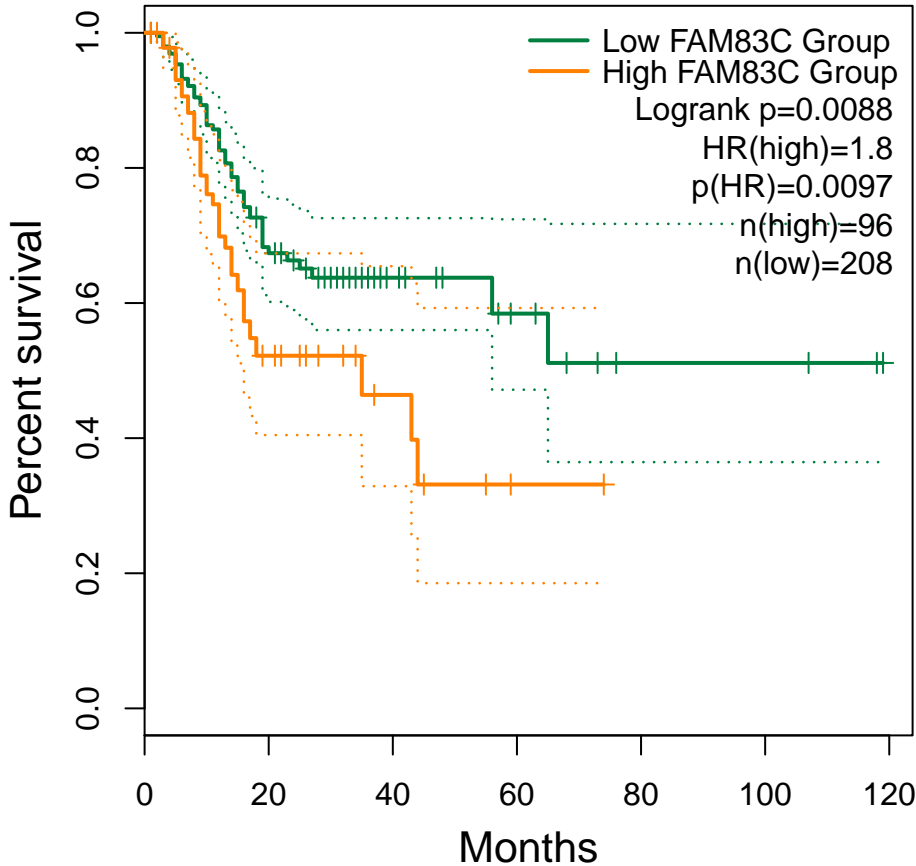

Supplement: Supplementary file 9 [file Data_Sheet_9.ZIP › Supplementary materials fig.8/DFS/FAM83C_survival_Z6yVg.pdf]

# Disease Free Survival

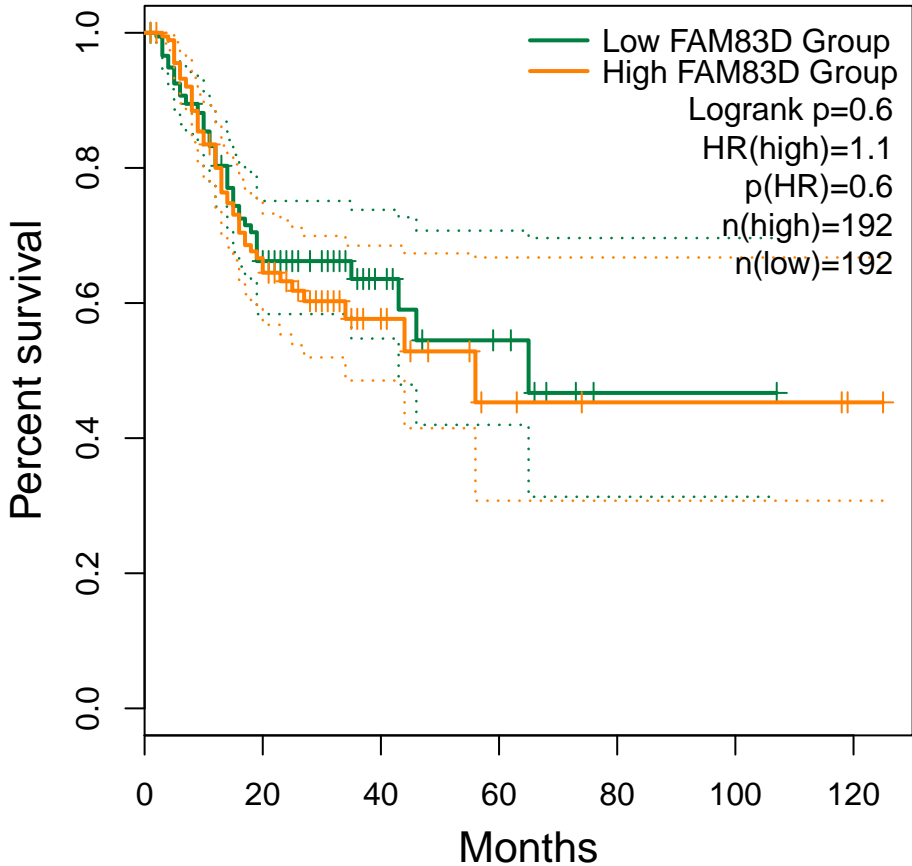

Supplement: Supplementary file 9 [file Data_Sheet_9.ZIP › Supplementary materials fig.8/DFS/FAM83D_survival_N9GiB.pdf]
