# Supplementary figures and images for: Comprehensive Analysis and Identification of Prognostic Biomarkers and Therapeutic Targets Among FAM83 Family Members for Gastric Cancer (part 2 of 2)
Source: Front Cell Dev Biol. 2021 Nov 19;9:719613. doi: 10.3389/fcell.2021.719613 (PMC8640971; doi:10.3389/fcell.2021.719613)

# Disease Free Survival

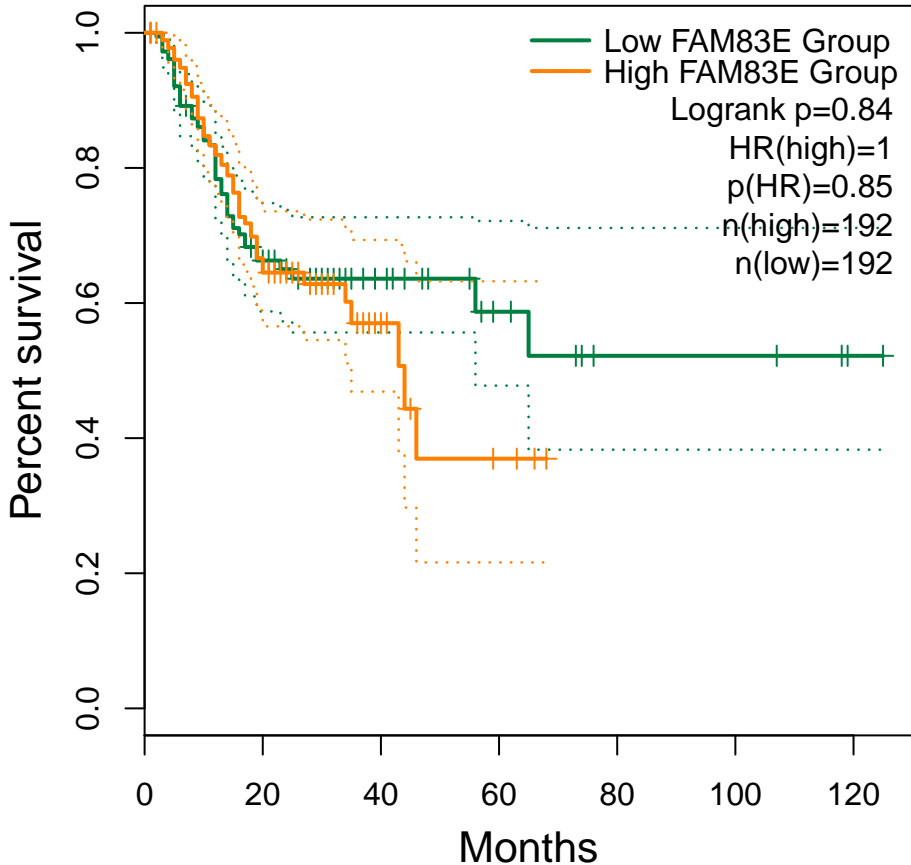

Supplement: Supplementary file 9 [file Data_Sheet_9.ZIP › Supplementary materials fig.8/DFS/FAM83E_survival_fL0T0.pdf]

# Disease Free Survival

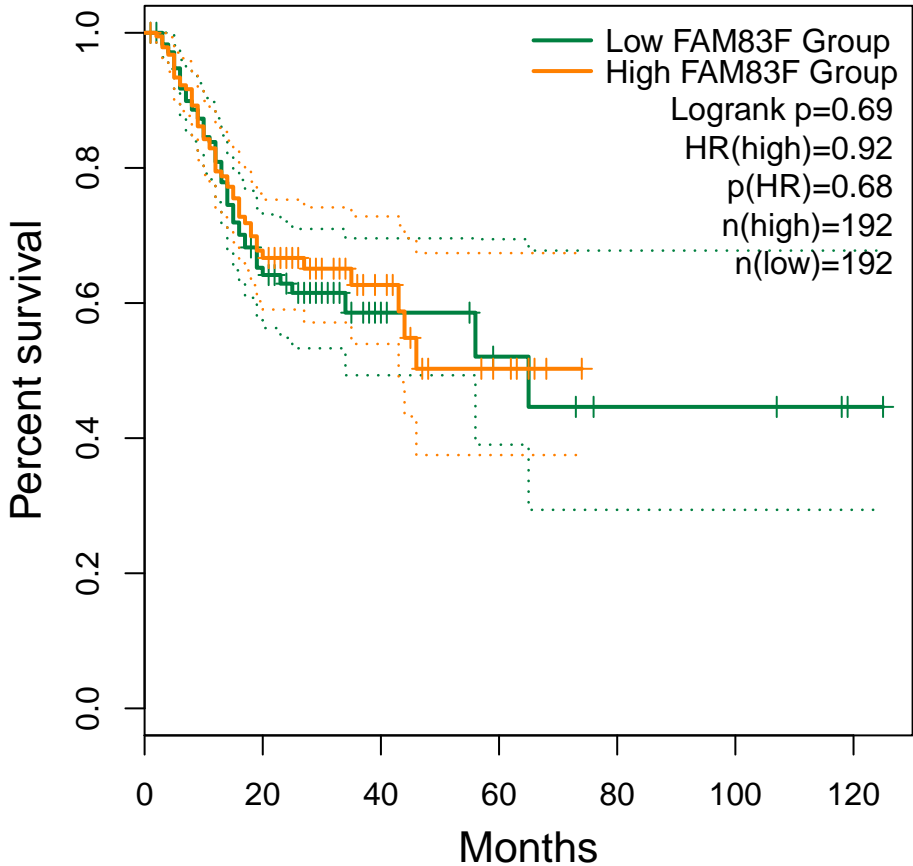

Supplement: Supplementary file 9 [file Data_Sheet_9.ZIP › Supplementary materials fig.8/DFS/FAM83F_survival_jndqa.pdf]

# Disease Free Survival

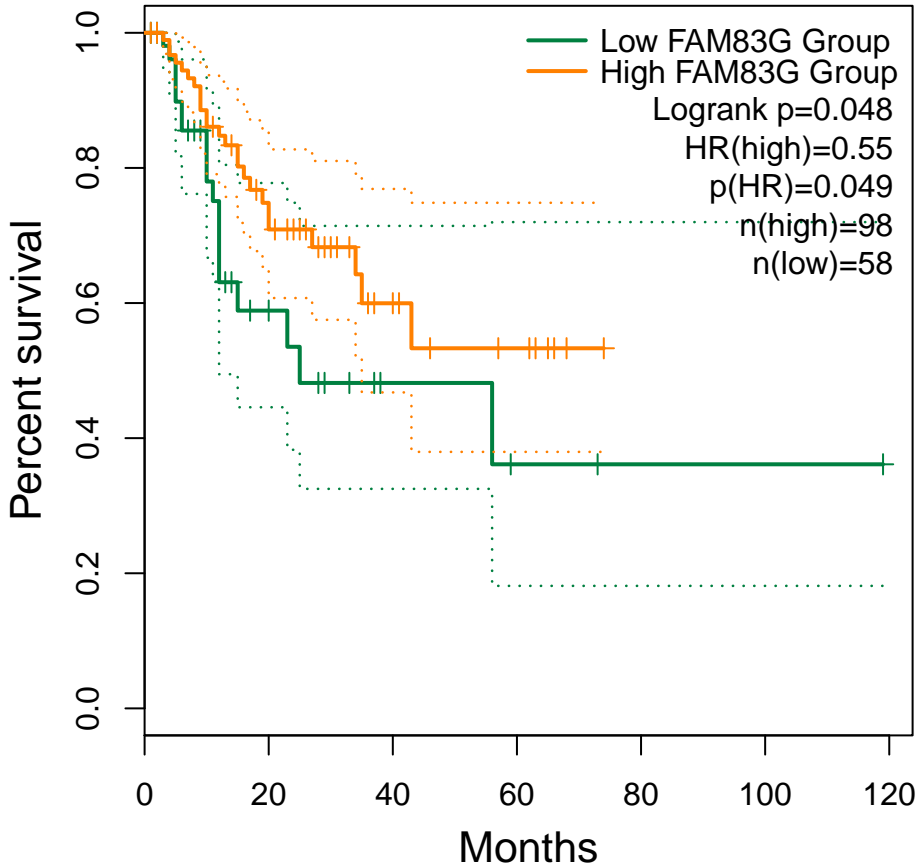

Supplement: Supplementary file 9 [file Data_Sheet_9.ZIP › Supplementary materials fig.8/DFS/FAM83G_survival_rSE4L.pdf]

# Disease Free Survival

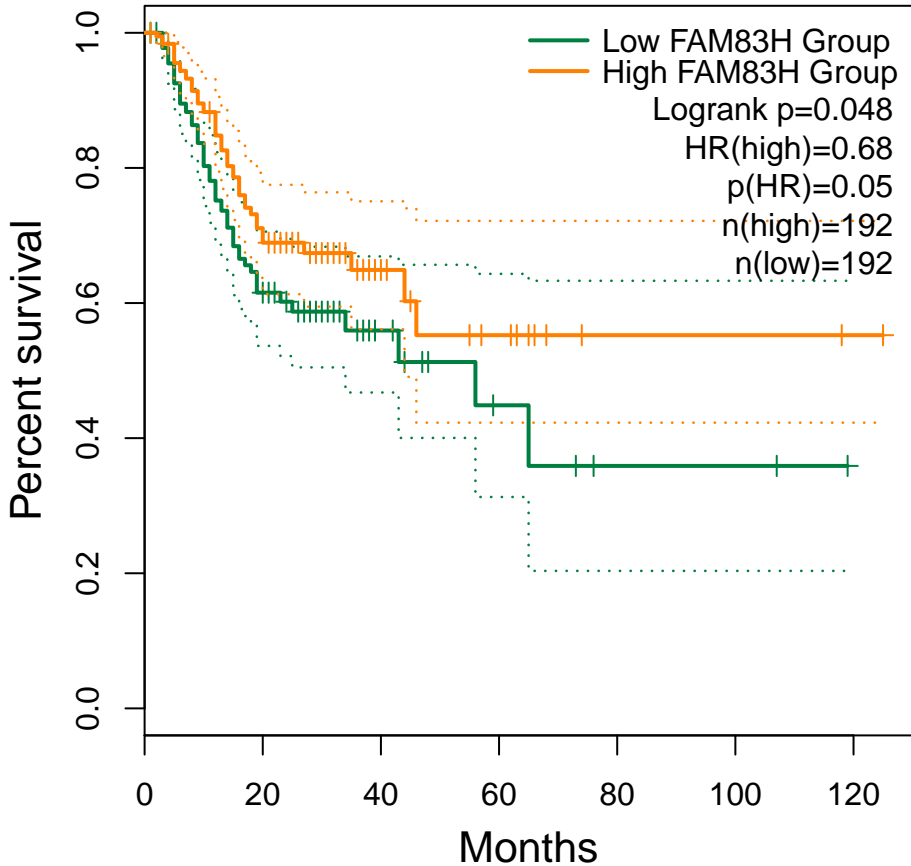

Supplement: Supplementary file 9 [file Data_Sheet_9.ZIP › Supplementary materials fig.8/DFS/FAM83H_survival_YojET.pdf]

# Overall Survival

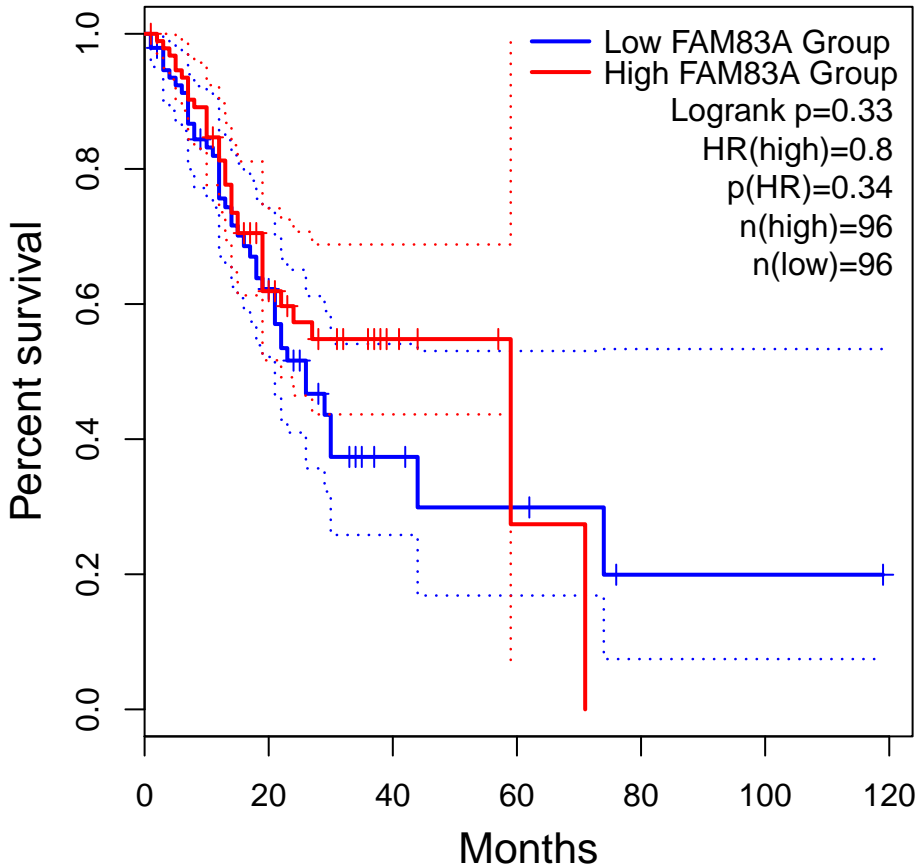

Supplement: Supplementary file 9 [file Data_Sheet_9.ZIP › Supplementary materials fig.8/OS/FAM83A_survival_LFgFf.pdf]

# Overall Survival

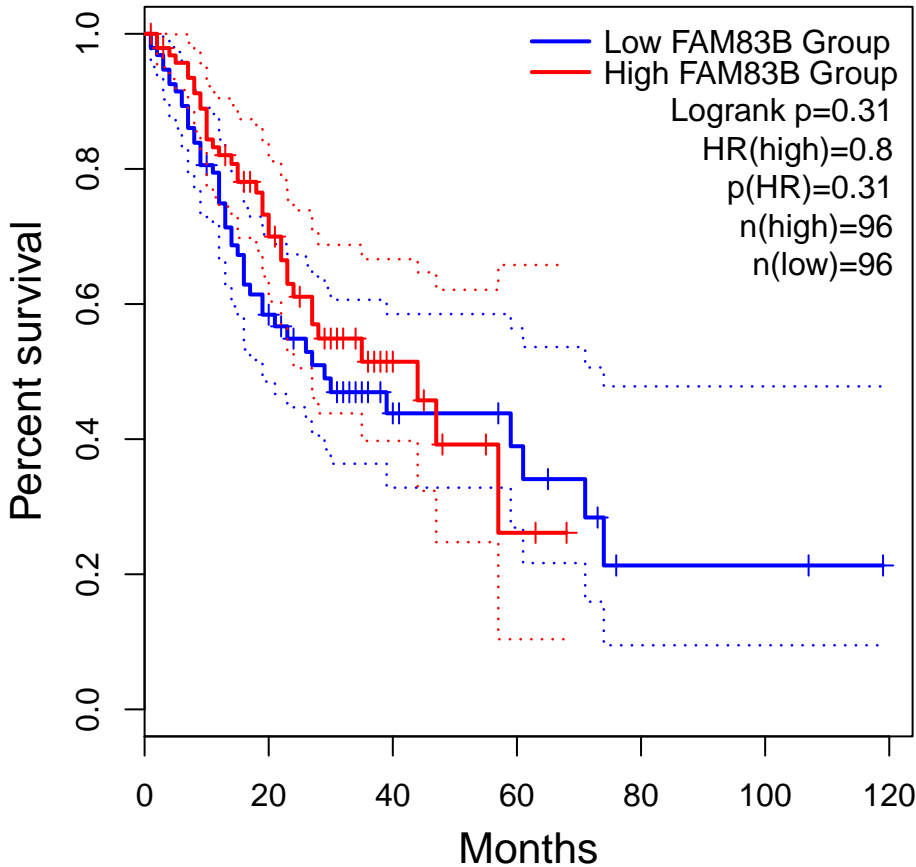

Supplement: Supplementary file 9 [file Data_Sheet_9.ZIP › Supplementary materials fig.8/OS/FAM83B_survival_SGDvE.pdf]

# Overall Survival

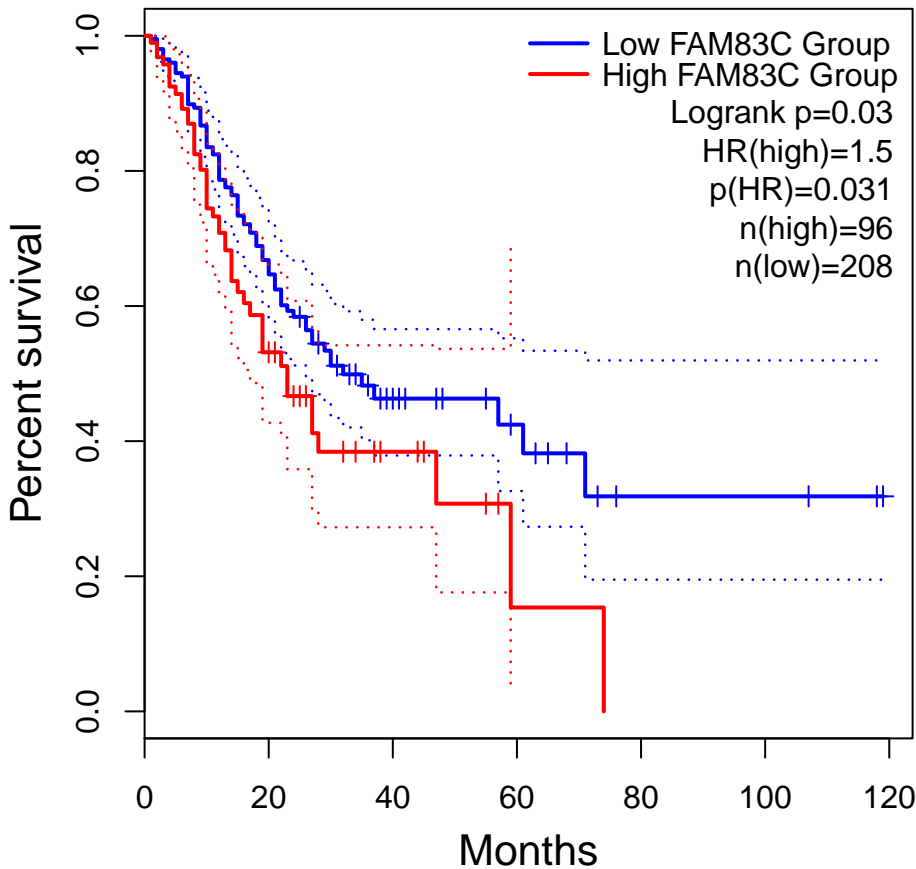

Supplement: Supplementary file 9 [file Data_Sheet_9.ZIP › Supplementary materials fig.8/OS/FAM83C_survival_AxVf5.pdf]

# Overall Survival

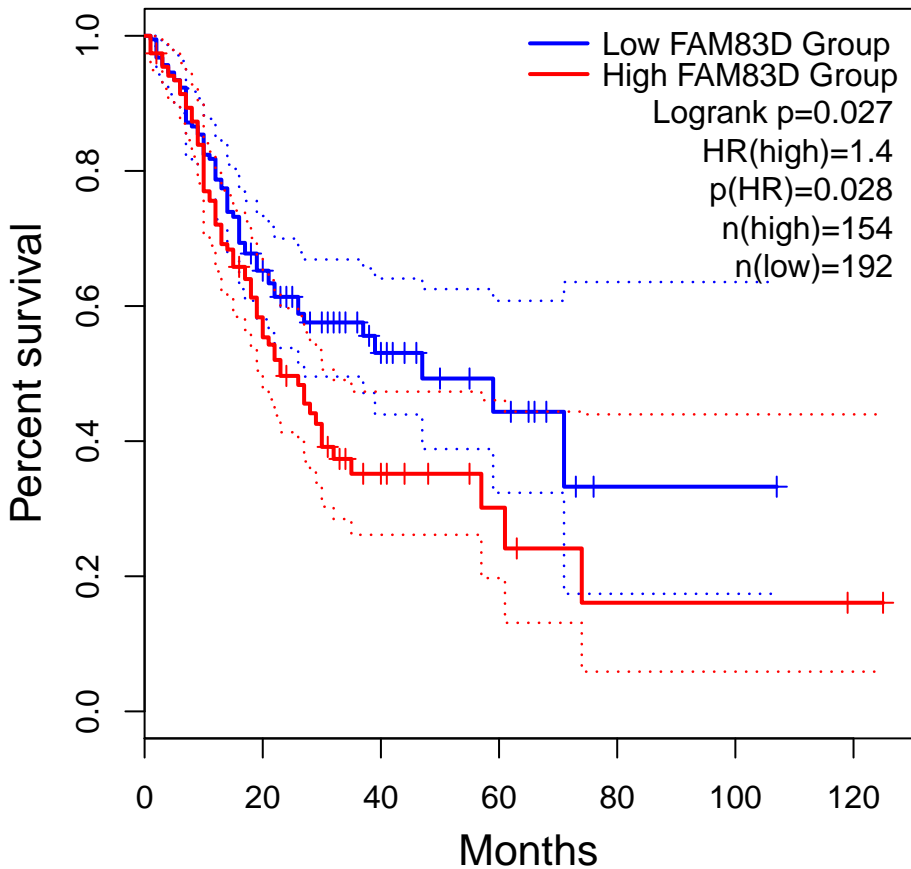

Supplement: Supplementary file 9 [file Data_Sheet_9.ZIP › Supplementary materials fig.8/OS/FAM83D_survival_u6MCw.pdf]

# Overall Survival

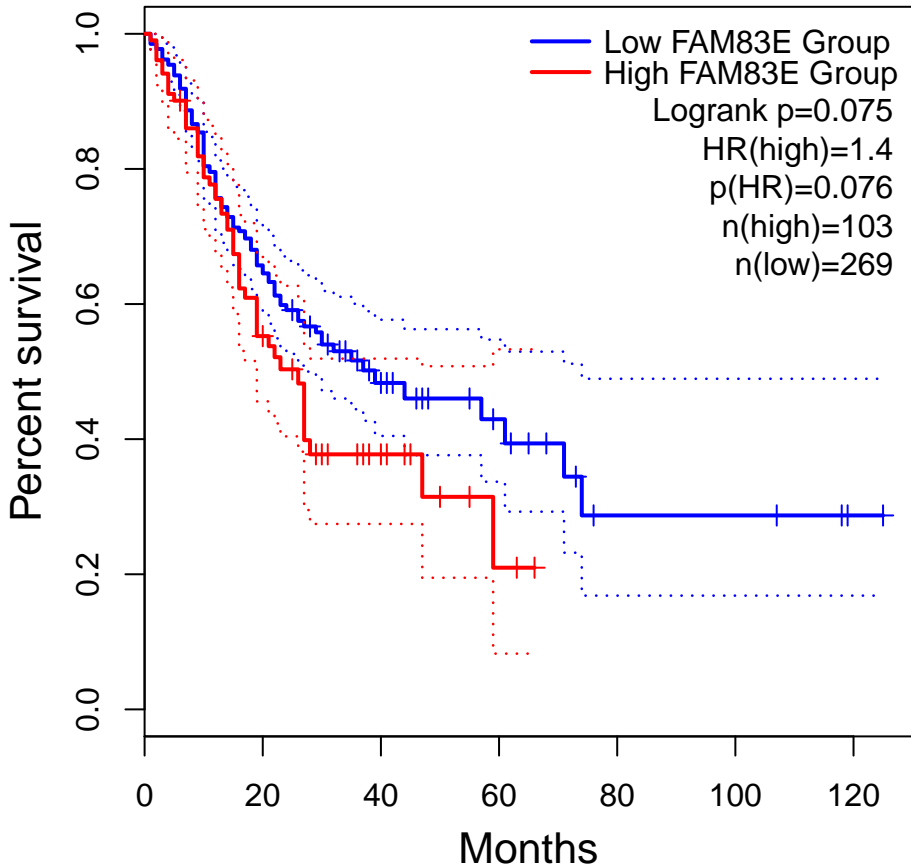

Supplement: Supplementary file 9 [file Data_Sheet_9.ZIP › Supplementary materials fig.8/OS/FAM83E_survival_CWZvu.pdf]

# Overall Survival

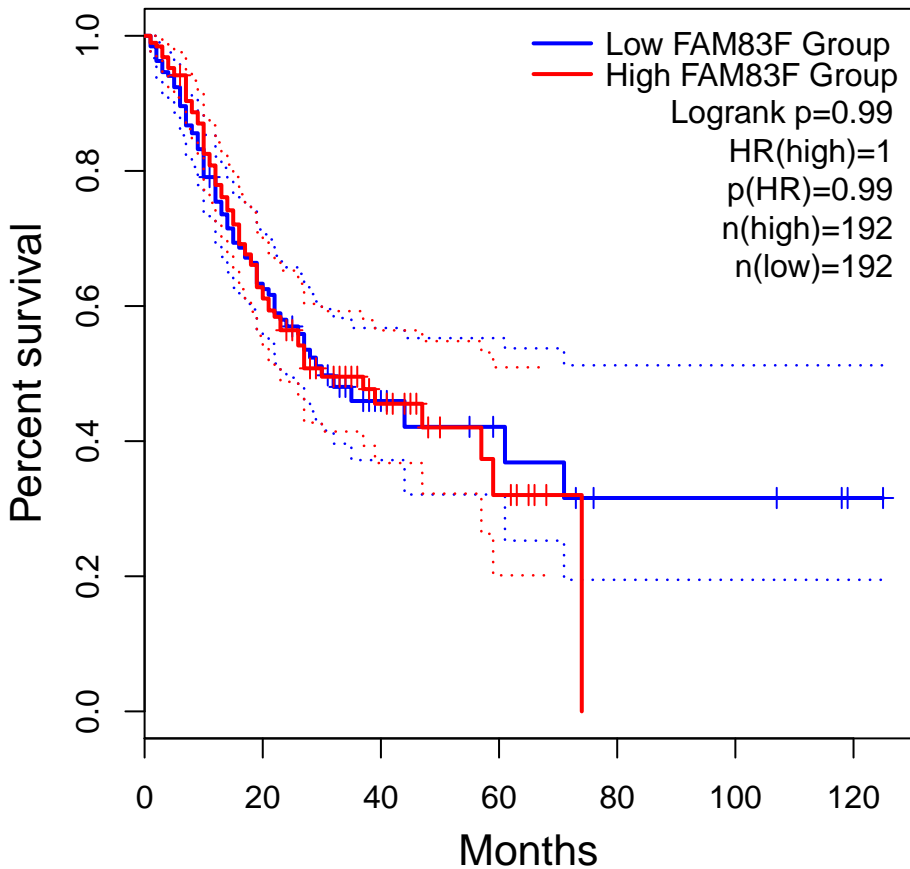

Supplement: Supplementary file 9 [file Data_Sheet_9.ZIP › Supplementary materials fig.8/OS/FAM83F_survival_H7EFD.pdf]

# Overall Survival

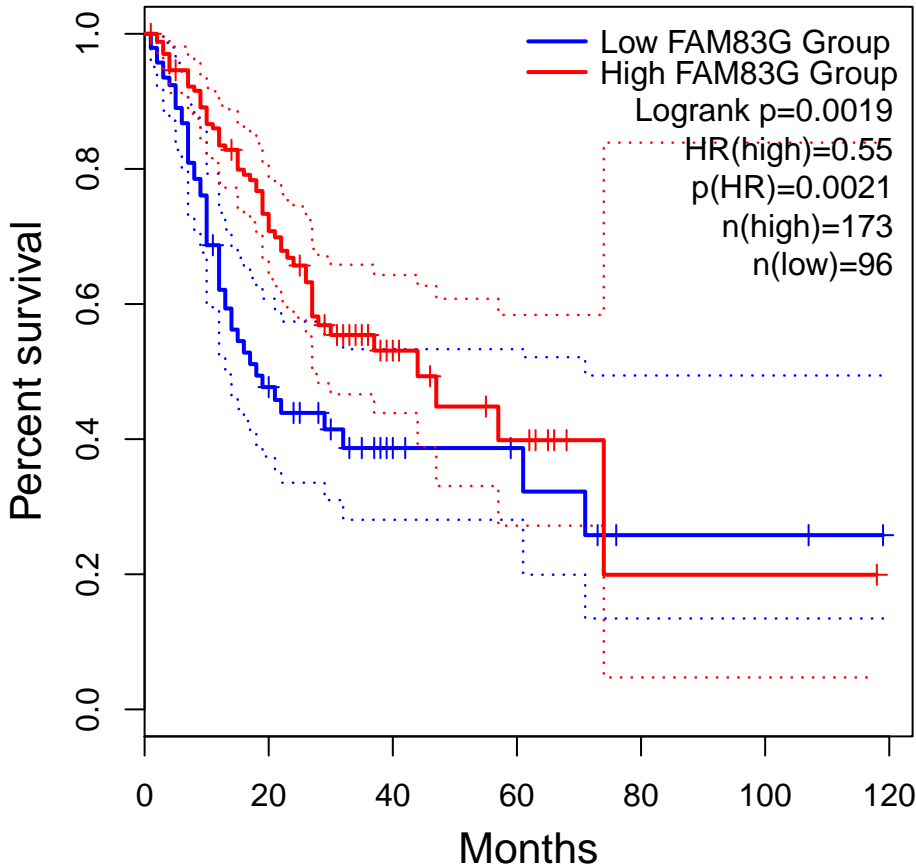

Supplement: Supplementary file 9 [file Data_Sheet_9.ZIP › Supplementary materials fig.8/OS/FAM83G_survival_oLKyO.pdf]

# Overall Survival

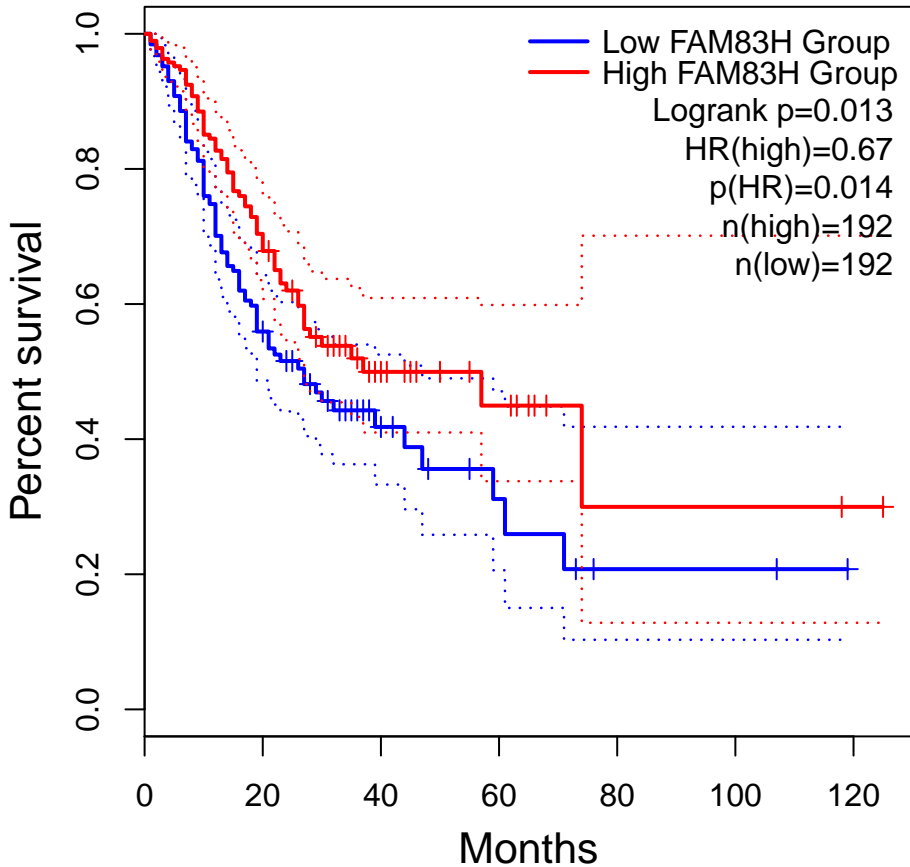

Supplement: Supplementary file 9 [file Data_Sheet_9.ZIP › Supplementary materials fig.8/OS/FAM83H_survival_DxZTt.pdf]

Coefficients

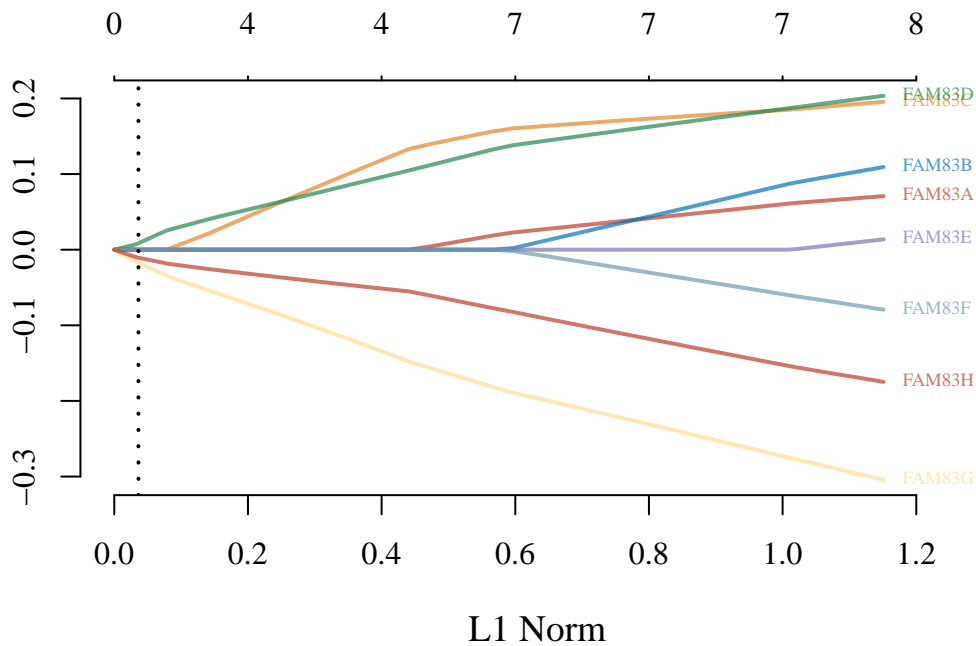

Supplement: Supplementary file 10 [file Data_Sheet_10.ZIP › Original source data of supplementary figure/Supplementary fig.1/supplementary fig1A.pdf]

Partial Likelihood Deviance

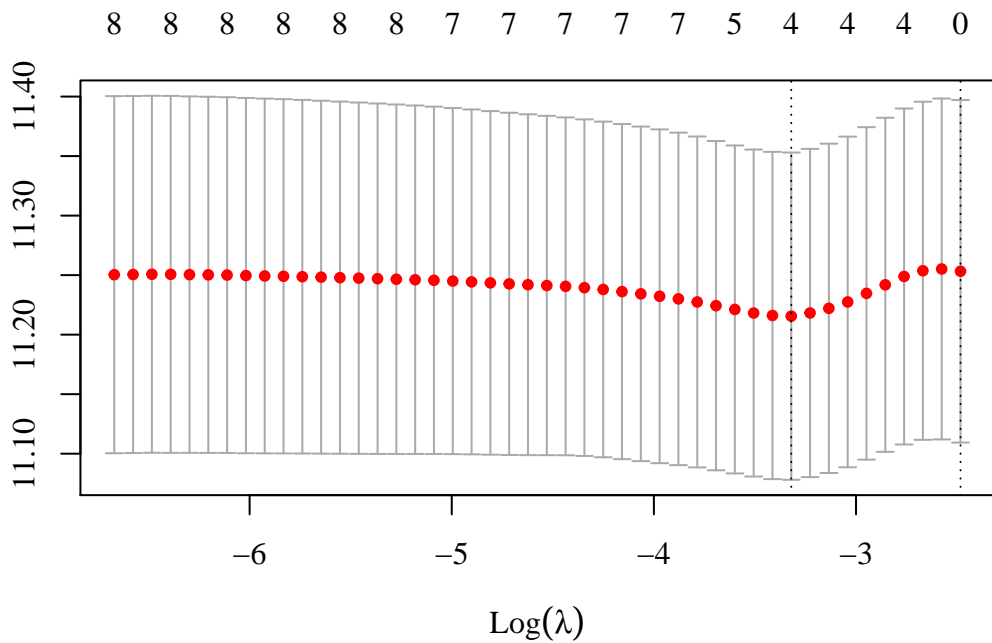

Supplement: Supplementary file 10 [file Data_Sheet_10.ZIP › Original source data of supplementary figure/Supplementary fig.1/supplementary fig1B.pdf]

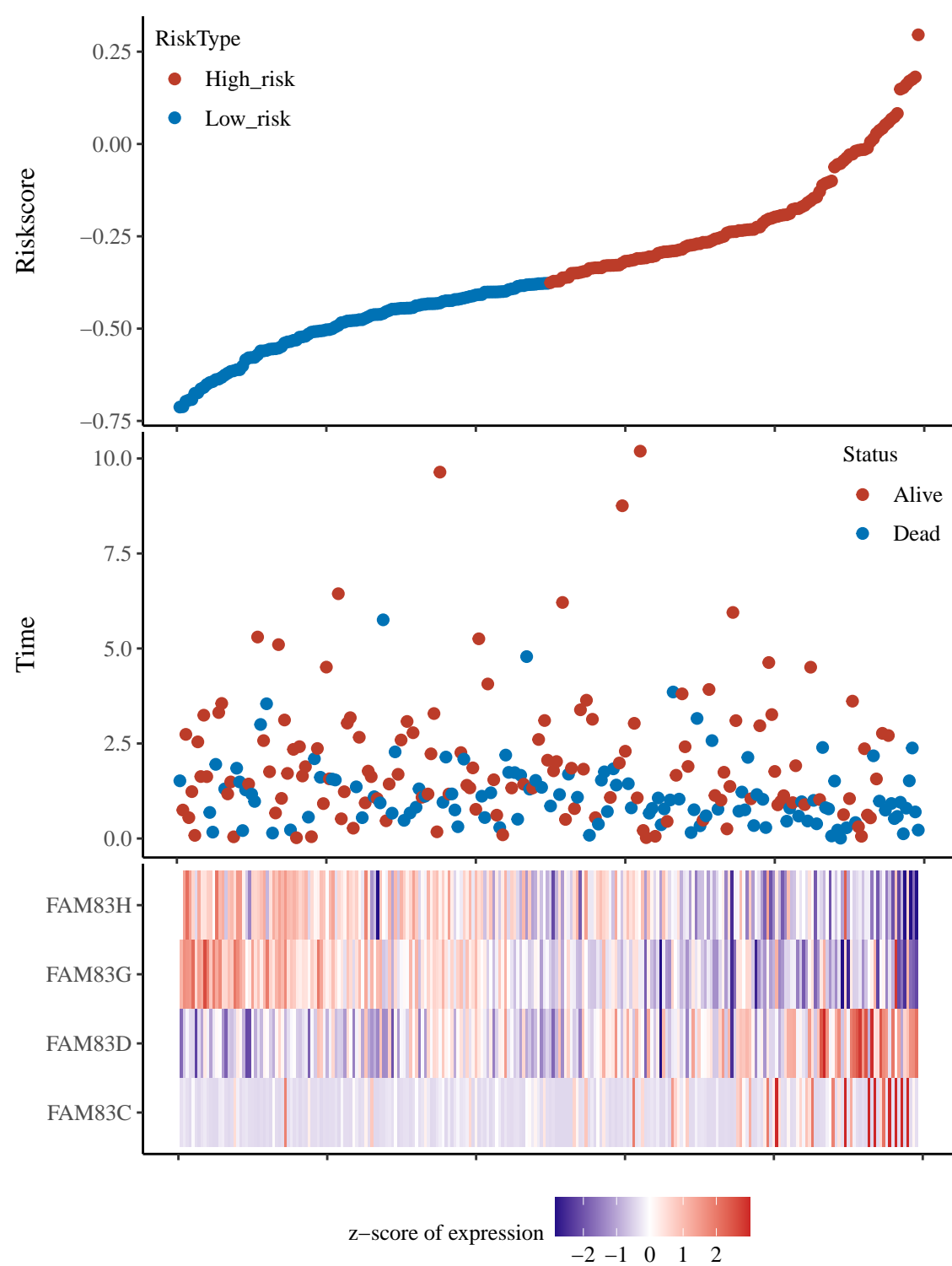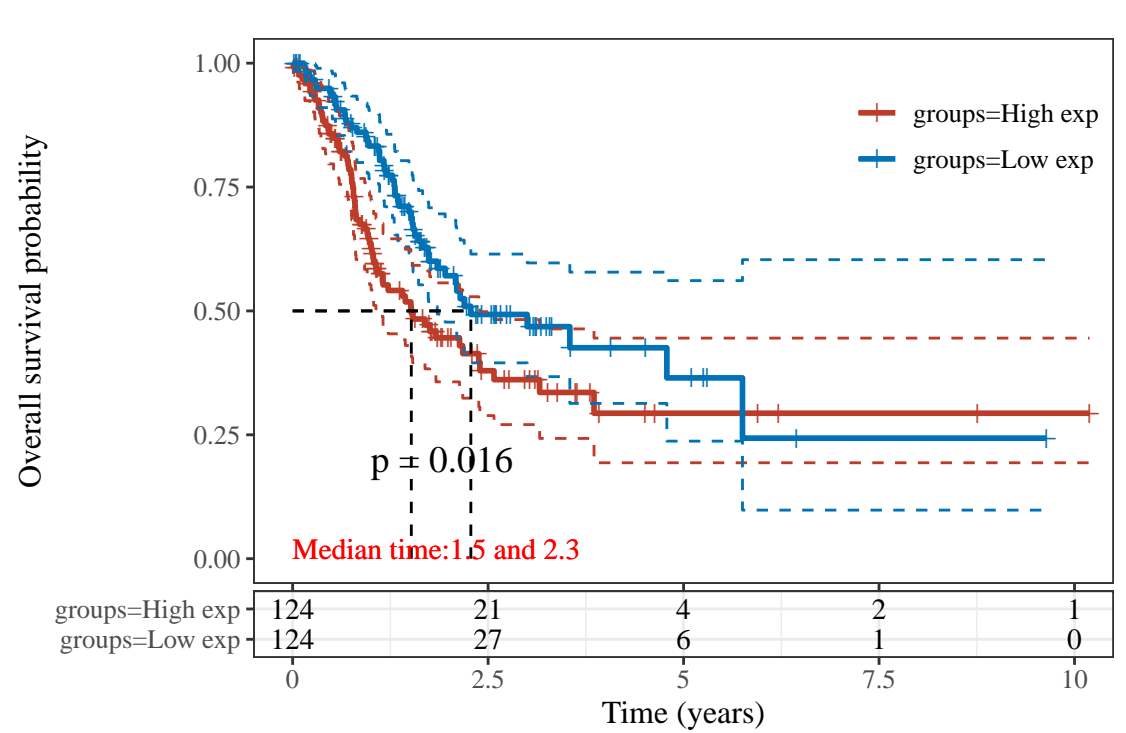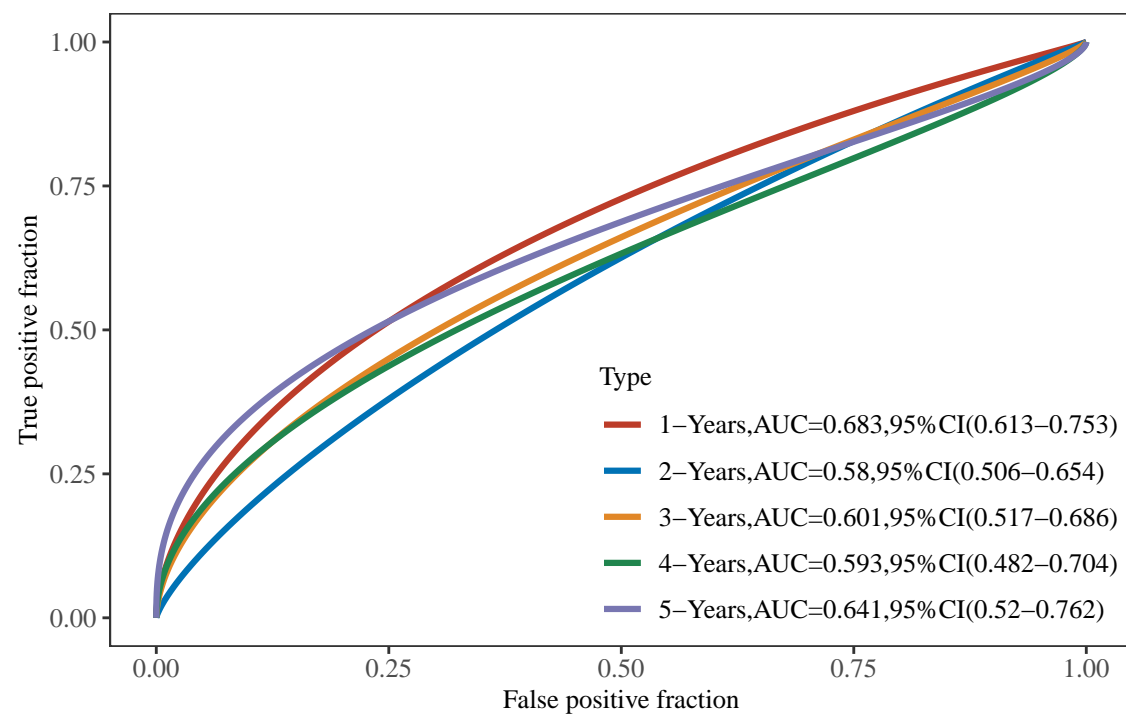

Supplement: Supplementary file 10 [file Data_Sheet_10.ZIP › Original source data of supplementary figure/Supplementary fig.1/supplementary fig1C,D,E.pdf]

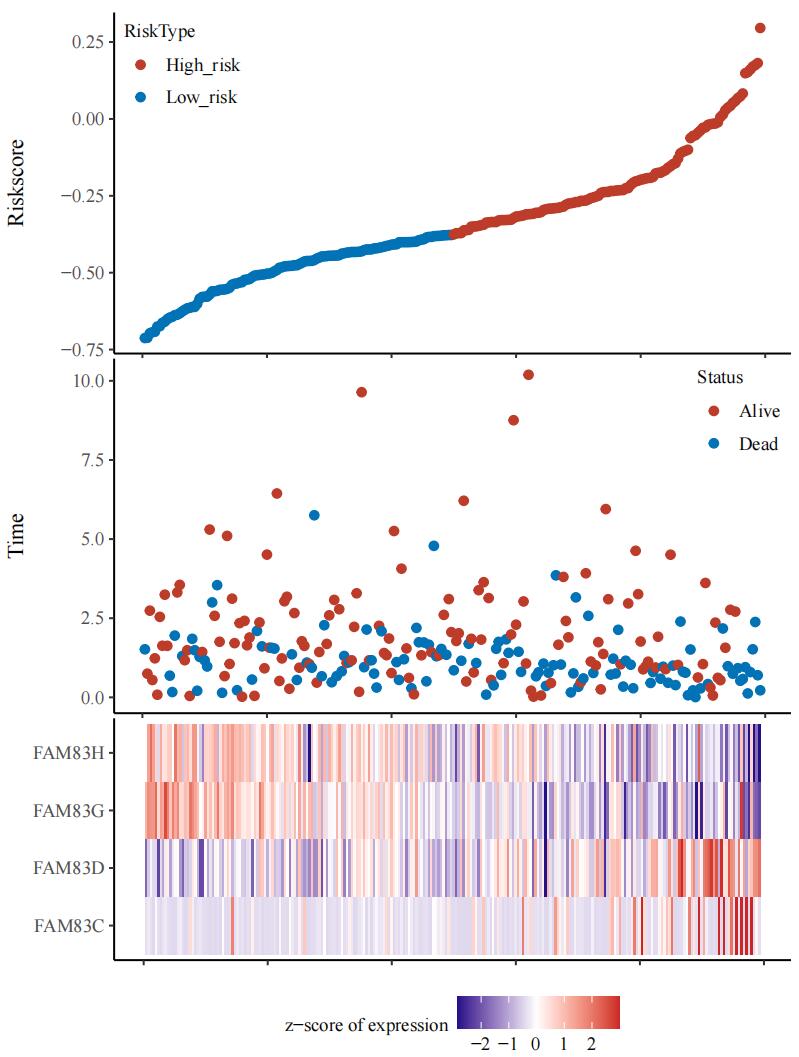

Supplement: Supplementary file 10 [file Data_Sheet_10.ZIP › Original source data of supplementary figure/Supplementary fig.1/supplementary fig1C.jpg]

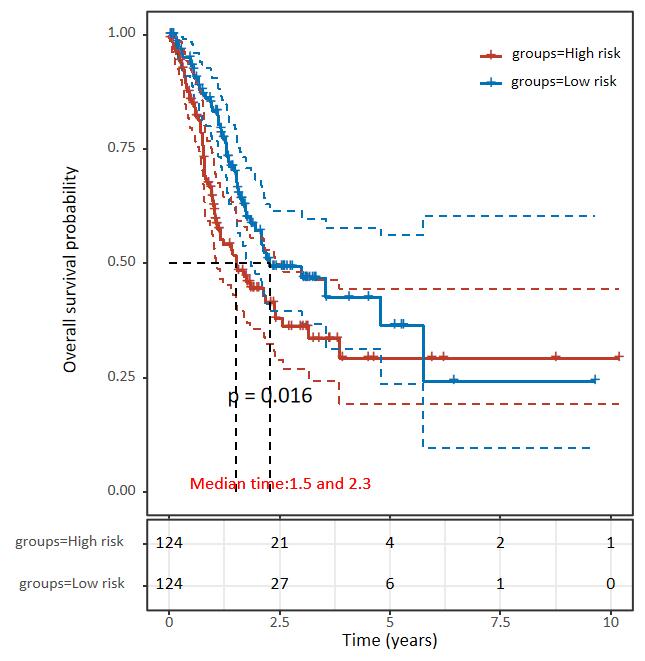

Supplement: Supplementary file 10 [file Data_Sheet_10.ZIP › Original source data of supplementary figure/Supplementary fig.1/supplementary fig1D.jpg]

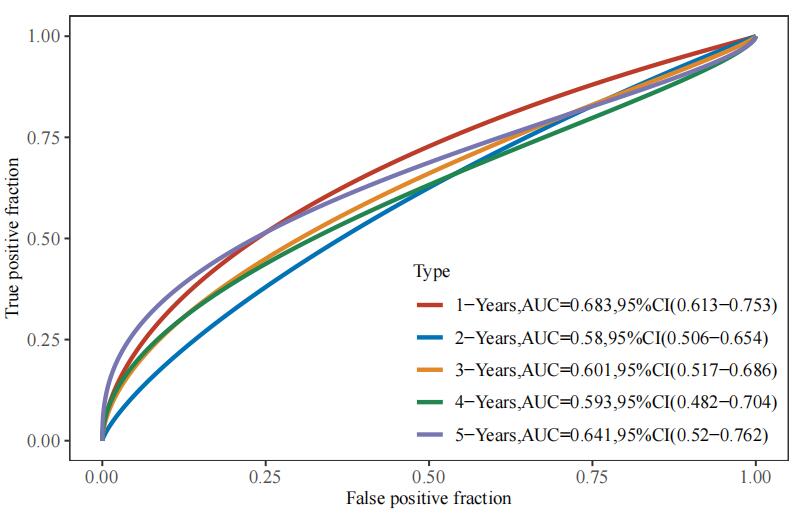

Supplement: Supplementary file 10 [file Data_Sheet_10.ZIP › Original source data of supplementary figure/Supplementary fig.1/supplementary fig1E.jpg]

Coefficients

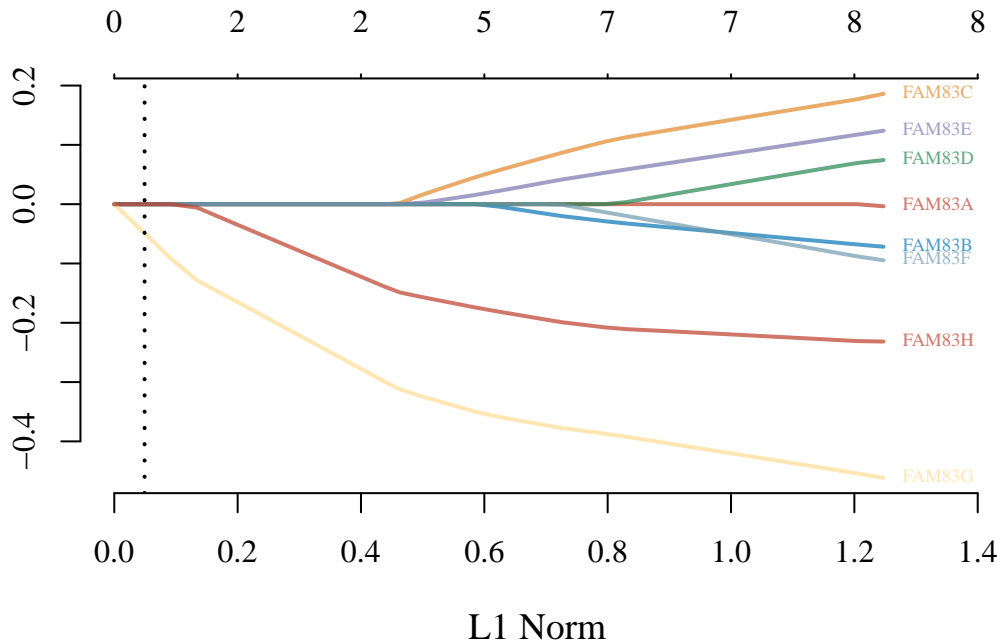

Supplement: Supplementary file 10 [file Data_Sheet_10.ZIP › Original source data of supplementary figure/Supplementary fig.2/supplementary fig2A.pdf]

Partial Likelihood Deviance

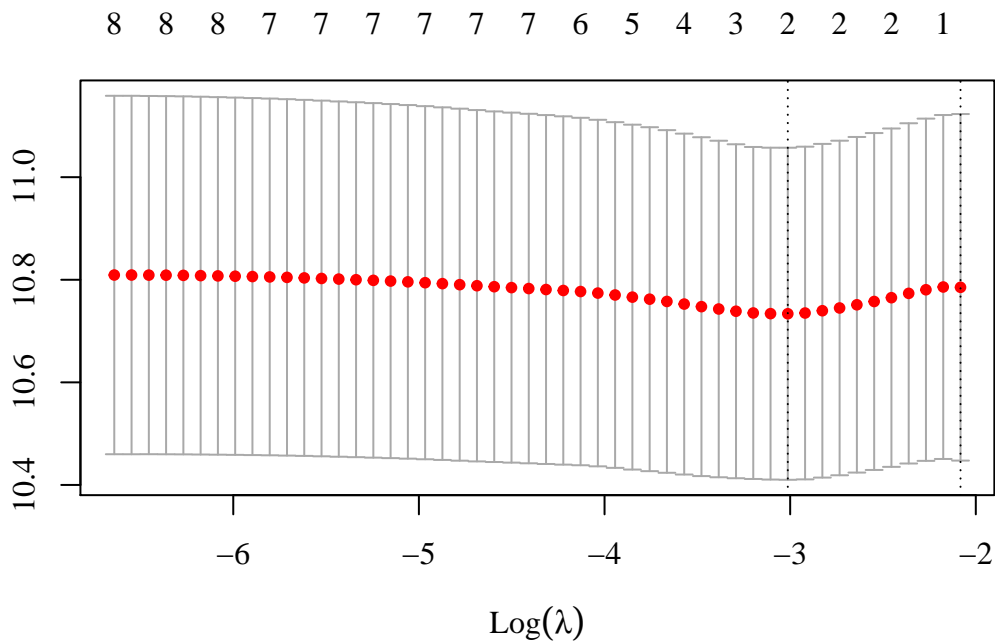

Supplement: Supplementary file 10 [file Data_Sheet_10.ZIP › Original source data of supplementary figure/Supplementary fig.2/supplementary fig2B.pdf]

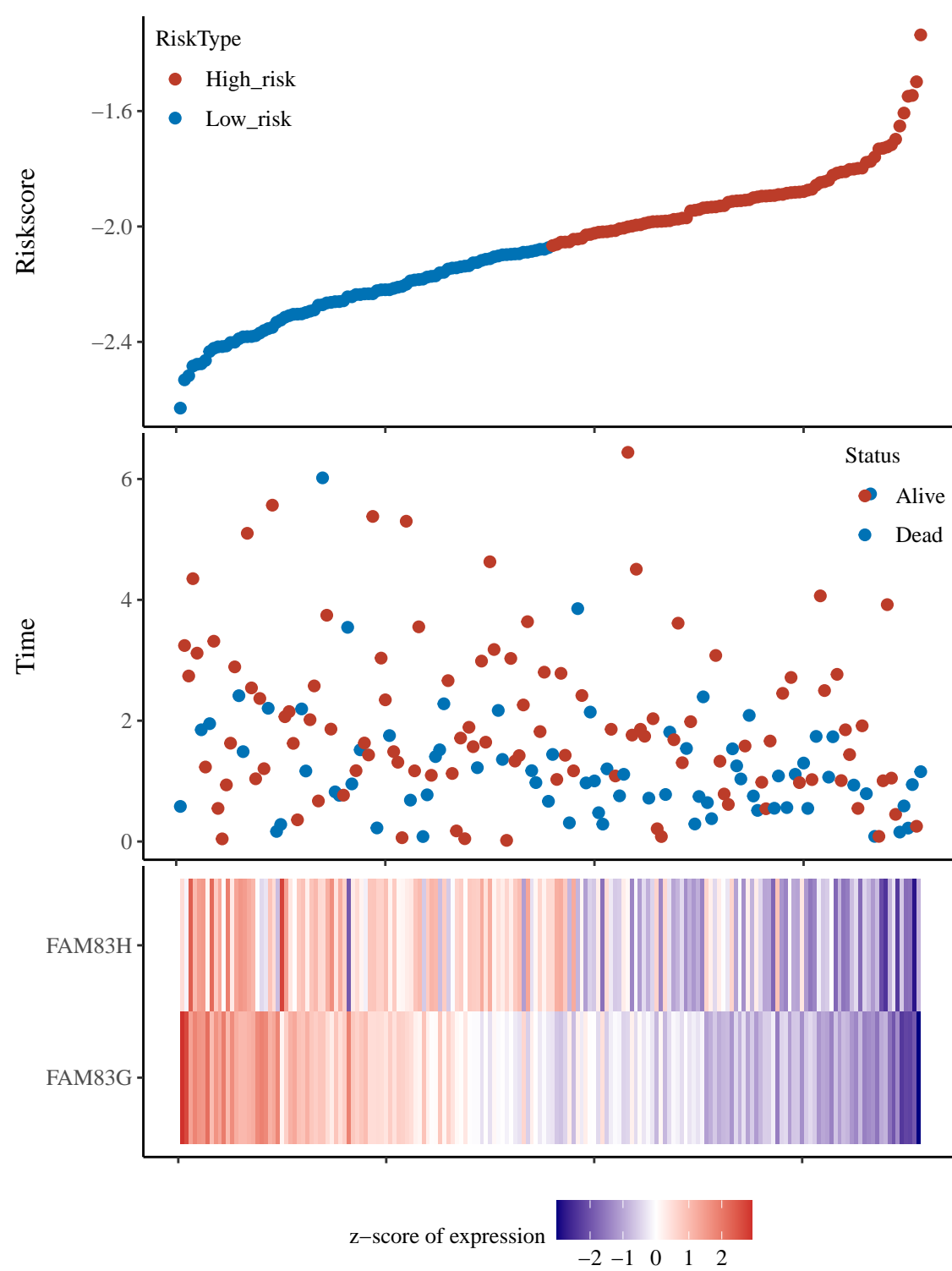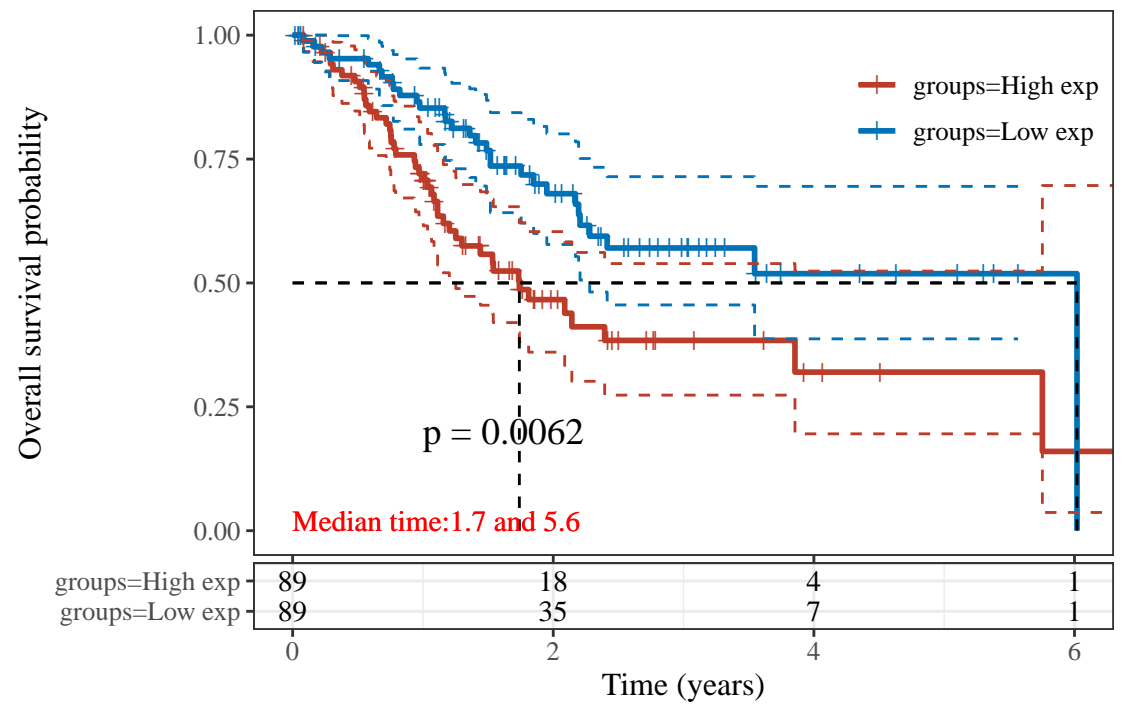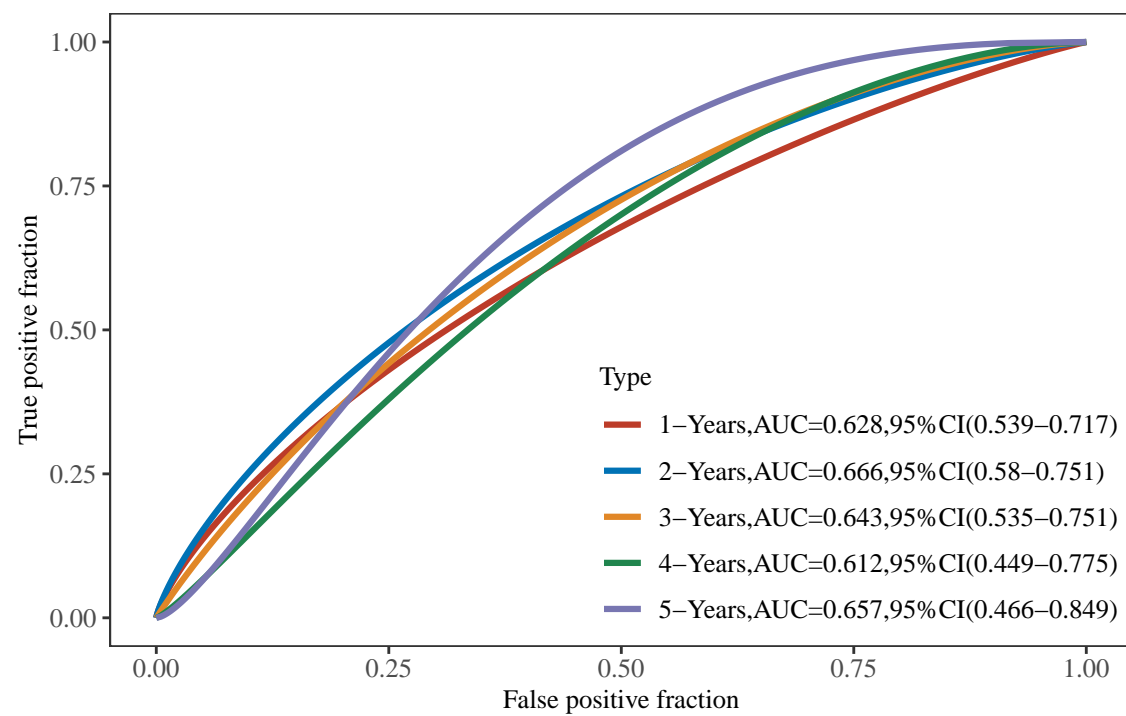

Supplement: Supplementary file 10 [file Data_Sheet_10.ZIP › Original source data of supplementary figure/Supplementary fig.2/supplementary fig2C,D,E.pdf]

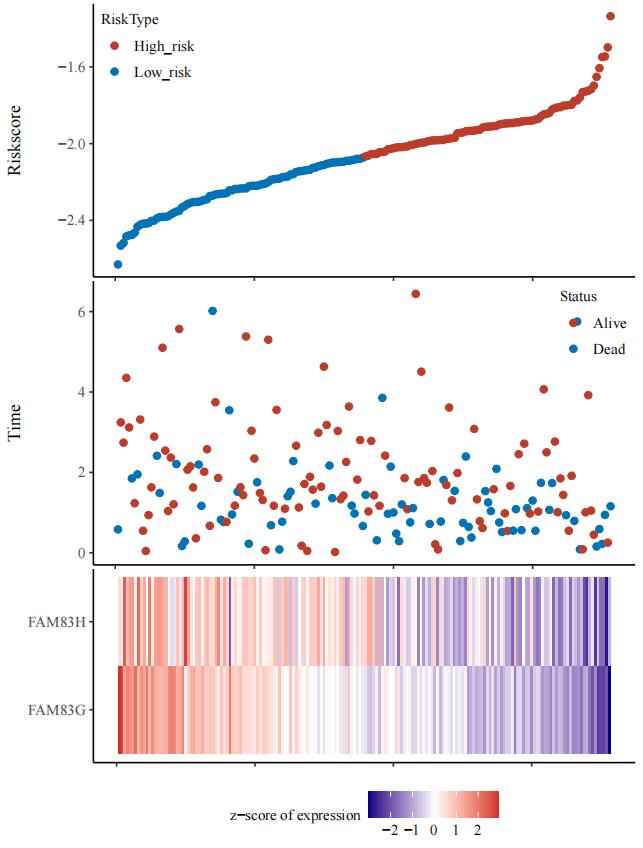

Supplement: Supplementary file 10 [file Data_Sheet_10.ZIP › Original source data of supplementary figure/Supplementary fig.2/supplementary fig2C.jpg]

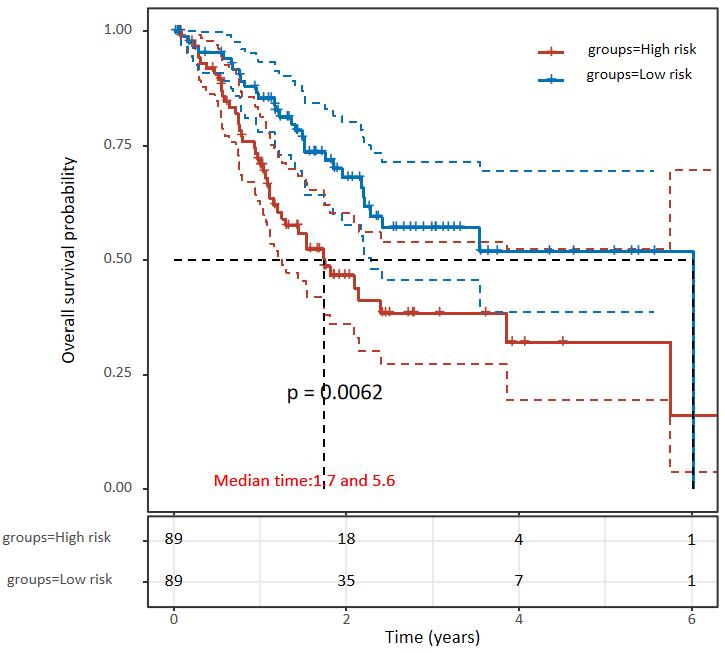

Supplement: Supplementary file 10 [file Data_Sheet_10.ZIP › Original source data of supplementary figure/Supplementary fig.2/supplementary fig2D.jpg]

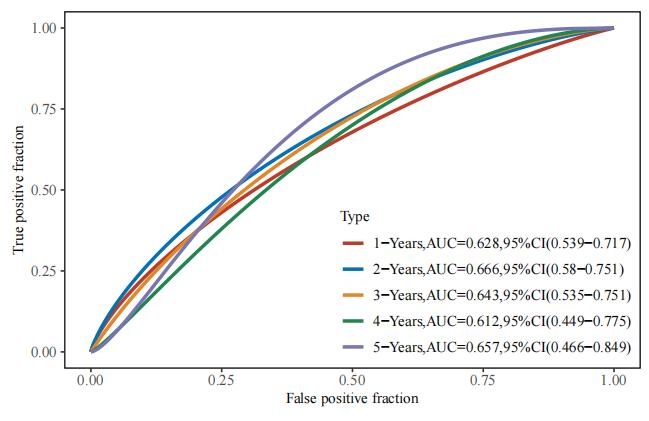

Supplement: Supplementary file 10 [file Data_Sheet_10.ZIP › Original source data of supplementary figure/Supplementary fig.2/supplementary fig2E.jpg]

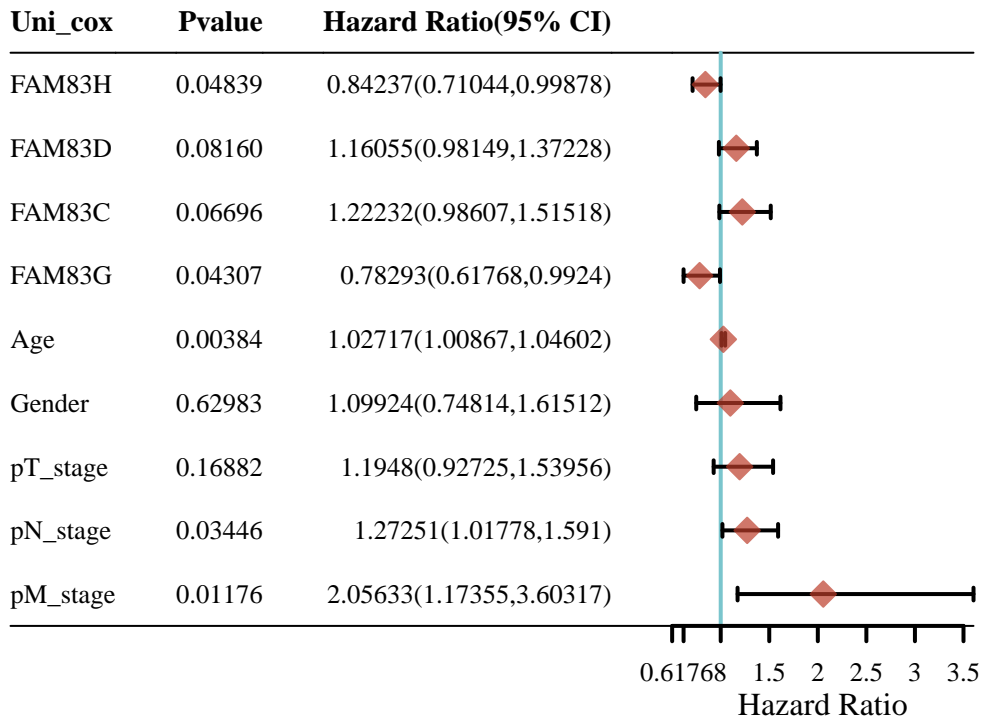

Supplement: Supplementary file 10 [file Data_Sheet_10.ZIP › Original source data of supplementary figure/Supplementary fig.3/supplementary fig3A.pdf]

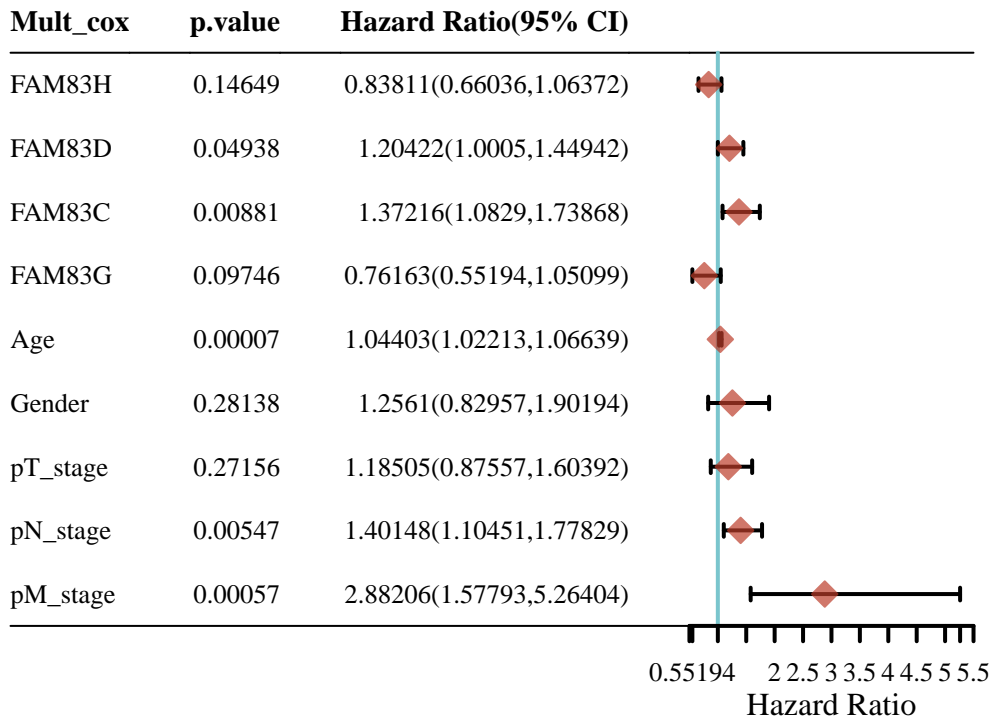

Supplement: Supplementary file 10 [file Data_Sheet_10.ZIP › Original source data of supplementary figure/Supplementary fig.3/supplementary fig3B.pdf]

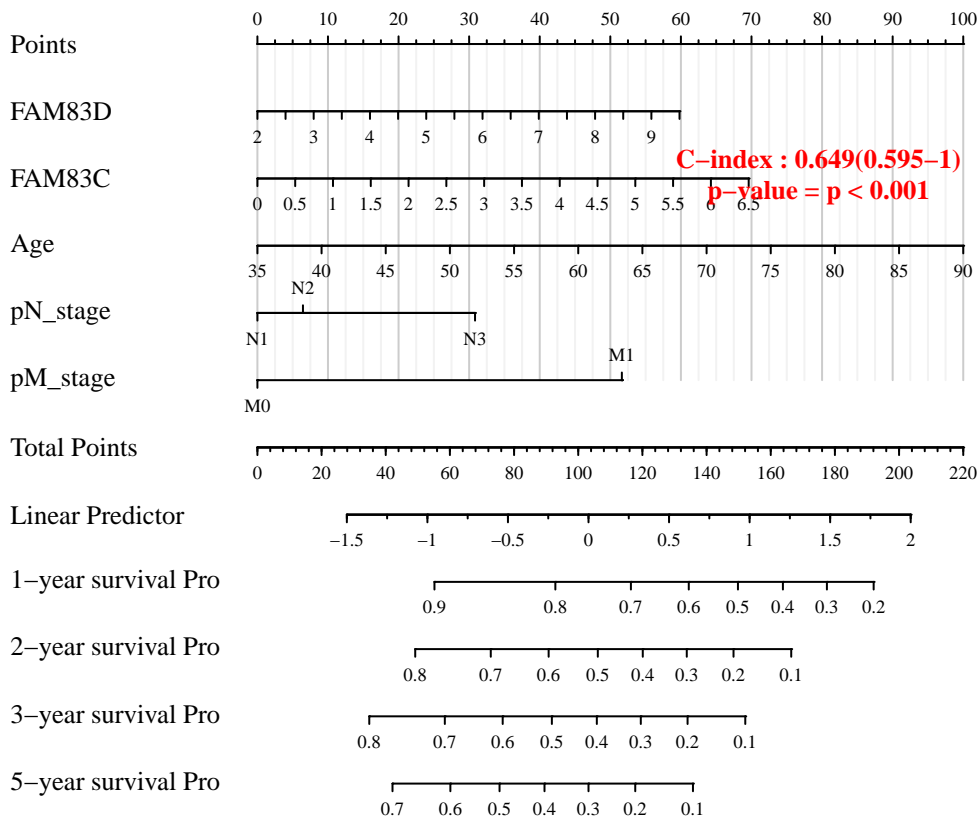

Supplement: Supplementary file 10 [file Data_Sheet_10.ZIP › Original source data of supplementary figure/Supplementary fig.3/supplementary fig3C.pdf]

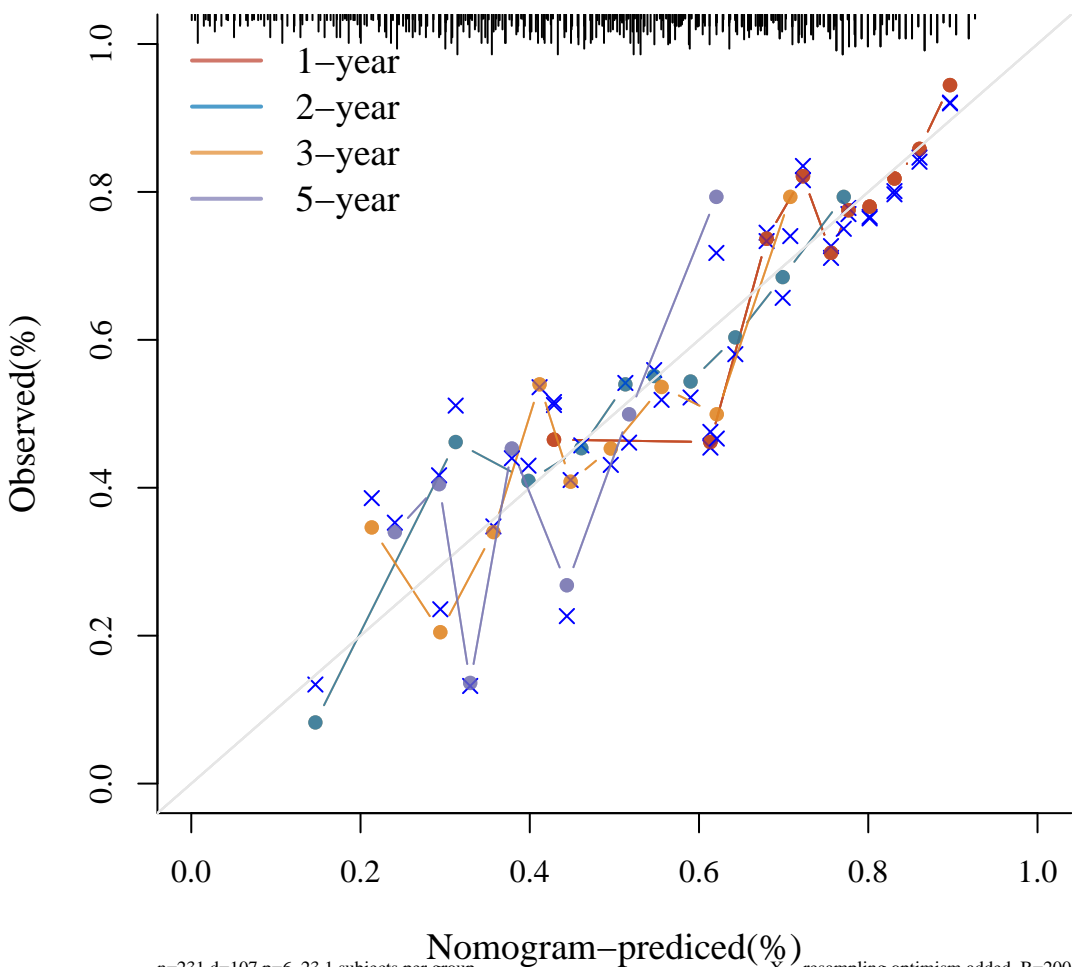

Supplement: Supplementary file 10 [file Data_Sheet_10.ZIP › Original source data of supplementary figure/Supplementary fig.3/supplementary fig3D.pdf]

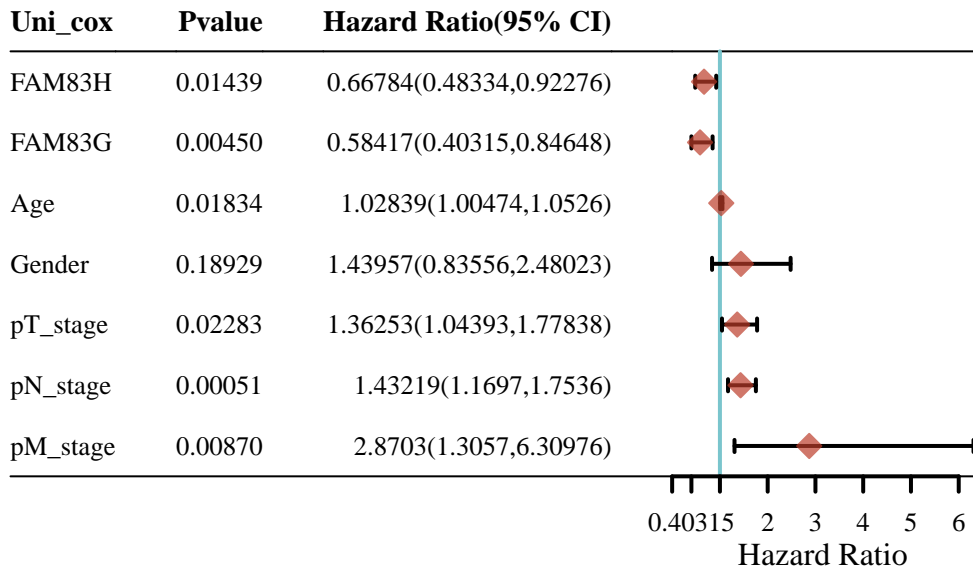

Supplement: Supplementary file 10 [file Data_Sheet_10.ZIP › Original source data of supplementary figure/Supplementary fig.4/supplementary fig4A.pdf]

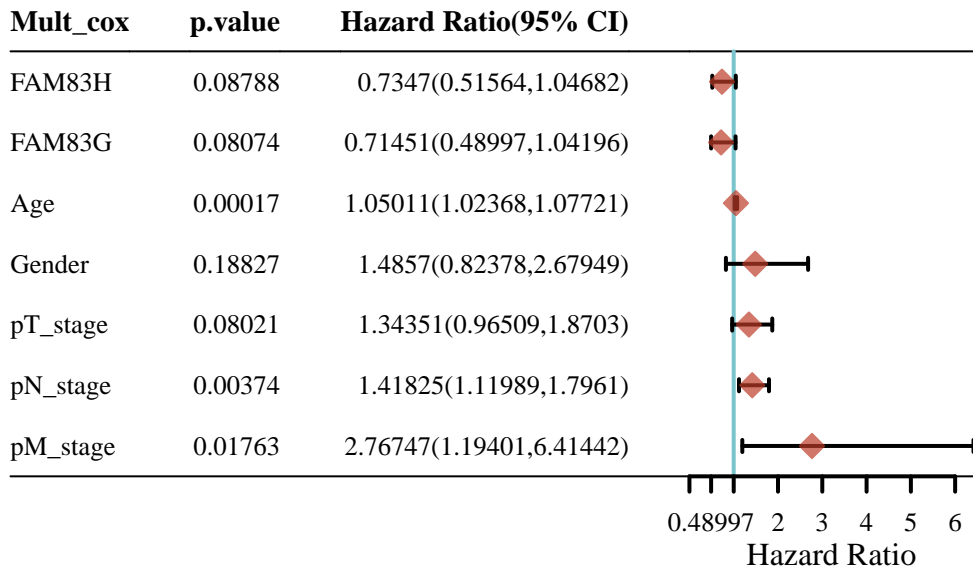

Supplement: Supplementary file 10 [file Data_Sheet_10.ZIP › Original source data of supplementary figure/Supplementary fig.4/supplementary fig4B.pdf]

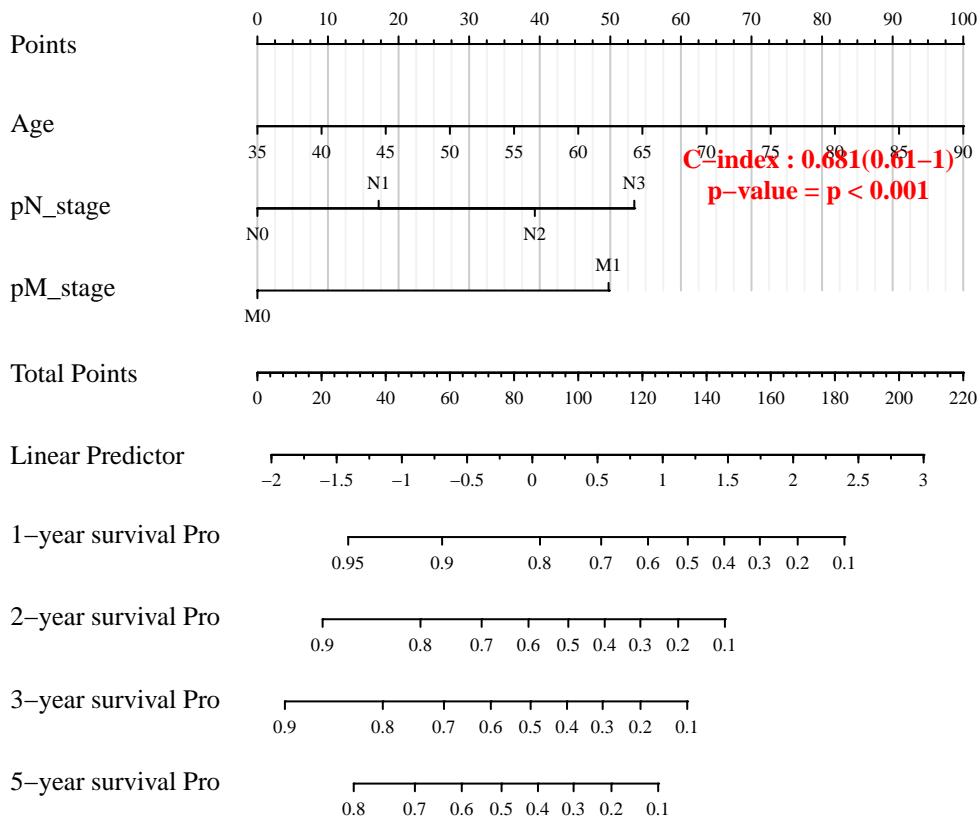

Supplement: Supplementary file 10 [file Data_Sheet_10.ZIP › Original source data of supplementary figure/Supplementary fig.4/supplementary fig4C.pdf]

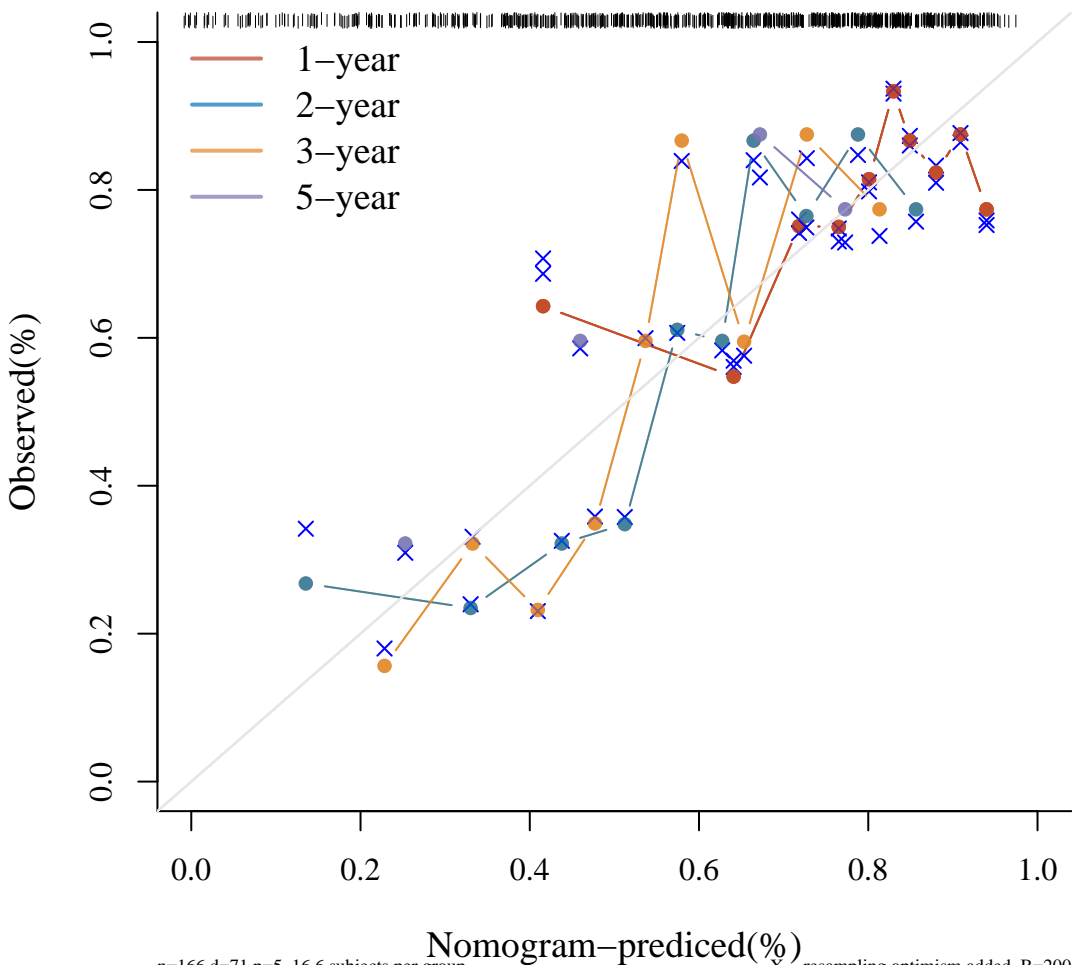

Supplement: Supplementary file 10 [file Data_Sheet_10.ZIP › Original source data of supplementary figure/Supplementary fig.4/supplementary fig4D.pdf]

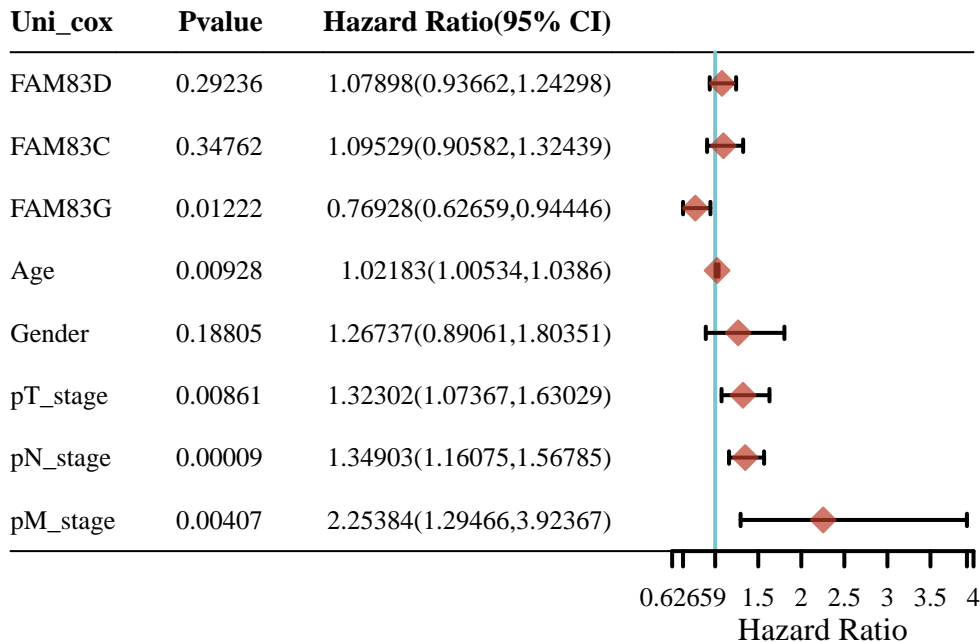

Supplement: Supplementary file 11 [file Data_Sheet_11.ZIP › Supplementary materials fig.9úa10/Supplementary materials fig.10/fig10A.pdf]

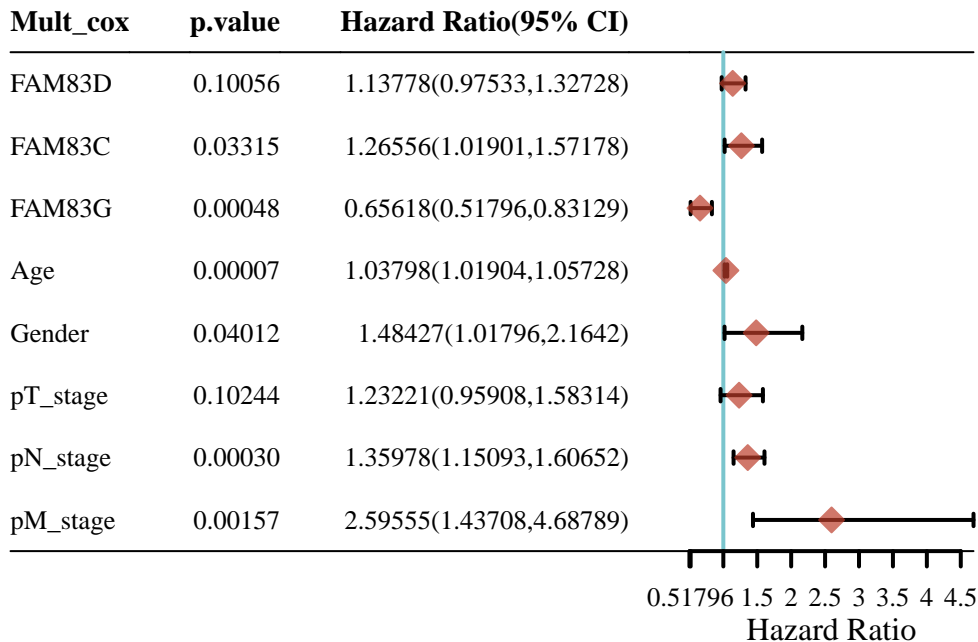

Supplement: Supplementary file 11 [file Data_Sheet_11.ZIP › Supplementary materials fig.9úa10/Supplementary materials fig.10/fig10B.pdf]

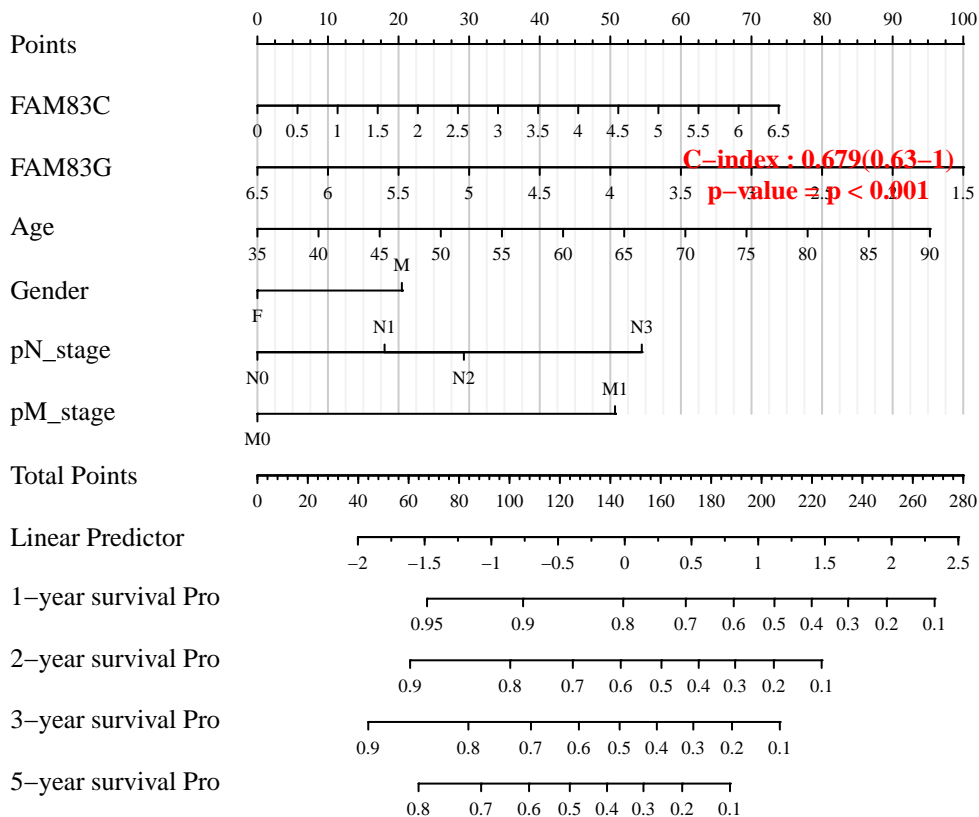

Supplement: Supplementary file 11 [file Data_Sheet_11.ZIP › Supplementary materials fig.9úa10/Supplementary materials fig.10/fig10C.pdf]

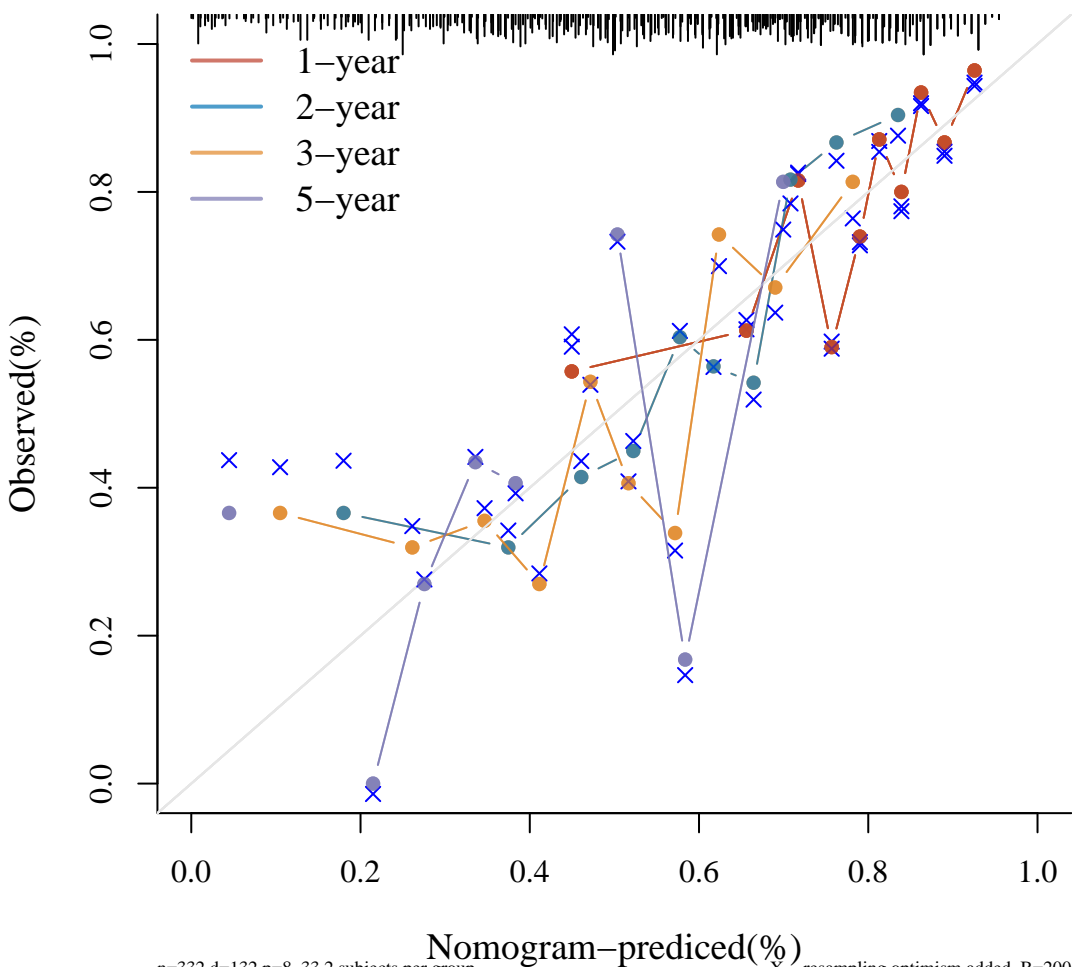

Supplement: Supplementary file 11 [file Data_Sheet_11.ZIP › Supplementary materials fig.9úa10/Supplementary materials fig.10/fig10D.pdf]

Coefficients

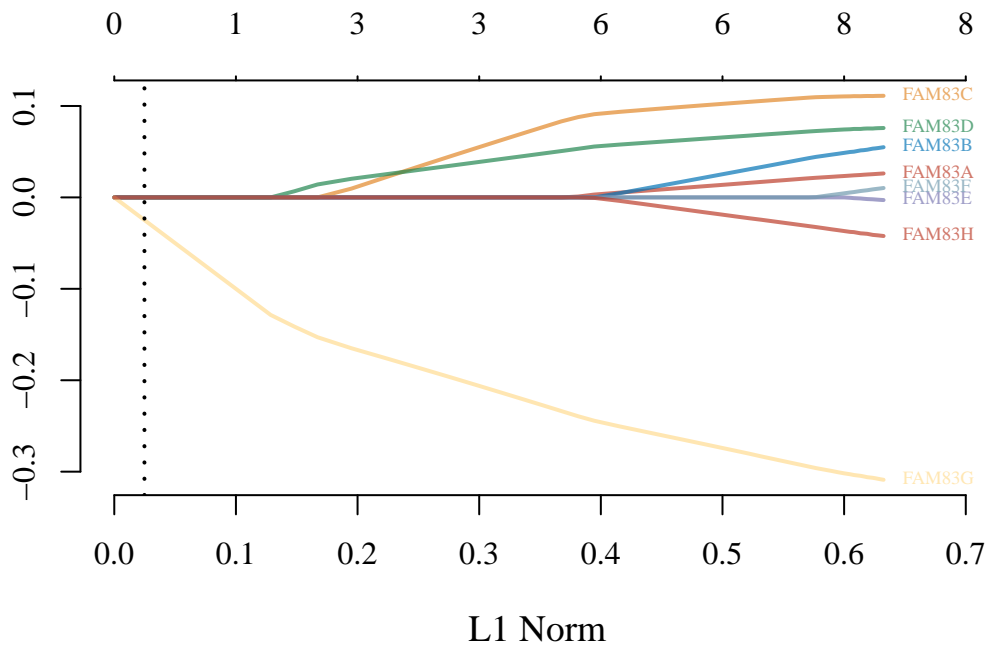

Supplement: Supplementary file 11 [file Data_Sheet_11.ZIP › Supplementary materials fig.9úa10/Supplementary materials fig.9/fig9A.pdf]

Partial Likelihood Deviance

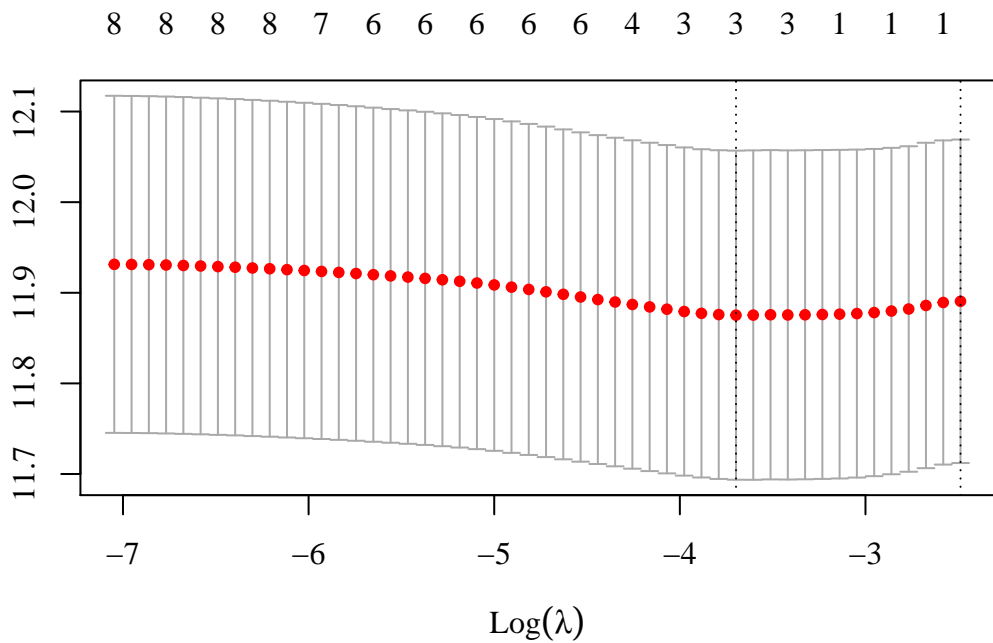

Supplement: Supplementary file 11 [file Data_Sheet_11.ZIP › Supplementary materials fig.9úa10/Supplementary materials fig.9/fig9B.pdf]

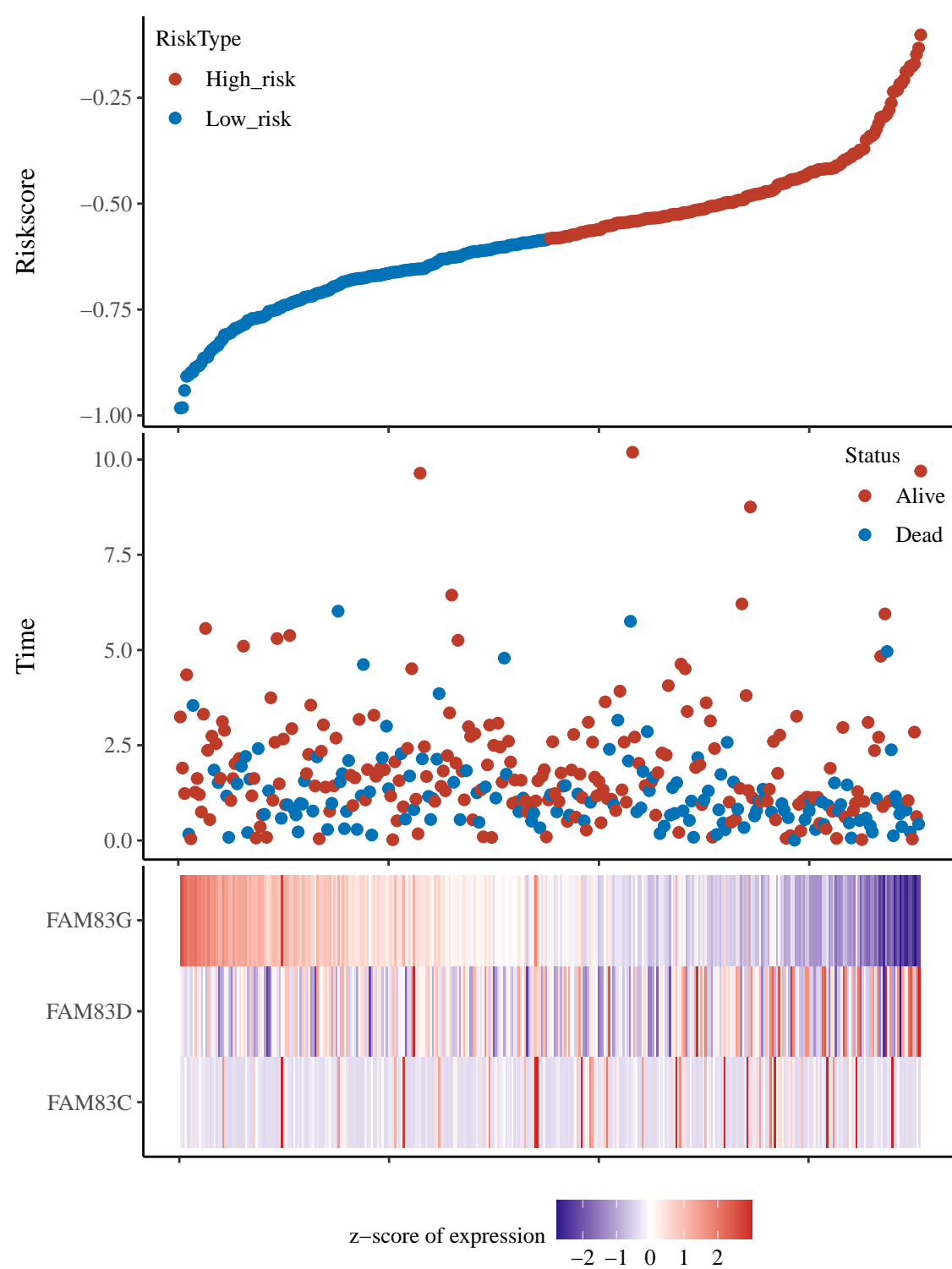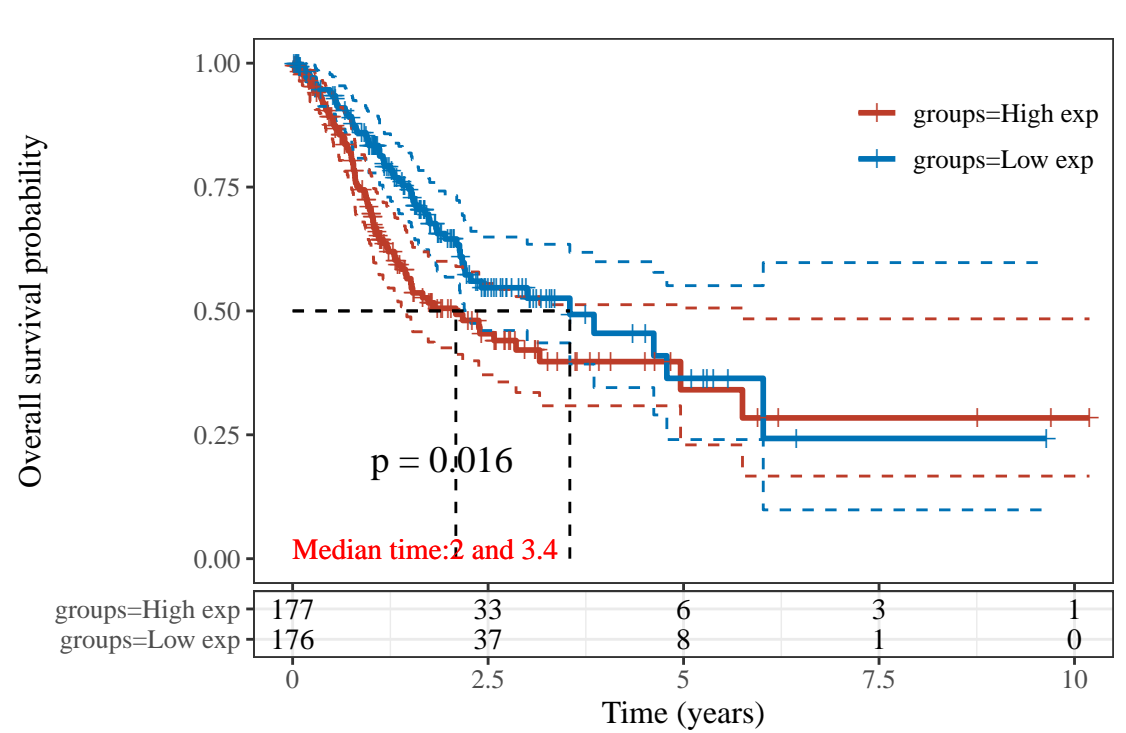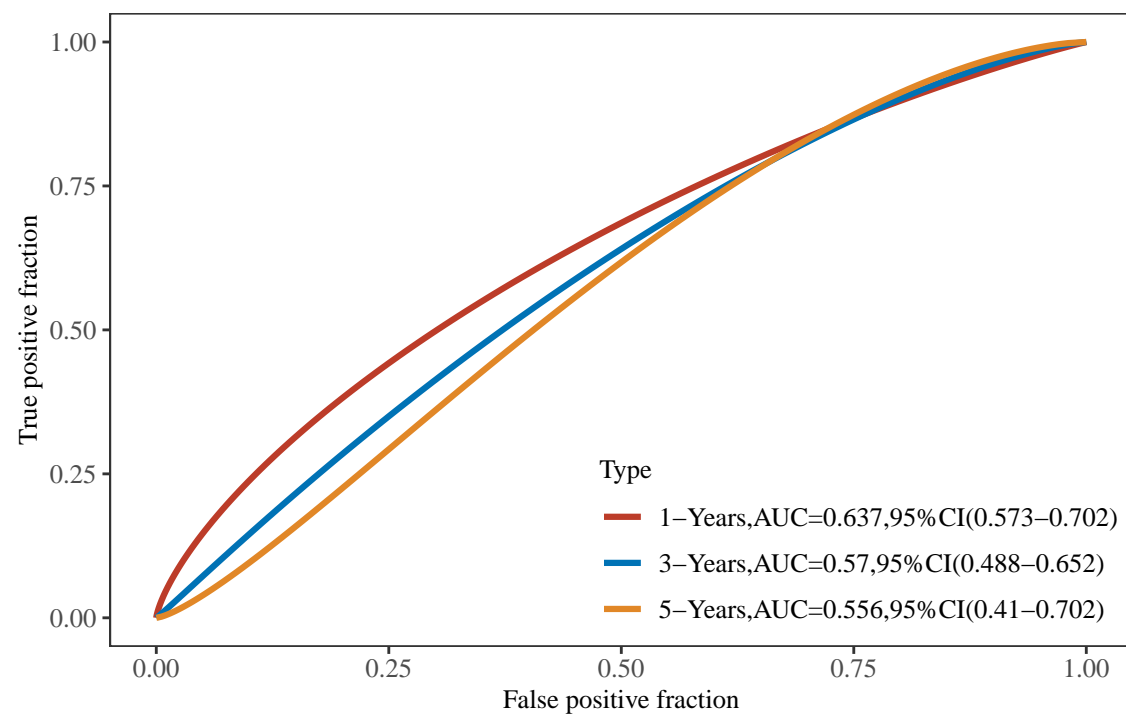

Supplement: Supplementary file 11 [file Data_Sheet_11.ZIP › Supplementary materials fig.9úa10/Supplementary materials fig.9/fig9C,D,E.pdf]

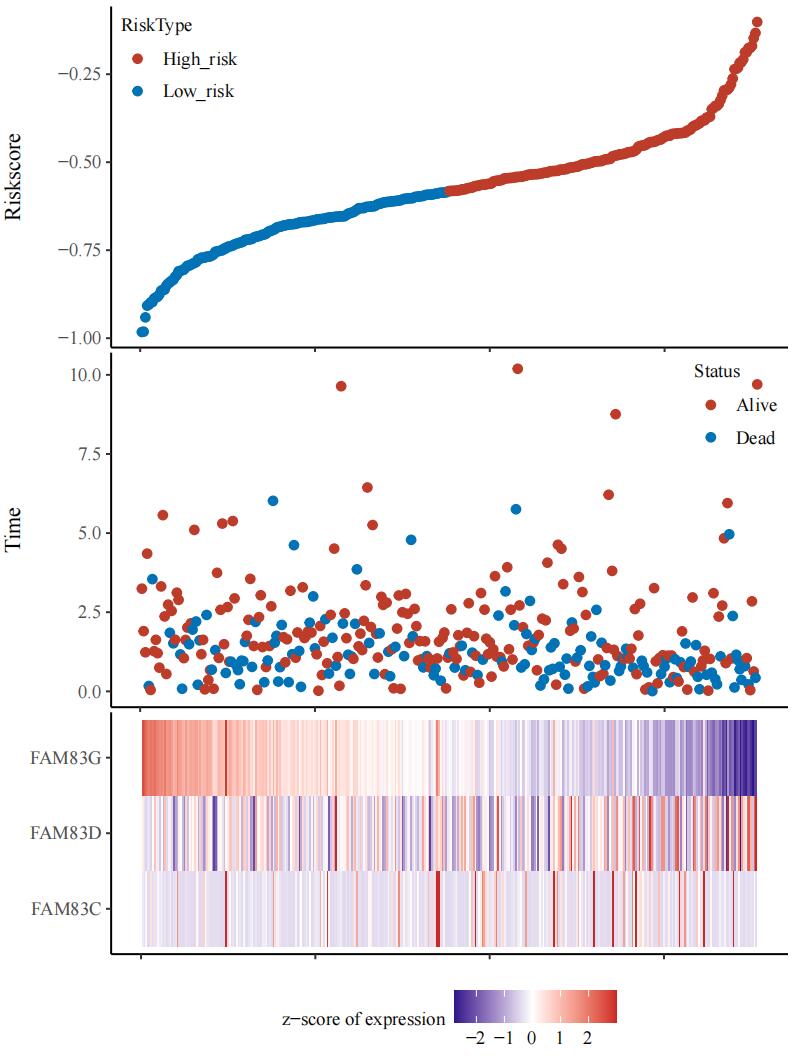

Supplement: Supplementary file 11 [file Data_Sheet_11.ZIP › Supplementary materials fig.9úa10/Supplementary materials fig.9/fig9C.jpg]

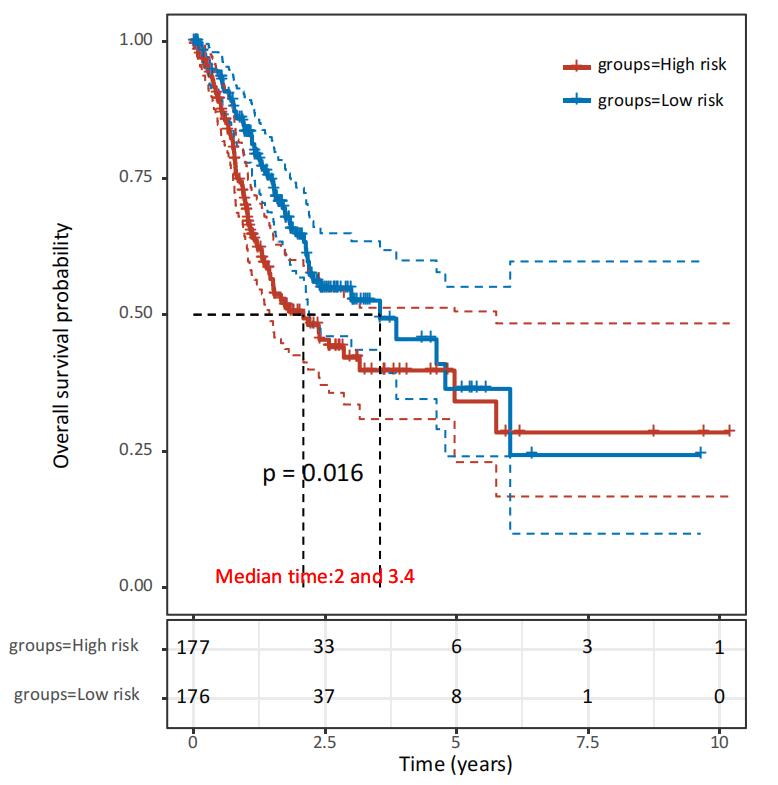

Supplement: Supplementary file 11 [file Data_Sheet_11.ZIP › Supplementary materials fig.9úa10/Supplementary materials fig.9/fig9D.jpg]

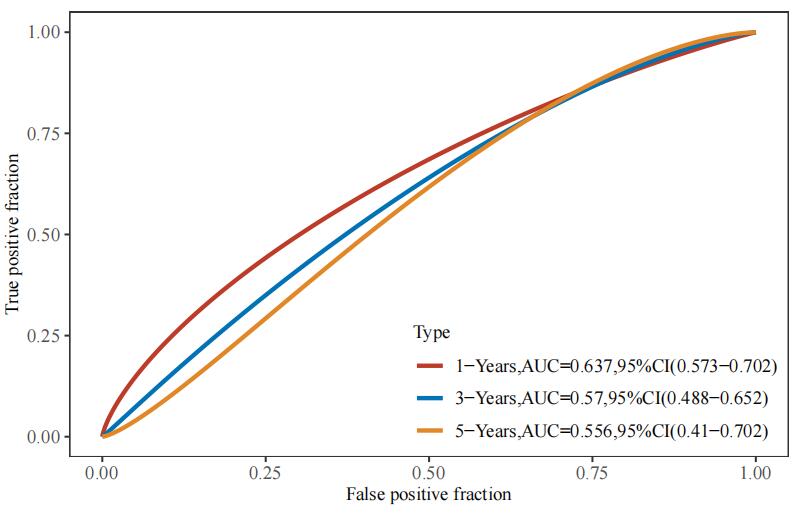

Supplement: Supplementary file 11 [file Data_Sheet_11.ZIP › Supplementary materials fig.9úa10/Supplementary materials fig.9/fig9E.jpg]

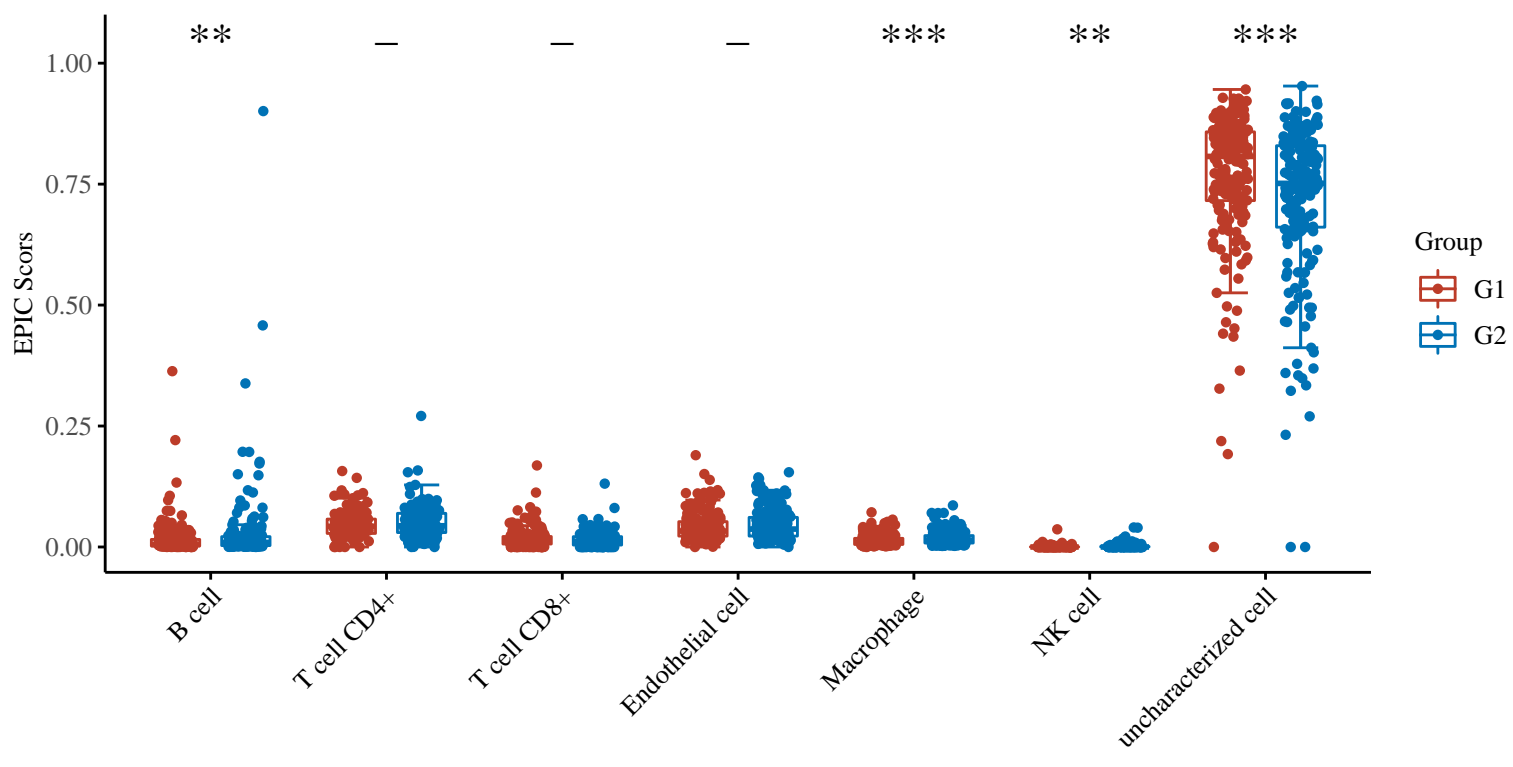

Supplement: Supplementary file 12 [file Data_Sheet_12.ZIP › Supplementary materials fig.11,12úa13/Supplementary materials fig.11/FAM83C/Box diagram/e2df2e2a-ae76-11eb-ac3b-0242ac1c0003_Immu.pdf]

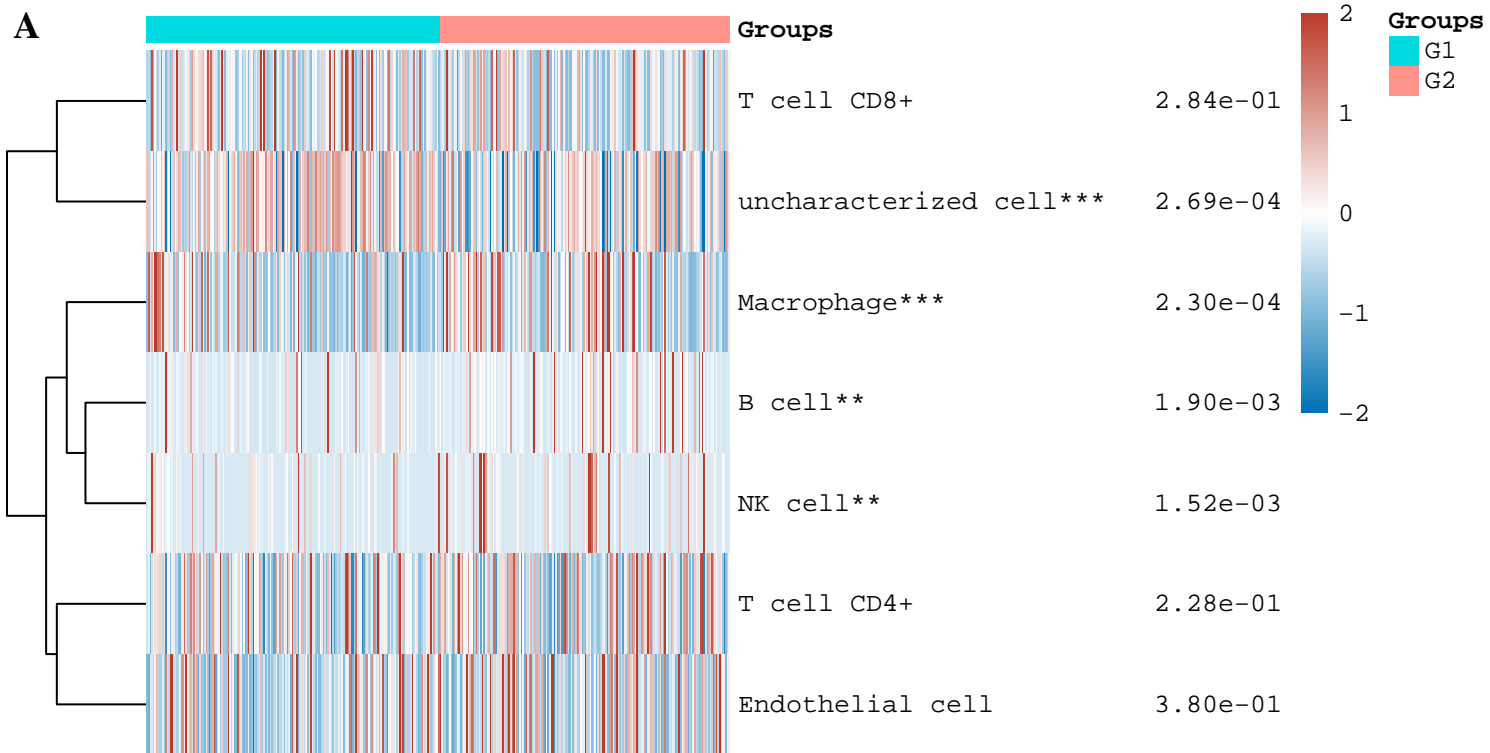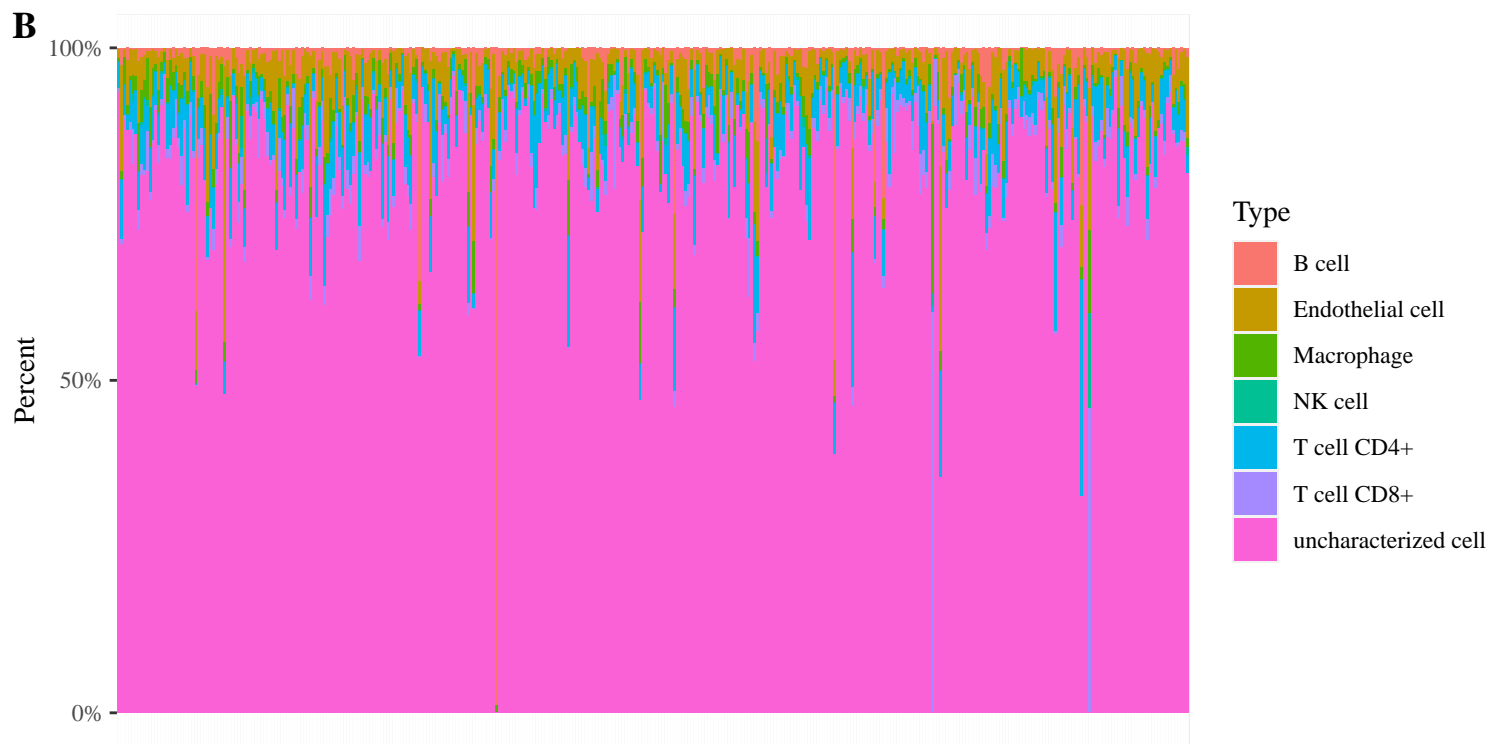

Supplement: Supplementary file 12 [file Data_Sheet_12.ZIP › Supplementary materials fig.11,12úa13/Supplementary materials fig.11/FAM83C/heatmap/f4ea74da-ae76-11eb-bf03-0242ac1c0003_Immu.pdf]

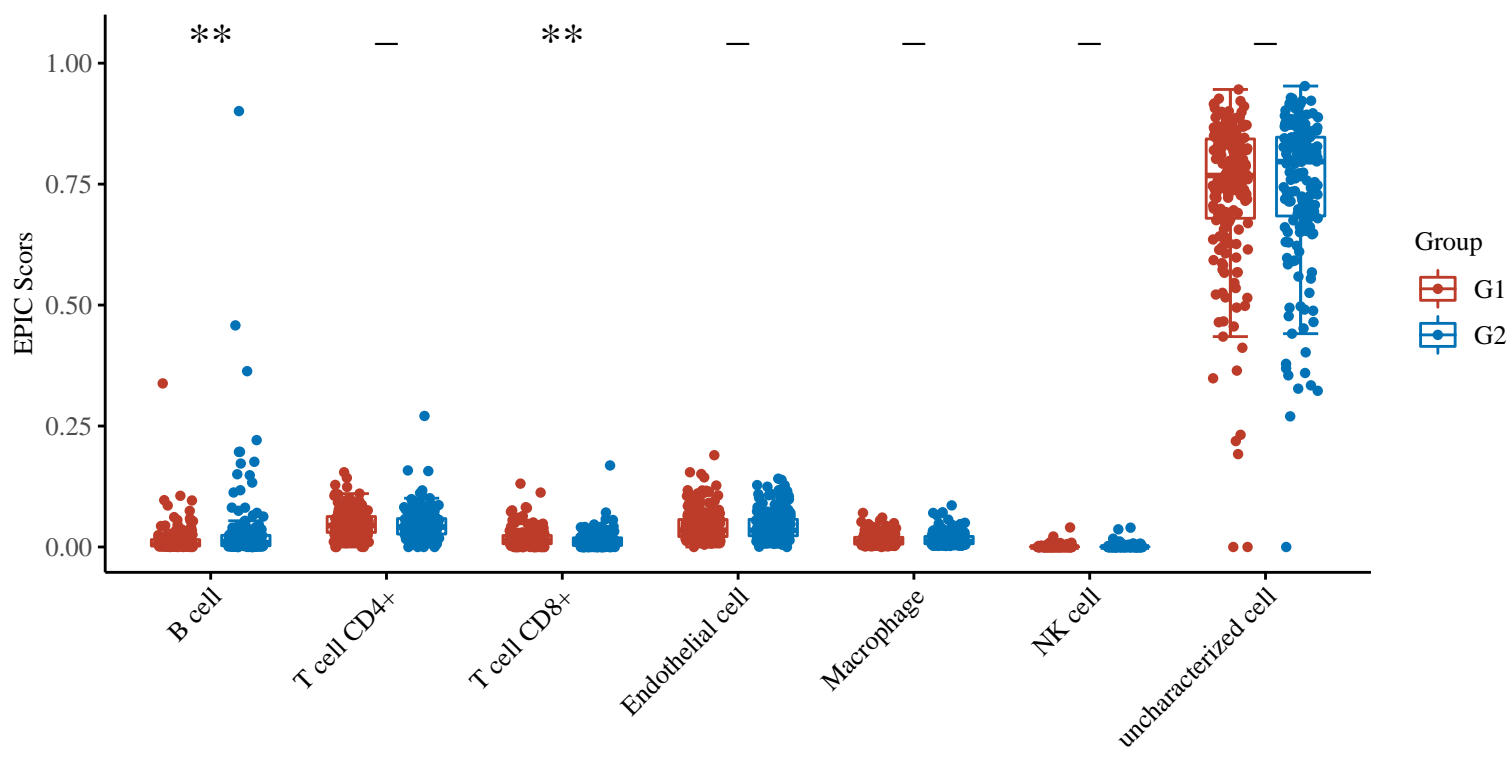

Supplement: Supplementary file 12 [file Data_Sheet_12.ZIP › Supplementary materials fig.11,12úa13/Supplementary materials fig.11/FAM83D/Box diagram/c077a6cc-ae77-11eb-aafa-0242ac1c0003_Immu.pdf]

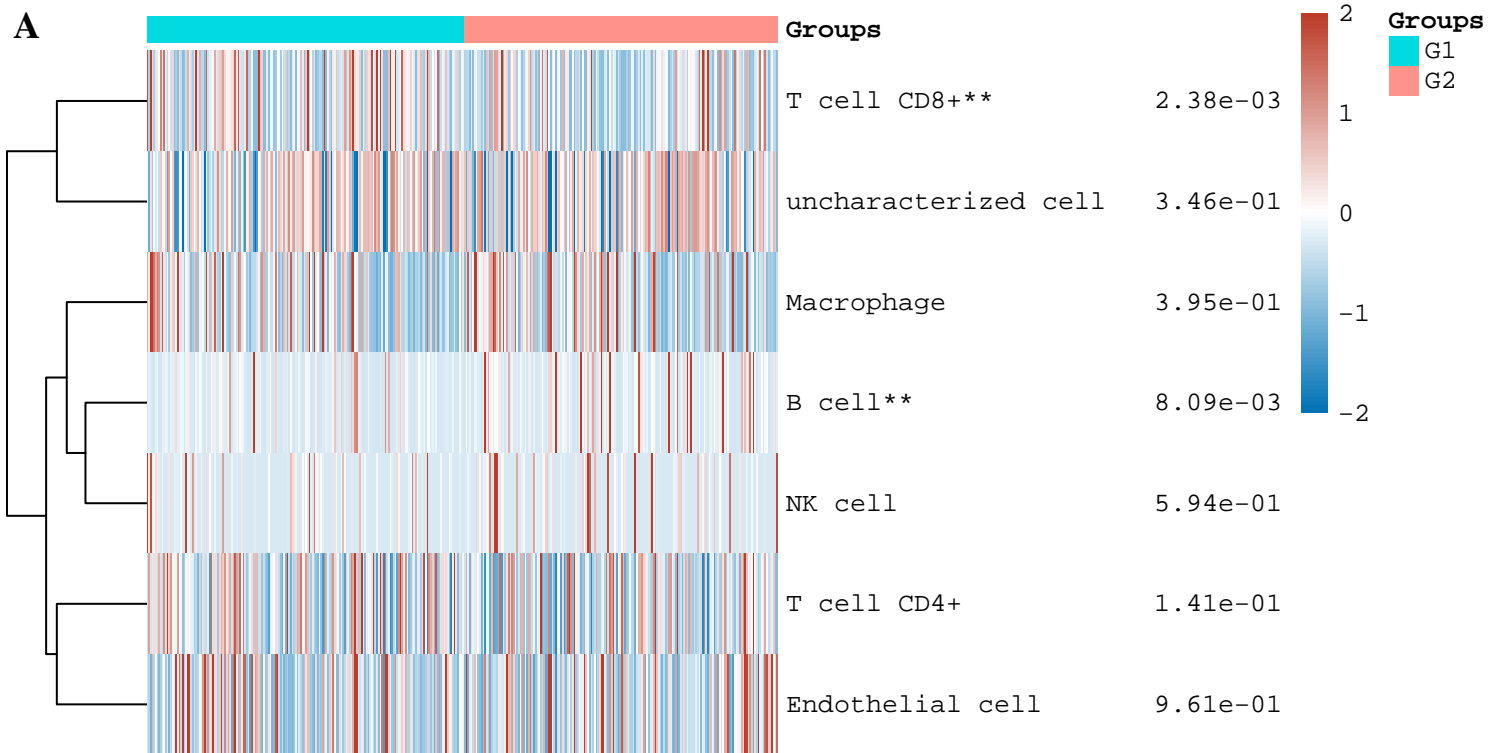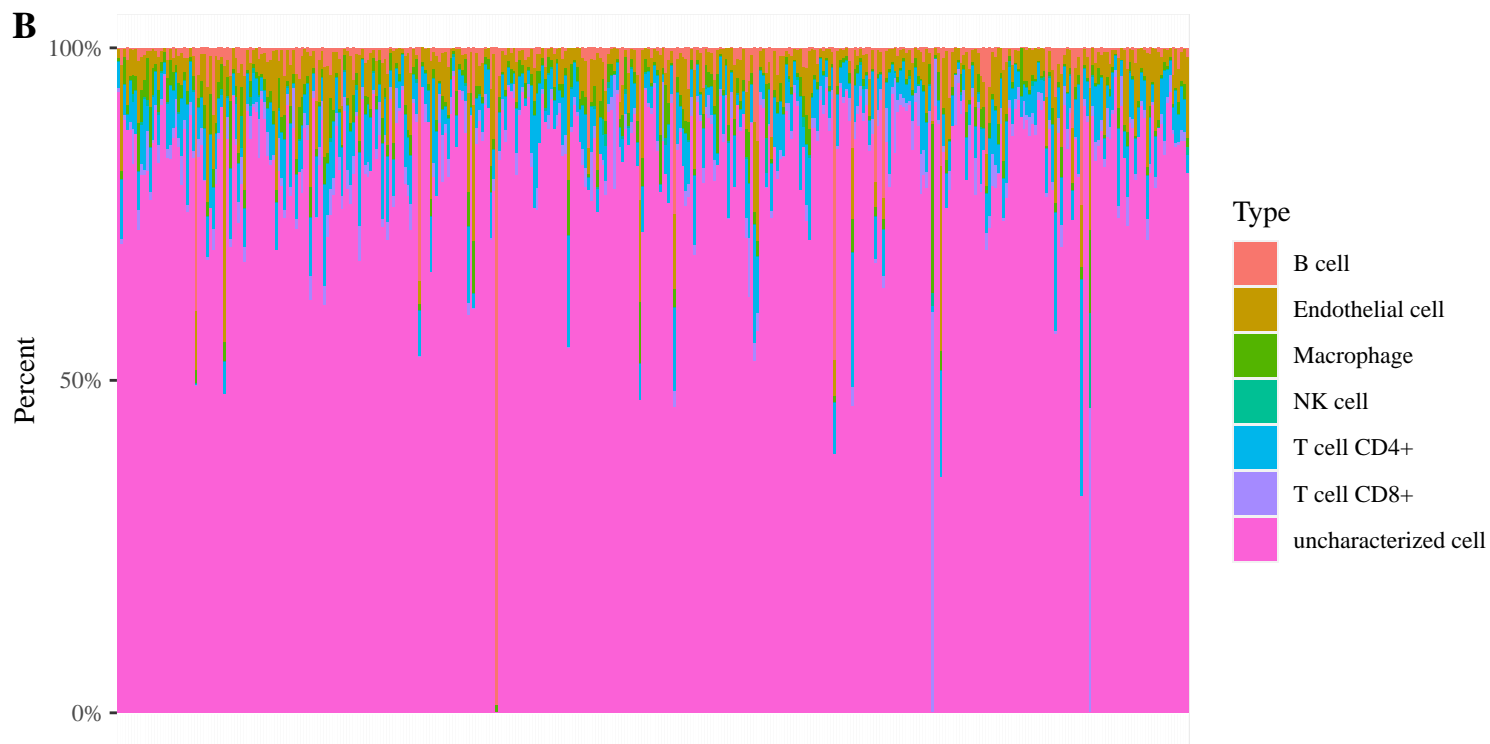

Supplement: Supplementary file 12 [file Data_Sheet_12.ZIP › Supplementary materials fig.11,12úa13/Supplementary materials fig.11/FAM83D/heatmap/7870675a-ae78-11eb-8843-0242ac1c0003_Immu.pdf]

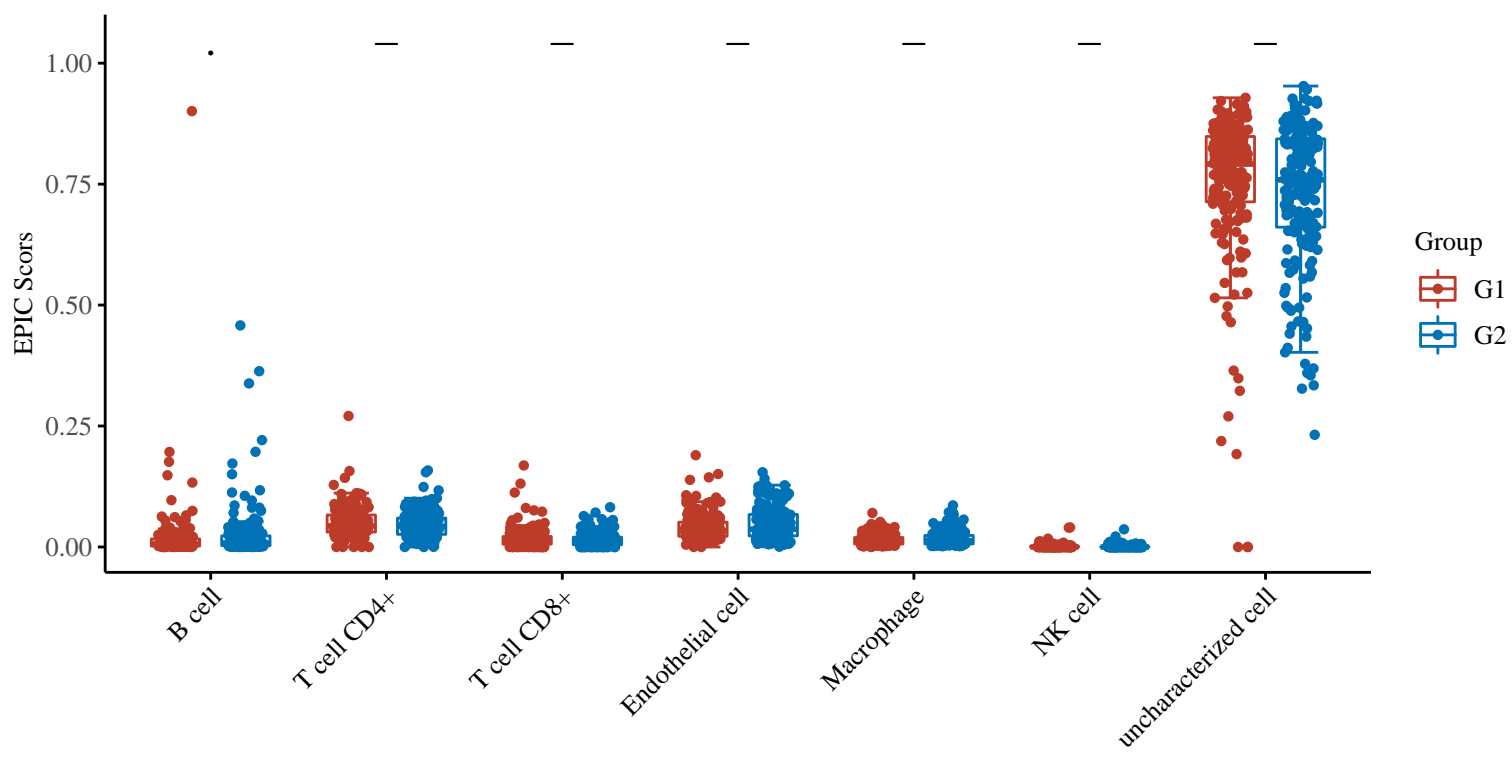

Supplement: Supplementary file 12 [file Data_Sheet_12.ZIP › Supplementary materials fig.11,12úa13/Supplementary materials fig.11/FAM83G/Box diagram/5b0f7142-ae73-11eb-9cf3-0242ac1c0003_Immu.pdf]

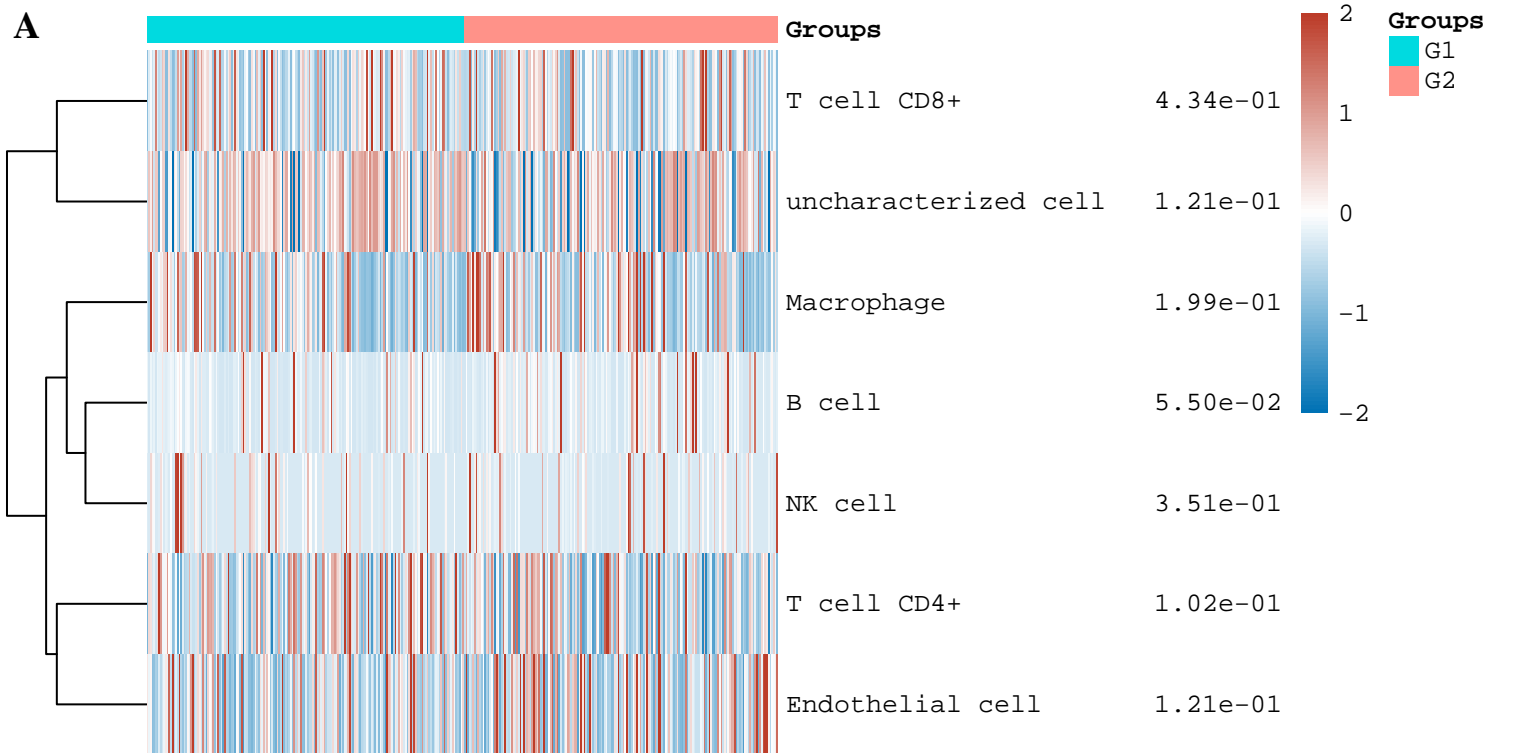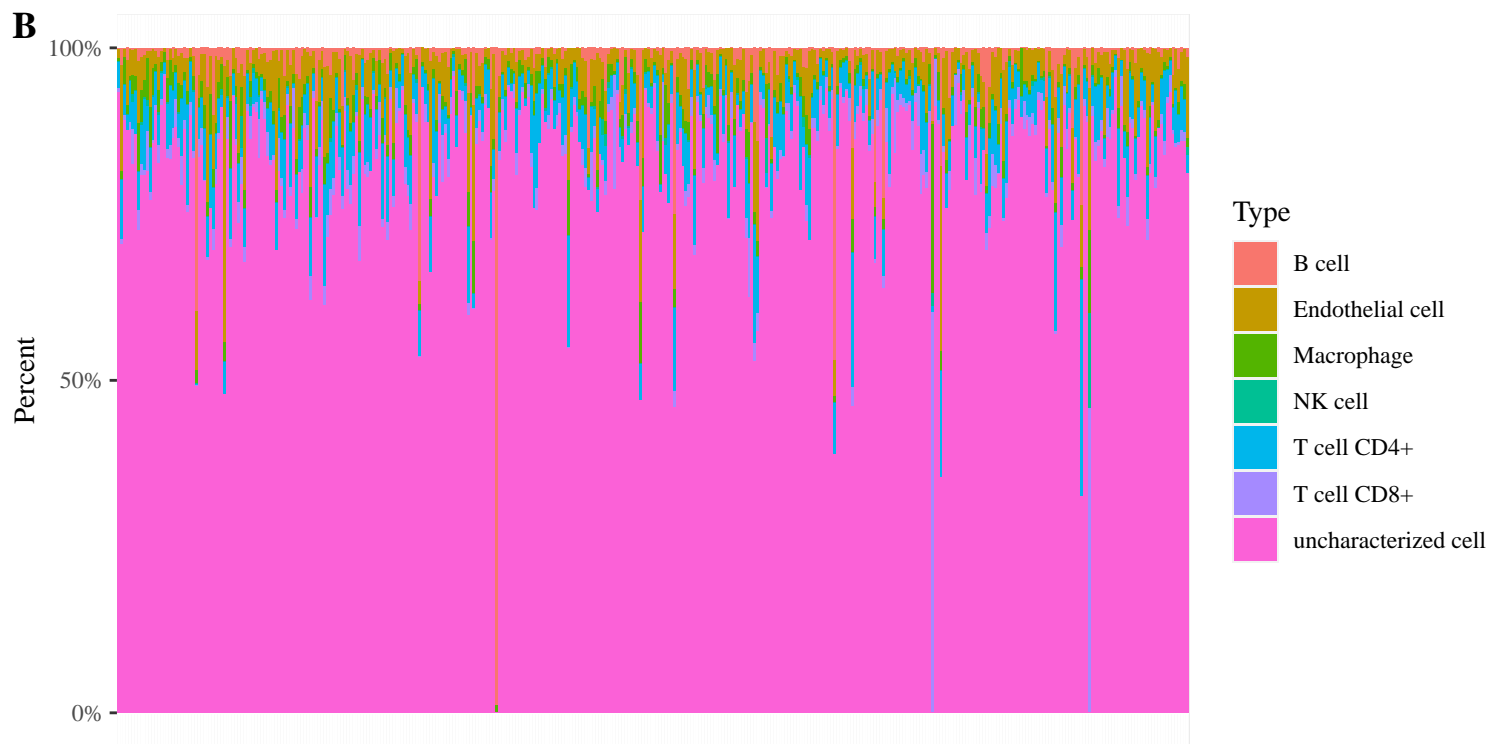

Supplement: Supplementary file 12 [file Data_Sheet_12.ZIP › Supplementary materials fig.11,12úa13/Supplementary materials fig.11/FAM83G/heatmap/44ea4c04-ae76-11eb-8e76-0242ac1c0003_Immu.pdf]

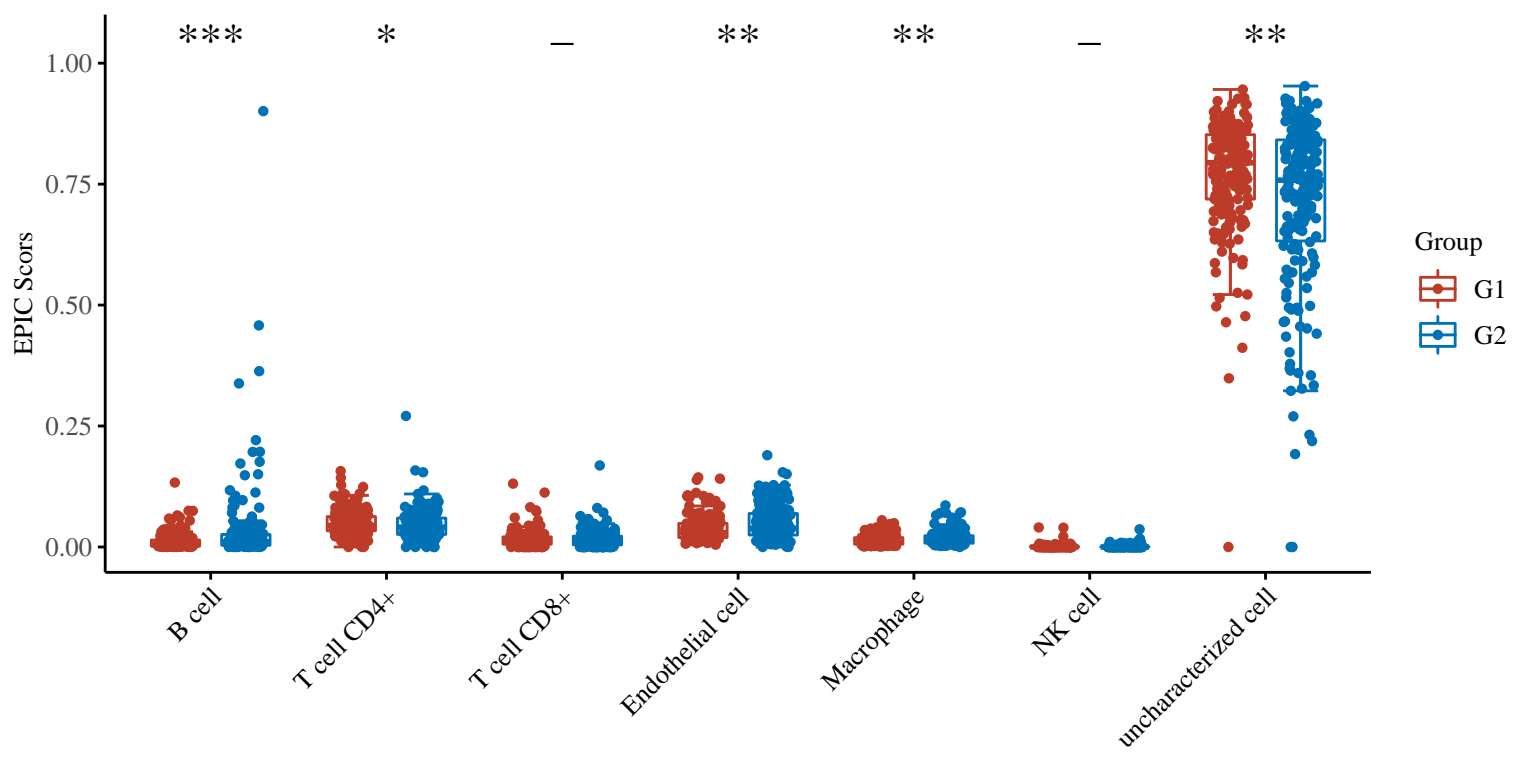

Supplement: Supplementary file 12 [file Data_Sheet_12.ZIP › Supplementary materials fig.11,12úa13/Supplementary materials fig.11/FAM83H/Box diagram/6c2d09ec-ae75-11eb-99ae-0242ac1c0003_Immu.pdf]

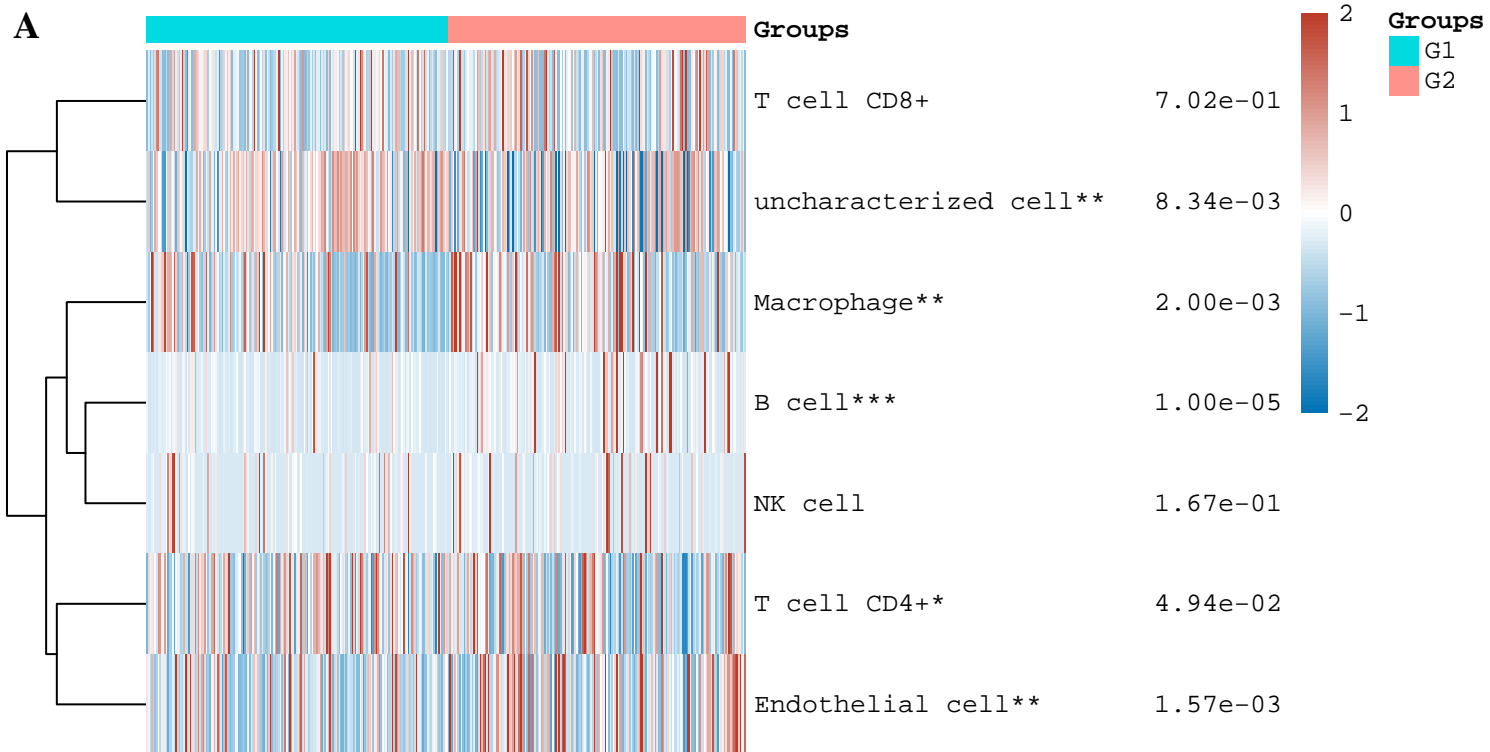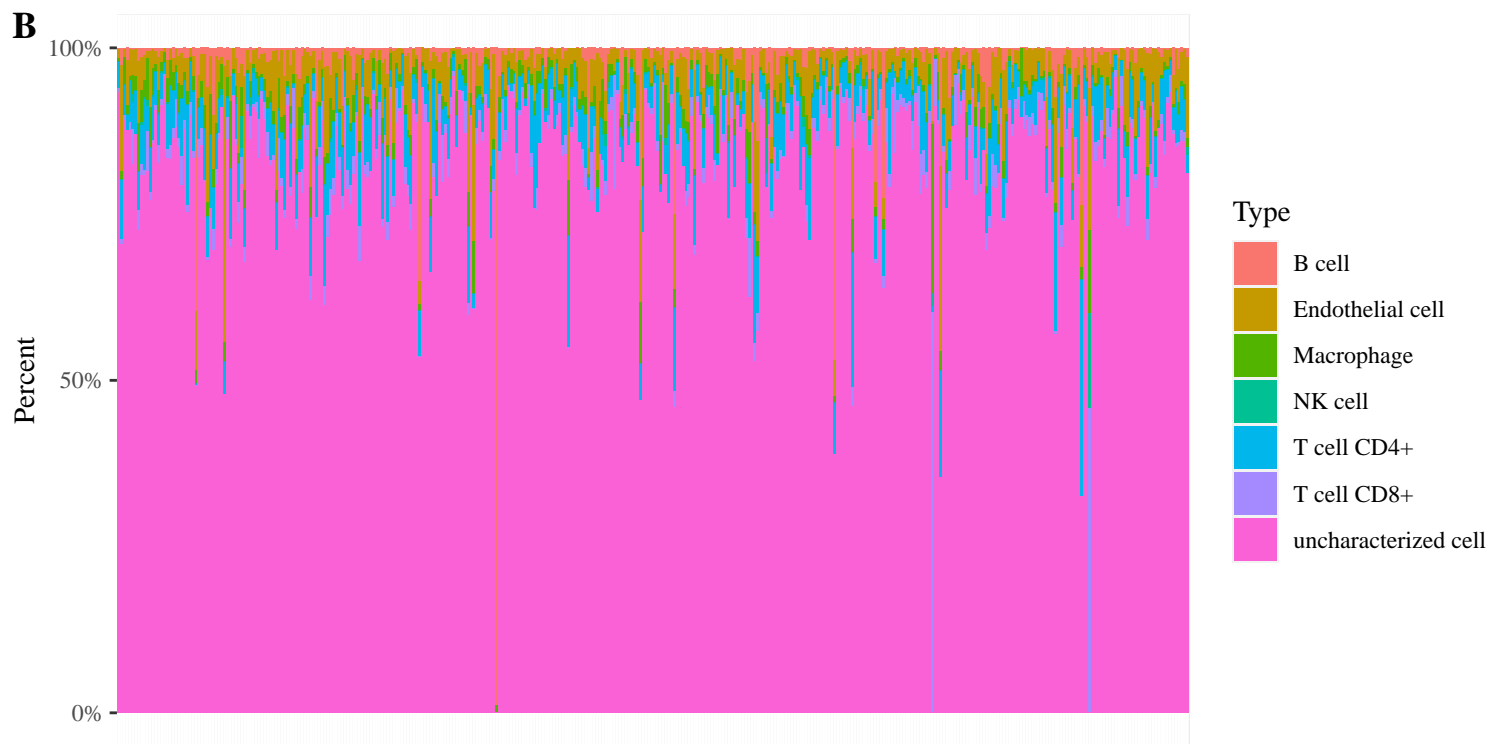

Supplement: Supplementary file 12 [file Data_Sheet_12.ZIP › Supplementary materials fig.11,12úa13/Supplementary materials fig.11/FAM83H/heatmap/2793abec-ae75-11eb-9ee7-0242ac1c0003_Immu.pdf]

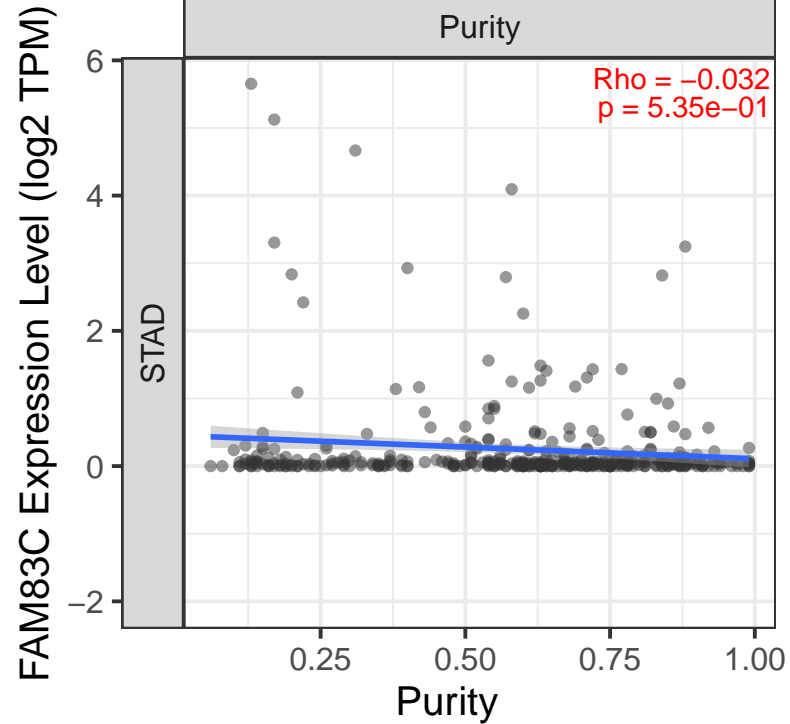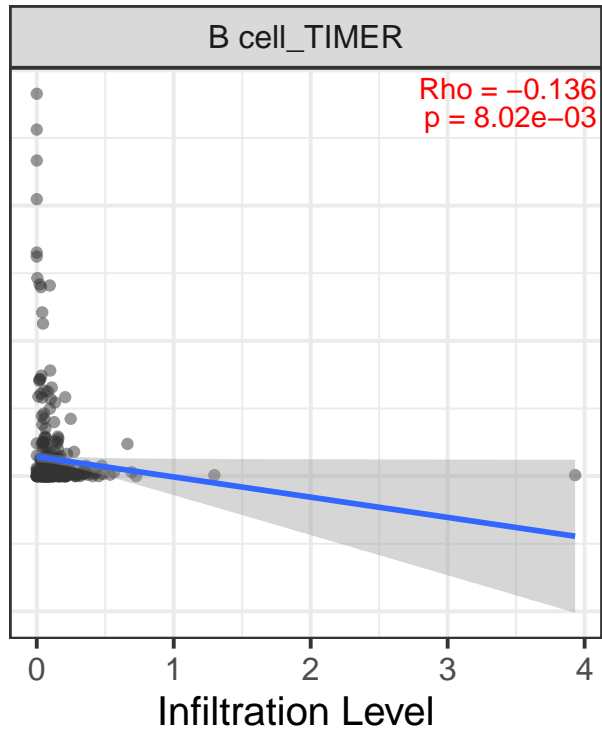

Supplement: Supplementary file 12 [file Data_Sheet_12.ZIP › Supplementary materials fig.11,12úa13/Supplementary materials fig.12/FAM83C/gene_plot(1).pdf]

FAM83D Expression Level (log2 TPM)

STAD

Purity

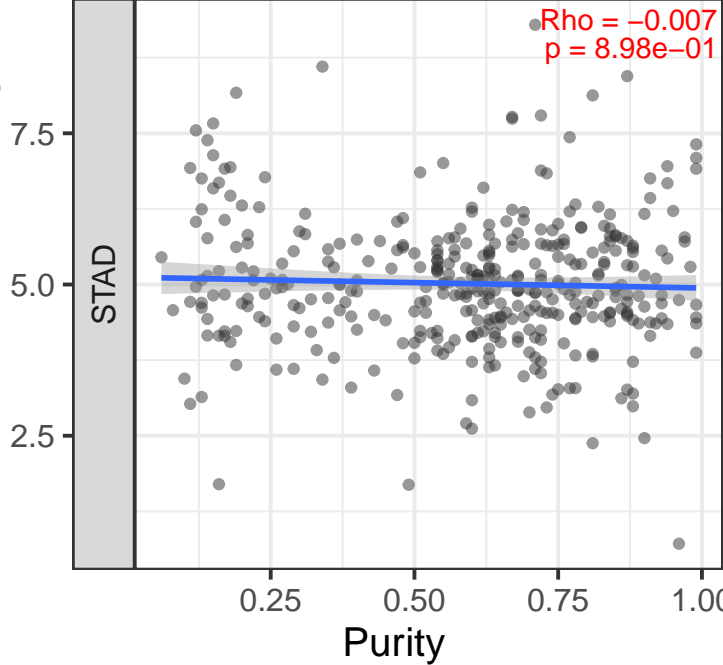

Macrophage\_TIMER

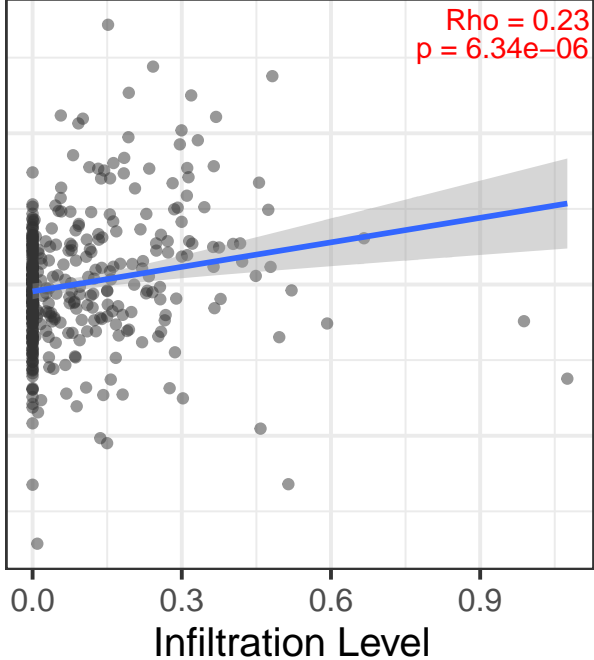

Supplement: Supplementary file 12 [file Data_Sheet_12.ZIP › Supplementary materials fig.11,12úa13/Supplementary materials fig.12/FAM83D/gene_plot(1).pdf]

FAM83D Expression Level (log2 TPM)

STAD

Purity

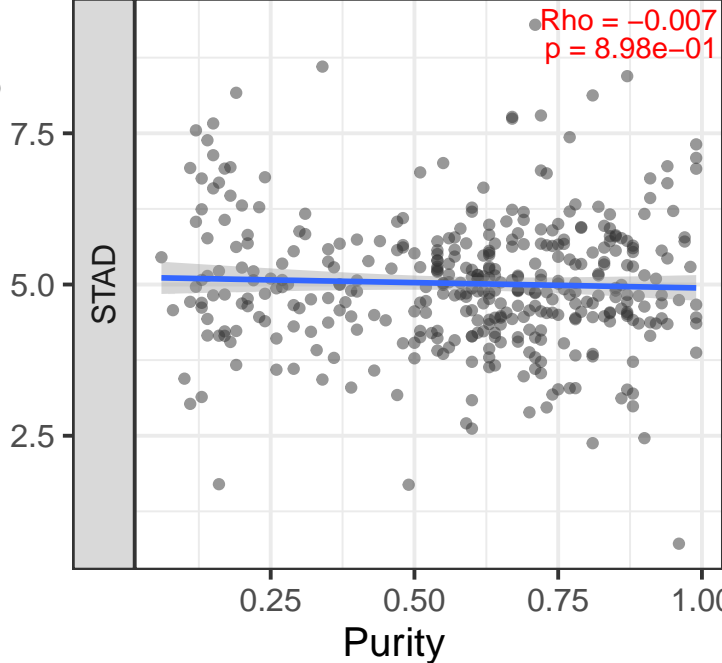

Myeloid dendritic cell\_TIMER

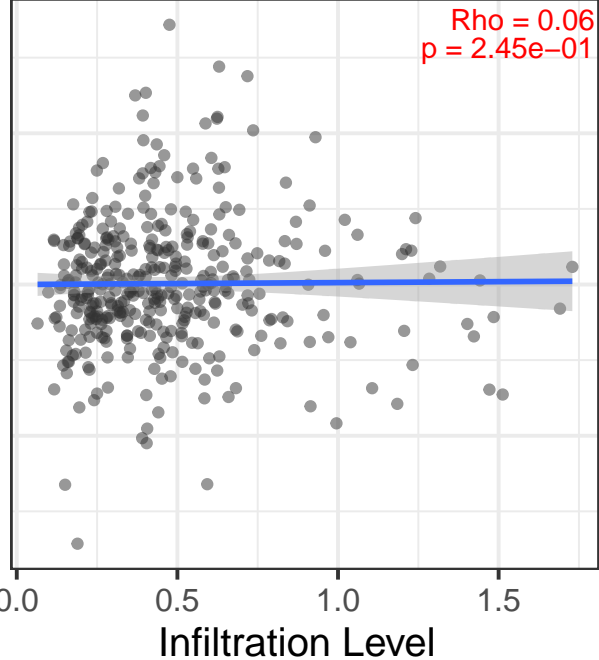

Supplement: Supplementary file 12 [file Data_Sheet_12.ZIP › Supplementary materials fig.11,12úa13/Supplementary materials fig.12/FAM83D/gene_plot(2).pdf]

FAM83D Expression Level (log2 TPM)

STAD

Purity

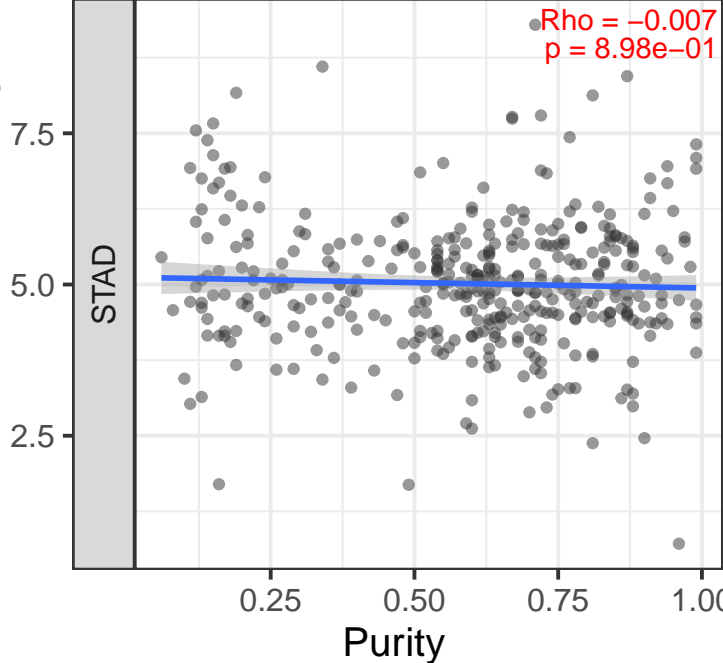

T cell CD4+\_TIMER

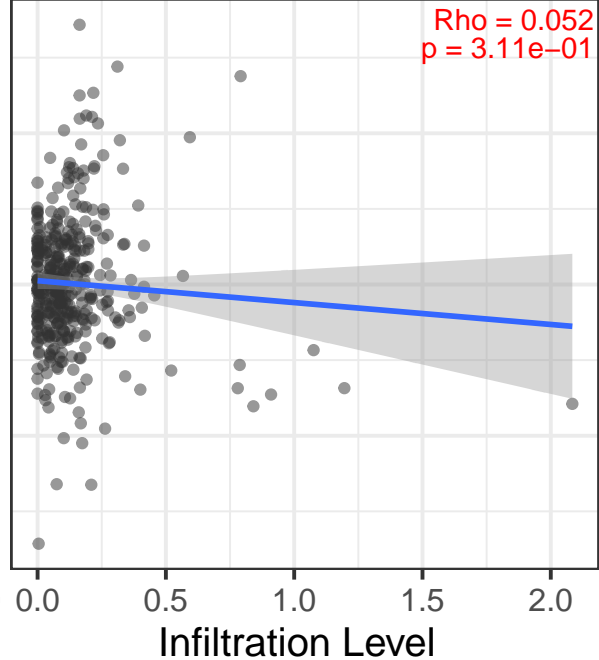

Supplement: Supplementary file 12 [file Data_Sheet_12.ZIP › Supplementary materials fig.11,12úa13/Supplementary materials fig.12/FAM83D/gene_plot(3).pdf]

FAM83D Expression Level (log2 TPM)

STAD

Purity

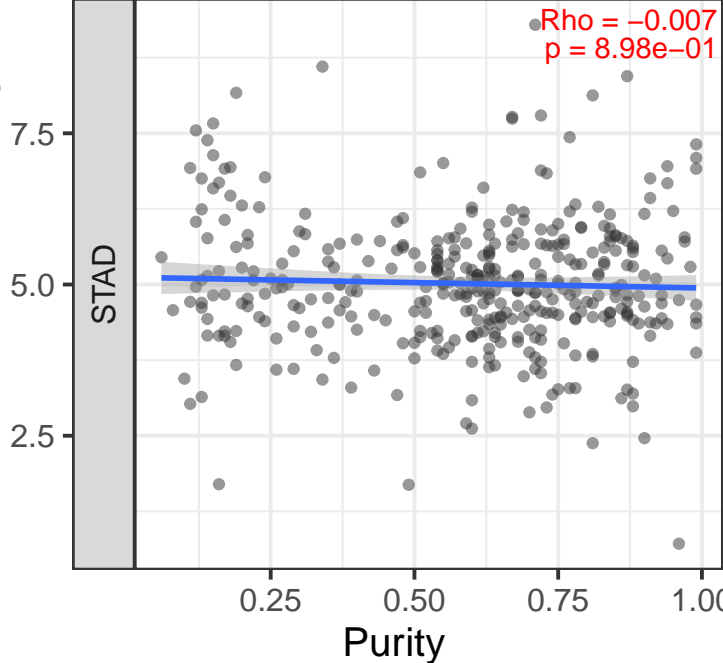

T cell CD8+\_TIMER

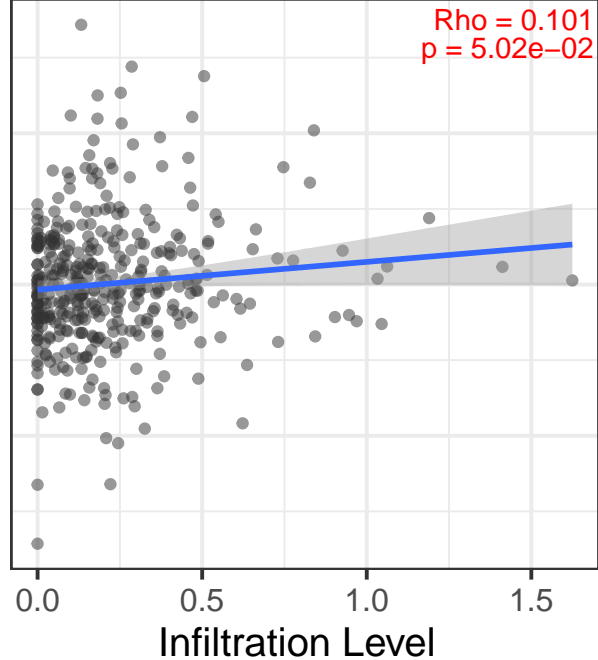

Supplement: Supplementary file 12 [file Data_Sheet_12.ZIP › Supplementary materials fig.11,12úa13/Supplementary materials fig.12/FAM83D/gene_plot(4).pdf]

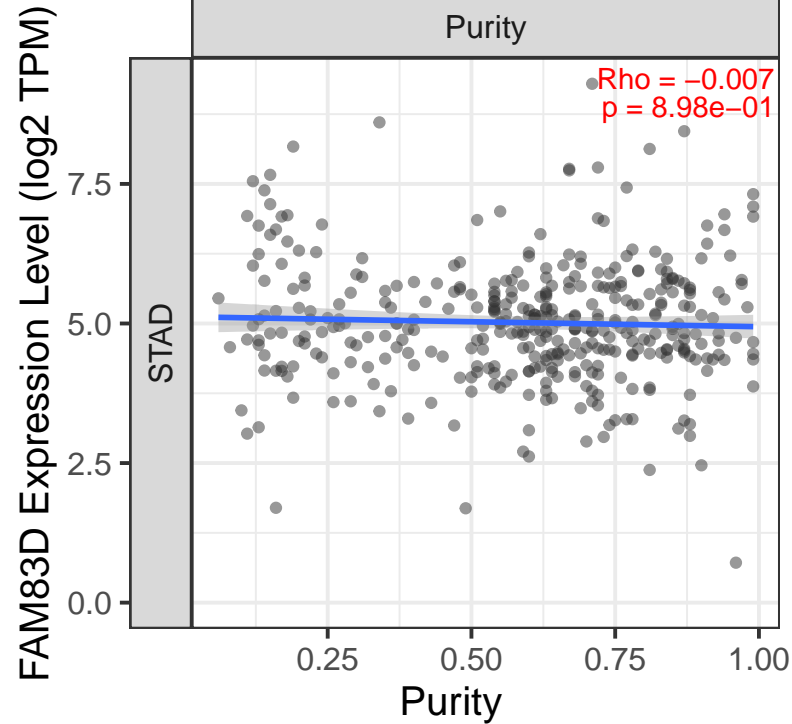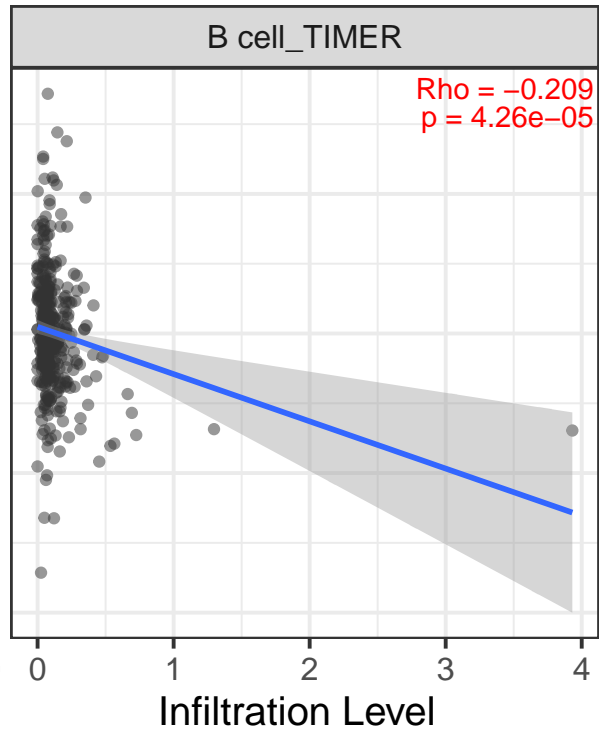

Supplement: Supplementary file 12 [file Data_Sheet_12.ZIP › Supplementary materials fig.11,12úa13/Supplementary materials fig.12/FAM83D/gene_plot.pdf]

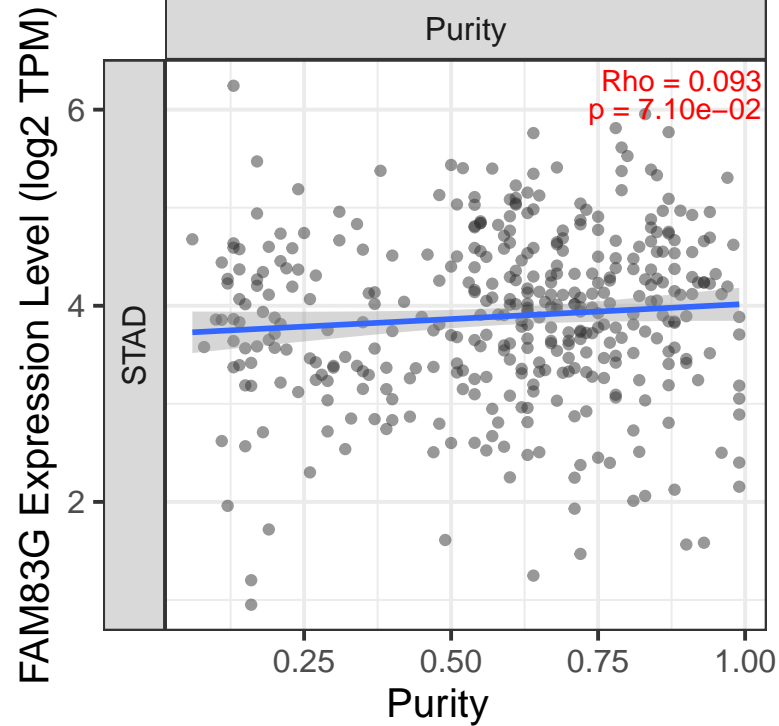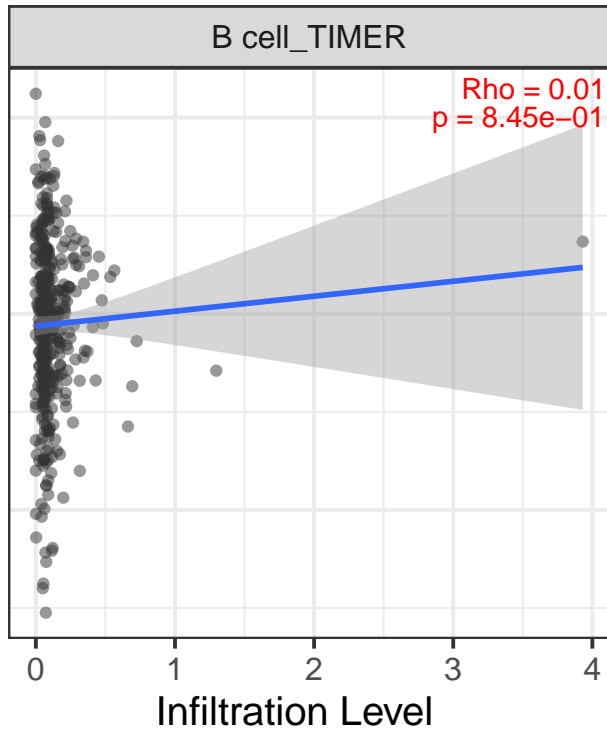

Supplement: Supplementary file 12 [file Data_Sheet_12.ZIP › Supplementary materials fig.11,12úa13/Supplementary materials fig.12/FAM83G/gene_plot(2).pdf]

FAM83H Expression Level (log2 TPM)

STAD

Purity

$Rho = 0.128$   
 $p = 1.24e-02$

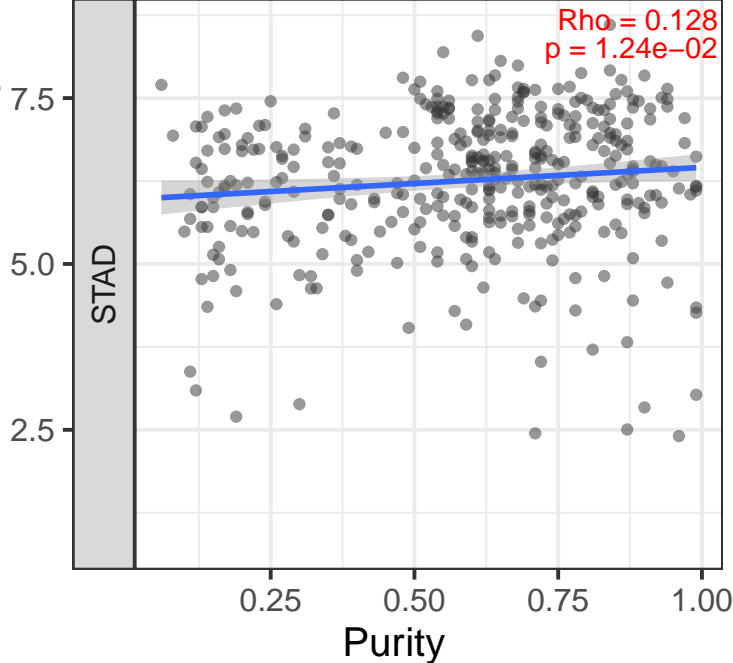

B cell\_TIMER

$Rho = -0.076$   
 $p = 1.42e-01$

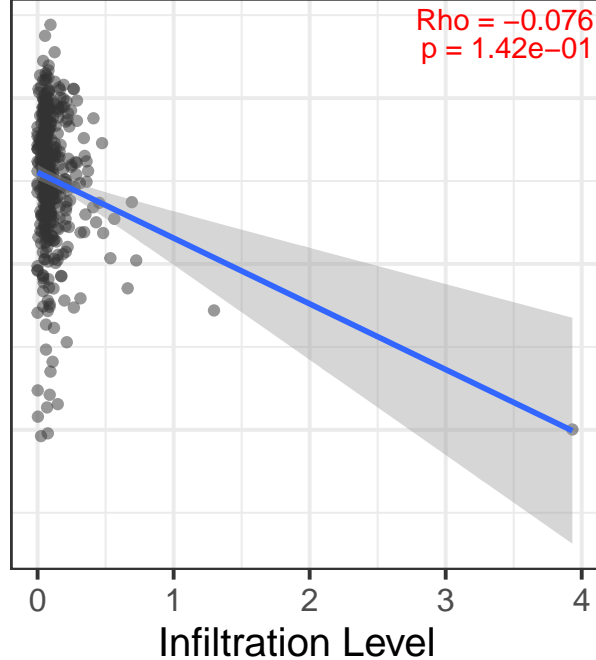

Supplement: Supplementary file 12 [file Data_Sheet_12.ZIP › Supplementary materials fig.11,12úa13/Supplementary materials fig.12/FAM83H/gene_plot(2).pdf]

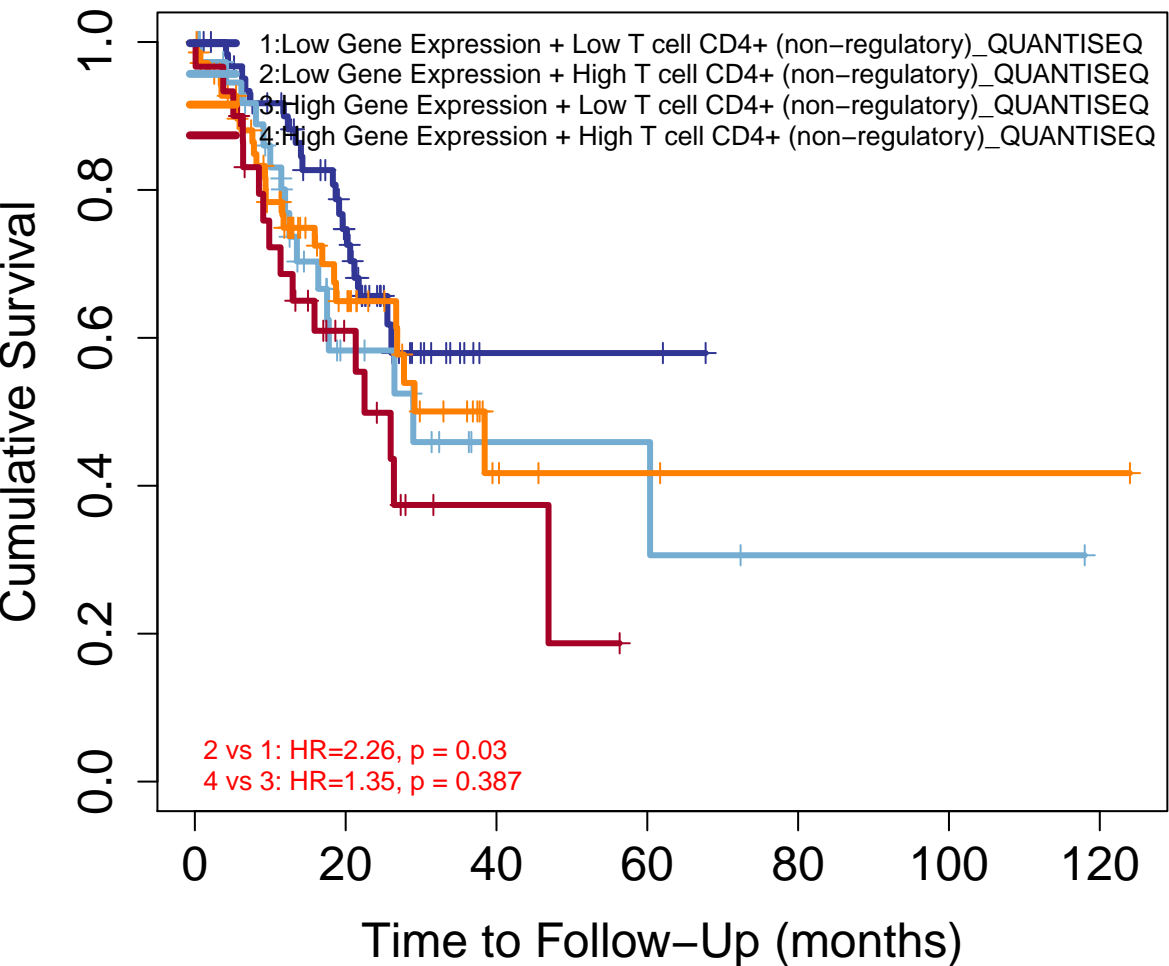

Supplement: Supplementary file 12 [file Data_Sheet_12.ZIP › Supplementary materials fig.11,12úa13/Supplementary materials fig.13/FAM83C/CD4+T/outcome_plot.pdf]

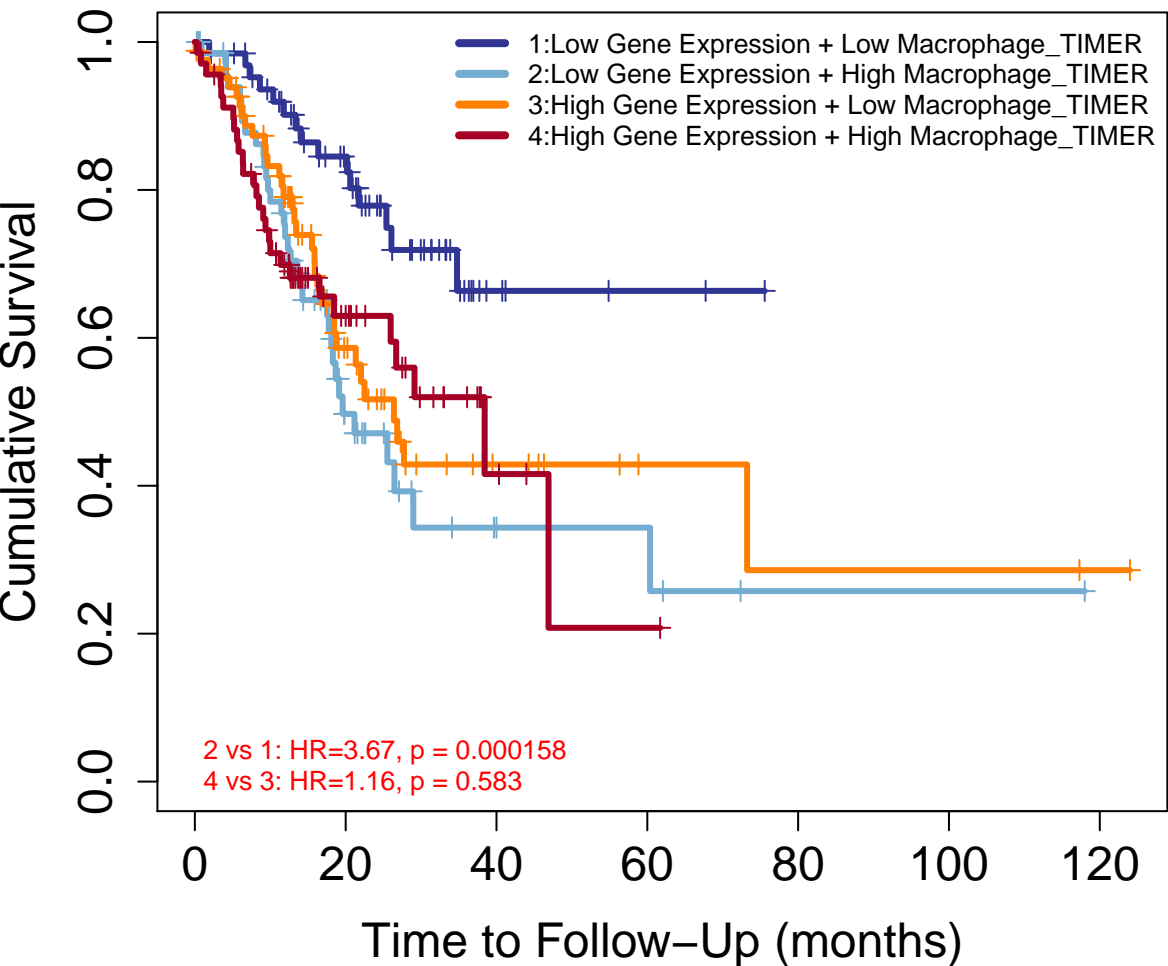

Supplement: Supplementary file 12 [file Data_Sheet_12.ZIP › Supplementary materials fig.11,12úa13/Supplementary materials fig.13/FAM83C/macrophage/outcome_plot.pdf]

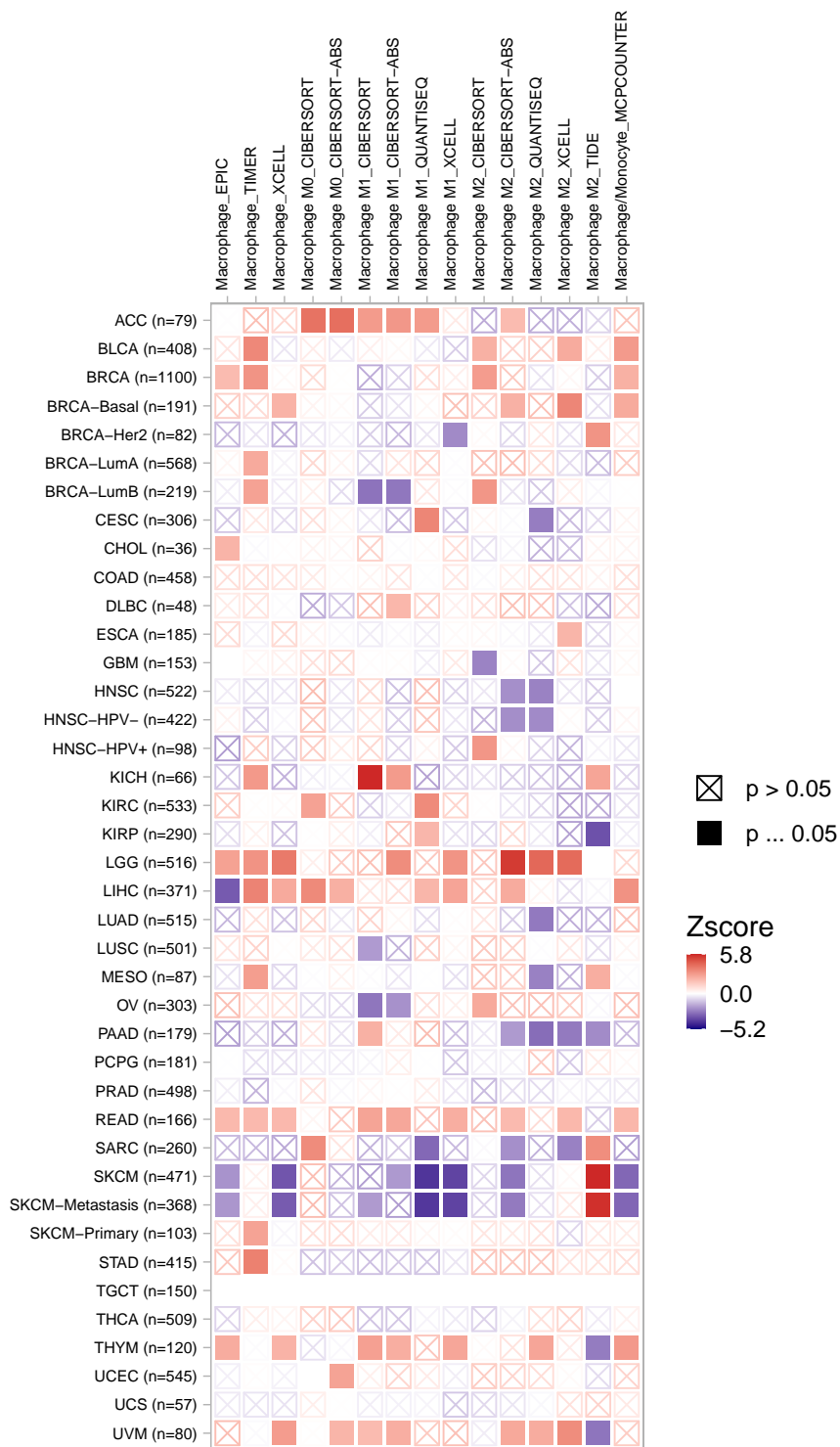

Supplement: Supplementary file 12 [file Data_Sheet_12.ZIP › Supplementary materials fig.11,12úa13/Supplementary materials fig.13/FAM83C/macrophage/outcome_table.pdf]

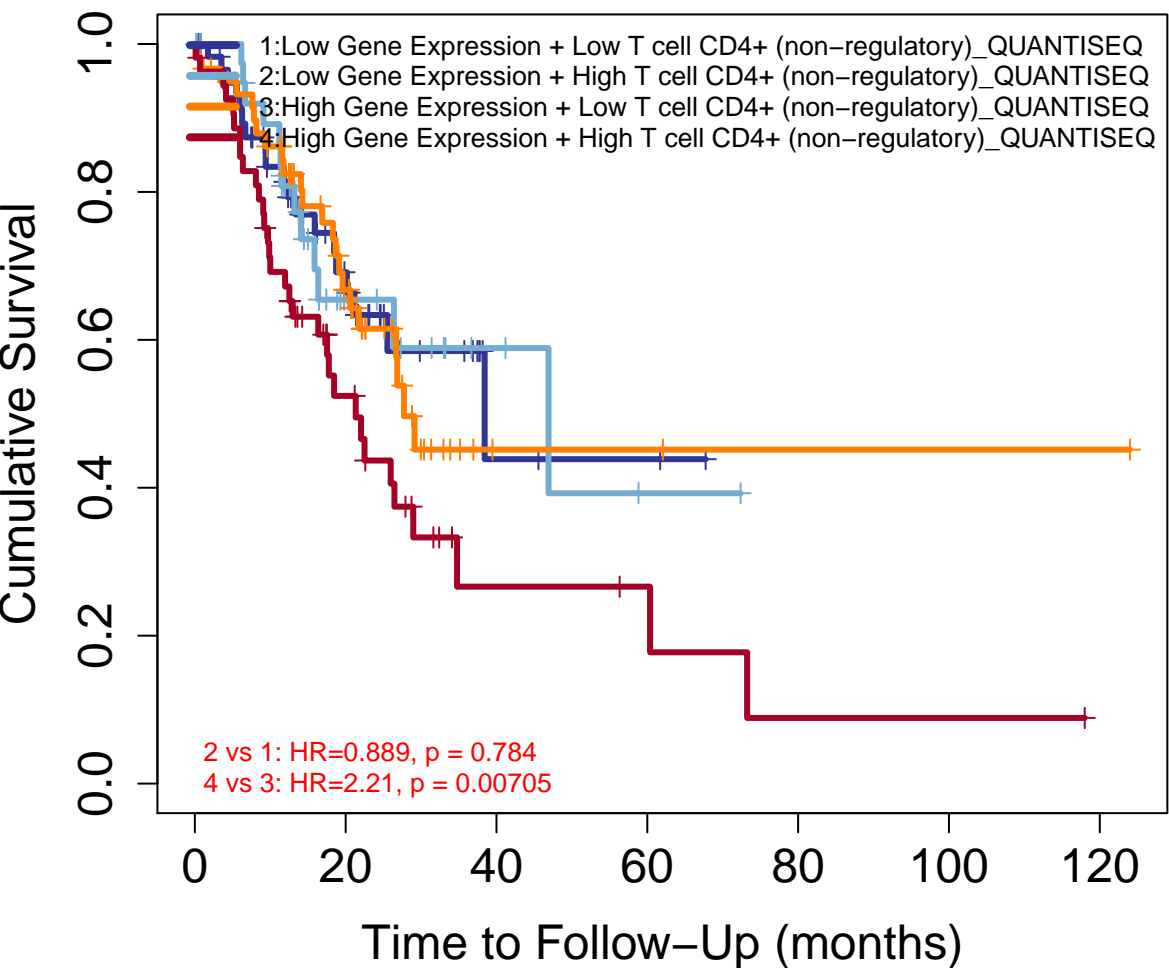

Supplement: Supplementary file 12 [file Data_Sheet_12.ZIP › Supplementary materials fig.11,12úa13/Supplementary materials fig.13/FAM83D/CD4+T/outcome_plot.pdf]

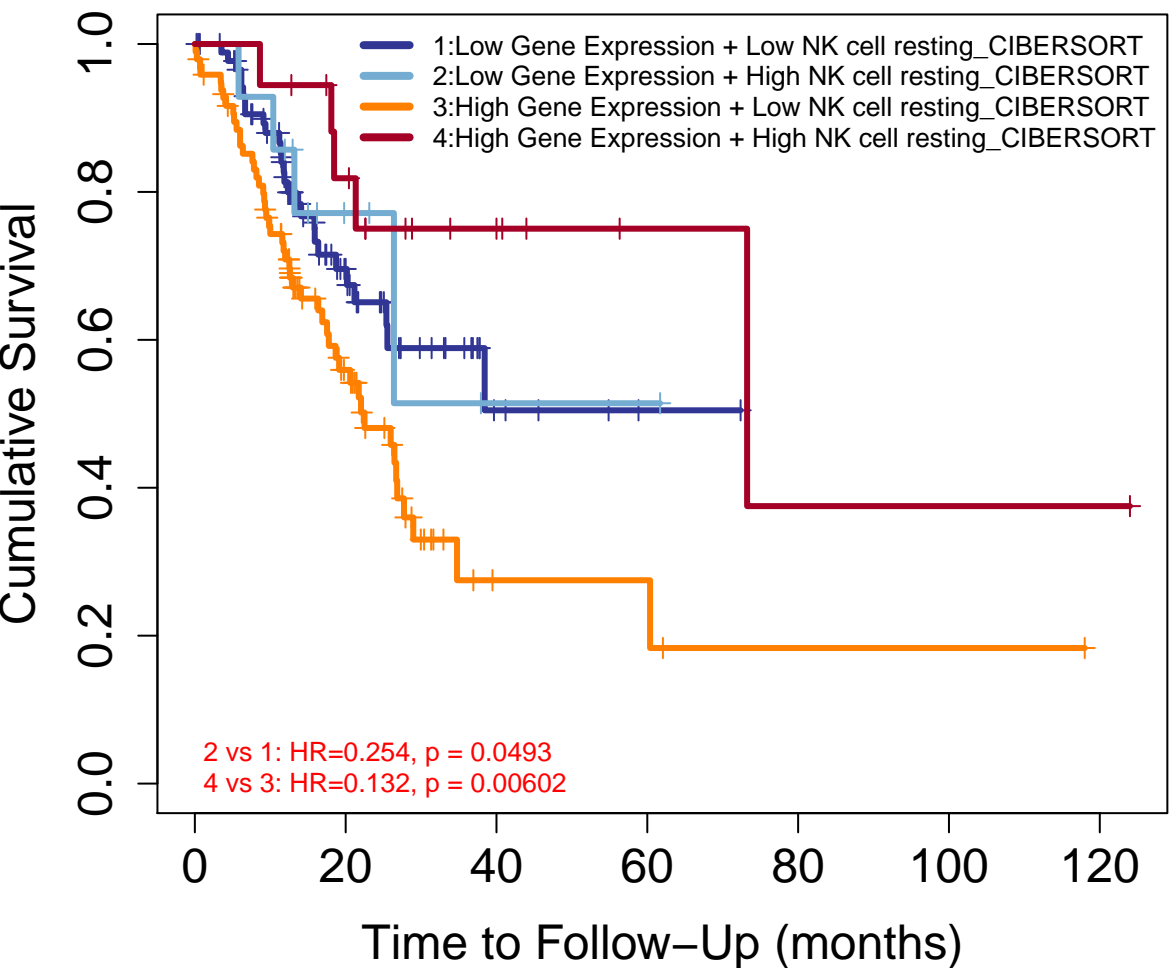

Supplement: Supplementary file 12 [file Data_Sheet_12.ZIP › Supplementary materials fig.11,12úa13/Supplementary materials fig.13/FAM83D/NK/outcome_plot.pdf]

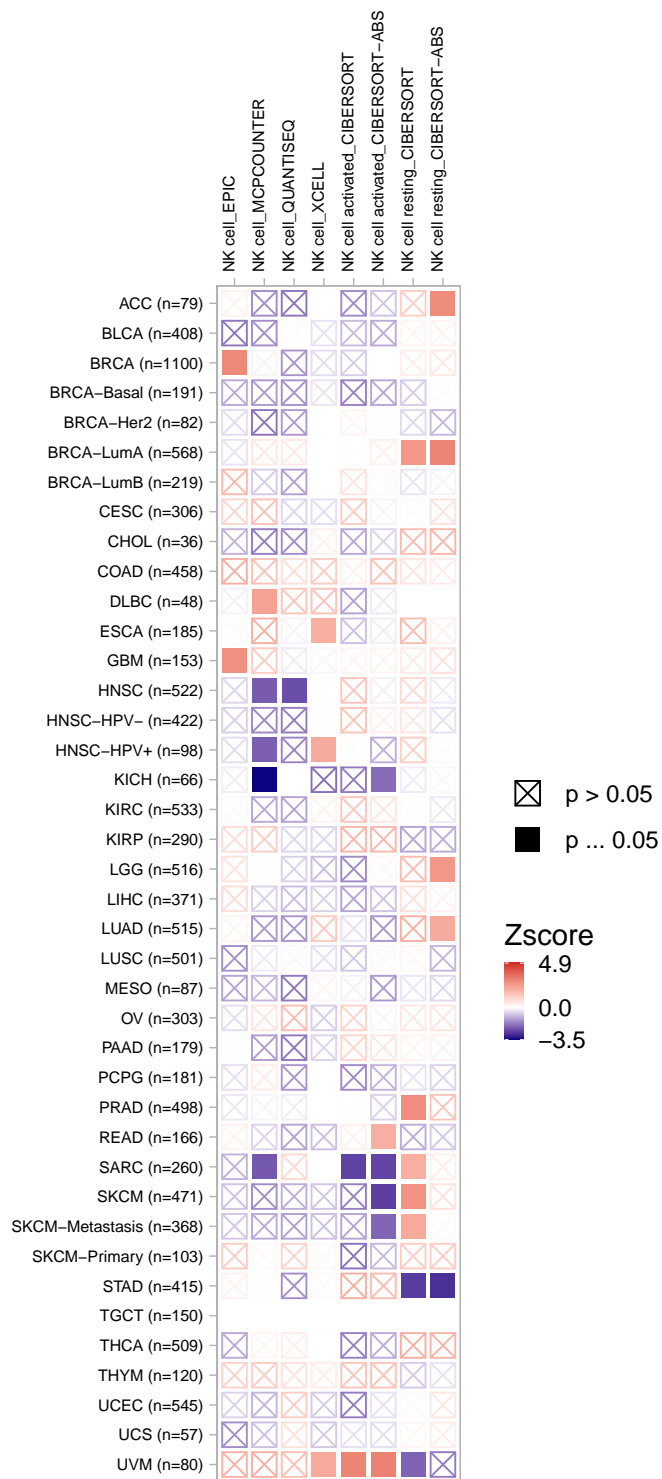

Supplement: Supplementary file 12 [file Data_Sheet_12.ZIP › Supplementary materials fig.11,12úa13/Supplementary materials fig.13/FAM83D/NK/outcome_table.pdf]

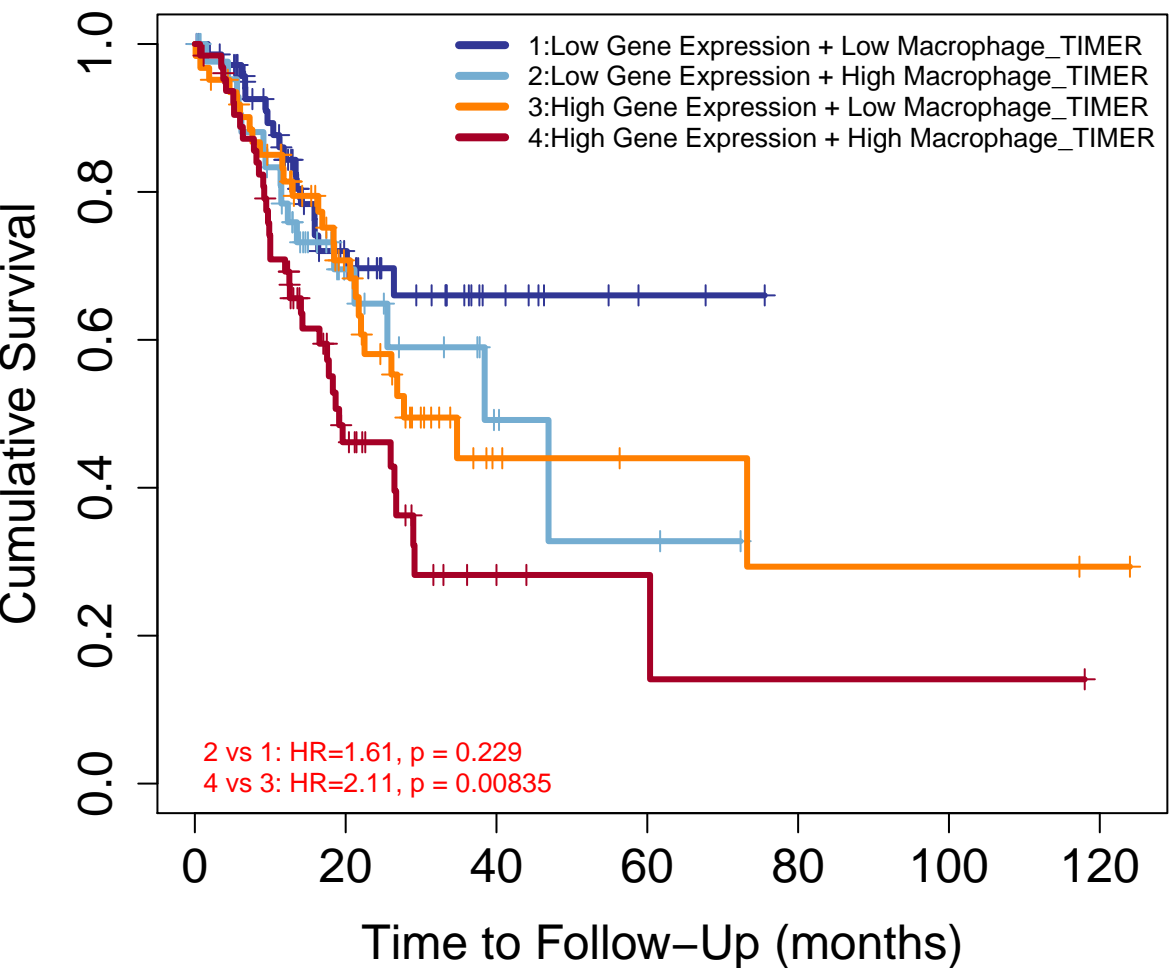

Supplement: Supplementary file 12 [file Data_Sheet_12.ZIP › Supplementary materials fig.11,12úa13/Supplementary materials fig.13/FAM83D/macrophage/outcome_plot.pdf]

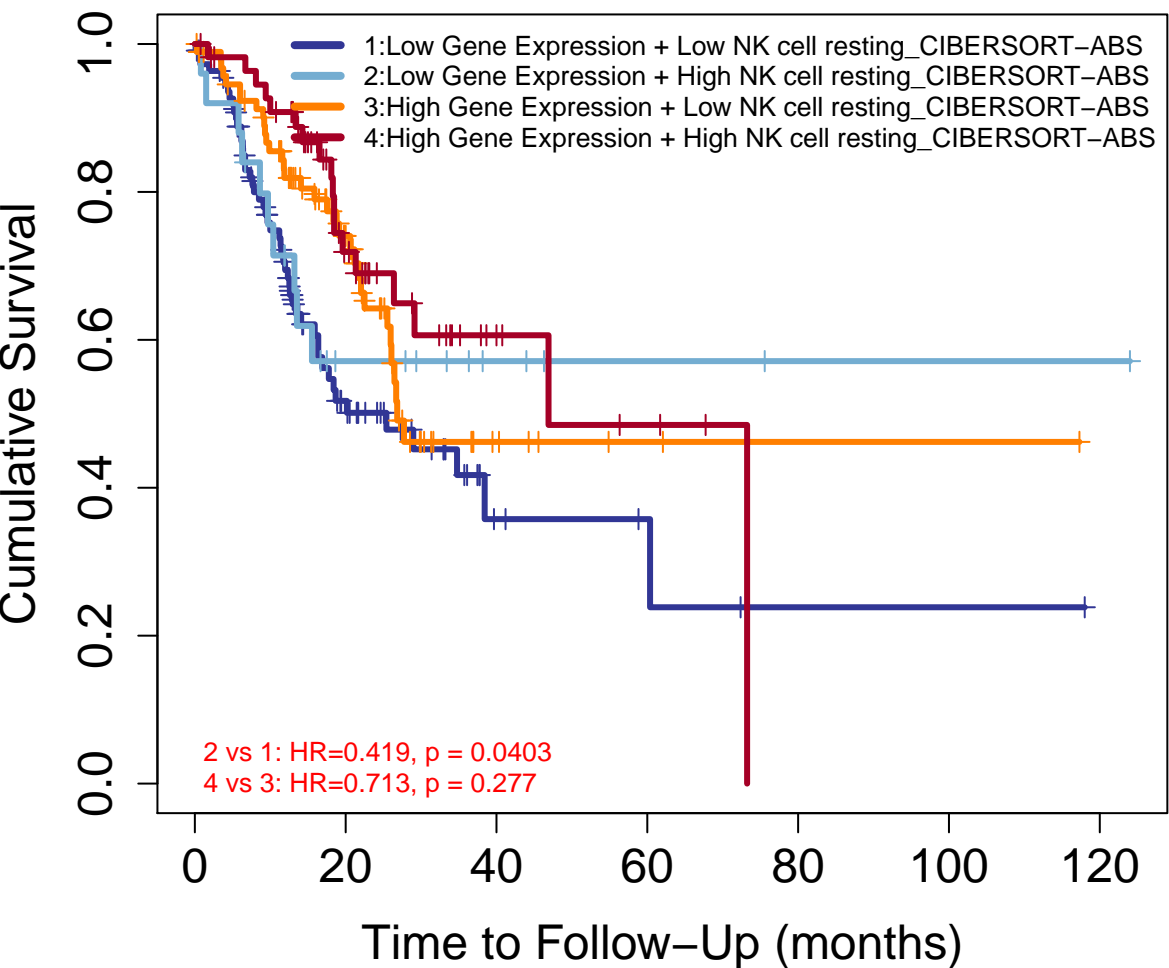

Supplement: Supplementary file 12 [file Data_Sheet_12.ZIP › Supplementary materials fig.11,12úa13/Supplementary materials fig.13/FAM83G/NK/outcome_plot.pdf]

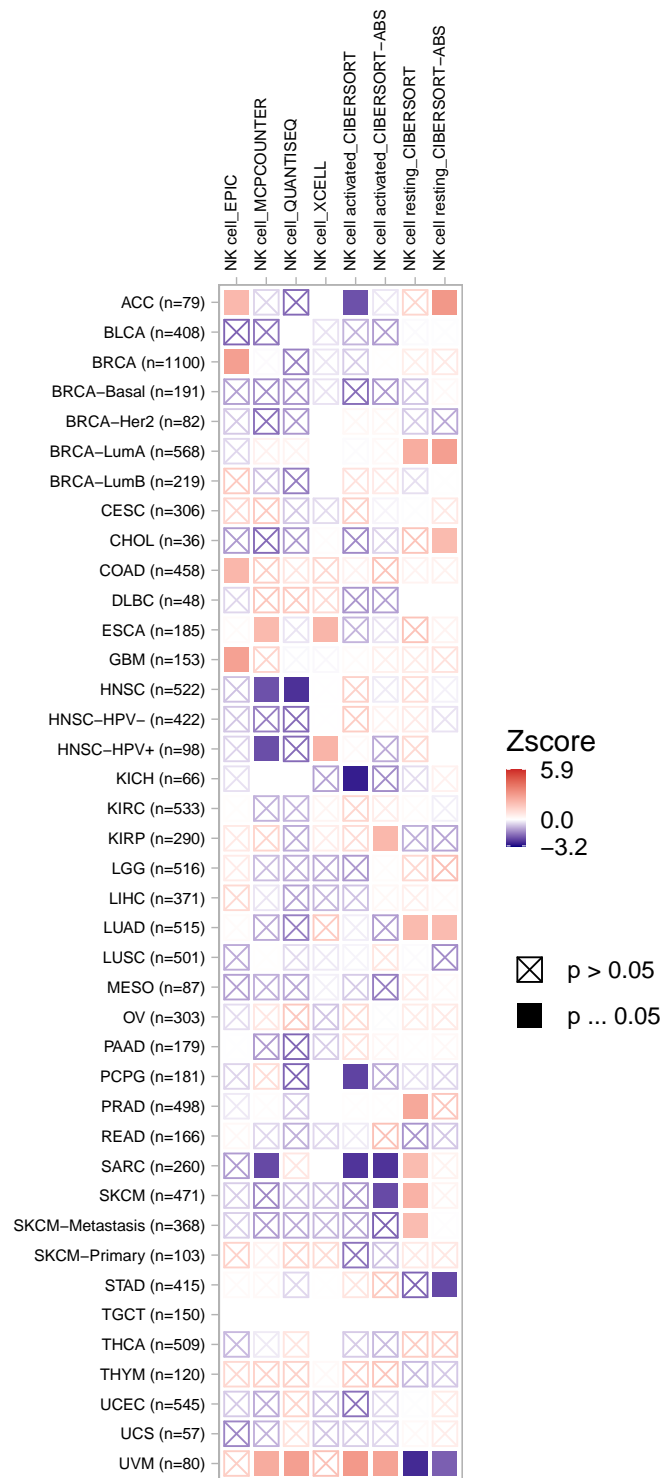

Supplement: Supplementary file 12 [file Data_Sheet_12.ZIP › Supplementary materials fig.11,12úa13/Supplementary materials fig.13/FAM83G/NK/outcome_table.pdf]

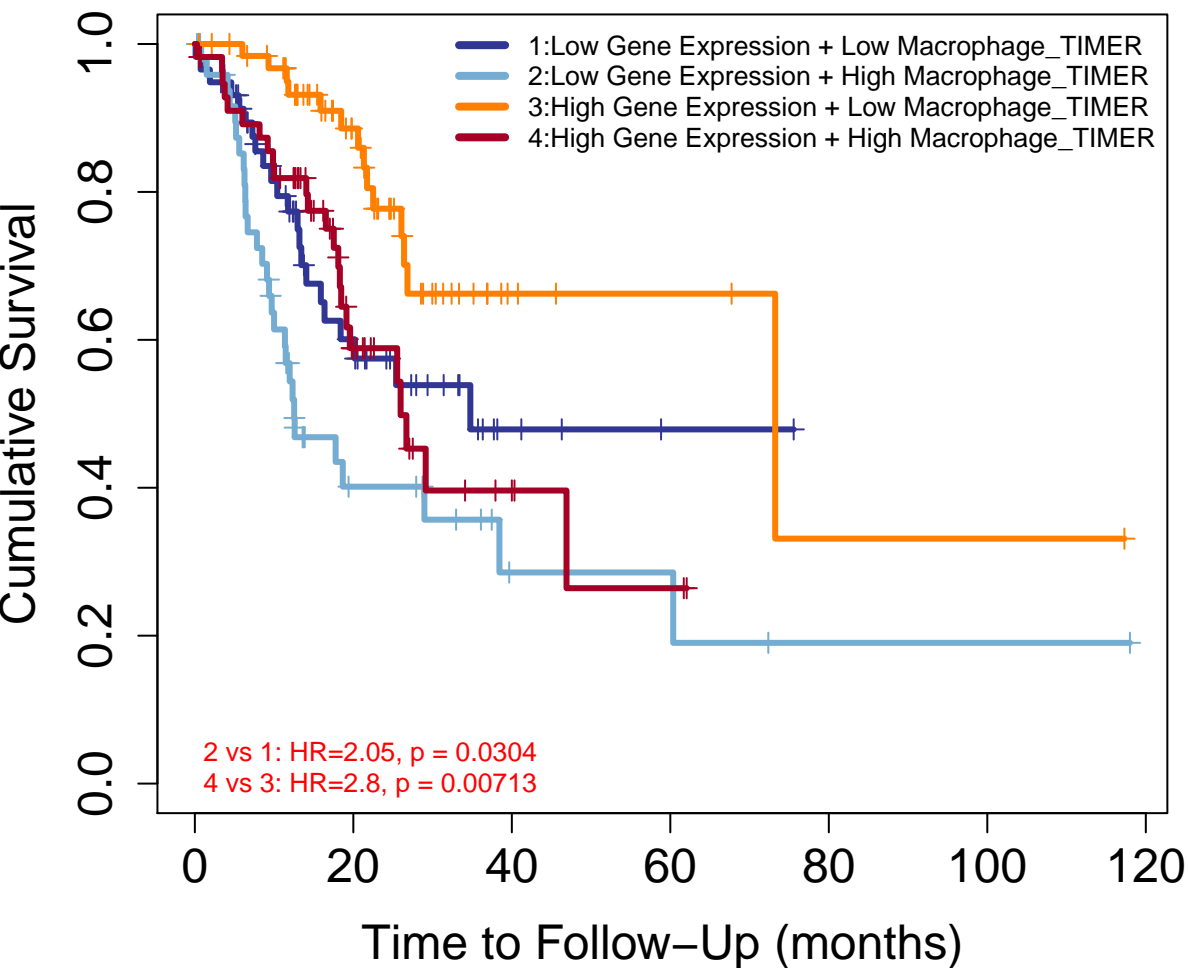

Supplement: Supplementary file 12 [file Data_Sheet_12.ZIP › Supplementary materials fig.11,12úa13/Supplementary materials fig.13/FAM83G/macrophage/outcome_plot.pdf]

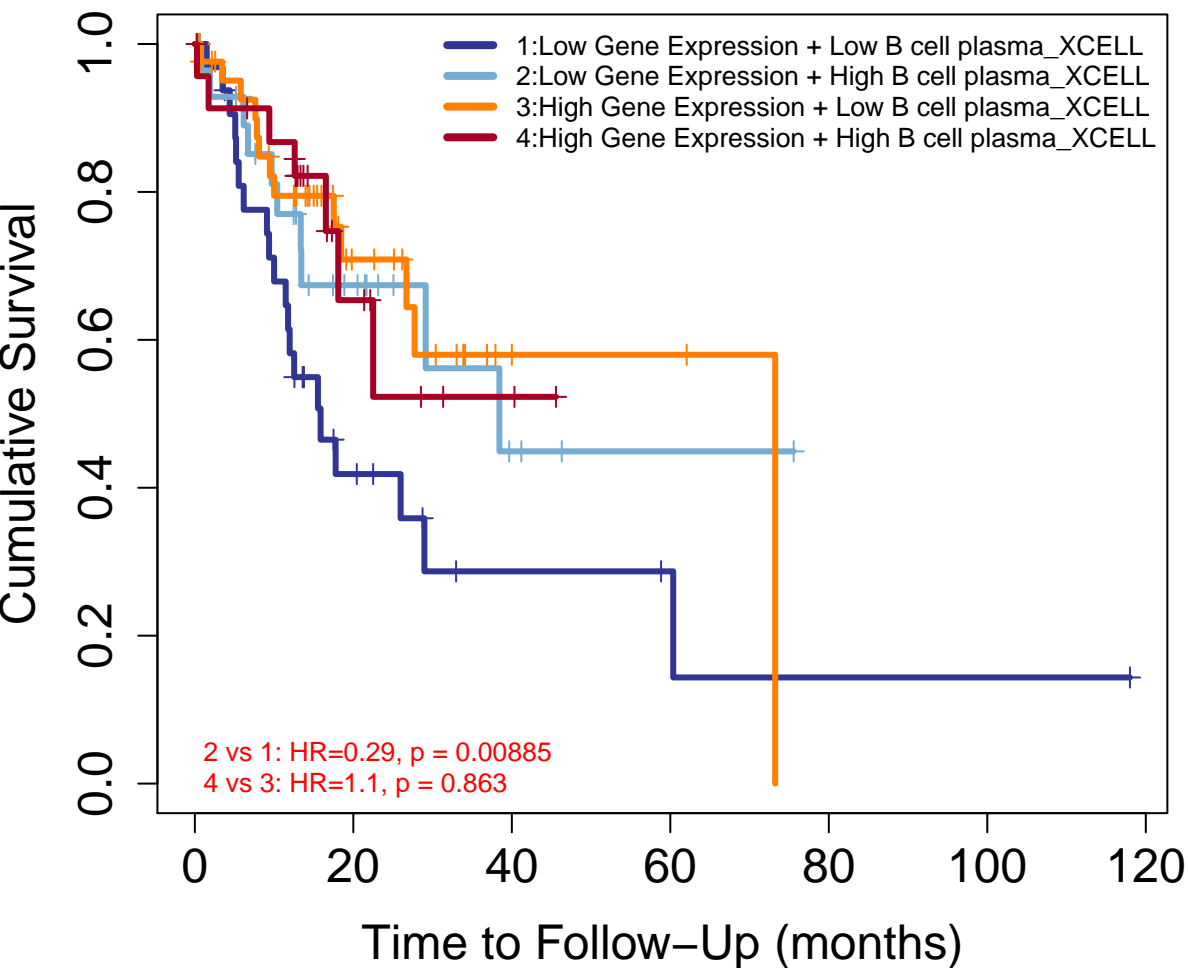

Supplement: Supplementary file 12 [file Data_Sheet_12.ZIP › Supplementary materials fig.11,12úa13/Supplementary materials fig.13/FAM83H/B/outcome_plot.pdf]

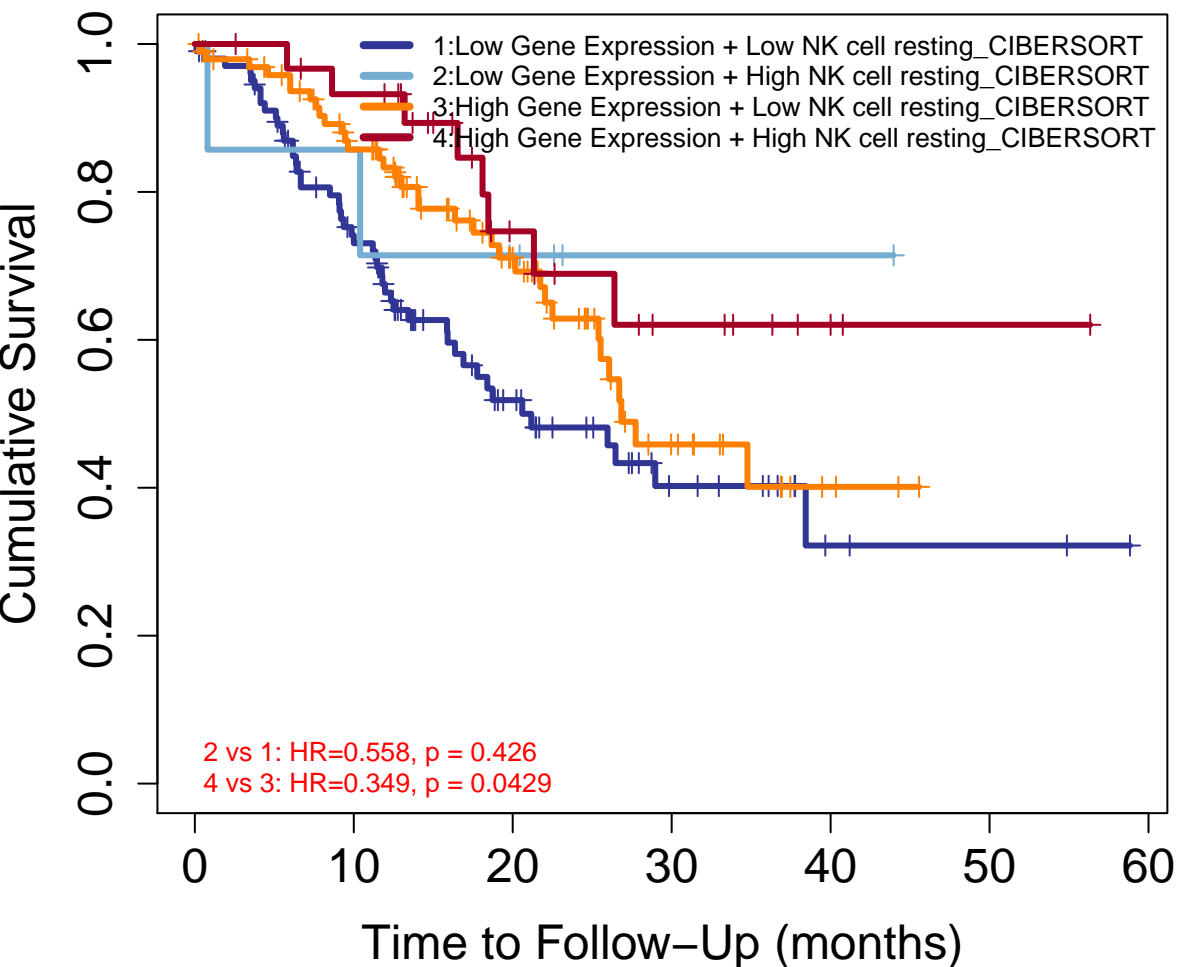

Supplement: Supplementary file 12 [file Data_Sheet_12.ZIP › Supplementary materials fig.11,12úa13/Supplementary materials fig.13/FAM83H/NK/outcome_plot(1).pdf]

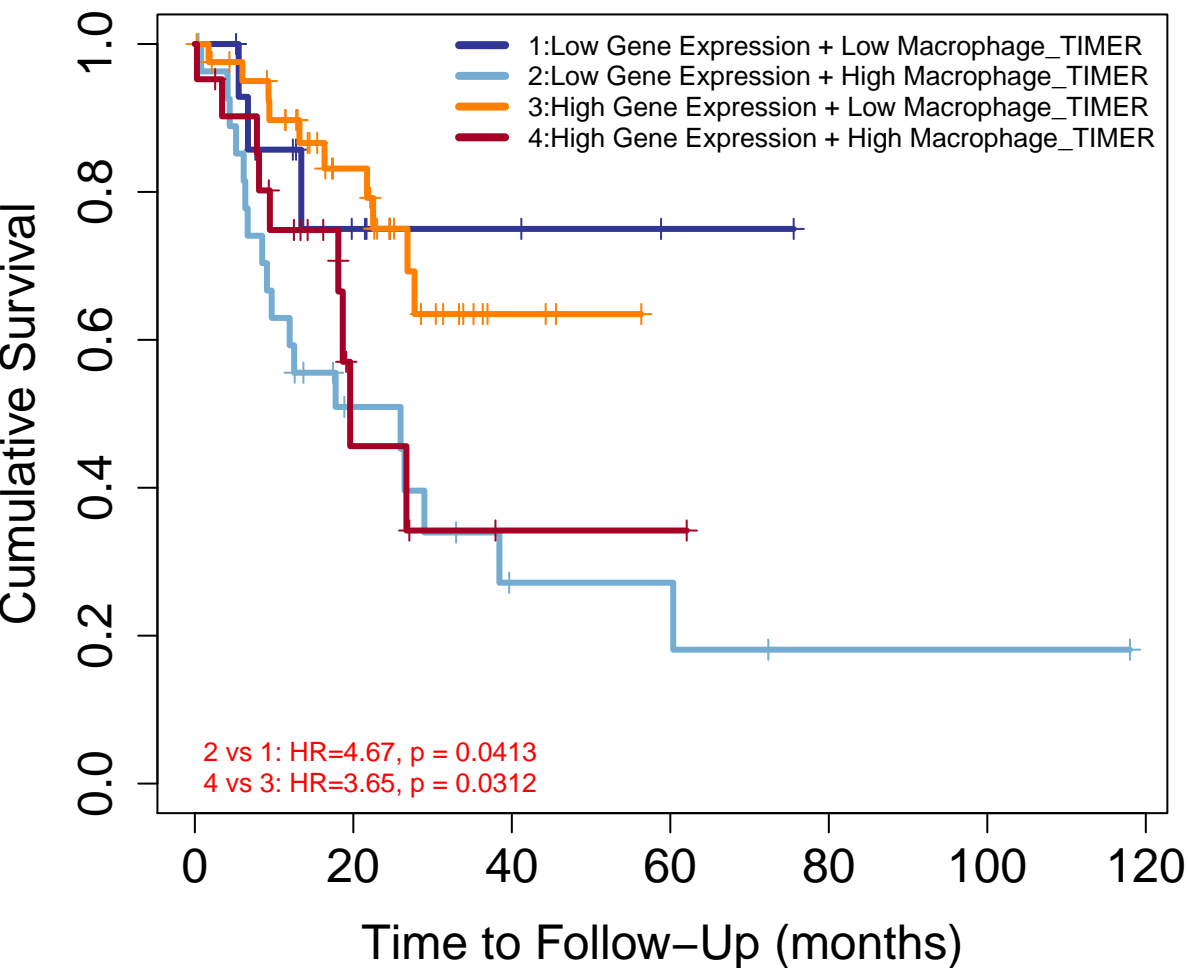

Supplement: Supplementary file 12 [file Data_Sheet_12.ZIP › Supplementary materials fig.11,12úa13/Supplementary materials fig.13/FAM83H/macrophage/outcome_plot.pdf]

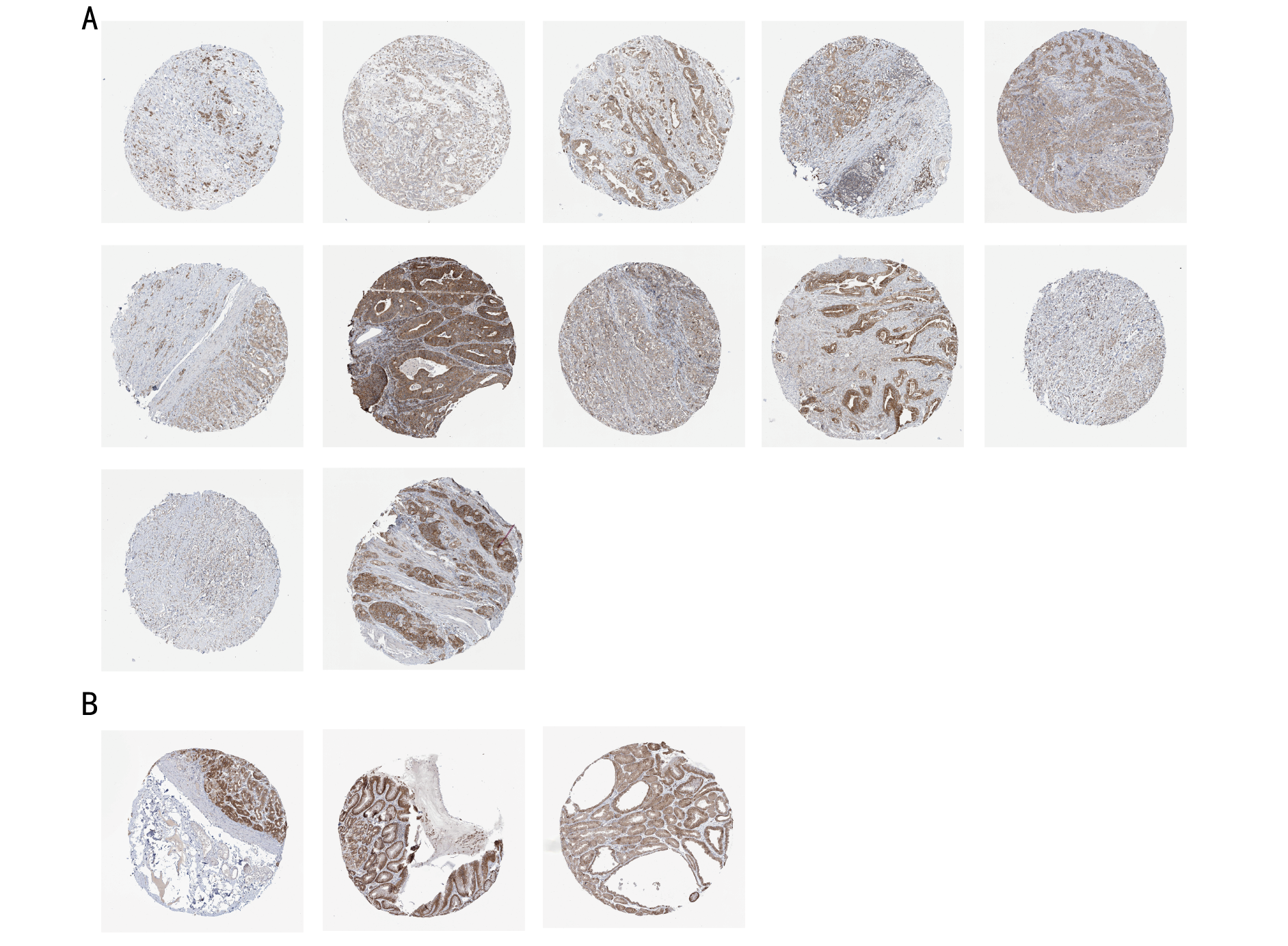

Supplement: Supplementary file 14 [file Data_Sheet_14.zip › Supplementary materials of fig.3 (IHC)/FAM83B.tif]

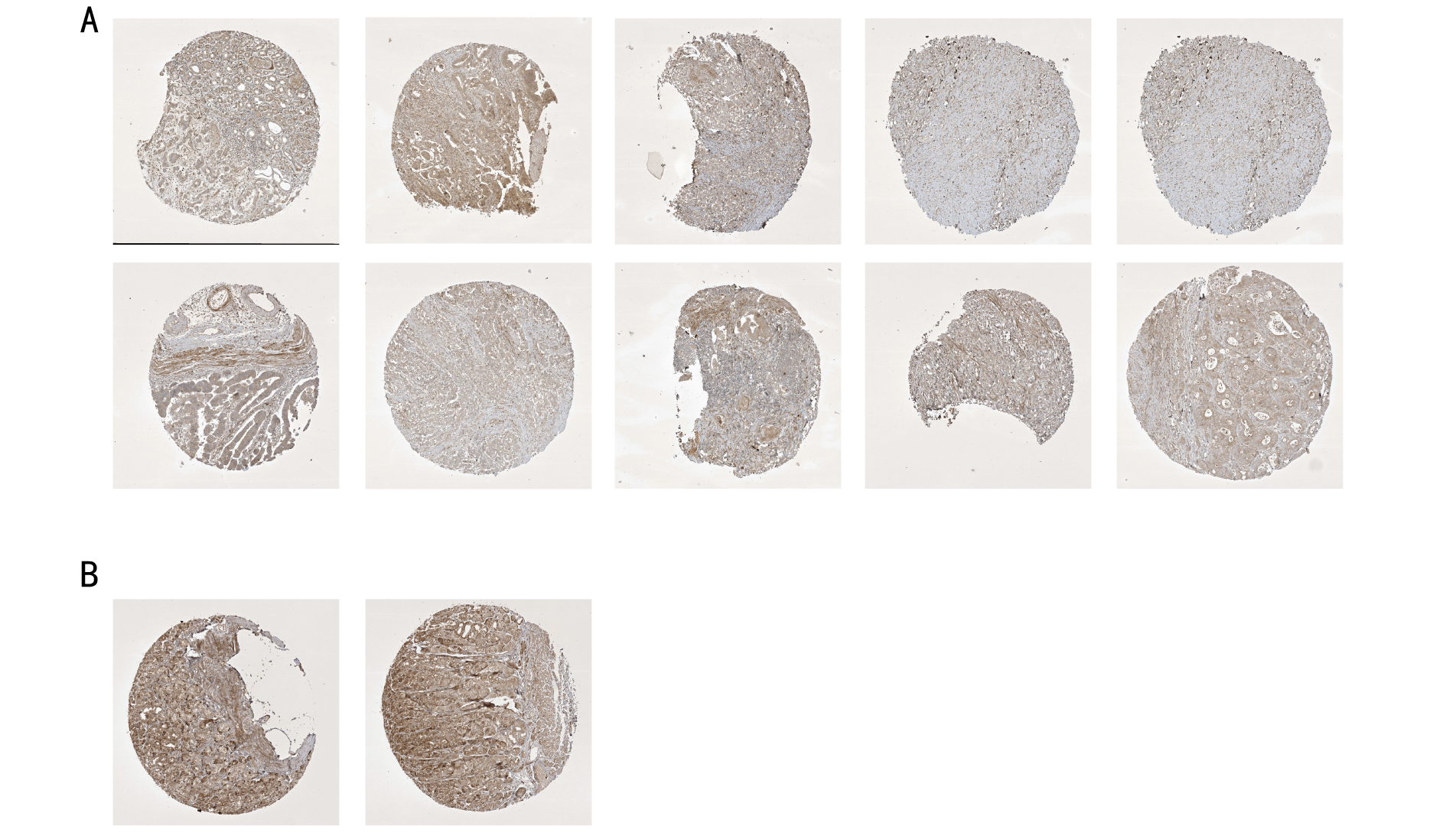

Supplement: Supplementary file 14 [file Data_Sheet_14.zip › Supplementary materials of fig.3 (IHC)/FAM83C.tif]

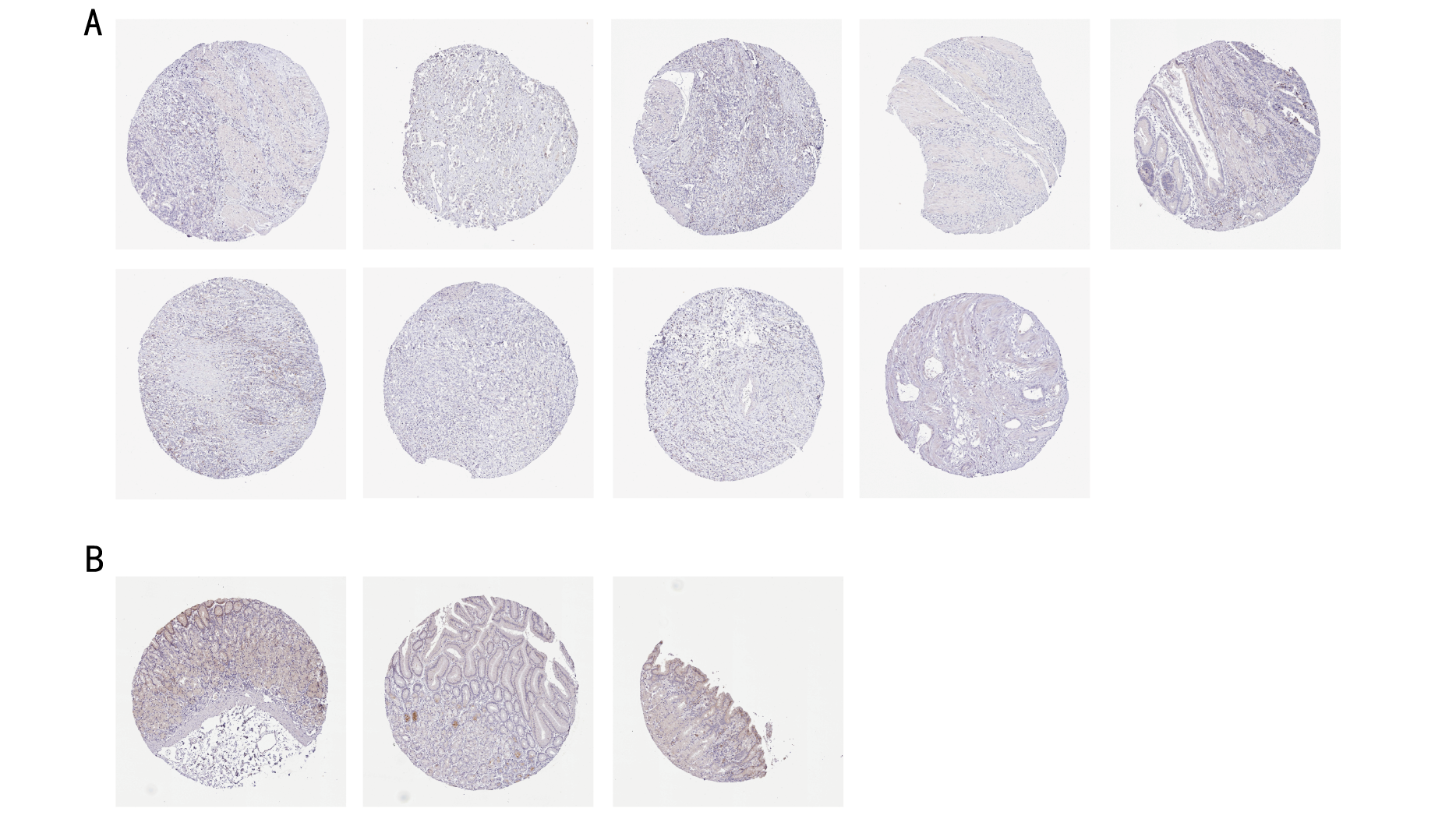

Supplement: Supplementary file 14 [file Data_Sheet_14.zip › Supplementary materials of fig.3 (IHC)/FAM83E.tif]

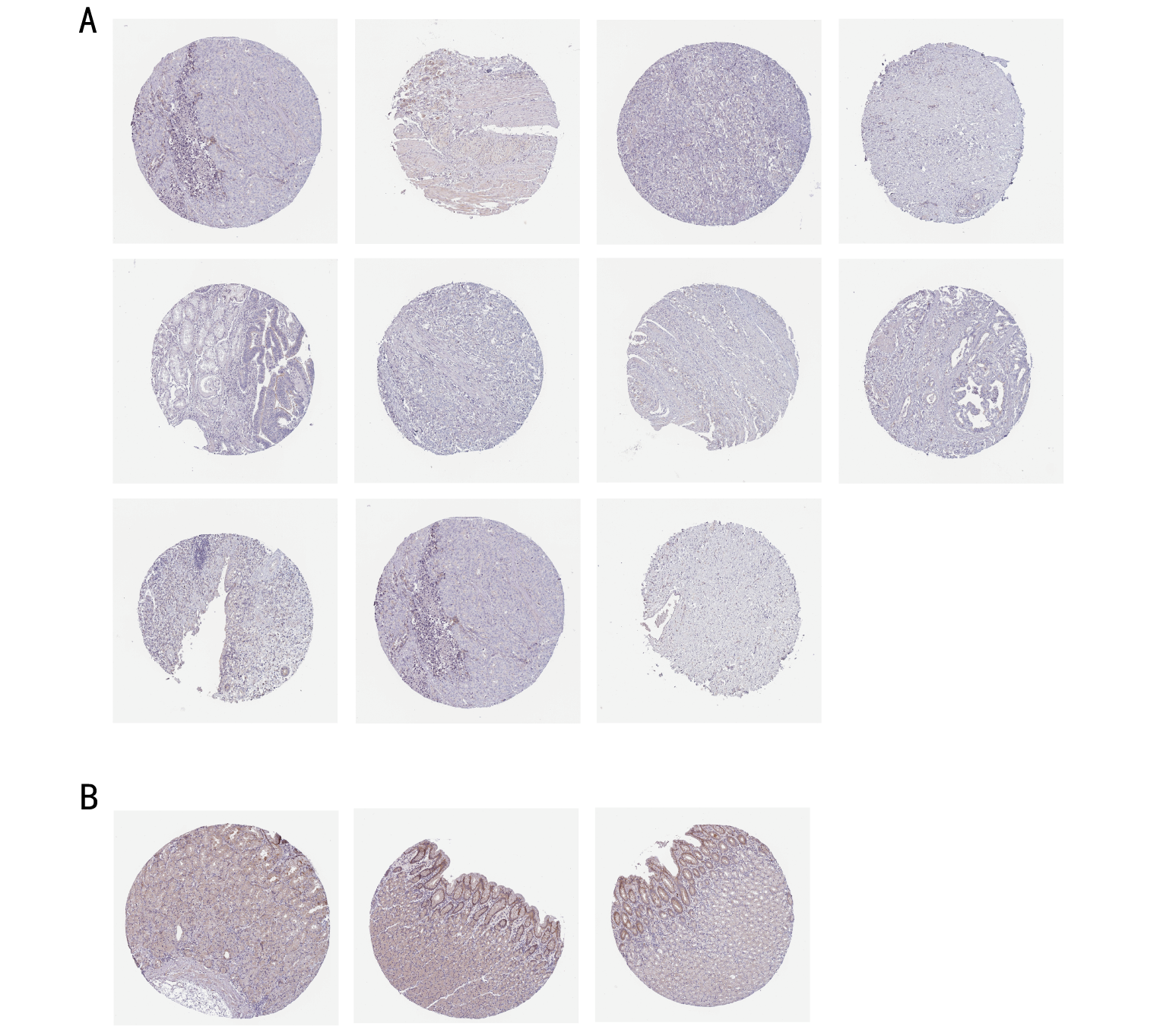

Supplement: Supplementary file 14 [file Data_Sheet_14.zip › Supplementary materials of fig.3 (IHC)/FAM83F.tif]

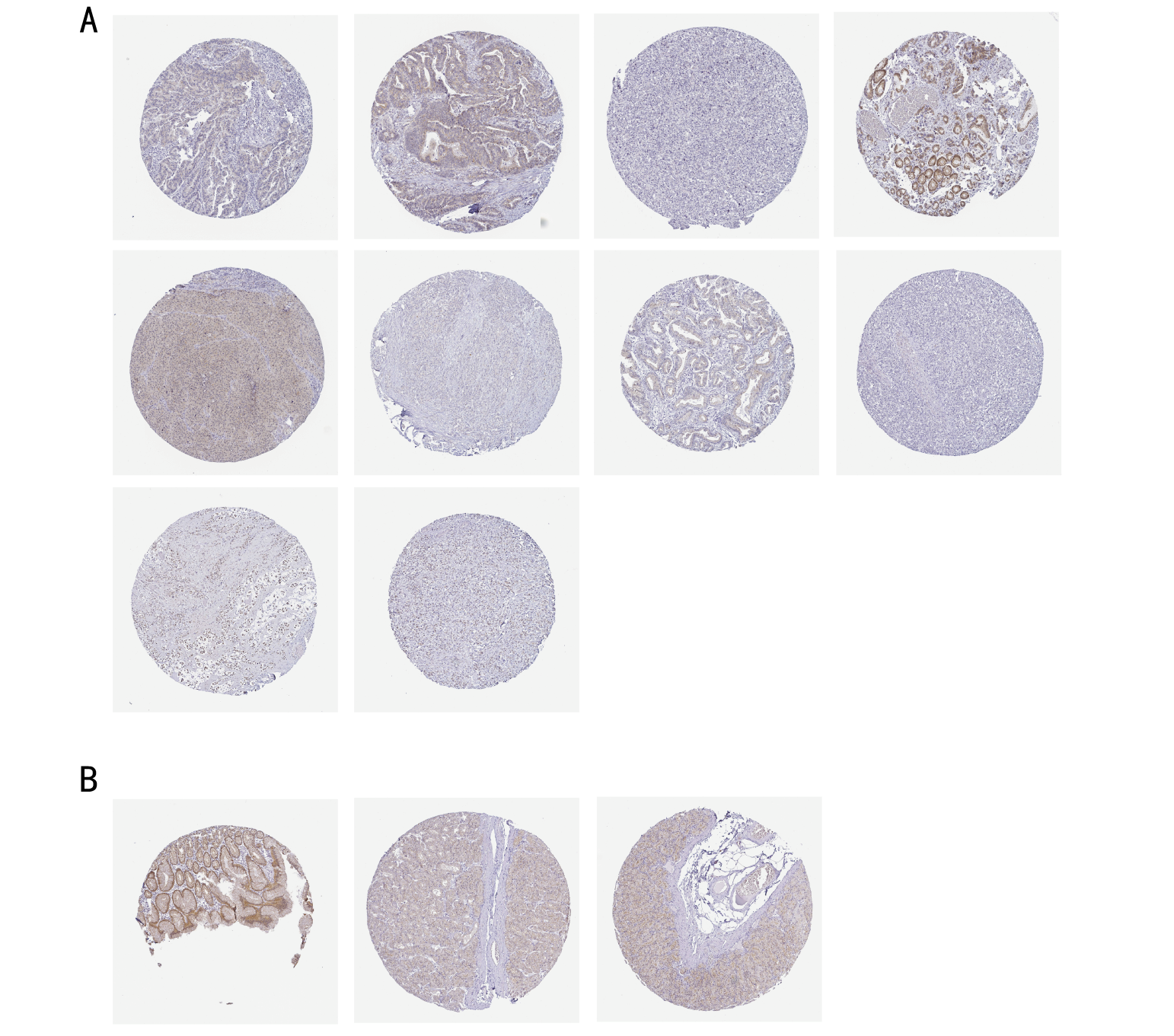

Supplement: Supplementary file 14 [file Data_Sheet_14.zip › Supplementary materials of fig.3 (IHC)/FAM83G.tif]

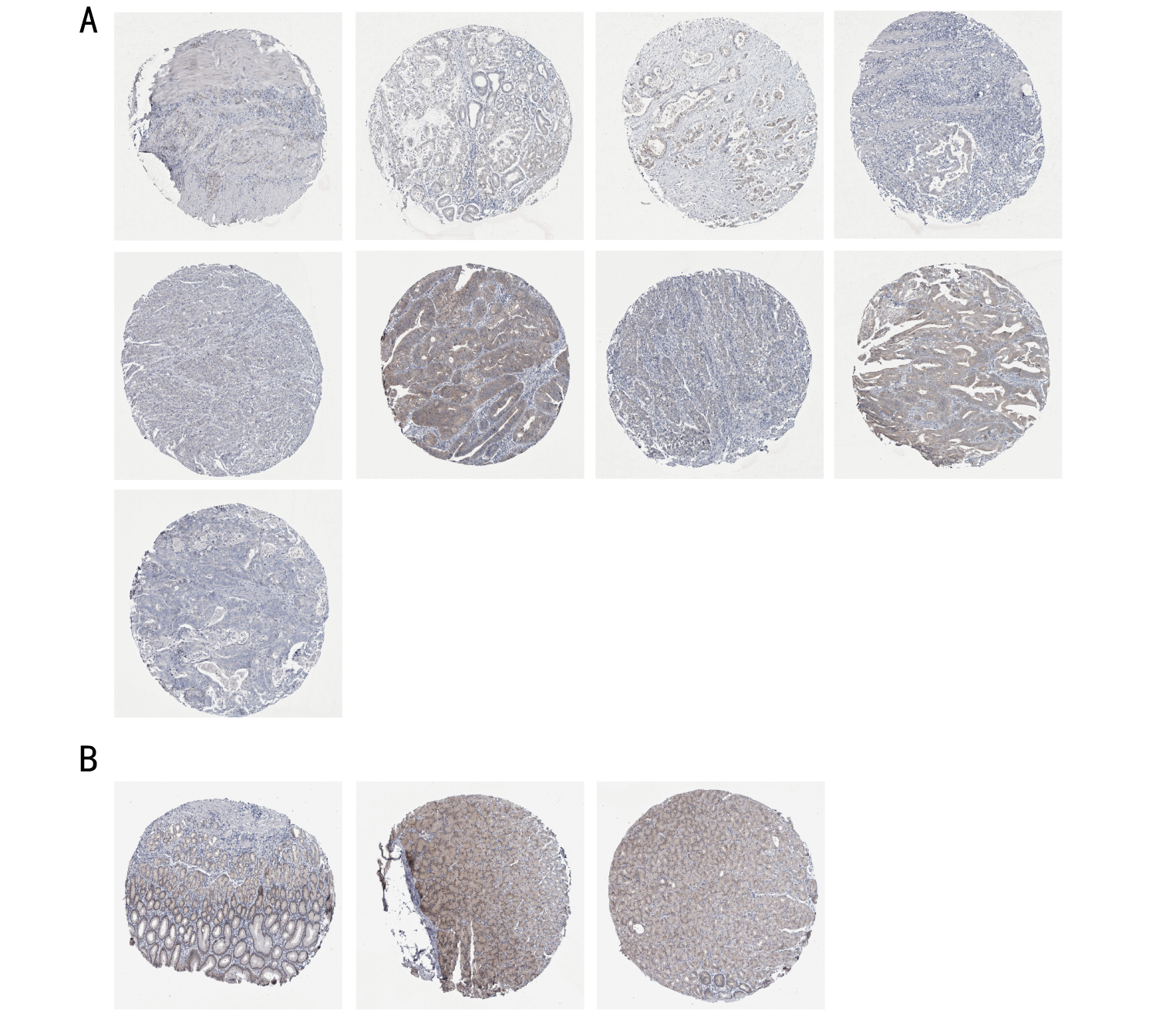

Supplement: Supplementary file 14 [file Data_Sheet_14.zip › Supplementary materials of fig.3 (IHC)/FAM83H.tif]
