# Supplementary material for: Comprehensive Analysis and Identification of Prognostic Biomarkers and Therapeutic Targets Among FAM83 Family Members for Gastric Cancer
Source: Front Cell Dev Biol. 2021 Nov 19;9:719613. doi: 10.3389/fcell.2021.719613 (PMC8640971; doi:10.3389/fcell.2021.719613)
Supplement: Supplementary file 12 [file Data_Sheet_12.ZIP › Supplementary materials fig.11,12úa13/Supplementary materials fig.12/FAM83C/gene_plot(2).pdf]

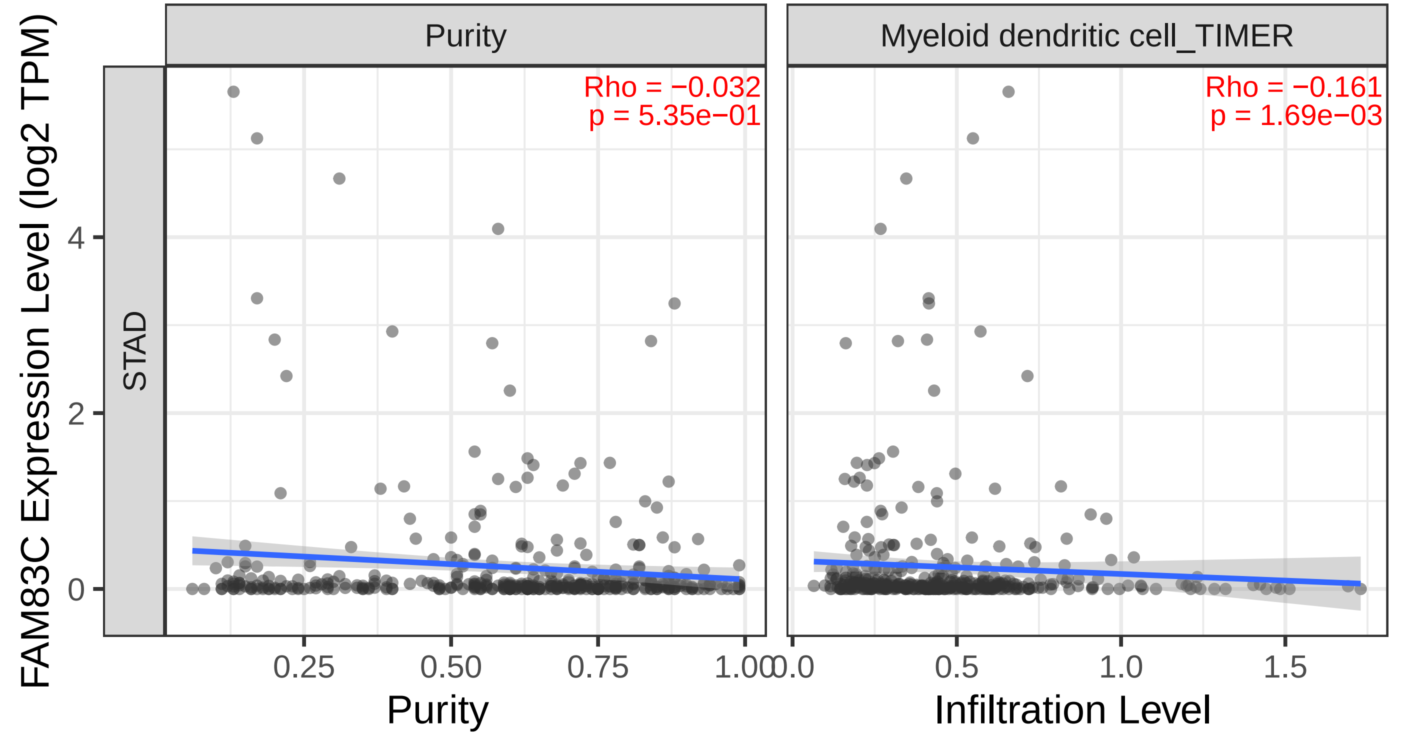

Purity
Myeloid dendritic cell_TIMER
STAD
Rho = −0.032
p = 5.35e−01
Rho = −0.161
p = 1.69e−03
FAM83C Expression Level (log2 TPM)
0
2
4
0.25
0.50
0.75
1.00
0.0
0.5
1.0
1.5
Purity
Infiltration Level
