# Supplementary material for: Comprehensive Analysis and Identification of Prognostic Biomarkers and Therapeutic Targets Among FAM83 Family Members for Gastric Cancer
Source: Front Cell Dev Biol. 2021 Nov 19;9:719613. doi: 10.3389/fcell.2021.719613 (PMC8640971; doi:10.3389/fcell.2021.719613)
Supplement: Supplementary file 12 [file Data_Sheet_12.ZIP › Supplementary materials fig.11,12úa13/Supplementary materials fig.12/FAM83G/gene_plot(3).pdf]

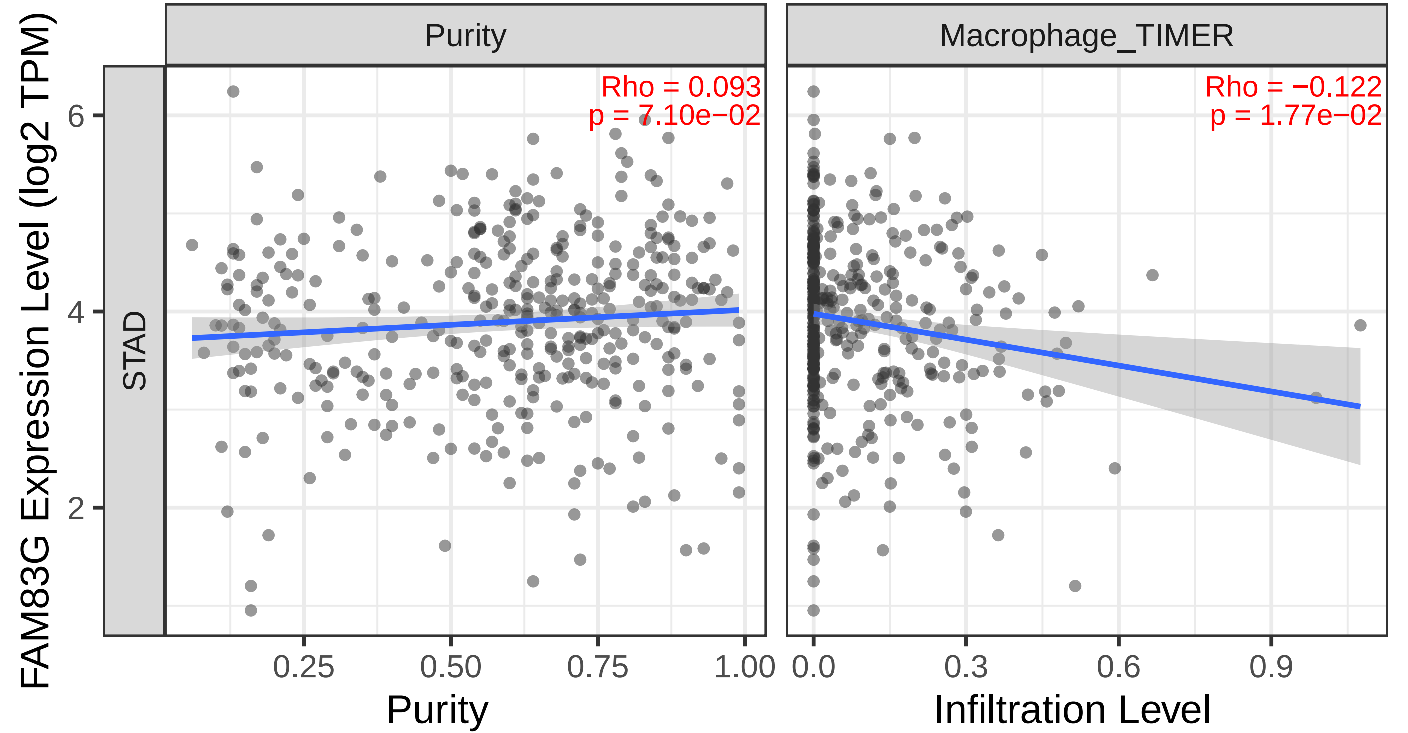

Purity
Macrophage_TIMER
Rho = 0.093
p = 7.10e−02
Rho = −0.122
p = 1.77e−02
STAD
FAM83G Expression Level (log2 TPM)
6
4
2
0.25
0.50
0.75
1.00
0.0
0.3
0.6
0.9
Purity
Infiltration Level
