# Supplementary material for: Comprehensive Analysis and Identification of Prognostic Biomarkers and Therapeutic Targets Among FAM83 Family Members for Gastric Cancer
Source: Front Cell Dev Biol. 2021 Nov 19;9:719613. doi: 10.3389/fcell.2021.719613 (PMC8640971; doi:10.3389/fcell.2021.719613)
Supplement: Supplementary file 14 [file Data_Sheet_14.zip › Supplementary materials of fig.3 (IHC)/figure legend.docx]

These figures were used to show the IHC analysis of FAM83B/C/E/F/G/H with protein expression levals. (A＆B) Differentially expressed proteins of FAM83B/C/E/F/G/H in gastric cancer tissues (A) and normal tissues (B) in The Human Protein Atlas database. The small image in each large figure represents a sample of patient.
