# Supplementary material for: Comprehensive Analysis and Identification of Prognostic Biomarkers and Therapeutic Targets Among FAM83 Family Members for Gastric Cancer
Source: Front Cell Dev Biol. 2021 Nov 19;9:719613. doi: 10.3389/fcell.2021.719613 (PMC8640971; doi:10.3389/fcell.2021.719613)
Supplement: Supplementary file 12 [file outcome_table.pdf › Supplementary materials fig.11,12úa13/Supplementary materials fig.13/FAM83H/B/outcome_table.pdf]

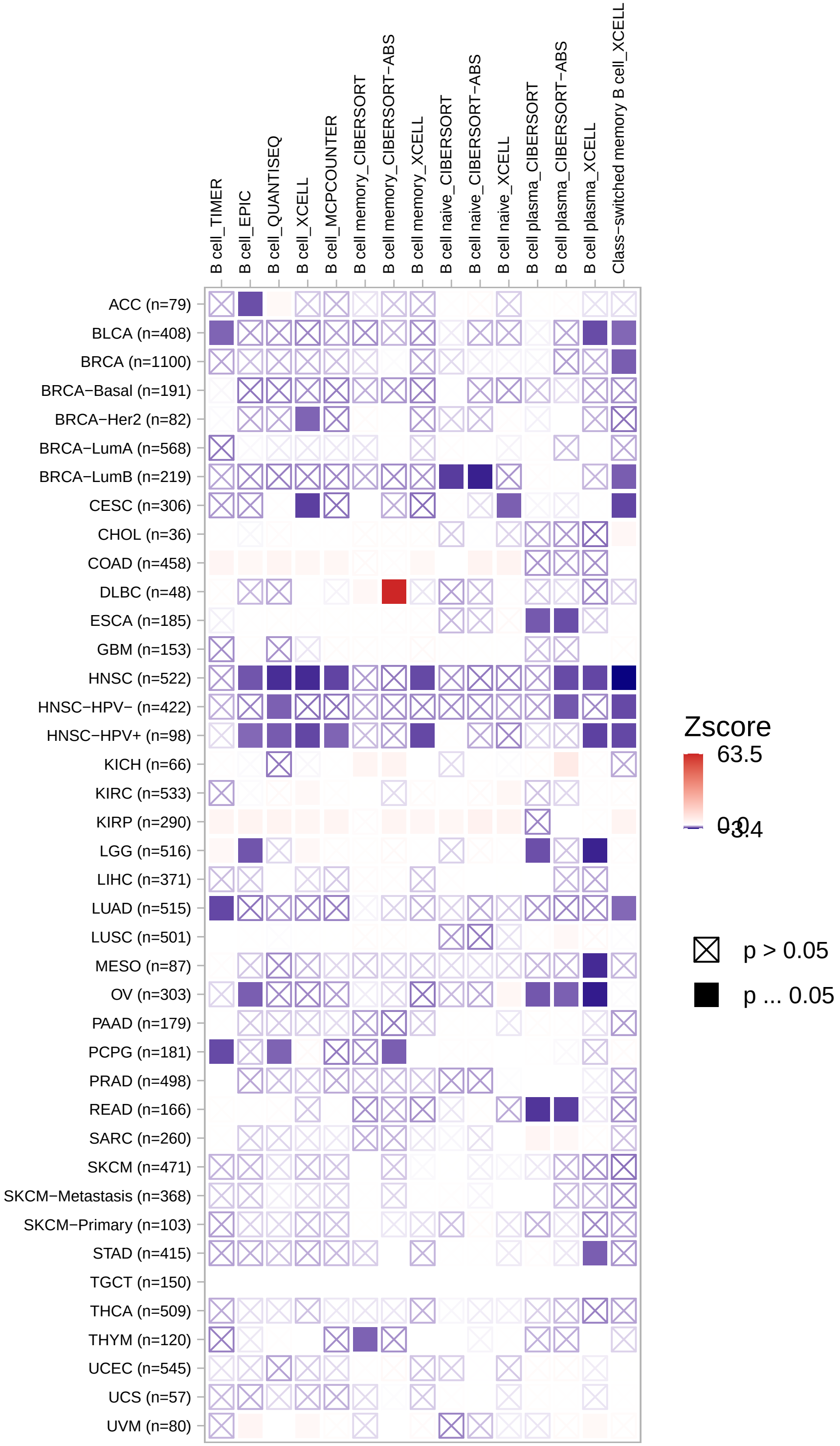

B cell_TIMER
B cell_EPIC
B cell_QUANTISEQ
B cell_XCELL
B cell_MCPCOUNTER
B cell memory_CIBERSORT
B cell memory_CIBERSORT−ABS
B cell memory_XCELL
B cell naive_CIBERSORT
B cell naive_CIBERSORT−ABS
B cell naive_XCELL
B cell plasma_CIBERSORT
B cell plasma_CIBERSORT−ABS
B cell plasma_XCELL
Class−switched memory B cell_XCELL
ACC (n=79)
BLCA (n=408)
BRCA (n=1100)
BRCA−Basal (n=191)
BRCA−Her2 (n=82)
BRCA−LumA (n=568)
BRCA−LumB (n=219)
CESC (n=306)
CHOL (n=36)
COAD (n=458)
DLBC (n=48)
ESCA (n=185)
GBM (n=153)
HNSC (n=522)
HNSC−HPV− (n=422)
HNSC−HPV+ (n=98)
KICH (n=66)
KIRC (n=533)
KIRP (n=290)
LGG (n=516)
LIHC (n=371)
LUAD (n=515)
LUSC (n=501)
MESO (n=87)
OV (n=303)
PAAD (n=179)
PCPG (n=181)
PRAD (n=498)
READ (n=166)
SARC (n=260)
SKCM (n=471)
SKCM−Metastasis (n=368)
SKCM−Primary (n=103)
STAD (n=415)
TGCT (n=150)
THCA (n=509)
THYM (n=120)
UCEC (n=545)
UCS (n=57)
UVM (n=80)
Zscore
63.5
0.0
−3.4
p > 0.05
p ... 0.05
